# Supplementary material for: Identification of the active mechanism of aminoglycoside entry in V. cholerae through characterization of sRNA ctrR, regulating carbohydrate utilization and transport
Source: bioRxiv. 2023 Jul 19:2023.07.19.549712. Preprint. [Version 1] doi: 10.1101/2023.07.19.549712 (PMC10370196; doi:10.1101/2023.07.19.549712)
Supplement: Supplement 1 [file media-1.pdf]

Supplementary figures:

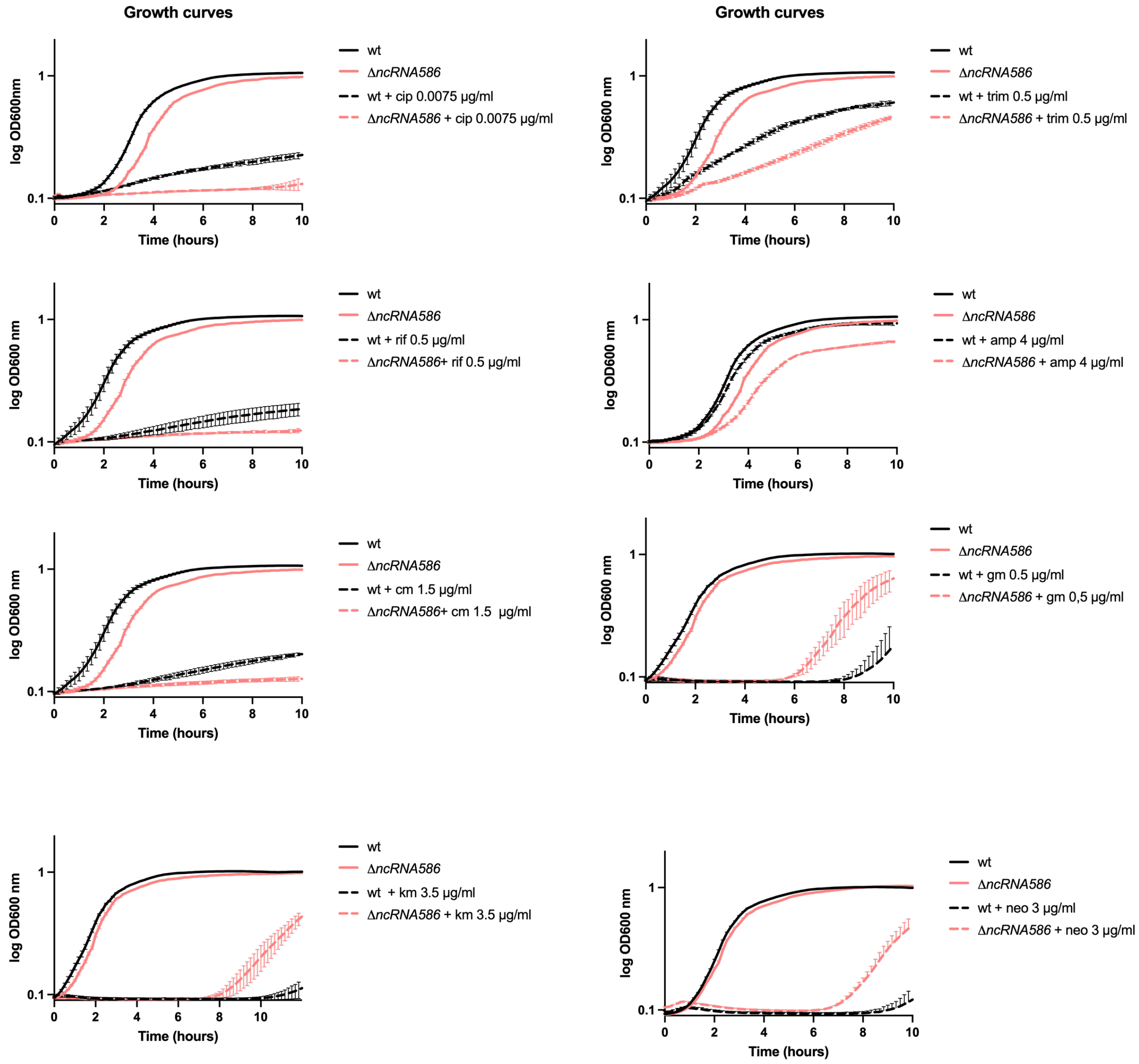

Figure S1: ncRNA586 effect on antibiotic susceptibility is specific to AGs. Growth in presence of antibiotics from five different classes (ciprofloxacin, rifampicin, chloramphenicol trimethoprim and ampicillin), or different aminoglycosides (gentamicin, kanamycin and and neomycin 3 µg/ml)**.**

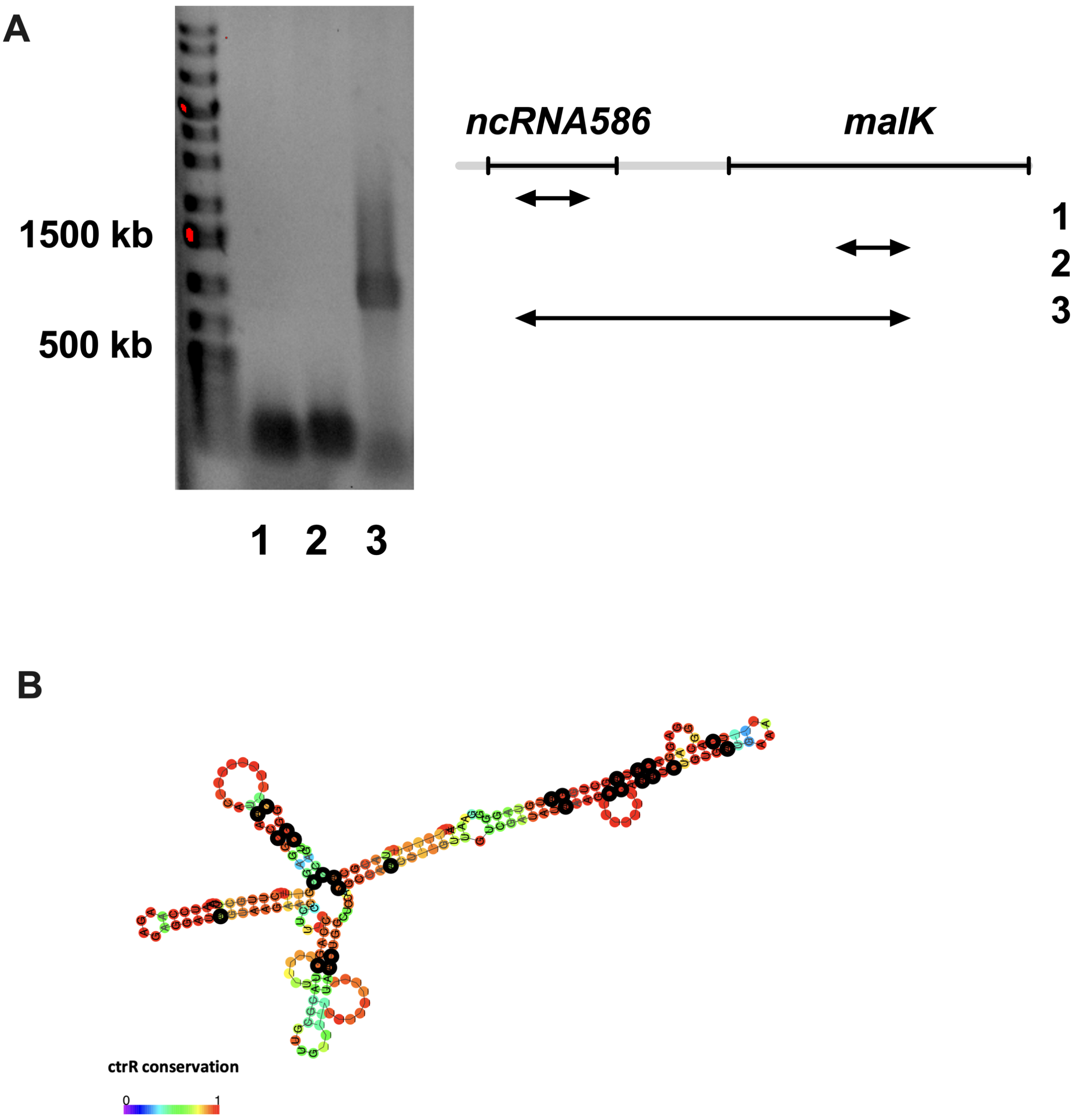

Figure S2: A. PCR on total *V. cholerae* RNA (following retrotranscription to obtain cDNA), using primers that bind *ncRNA586* (expected 88 pb amplicon) (1), *malK* (expected 162 pb amplicon) (2) and that bind *ncRNA586* in 5' and *malK* in 3' (expected 968 pb amplicon) (3). **B.** Conserved secondary structure prediction between Vibrio species (*V. cholerae*, *V. mimicus*, *V. parahaemolyticus*, *V. vulnificus* and *V. tasmaniensis*). Base pairing probabilities are shown as a gradient of colors, green being the lowest (0) and red being the highest (1).

A

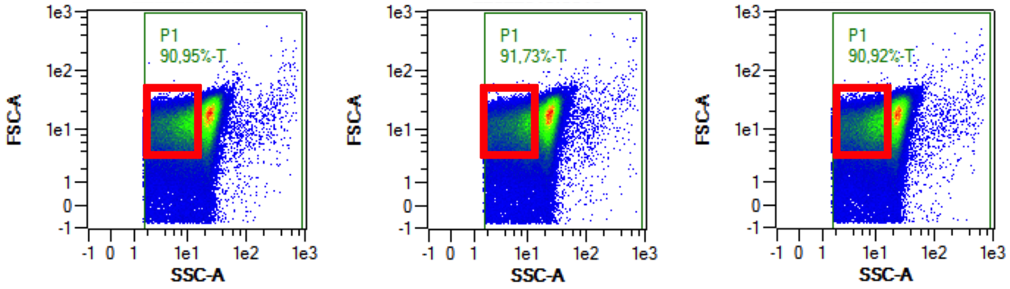

B

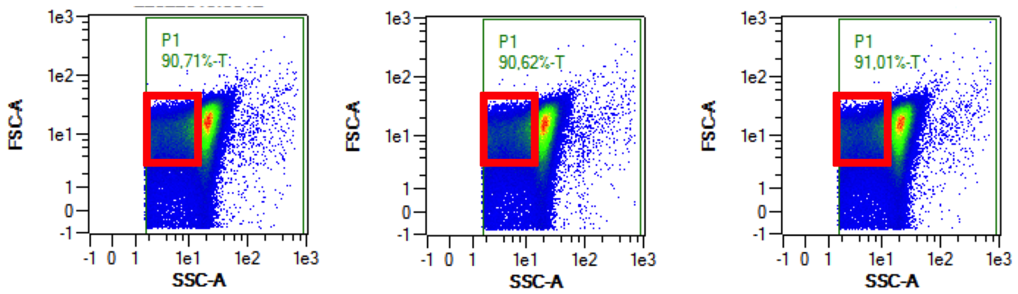

Figure S3: Altered cell morphology in the p*ctrR*+ strain (**A**) when compared to a control strain p0 (**B**). FSC (Forward scatter, X-axis) and SCC (Side scatter, Y-axis) parameters through FACS are shown. The populations of interest are highlighted by a red rectangle. Distribution of the p*ctrR*+ population reveals smaller cells that might be damaged by the antibiotic treatment as well as overexpression of the construct.

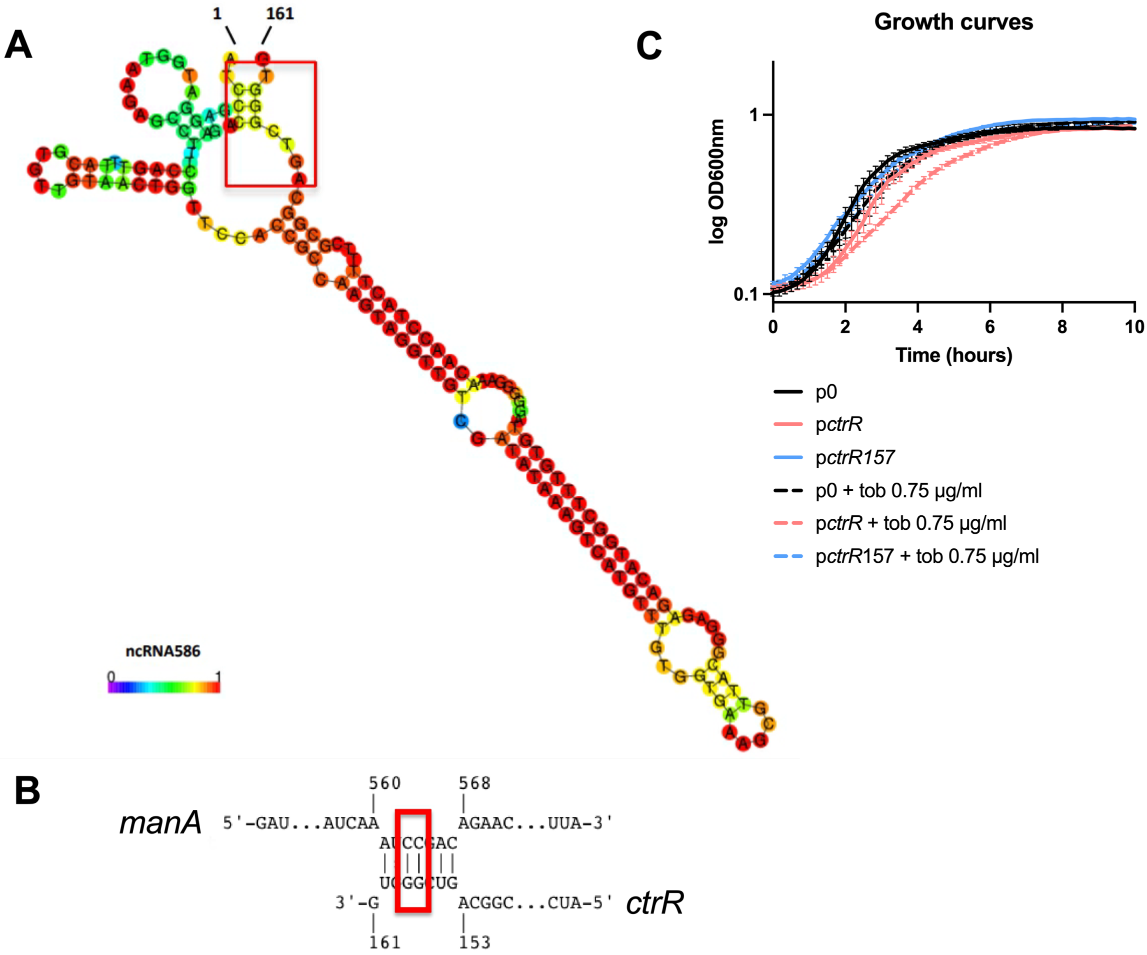

Figure S4: *ctrR* in predicted to base-paired with *manA* mRNA. A. Secondary structure of *ncRNA586* as predicted by RNAfold. The red square highlights the region predicted to interact with its putative targets. The first (1) and last (161) bases of ncRNA586 are shown. **B.** Region of interaction between *ctrR* and one of its targets (*manA*). The red square highlights the two nucleotides that were modified (GG to CC for *ctrR*, CC to GG for *manA*) to test the interaction between *ctrR* and *manA*. **C**. Growth curve of *V. cholerae* carrying the empty vector (p0), overexpressing *ctrR* (p*ctrR*+) or overexpressing *ctrR157* (p*ctr157*+), treated or not with tobramycin.

Supplementary tables:

Table S1: Differential genes expression of *V. cholerae* WT not treated vs upon sub-MIC tobramycin treatment.

Feature ID	Experiment - Fold Change (original values)	Experiment - Difference (normalized values)	Experiment - Fold Change (normalized values)	Baggerley's test: tob vs nt normalized values - Weighted proportions difference	Baggerley's test: tob vs nt normalized values - Weighted proportions fold change	Baggerley's test: tob vs nt normalized values - Test statistic	Baggerley's test: tob vs nt normalized values - P-value
VC1821	-19,363472	-228,37867	-22,503274	-0,0002229	-22,503274	-7,0741151	1,5044E-12
tRNA-Arg-6	-2,6773828	-28,059447	-2,820095	-2,738E-05	-2,820095	-6,2936553	3,101E-10
eno	-1,7718251	-920,41821	-1,8876542	-0,0008982	-1,8876542	-5,9821566	2,2021E-09
lamB	-5,5323333	-7697,9146	-4,4727473	-0,0075117	-4,4727473	-5,8368821	5,3191E-09
tatA	1,31832691	74,2071977	1,30224351	7,2412E-05	1,30224351	5,40688872	6,4131E-08
ctrR	-5,0071993	-8922,9306	-4,8225695	-0,0087071	-4,8225695	-5,3207913	1,0332E-07
VC1828	-17,722882	-202,11306	-19,025931	-0,0001972	-19,025931	-5,0081088	5,497E-07
VC0411	-1,5469315	-106,26349	-1,5062801	-0,0001037	-1,5062801	-4,9877325	6,1094E-07
VCA0392	1,36422326	49,4908324	1,38926508	4,8294E-05	1,38926508	4,91865774	8,7142E-07
VCA0514	1,4757237	45,7738674	1,47545574	4,4667E-05	1,47545574	4,80565047	1,5425E-06
VC1826	-41,303681	-429,72539	-56,016582	-0,0004193	-56,016582	-4,7415543	2,1209E-06
malE	-4,8547625	-5455,0394	-4,3579838	-0,0053231	-4,3579838	-4,6018507	4,1877E-06
VC2143	-1,6927887	-453,00959	-1,957477	-0,0004421	-1,957477	-4,5375177	5,6922E-06
VCA0508	1,93829384	52,3459875	1,96478916	5,108E-05	1,96478916	4,38059815	1,1836E-05
VC1055	1,79783414	54,180986	1,76097705	5,287E-05	1,76097705	4,36222403	1,2875E-05
VC1920	1,75674528	28,3058133	1,72043221	2,7621E-05	1,72043221	4,0530588	5,0553E-05
VC2283	-2,5331897	-56,001947	-2,5186418	-5,465E-05	-2,5186418	-4,0526493	5,0642E-05
malQ	-5,6337395	-424,7325	-7,0837657	-0,0004145	-7,0837657	-3,9462297	7,9393E-05
VC0034	1,49359784	26,4417742	1,48228684	2,5802E-05	1,48228684	3,92597573	8,638E-05
VCA1115	1,78907795	22,4085518	1,68125291	2,1866E-05	1,68125291	3,90457856	9,4391E-05
VCA0741	-2,0626387	-1258,2909	-2,0014183	-0,0012279	-2,0014183	-3,8945341	9,8389E-05
VC1148	-1,5212746	-69,229599	-1,4749997	-6,755E-05	-1,4749997	-3,8238475	0,00013139
VC1461	-1,6044993	-19,326977	-1,6688235	-1,886E-05	-1,6688235	-3,8119492	0,00013788
VC0410	-1,5077954	-166,42139	-1,6036364	-0,0001624	-1,6036364	-3,7487745	0,0001777
recR	1,62912273	39,9368531	1,58690879	3,8971E-05	1,58690879	3,72285166	0,00019699
rpoB	1,62965718	59,6922162	1,63018373	5,8248E-05	1,63018373	3,67865961	0,00023447
VC0412	-1,5530355	-38,175071	-1,540191	-3,725E-05	-1,540191	-3,6285265	0,00028505
VC0590	2,58207946	23,3935206	2,43163544	2,2828E-05	2,43163544	3,56484738	0,00036407
VCA0043	1,40967129	26,4412204	1,39173256	2,5802E-05	1,39173256	3,55408991	0,00037929
malG	-3,6932035	-540,23669	-4,4272186	-0,0005272	-4,4272186	-3,5307464	0,00041439
VC1825	-4,880914	-532,53668	-5,809387	-0,0005197	-5,809387	-3,4733019	0,0005141
VC0413	-1,5414845	-55,461669	-1,5256144	-5,412E-05	-1,5256144	-3,4560546	0,00054815
VCA1027	-3,2348871	-1003,731	-3,6302852	-0,0009794	-3,6302852	-3,4263311	0,0006118

VC2516	1,32035719	29,5489571	1,30401388	2,8834E-05	1,30401388	3,42026696	0,0006256
VCA0821	2,21836566	46,9006208	2,1804536	4,5766E-05	2,1804536	3,39686891	0,00068162
VC1822	-2,6836448	-61,213993	-2,9691724	-5,973E-05	-2,9691724	-3,3941259	0,00068849
VC2165	1,52381205	19,5963465	1,48657436	1,9122E-05	1,48657436	3,39182225	0,0006943
malF	-3,0997594	-886,67389	-3,8109529	-0,0008652	-3,8109529	-3,3192973	0,00090245
VC2545	1,35086212	83,3290417	1,32454743	8,1313E-05	1,32454743	3,31529084	0,00091548
VC1453	-1,4029312	-28,022544	-1,4184456	-2,734E-05	-1,4184456	-3,2730326	0,00106401
VCA1030	-1,2233121	-36,157522	-1,2166633	-3,528E-05	-1,2166633	-3,2564343	0,00112822
VCA0447	2,43715047	54,3436203	2,38600639	5,3029E-05	2,38600639	3,21946501	0,00128431
VC0589	2,6095319	15,3656161	2,38164008	1,4994E-05	2,38164008	3,21327082	0,00131233
VC1227	2,32873297	8,90285015	2,18750667	8,6875E-06	2,18750667	3,15442728	0,00160814
VCA0845	1,33099403	23,3934036	1,31832948	2,2827E-05	1,31832948	3,10439214	0,00190671
VC0124	1,3854802	24,9307326	1,36879618	2,4328E-05	1,36879618	3,09969919	0,00193718
pepN	1,53475792	17,4131638	1,46606873	1,6992E-05	1,46606873	3,09470715	0,00197008
VCA1021	2,05679324	60,405597	2,06105071	5,8944E-05	2,06105071	3,08236827	0,00205362
tRNA-Arg-5	-1,8815859	-16,858395	-1,965382	-1,645E-05	-1,965382	-3,0505012	0,00228461
malS	-2,7980561	-46,012991	-3,3265528	-4,49E-05	-3,3265528	-3,0421981	0,00234859
VC2658	-1,8964391	-494,57805	-2,039557	-0,0004826	-2,039557	-3,0366588	0,00239218
VC0749	1,58192393	17,4646201	1,52682852	1,7042E-05	1,52682852	3,0361316	0,00239636
VCA0185	2,35702594	8,3602428	2,11911688	8,158E-06	2,11911688	2,99981697	0,00270143
VCA0719	-1,6815755	-10,688001	-1,8274187	-1,043E-05	-1,8274187	-2,9994125	0,00270502
VCA0015	-3,5568826	-119,22846	-3,8992606	-0,0001163	-3,8992606	-2,996435	0,00273158
VC1053	1,3316118	24,692115	1,30429226	2,4095E-05	1,30429226	2,99049141	0,0027853
VC0566	1,51849946	47,7747126	1,52895546	4,6619E-05	1,52895546	2,97249506	0,00295391
VC2659	-1,9583217	-580,40422	-2,0921688	-0,0005664	-2,0921688	-2,9497977	0,00317984
clpP	1,51601393	41,0174361	1,47508168	4,0025E-05	1,47508168	2,94677727	0,00321106
VCA1051	1,68965645	13,9753111	1,61570408	1,3637E-05	1,61570408	2,94158552	0,00326538
VC1358	1,84614103	82,7890871	1,85519627	8,0786E-05	1,85519627	2,91072246	0,00360597
VC1318	1,14151958	3119,35546	1,48261114	0,00304389	1,48261114	2,90811696	0,00363615
VC0856	2,35673015	20,0990983	2,26828199	1,9613E-05	2,26828199	2,9044133	0,00367944
VCA0449	2,41577406	10,4358746	2,35381739	1,0183E-05	2,35381739	2,90156947	0,003713
VC1039	1,51446013	22,096447	1,47677705	2,1562E-05	1,47677705	2,88698511	0,00388954
VC0031	1,97041441	12,6175898	1,84783081	1,2312E-05	1,84783081	2,86840888	0,00412544
rbfA	1,78945058	12,6880829	1,71961153	1,2381E-05	1,71961153	2,86472627	0,00417371
VCA0697	-1,8501559	-27,496735	-2,0148304	-2,683E-05	-2,0148304	-2,8392134	0,00452251
VC2515	1,20365716	38,3174799	1,21024709	3,7391E-05	1,21024709	2,8377594	0,00454316
VC1827	-48,042461	-309,48169	-60,798769	-0,000302	-60,798769	-2,836712	0,0045581
VC1226	1,54145305	13,9667314	1,47413715	1,3629E-05	1,47413715	2,83371474	0,00460105
VC0087	1,22385381	27,8312529	1,24545054	2,7158E-05	1,24545054	2,82139723	0,00478151
bioD	-1,5782473	-210,82852	-1,6351922	-0,0002057	-1,6351922	-2,7794144	0,00544572
VCA0013	-2,7467449	-433,79188	-3,5492039	-0,0004233	-3,5492039	-2,7605233	0,00577091
VC1329	-1,8398097	-10,282259	-2,025514	-1,003E-05	-2,025514	-2,7553274	0,00586336
VCA0899	-1,8887646	-98,189142	-1,8941489	-9,581E-05	-1,8941489	-2,7371457	0,00619751
tRNA-Cys-1	-1,7705194	-19,779537	-1,7831641	-1,93E-05	-1,7831641	-2,695712	0,00702386
VC1820	-19,725955	-281,33844	-23,716402	-0,0002745	-23,716402	-2,6795462	0,00737224

VC1824	-3,5808263	-92,311399	-3,9446926	-9,008E-05	-3,9446926	-2,6764072	0,00744164
VC0467	1,52652336	15,5941857	1,43741487	1,5217E-05	1,43741487	2,67272294	0,00752386
VCA0539	1,71006983	25,1752055	1,69871817	2,4566E-05	1,69871817	2,66544789	0,00768861
VCA0435	1,56517431	11,7873616	1,50117763	1,1502E-05	1,50117763	2,66194442	0,00776909
VCA0180	-1,6635557	-69,381914	-1,6696277	-6,77E-05	-1,6696277	-2,65482	0,00793511
rplN	1,31789716	129,807795	1,13459172	0,00012667	1,13459172	2,6416223	0,00825103
VC2336	-1,7972188	-21,329456	-1,9120579	-2,081E-05	-1,9120579	-2,6145902	0,00893348
glgC (NC_002506 637318..638582)	-2,1074099	-10,469394	-2,265715	-1,022E-05	-2,265715	-2,6139926	0,00894912
VC2657	-1,8521956	-639,97119	-1,8571014	-0,0006245	-1,8571014	-2,6088571	0,00908456
tuf (NC_002505 381739..382964)	1,60930422	2818,7452	2,04616388	0,00275056	2,04616388	2,5897515	0,00960456
VCA0718	-1,5595379	-18,596698	-1,6192086	-1,815E-05	-1,6192086	-2,5726285	0,01009297
VCA0303	-1,5110229	-9,3410064	-1,6108945	-9,115E-06	-1,6108945	-2,5606509	0,01044765
VC1970	-1,4502547	-14,933655	-1,5120525	-1,457E-05	-1,5120525	-2,5472092	0,01085886
VC0018	3,30839329	372,038406	3,18137506	0,00036304	3,18137506	2,5427013	0,01099996
VC2279	1,3042042	17,7209051	1,27532986	1,7292E-05	1,27532986	2,53643534	0,01119877
VC1971	-1,8237828	-37,111673	-1,8518919	-3,621E-05	-1,8518919	-2,508023	0,0121409
VC2662	1,72525926	130,754243	1,71594198	0,00012759	1,71594198	2,50557885	0,01222516
VC1414	1,69435889	33,7642584	1,5828845	3,2947E-05	1,5828845	2,48055332	0,0131179
VC1592	-1,82957	-8,7931449	-1,990787	-8,58E-06	-1,990787	-2,479248	0,013166
VC2490	2,7886045	12,2674362	2,52576421	1,1971E-05	2,52576421	2,4470094	0,01440475
VCA0391	1,44810362	12,7710551	1,36939432	1,2462E-05	1,36939432	2,44404582	0,01452361
groES (NC_002506 765801..766132)	5,18920602	90,0721523	5,06015749	8,7893E-05	5,06015749	2,44004752	0,01468538
VCA0513	1,44695114	27,7143369	1,38531364	2,7044E-05	1,38531364	2,43897096	0,01472919
VCA0538	1,85632519	9,95240366	1,70089245	9,7116E-06	1,70089245	2,43364417	0,01494772
gmhA	1,23255984	19,8154547	1,21989293	1,9336E-05	1,21989293	2,42674236	0,0152351
VCA0130	-2,3912329	-38,833039	-2,5112807	-3,789E-05	-2,5112807	-2,424006	0,01535038
VC0960	1,31076842	22,0857247	1,26528705	2,1551E-05	1,26528705	2,4210555	0,01547554
VCA0213	-1,5070304	-12,931415	-1,5825345	-1,262E-05	-1,5825345	-2,3964747	0,01655367
VCA0237	-2,234224	-23,237529	-2,1951282	-2,268E-05	-2,1951282	-2,3825086	0,01719516
VC1462	-1,376459	-10,63497	-1,4300561	-1,038E-05	-1,4300561	-2,3762368	0,01749027
VC2760	2,22433445	5,55488972	2,02487608	5,4205E-06	2,02487608	2,3761955	0,01749223
VC0468	1,56425682	10,0205308	1,46029645	9,7781E-06	1,46029645	2,37157551	0,01771246
glpK	-5,3429341	-340,81794	-7,0292696	-0,0003326	-7,0292696	-2,3653572	0,01801274
VC0179	1,76839953	143,033084	1,8591634	0,00013957	1,8591634	2,36002396	0,01827379
glyA	1,86478674	48,5913794	1,8427964	4,7416E-05	1,8427964	2,33736226	0,01942041
hscA	1,46162671	13,3164406	1,39704437	1,2994E-05	1,39704437	2,33681354	0,01944892
VC2290	1,173456	38,2151396	1,16855783	3,7291E-05	1,16855783	2,33180233	0,01971113
groEL (NC_002506 766120..767753)	3,89334862	50,4438616	3,86299493	4,9224E-05	3,86299493	2,32908793	0,01985446
VC0078	-1,2147975	-44,407613	-1,2169286	-4,333E-05	-1,2169286	-2,3238202	0,02013517
VCA0450	2,22051234	7,29823974	2,12048939	7,1217E-06	2,12048939	2,31905077	0,02039233
VC1125	-1,2776362	-21,640794	-1,2913363	-2,112E-05	-1,2913363	-2,3145981	0,02063497
truB	1,74600735	9,41012216	1,66224329	9,1825E-06	1,66224329	2,31383847	0,02067662
VC2681	1,65421565	16,1899375	1,57616116	1,5798E-05	1,57616116	2,30399529	0,02122295
tRNA-Arg-8	-1,9230673	-67,9442	-1,8930064	-6,63E-05	-1,8930064	-2,3024775	0,02130831

VCA0175	1,5433579	11,1200335	1,4412616	1,0851E-05	1,4412616	2,29236403	0,02188468
VC0335	-1,3282667	-13,632138	-1,3811202	-1,33E-05	-1,3811202	-2,2914812	0,02193563
VC1174	-2,0357739	-4,2194731	-2,4048198	-4,117E-06	-2,4048198	-2,285358	0,02229188
VC0488	-1,637462	-6,3754337	-1,7450903	-6,221E-06	-1,7450903	-2,2784752	0,02269832
VC0285	-2,0685945	-27,527032	-2,1991485	-2,686E-05	-2,1991485	-2,2747119	0,02292326
aceE	2,67755842	67,5233686	2,71353731	6,589E-05	2,71353731	2,25730585	0,02398902
VC1300	-1,1991746	-22,776556	-1,181776	-2,223E-05	-1,181776	-2,2553285	0,02411275
VC2292	1,21308629	38,1669118	1,25030007	3,7244E-05	1,25030007	2,25523278	0,02411877
VC0428	-1,7373401	-210,39748	-1,7387476	-0,0002053	-1,7387476	-2,2524938	0,02429113
VCA1114	1,63438387	8,26506584	1,51925952	8,0651E-06	1,51925952	2,25145605	0,0243567
VC0329	1,6748806	112,194429	1,70216287	0,00010948	1,70216287	2,25084757	0,02439524
VCA0728	1,3046898	24,4232623	1,33285006	2,3832E-05	1,33285006	2,2442333	0,02481743
VC0478	-1,3122824	-416,69647	-1,4552789	-0,0004066	-1,4552789	-2,2422671	0,02494416
yajC	1,37005871	64,1814323	1,36924435	6,2629E-05	1,36924435	2,2422412	0,02494585
infB	1,77203663	74,5916721	1,77391883	7,2787E-05	1,77391883	2,22900536	0,02581362
VC2418	1,45846738	10,8127491	1,35956835	1,0551E-05	1,35956835	2,22336533	0,02619121
VC2614	-1,536415	-553,06109	-1,5379807	-0,0005397	-1,5379807	-2,2223207	0,0262617
VCA0748	-7,6847321	-156,5616	-9,453909	-0,0001528	-9,453909	-2,2178985	0,02656182
hslU	2,20242698	43,264259	2,12008374	4,2218E-05	2,12008374	2,21151317	0,02700038
VC0302	-1,6417205	-9,6581605	-1,681951	-9,425E-06	-1,681951	-2,2108322	0,0270475
23Sb	1,10210201	3640,34897	1,37918471	0,00355228	1,37918471	2,20616525	0,02737248
VC0651	-1,4051662	-35,766579	-1,4131447	-3,49E-05	-1,4131447	-2,1972963	0,02799934
VC1418	1,514894	17,0765907	1,46277278	1,6663E-05	1,46277278	2,19249175	0,02834406
VCA1023	1,4333769	9,99982363	1,38108263	9,7579E-06	1,38108263	2,19121973	0,02843593
tRNA-Arg-3	-1,6469166	-23,97476	-1,6682042	-2,339E-05	-1,6682042	-2,1885793	0,02862748
VC2037	-1,7304751	-169,20692	-1,9473592	-0,0001651	-1,9473592	-2,1769444	0,02948477
ilvH	1,68391745	18,719692	1,6656308	1,8267E-05	1,6656308	2,17351333	0,02974175
VC1486	1,3526151	11,7034916	1,30798241	1,142E-05	1,30798241	2,16458438	0,03041957
VC1425	1,58429526	83,2516423	1,60337514	8,1238E-05	1,60337514	2,16347809	0,0305045
VC1178	1,45699663	12,720047	1,36081442	1,2412E-05	1,36081442	2,15496485	0,03116463
groEL (NC_002505 2832599..2834274)	2,65353996	503,538714	2,50520429	0,00049136	2,50520429	2,14921518	0,03161739
clpX	1,21678111	48,9529537	1,22857976	4,7769E-05	1,22857976	2,14449788	0,03199306
VC1624	2,91647046	21,2111641	2,83002402	2,0698E-05	2,83002402	2,13702586	0,03259596
xerD	1,49137104	9,31208791	1,38698081	9,0868E-06	1,38698081	2,12819127	0,03332127
hisG	1,94686331	9,00225761	1,78669677	8,7845E-06	1,78669677	2,12634672	0,03347445
VC1849	1,59180432	128,945133	1,50421764	0,00012583	1,50421764	2,11442232	0,03447926
VC1623	2,66655342	25,5133797	2,58088621	2,4896E-05	2,58088621	2,1012731	0,03561707
VCA0784	-1,4440538	-72,888025	-1,4750385	-7,112E-05	-1,4750385	-2,1011207	0,03563043
VCA0451	2,33592658	3,81660758	2,30056711	3,7243E-06	2,30056711	2,09848936	0,03586199
VC2741	1,27818238	12,4339801	1,26461408	1,2133E-05	1,26461408	2,08778856	0,03681695
VC0753	1,34981663	11,2421265	1,29662482	1,097E-05	1,29662482	2,08714661	0,03687493
VCA0174	1,4065942	10,3612658	1,32396095	1,0111E-05	1,32396095	2,08164649	0,03737481
VC2458	1,52232286	28,9296896	1,51151992	2,823E-05	1,51151992	2,08001022	0,03752466
VC0558	-1,2246664	-18,989572	-1,2122755	-1,853E-05	-1,2122755	-2,0789556	0,03762147
tRNA-Ser-2	1,19226375	20,0664576	1,19973156	1,9581E-05	1,19973156	2,07780509	0,03772736

VC2656	-1,6700879	-652,04472	-1,6353826	-0,0006363	-1,6353826	-2,0775774	0,03774837
VC1476	1,31784786	11,7565174	1,27758468	1,1472E-05	1,27758468	2,07334142	0,03814056
VCA0954	-1,3582477	-90,081558	-1,3876683	-8,79E-05	-1,3876683	-2,0732837	0,03814593
VC0244	1,5402497	391,618748	1,43211626	0,00038214	1,43211626	2,07090198	0,03836801
VC2699	-1,8742954	-196,71138	-1,9340438	-0,000192	-1,9340438	-2,0631323	0,03910013
VC0408	-1,3784465	-32,77847	-1,3512646	-3,199E-05	-1,3512646	-2,0615387	0,03925173
VCA0337	1,36838683	10,7328996	1,3024072	1,0473E-05	1,3024072	2,05652014	0,03973245
VC1831	-1,4106654	-12,342888	-1,5045512	-1,204E-05	-1,5045512	-2,0533482	0,04003886
metG	1,27550101	17,2348174	1,25998211	1,6818E-05	1,25998211	2,05310256	0,04006268
VCA0463	1,27160843	13,1385856	1,23903449	1,2821E-05	1,23903449	2,05138897	0,04022913
VC0556	1,57295698	15,5692817	1,50500582	1,5193E-05	1,50500582	2,0494285	0,04042029
groES (NC_002505 2834287..2834618)	3,32607223	701,923371	3,26404636	0,00068494	3,26404636	2,04807087	0,04055312
VC0787	3,1881259	2,90100195	2,81452116	2,8308E-06	2,81452116	2,03468149	0,04188298
VC2001	-1,8802938	-72,741735	-1,8453324	-7,098E-05	-1,8453324	-2,0346702	0,04188411
VCA0785	-1,4403737	-6,1879477	-1,5717254	-6,038E-06	-1,5717254	-2,0315078	0,04220356
VC2566	-1,1944352	-11,09216	-1,2817417	-1,082E-05	-1,2817417	-2,027079	0,04265438
VCA0964	-1,327913	-13,377538	-1,3700049	-1,305E-05	-1,3700049	-2,0205151	0,04333003
VCA0623	1,86191244	74,8001935	1,84571623	7,2991E-05	1,84571623	2,01875841	0,04351239
VC1117	1,92507862	21,6619808	1,82364182	2,1138E-05	1,82364182	2,01513528	0,04389055
VC2047	-1,3160996	-11,540565	-1,3698397	-1,126E-05	-1,3698397	-2,0148417	0,04392129
glpC	-11,790012	-243,36015	-15,351582	-0,0002375	-15,351582	-2,0128209	0,04413356
VC0654	-1,5934075	-213,59842	-1,6358319	-0,0002084	-1,6358319	-2,0117805	0,04424313
VC0178	1,64638782	95,6621012	1,72545602	9,3348E-05	1,72545602	2,00906839	0,04452995
VC2387	1,69349339	74,8648562	1,72342822	7,3054E-05	1,72342822	2,00791204	0,04465268
VC1315	-1,6749812	-65,299271	-1,7992075	-6,372E-05	-1,7992075	-2,0064883	0,04480424
rpsL	1,63297433	194,922008	1,46309713	0,00019021	1,46309713	2,00149757	0,04533887
lpxC	-1,1922332	-124,75223	-1,3404949	-0,0001217	-1,3404949	-2,0002437	0,04547401
VC2675	2,14838862	24,4162168	2,0417823	2,3826E-05	2,0417823	1,99926206	0,04558008
VCA0108	1,69551346	6,20528421	1,54463367	6,0552E-06	1,54463367	1,99610915	0,04592209
VC1546	2,14872554	5,13477867	2,00142675	5,0106E-06	2,00142675	1,99560105	0,04597742
VCA0486	1,61757293	68,3450974	1,64789915	6,6692E-05	1,64789915	1,99521774	0,04601922
VCA1075	-1,3761006	-237,847	-1,5833506	-0,0002321	-1,5833506	-1,987913	0,04682139
VC1689	-1,8140398	-23,883859	-2,0076066	-2,331E-05	-2,0076066	-1,9855609	0,04708216
VCA0173	1,25212292	14,8061237	1,20130828	1,4448E-05	1,20130828	1,98481566	0,04716503
VCA0084	1,37887185	9,09551024	1,33653563	8,8755E-06	1,33653563	1,98248684	0,04742482
VC1803	1,45067579	66,4591451	1,46387539	6,4851E-05	1,46387539	1,97544547	0,04821768
VC1972	-1,6591748	-42,944017	-1,6633922	-4,191E-05	-1,6633922	-1,9730509	0,04848981
VC2291	1,2141614	44,1457344	1,20733757	4,3078E-05	1,20733757	1,97218285	0,04858878
VC0165	1,48709706	35,7383178	1,46519175	3,4874E-05	1,46519175	1,96269014	0,04968227
VCA0301	-1,2228854	-9,8895566	-1,3034207	-9,65E-06	-1,3034207	-1,9626866	0,04968265
glpA	-3,9940265	-139,00832	-4,8527036	-0,0001356	-4,8527036	-1,9541925	0,05067853
VC0482	1,8772836	4,75317086	1,72889973	4,6382E-06	1,72889973	1,95161643	0,05098381
VC0188	2,35995384	24,8483088	2,24173938	2,4247E-05	2,24173938	1,95035264	0,05113417
VC2367	1,26750736	14,715559	1,24434518	1,436E-05	1,24434518	1,94982848	0,05119661
VC1173	-2,0691113	-3,1453837	-2,3451322	-3,069E-06	-2,3451322	-1,947934	0,05142291

VC2145	-1,5106643	-124,06416	-1,5255962	-0,0001211	-1,5255962	-1,9453925	0,05172781
VCA0107	1,79565061	5,14387957	1,64529647	5,0194E-06	1,64529647	1,94021947	0,05235307
VC0698	1,28034835	17,9130521	1,24424426	1,748E-05	1,24424426	1,93767908	0,05266244
VC0553	1,34112065	10,1348375	1,28045793	9,8897E-06	1,28045793	1,93375172	0,05314371
VC0360	1,72540562	257,186263	1,51351539	0,00025096	1,51351539	1,93199474	0,05336023
VC1301	-1,5048981	-141,04255	-1,5141467	-0,0001376	-1,5141467	-1,9312414	0,05345325
VC0416	-1,1857872	-15,135706	-1,2292481	-1,477E-05	-1,2292481	-1,9308278	0,0535044
VC0842	2,23450028	11,8693125	2,18588099	1,1582E-05	2,18588099	1,91558497	0,05541802
VCA0763	1,54068733	6,41972231	1,47021273	6,2644E-06	1,47021273	1,91470762	0,05552985
VC0487	-3,9929544	-67,715885	-3,7135147	-6,608E-05	-3,7135147	-1,9123223	0,05583494
VCA0356	1,32057139	13,7491355	1,27688963	1,3417E-05	1,27688963	1,91002569	0,05612995
VCA0488	1,31721401	39,3423763	1,34719437	3,8391E-05	1,34719437	1,90979661	0,05615948
VC1435	-1,4870839	-15,726886	-1,5646031	-1,535E-05	-1,5646031	-1,9043228	0,05686821
infC	1,3890364	2517,86515	1,85122065	0,00245695	1,85122065	1,90418185	0,05688659
VC0286	-2,0813651	-8,9612313	-2,4539063	-8,744E-06	-2,4539063	-1,9041316	0,0568931
tsf	1,92363942	231,107843	1,70058471	0,00022552	1,70058471	1,90234863	0,05712567
VC2483	1,79125303	12,877297	1,73552875	1,2566E-05	1,73552875	1,90058046	0,05735706
rpmC	2,84330405	1529,77677	3,0549319	0,00149277	3,0549319	1,89722282	0,05779861
VC1266	2,79617742	7,30838749	2,57793314	7,1316E-06	2,57793314	1,8965473	0,05788779
VC1477	1,45182907	145,292219	1,49806218	0,00014178	1,49806218	1,88663102	0,05921002
VCA0946	-2,1462452	-251,23113	-2,5583312	-0,0002452	-2,5583312	-1,8830981	0,05968716
VCA0098	2,1271751	5,88956275	1,93838401	5,7471E-06	1,93838401	1,88304618	0,05969417
VCA0367	1,33270847	25,0243799	1,36084913	2,4419E-05	1,36084913	1,88172175	0,05987385
VC1810	-1,204506	-15,873555	-1,2480877	-1,549E-05	-1,2480877	-1,8769529	0,0605246
VC0985	2,53731319	247,824984	2,50977684	0,00024183	2,50977684	1,8766581	0,06056502
VCA0493	1,38869222	22,2046767	1,37886201	2,1668E-05	1,37886201	1,87615197	0,06063447
VC1093	1,45285352	12,3300044	1,36128126	1,2032E-05	1,36128126	1,8753986	0,06073796
tgt	1,70872219	21,012172	1,64695611	2,0504E-05	1,64695611	1,86754882	0,06182507
pdhR	1,48426221	15,99464	1,47292932	1,5608E-05	1,47292932	1,86342316	0,0624028
rrmJ	1,42346718	25,856638	1,39604174	2,5231E-05	1,39604174	1,86152873	0,06266962
aceF	2,49836967	71,3985547	2,5620976	6,9671E-05	2,5620976	1,85470887	0,06363788
VCA0131	-1,6046885	-7,718443	-1,778265	-7,532E-06	-1,778265	-1,8536793	0,0637851
VC2251	1,45138215	110,375941	1,41195771	0,00010771	1,41195771	1,84983482	0,06433741
VC1593	-1,8363188	-36,17006	-2,0031691	-3,53E-05	-2,0031691	-1,8417676	0,06550921
VCA0754a	1,91017691	4,02510548	1,83156484	3,9277E-06	1,83156484	1,84091885	0,06563351
VC0134	-1,7179173	-83,740031	-1,6502518	-8,171E-05	-1,6502518	-1,8405266	0,06569106
VCA0998	1,70031784	5,63297422	1,60628132	5,4967E-06	1,60628132	1,84009218	0,06575476
VC2469	1,13871758	17,3468627	1,13873191	1,6927E-05	1,13873191	1,83742472	0,06614727
flgE	-1,3034275	-102,14868	-1,4066412	-9,968E-05	-1,4066412	-1,8362942	0,06631422
VC0274	-1,3644321	-59,006939	-1,3883901	-5,758E-05	-1,3883901	-1,8339282	0,0666647
rpsS	2,47062551	1514,81475	2,79890532	0,00147817	2,79890532	1,83271189	0,0668455
VC1159	1,470562	25,3722367	1,40840159	2,4758E-05	1,40840159	1,83170952	0,06699476
VC0407	-1,2248325	-21,315785	-1,1967484	-2,08E-05	-1,1967484	-1,8307678	0,06713525
frr	1,27526418	37,8278091	1,30791515	3,6913E-05	1,30791515	1,82957485	0,06731362
VCA0948	-1,4329974	-52,698872	-1,4548851	-5,142E-05	-1,4548851	-1,8283238	0,06750106

VC0711	2,19944883	46,7202829	2,14614466	4,559E-05	2,14614466	1,82799885	0,06754978
VC0748	1,36831275	14,6559961	1,3360398	1,4301E-05	1,3360398	1,82523239	0,06796603
glpD	-2,2800455	-37,962391	-2,8435934	-3,704E-05	-2,8435934	-1,8241379	0,06813127
rplK	1,73561267	1008,68279	1,81110305	0,00098428	1,81110305	1,8224396	0,06838838
VC0565	-1,2314942	-19,187356	-1,236057	-1,872E-05	-1,236057	-1,8205183	0,06868016
VCA1026	-2,3849787	-159,34269	-2,7186592	-0,0001555	-2,7186592	-1,8187253	0,06895343
VC1741	3,77470963	2,23601345	3,1107336	2,1819E-06	3,1107336	1,81836231	0,06900884
VC0414	-1,4181059	-30,568516	-1,4291089	-2,983E-05	-1,4291089	-1,8169912	0,06921852
recO	1,46333881	6,89434998	1,37961172	6,7276E-06	1,37961172	1,81647215	0,06929803
VC1637	1,69675493	13,2710278	1,60482387	1,295E-05	1,60482387	1,81073394	0,07018212
VCA0941	1,34293461	18,028265	1,31844035	1,7592E-05	1,31844035	1,809232	0,07041504
VCA0399	1,29346802	11,7302275	1,25450773	1,1446E-05	1,25450773	1,80726	0,0707218
rplL	2,00381906	278,332206	1,8190106	0,0002716	1,8190106	1,7999748	0,07186471
tRNA-Arg-7	-1,8430161	-18,545413	-1,7977562	-1,81E-05	-1,7977562	-1,7979673	0,07218227
VCA0047	-1,2683286	-19,286046	-1,3385098	-1,882E-05	-1,3385098	-1,7960503	0,07248657
VC0606	-1,4632146	-5,6690967	-1,5684286	-5,532E-06	-1,5684286	-1,7903189	0,07340272
VC1545	2,14962315	3,23373926	1,98681173	3,1555E-06	1,98681173	1,79030698	0,07340463
secG	-1,3891918	-515,24984	-1,4986041	-0,0005028	-1,4986041	-1,7893243	0,07356268
VC2571	1,34434139	157,459246	1,17268695	0,00015365	1,17268695	1,78894726	0,07362337
VCA0742	-1,8281099	-125,88801	-1,8002231	-0,0001228	-1,8002231	-1,7865052	0,07401755
VC0472	1,52650037	19,1050487	1,48599698	1,8643E-05	1,48599698	1,78273995	0,07462872
VC0554	1,33769223	8,90063994	1,27006888	8,6853E-06	1,27006888	1,78236661	0,07468952
VC0142a	2,0651703	3,13931083	2,01783374	3,0634E-06	2,01783374	1,78225422	0,07470784
VC0841	1,61688969	24,6503633	1,59755388	2,4054E-05	1,59755388	1,78195379	0,07475684
VCA0097	1,49157679	7,60576474	1,42590299	7,4218E-06	1,42590299	1,77950211	0,07515753
rplX	1,35698132	251,635902	1,22946051	0,00024555	1,22946051	1,77714995	0,07554365
glpQ	-3,6906652	-94,051052	-4,4505492	-9,178E-05	-4,4505492	-1,770606	0,07662633
VC2166	1,36574669	9,30525088	1,29484624	9,0801E-06	1,29484624	1,76408301	0,07771806
VC1801	2,78021779	2,40874556	2,50921713	2,3505E-06	2,50921713	1,76289943	0,07791751
VCA0795	1,78614729	4,19640138	1,64987138	4,0949E-06	1,64987138	1,75712382	0,07889676
VC0067	1,55477438	5,87714178	1,43233187	5,735E-06	1,43233187	1,75452688	0,07934032
tolC	1,20729411	33,949059	1,22083379	3,3128E-05	1,22083379	1,75424831	0,07938804
rplJ	2,03278155	607,404459	1,78463948	0,00059271	1,78463948	1,75308806	0,07958699
VCA0109	1,84025675	4,10050967	1,66275975	4,0013E-06	1,66275975	1,74982039	0,08014936
VC1843	1,75840356	112,623711	1,82128978	0,0001099	1,82128978	1,74965461	0,08017803
VC0656	-1,6527101	-25,964844	-1,6539754	-2,534E-05	-1,6539754	-1,7492402	0,08024957
VC1715	1,33060988	8,23127274	1,28122269	8,0321E-06	1,28122269	1,74478792	0,08102184
VC1010	1,29454188	29,2964748	1,29754799	2,8588E-05	1,29754799	1,74188091	0,08152936
VC0243	1,4898145	365,797196	1,42046165	0,00035695	1,42046165	1,74134612	0,08162299
fabG	1,21200278	34,3447341	1,2489172	3,3514E-05	1,2489172	1,74110025	0,08166608
VC0862	-1,4264505	-22,644922	-1,5776183	-2,21E-05	-1,5776183	-1,7405666	0,08175964
VC1842	1,67397193	26,8080706	1,64640847	2,616E-05	1,64640847	1,73969101	0,08191338
VC0886	2,26830362	9,84210419	2,04210485	9,604E-06	2,04210485	1,7392305	0,0819943
VC2035	-1,5898133	-13,499554	-1,5841329	-1,317E-05	-1,5841329	-1,7387079	0,08208623
dnaK	2,30232491	273,477229	2,24928856	0,00026686	2,24928856	1,73287875	0,08311728

VC1498	2,21549559	26,0513775	2,14553809	2,5421E-05	2,14553809	1,73278166	0,08313458
VCA0445	1,61993402	12,0424681	1,53665339	1,1751E-05	1,53665339	1,72841745	0,08391348
VC0164	1,61262856	19,1354602	1,5715202	1,8673E-05	1,5715202	1,72772143	0,08403826
VCA0324	1,25598605	17,6671163	1,26150415	1,724E-05	1,26150415	1,7276162	0,08405712
VCA0858	-1,4636012	-21,097994	-1,6201041	-2,059E-05	-1,6201041	-1,7274712	0,08408313
VC0731	1,47227215	189,474541	1,45235335	0,00018489	1,45235335	1,72395354	0,0847163
VC0671	1,15628474	24,0550802	1,15659528	2,3473E-05	1,15659528	1,72354428	0,08479021
VC0457	1,29936462	9,12107836	1,24303729	8,9004E-06	1,24303729	1,721912	0,08508551
VC1264	3,12113444	19,813138	2,95383482	1,9334E-05	2,95383482	1,71773597	0,08584487
tRNA-Leu-1	-1,9047988	-74,599612	-1,8218072	-7,279E-05	-1,8218072	-1,7174404	0,08589883
VCA0017	-1,8581022	-13,865655	-1,9351459	-1,353E-05	-1,9351459	-1,7122873	0,08684377
VCA0924	1,60403049	5,15720053	1,46686808	5,0324E-06	1,46686808	1,71117629	0,08704862
VCA0052	-1,8376276	-79,717129	-1,892172	-7,779E-05	-1,892172	-1,7104608	0,08718076
pfkA	-2,3064603	-513,32466	-2,4664304	-0,0005009	-2,4664304	-1,7089782	0,08745506
VCA0079	-1,1914476	-25,568288	-1,1988082	-2,495E-05	-1,1988082	-1,708438	0,08755515
VC0982	1,13662655	33,596112	1,18422111	3,2783E-05	1,18422111	1,70252303	0,08865741
VC1415	-1,8829491	-8,8747339	-1,9101826	-8,66E-06	-1,9101826	-1,7024053	0,08867948
VC0577	-1,1847054	-35,906887	-1,1654095	-3,504E-05	-1,1654095	-1,7016971	0,08881223
VC1419	1,47095018	8,38441559	1,39729811	8,1816E-06	1,39729811	1,70162321	0,08882608
tig	1,34595906	77,7350185	1,33735813	7,5855E-05	1,33735813	1,70150479	0,08884833
VC1316	-1,2844216	-30,474451	-1,3243579	-2,974E-05	-1,3243579	-1,6999267	0,08914476
VC1265	2,70606411	7,30430125	2,47939559	7,1276E-06	2,47939559	1,6964945	0,08979235
VC0863	-1,9380995	-67,291651	-2,1708093	-6,566E-05	-2,1708093	-1,6948519	0,0901036
VCA0676	5,76295842	40,0150855	5,72135566	3,9047E-05	5,72135566	1,69250969	0,09054893
rplV	2,69122276	718,021596	2,51550245	0,00070065	2,51550245	1,69145077	0,09075081
VC0180	1,39034243	58,1338968	1,50064314	5,6728E-05	1,50064314	1,69034368	0,09096228
VC0657	-1,4004316	-37,41631	-1,3896682	-3,651E-05	-1,3896682	-1,6867612	0,09164934
VC1483	1,41587544	73,2805178	1,40411062	7,1508E-05	1,40411062	1,68663515	0,09167359
VC1444	1,18876732	14,9089694	1,19087545	1,4548E-05	1,19087545	1,68267179	0,09243869
VC1793	1,91886923	3,44304636	1,75436639	3,3598E-06	1,75436639	1,6819528	0,09257804
VC0480	1,25792608	42,0832065	1,29531848	4,1065E-05	1,29531848	1,68145279	0,09267508
VCA0535	-1,53435	-22,329197	-1,5954252	-2,179E-05	-1,5954252	-1,679521	0,0930506
VC0815	1,62322698	7,94825137	1,52321823	7,756E-06	1,52321823	1,6782286	0,09330254
VC0854	1,96160202	130,684633	2,01406733	0,00012752	2,01406733	1,67687757	0,09356651
hisD	1,78915409	4,82141879	1,64492253	4,7048E-06	1,64492253	1,67647578	0,09364509
VC2038	-1,4810048	-3,9776828	-1,6130073	-3,881E-06	-1,6130073	-1,6731701	0,09429385
VC2205	-1,3233977	-31,14768	-1,3061824	-3,039E-05	-1,3061824	-1,6731307	0,09430166
VCA0677	6,57408387	44,6844261	6,58208421	4,3603E-05	6,58208421	1,66659756	0,09559455
VC0479	1,31924433	80,570472	1,32255022	7,8621E-05	1,32255022	1,66356122	0,09620022
VC0926	1,51857485	5,04848723	1,44706061	4,9264E-06	1,44706061	1,66343227	0,09622599
rpsC	2,76693795	502,461166	2,7143213	0,00049031	2,7143213	1,66338639	0,09623519
VC0750	1,48907294	25,8863097	1,48904024	2,526E-05	1,48904024	1,66325886	0,09626072
VC1641	2,27692088	66,985965	2,28662875	6,5365E-05	2,28662875	1,66319354	0,09627378
VC0655	-1,5676125	-81,210986	-1,504717	-7,925E-05	-1,504717	-1,6615624	0,09660061
VCA0516	-3,2236812	-395,90884	-3,7470241	-0,0003863	-3,7470241	-1,6614751	0,09661812

VCA0622	2,54312084	4,30341875	2,25308973	4,1993E-06	2,25308973	1,66039977	0,09683411
VC2137	-1,1393056	-14,291496	-1,1368075	-1,395E-05	-1,1368075	-1,6568971	0,09754033
VC1185	-1,467137	-6,4304675	-1,5624739	-6,275E-06	-1,5624739	-1,6558866	0,09774483
rpmD	1,90896985	508,849426	1,77692656	0,00049654	1,77692656	1,65468537	0,09798839
rplB	2,31678104	326,869204	2,21476587	0,00031896	2,21476587	1,65395291	0,09813714
fliP	-1,248955	-17,340094	-1,2658666	-1,692E-05	-1,2658666	-1,6537145	0,0981856
VC0834	2,071476	8,85876909	1,96614965	8,6445E-06	1,96614965	1,65358468	0,098212
VC0348	-1,0628172	-82,406619	-1,2036053	-8,041E-05	-1,2036053	-1,648157	0,09932054
glpT	-3,5862811	-118,58143	-4,3654798	-0,0001157	-4,3654798	-1,6457113	0,09982329
oppD	1,39942051	10,26266	1,32401751	1,0014E-05	1,32401751	1,64308034	0,10036636
VC1135	1,78460479	8,80269152	1,72721013	8,5897E-06	1,72721013	1,64167934	0,10065655
rpmI	1,40381269	703,421611	1,53685536	0,0006864	1,53685536	1,63679615	0,10167317
VC1231	1,63602327	4,57382713	1,47928825	4,4632E-06	1,47928825	1,62868831	0,10337907
VC1532	-1,9197553	-143,08542	-2,0854931	-0,0001396	-2,0854931	-1,6263242	0,10388077
VC1114	-1,3442198	-23,527809	-1,3170181	-2,296E-05	-1,3170181	-1,6253152	0,10409548
rplO	1,85786794	442,876356	1,68800364	0,00043216	1,68800364	1,62356214	0,10446935
VC0319	-1,1414379	-12,579094	-1,149724	-1,227E-05	-1,149724	-1,6212832	0,10495697
VCA0333	1,37937717	83,5627583	1,32474176	8,1541E-05	1,32474176	1,61900068	0,10544719
leuS	1,23514152	10,8094635	1,20858293	1,0548E-05	1,20858293	1,61865197	0,10552221
VC1768	1,46295629	38,4170057	1,52715057	3,7488E-05	1,52715057	1,61692625	0,10589425
VC0172	1,5082717	8,46884138	1,44876412	8,264E-06	1,44876412	1,61525269	0,10625604
VC1313	-1,2229994	-6,591756	-1,3027553	-6,432E-06	-1,3027553	-1,6124587	0,10686222
VC1548	2,11378223	3,95419861	1,97710037	3,8585E-06	1,97710037	1,61155214	0,1070595
VCA0887	1,47427105	5,49909933	1,37353205	5,3661E-06	1,37353205	1,61130067	0,10711425
rplR	1,73141909	313,212939	1,53389489	0,00030564	1,53389489	1,61112334	0,10715291
VCA0135	-1,5788874	-11,678105	-1,7730338	-1,14E-05	-1,7730338	-1,6074432	0,10795727
VC1110	1,11638514	25,7134345	1,15611511	2,5091E-05	1,15611511	1,6061262	0,10824625
secY	1,85756992	439,822486	1,67965418	0,00042918	1,67965418	1,60543759	0,10839761
mtlR	-1,2107506	-7,6265564	-1,2747317	-7,442E-06	-1,2747317	-1,6035167	0,10882067
VC2517	1,29646616	18,7666403	1,31141775	1,8313E-05	1,31141775	1,60168529	0,1092253
VC2167	1,31979361	9,00697474	1,24879377	8,7891E-06	1,24879377	1,60139857	0,10928872
flgL	-1,2800045	-25,754351	-1,3056322	-2,513E-05	-1,3056322	-1,5997261	0,10965943
VC0835	2,09945062	7,42805295	2,00712487	7,2484E-06	2,00712487	1,59750006	0,11015437
VC0092	1,73190257	17,1930995	1,61410858	1,6777E-05	1,61410858	1,5939126	0,1109557
VC1217	2,16685085	10,8909739	1,98510336	1,0628E-05	1,98510336	1,59047943	0,11172686
VC0873	1,43257929	9,94992955	1,38355295	9,7092E-06	1,38355295	1,58987949	0,11186203
VCA0319	1,56903862	6,04708786	1,5044852	5,9008E-06	1,5044852	1,58514906	0,11293253
VC2033	-2,3528233	-1067,3585	-2,6785239	-0,0010415	-2,6785239	-1,5834707	0,11331429
VC0695	1,81544702	4,1648422	1,64307072	4,0641E-06	1,64307072	1,58329351	0,11335464
VCA0490	1,3354128	9,31683379	1,27646015	9,0914E-06	1,27646015	1,58265755	0,1134996
VCA0487	1,40841733	23,8672073	1,41135043	2,329E-05	1,41135043	1,5817958	0,11369628
VCA0397	1,20602282	14,1640251	1,1776966	1,3821E-05	1,1776966	1,57801967	0,11456114
VC0191	-1,3994991	-4,2932456	-1,4784945	-4,189E-06	-1,4784945	-1,5768865	0,11482171
VC1343	-1,4205022	-48,99052	-1,458701	-4,781E-05	-1,458701	-1,5760985	0,1150032
rplP	2,79635043	639,186331	2,71124616	0,00062372	2,71124616	1,56949902	0,11653178

dcuC	-1,4951866	-130,2113	-1,6224268	-0,0001271	-1,6224268	-1,5672442	0,11705768
hscB	1,39491035	5,90967949	1,32104166	5,7667E-06	1,32104166	1,56598079	0,11735317
VCA0471	1,45202658	29,1787062	1,44474271	2,8473E-05	1,44474271	1,56459556	0,11767786
VCA0607	1,32554043	20,2126909	1,30831297	1,9724E-05	1,30831297	1,56205014	0,11827625
VC1544	1,92313091	9,59363133	1,82775252	9,3615E-06	1,82775252	1,56192877	0,11830484
VCA0457	1,52859712	4,49751346	1,44129933	4,3887E-06	1,44129933	1,5617291	0,11835187
VC0486	-4,5476602	-96,634176	-4,2195305	-9,43E-05	-4,2195305	-1,5616243	0,11837662
rpsE	1,76664173	178,316367	1,69294985	0,000174	1,69294985	1,55858731	0,11909416
VC1547	2,16397363	3,46844636	2,07431053	3,3845E-06	2,07431053	1,55248231	0,12054693
VCA0752	1,96260125	36,2780874	1,98110713	3,54E-05	1,98110713	1,55098698	0,12090485
VC1640	1,85554363	239,295327	1,61584998	0,00023351	1,61584998	1,54796947	0,12162974
VCA0049	-1,2867386	-11,788668	-1,391187	-1,15E-05	-1,391187	-1,5468915	0,12188948
VC0890	1,36528893	9,918612	1,29920435	9,6787E-06	1,29920435	1,54300368	0,12282995
VCA0099	1,38420625	6,8354541	1,29268378	6,6701E-06	1,29268378	1,54132612	0,12323749
VC0082	1,48161694	4,70852257	1,40430597	4,5946E-06	1,40430597	1,54122561	0,12326194
VCA0189	1,28296245	8,00446976	1,21855585	7,8108E-06	1,21855585	1,5380896	0,12402676
VC1753	-1,1903668	-6,4735238	-1,2767218	-6,317E-06	-1,2767218	-1,5363968	0,12444114
VCA1042	1,2263167	16,9106484	1,19356145	1,6502E-05	1,19356145	1,53352516	0,12514657
VCA0021	1,38076562	6,11907836	1,2922971	5,971E-06	1,2922971	1,52997948	0,12602186
VC0409	-1,301852	-370,69584	-1,3421429	-0,0003617	-1,3421429	-1,5288744	0,12629568
VC2084	-2,5994322	-165,78393	-2,5204342	-0,0001618	-2,5204342	-1,5283834	0,12641746
VCA0236	-1,9464979	-126,74371	-1,7842886	-0,0001237	-1,7842886	-1,5282507	0,12645037
VCA0898	2,15484316	32,6220511	2,11143113	3,1833E-05	2,11143113	1,52730986	0,12668406
VC2601	-1,3115392	-17,48418	-1,3394325	-1,706E-05	-1,3394325	-1,5270218	0,12675567
tRNA-Gln-2	-1,9691201	-2,4670803	-2,1129513	-2,407E-06	-2,1129513	-1,526934	0,12677748
VC1460	-1,3627509	-4,295594	-1,4414421	-4,192E-06	-1,4414421	-1,5264708	0,1268927
tRNA-Leu-11	-1,3995691	-21,29233	-1,3954886	-2,078E-05	-1,3954886	-1,519618	0,12860708
VC1642	2,5740921	86,8512984	2,52402195	8,475E-05	2,52402195	1,51886702	0,12879606
VC1368	1,22221049	12,3643047	1,1646097	1,2065E-05	1,1646097	1,51867183	0,12884517
tRNA-Asn-2	-1,4083549	-194,54574	-1,6110362	-0,0001898	-1,6110362	-1,5170123	0,12926366
VC1767	1,39542853	13,0515417	1,38966181	1,2736E-05	1,38966181	1,51648214	0,12939753
VC1134	1,82392941	5,08429197	1,68545059	4,9613E-06	1,68545059	1,51543494	0,12966236
VCA0048	-1,265561	-23,296135	-1,3104364	-2,273E-05	-1,3104364	-1,5152525	0,12970852
VC0228	1,44628814	41,9847393	1,50578257	4,0969E-05	1,50578257	1,51505386	0,12975885
rpsQ	2,65142058	546,383959	2,54454289	0,00053317	2,54454289	1,51104124	0,13077803
VC2013	-1,179074	-265,95476	-1,4035632	-0,0002595	-1,4035632	-1,5086584	0,13138619
VCA0859	-1,4710977	-43,236807	-1,6000433	-4,219E-05	-1,6000433	-1,5076234	0,13165099
VC1069	1,62958036	4,10602996	1,54681059	4,0067E-06	1,54681059	1,50006979	0,13359638
VCA0656	-1,7234527	-33,846733	-1,9969886	-3,303E-05	-1,9969886	-1,4971796	0,13434659
VC0512	1,22867375	10,5973143	1,22724637	1,0341E-05	1,22724637	1,49680209	0,13444482
VC0186	1,17465111	22,112482	1,19850679	2,1578E-05	1,19850679	1,4958266	0,13469892
tRNA-Phe-2	-1,7505002	-51,890155	-1,7262627	-5,063E-05	-1,7262627	-1,4921534	0,13565903
VC1367	-1,3461459	-18,041229	-1,4312981	-1,76E-05	-1,4312981	-1,4903042	0,13614435
VC1463	-1,3384976	-5,3267371	-1,497131	-5,198E-06	-1,497131	-1,4900828	0,13620252
VC2735	1,82250704	15,4982271	1,73203101	1,5123E-05	1,73203101	1,49000417	0,13622323

VCA0947	-1,3958309	-42,857671	-1,457354	-4,182E-05	-1,457354	-1,489924	0,13624431
glnL	-1,236651	-4,9736079	-1,3495197	-4,853E-06	-1,3495197	-1,4898629	0,13626035
VC1451	1,64637248	3,53755233	1,52893295	3,452E-06	1,52893295	1,48986044	0,13626099
def (NC_002505 44278..44828)	1,1067917	14,1051077	1,11067102	1,3764E-05	1,11067102	1,48964949	0,13631648
VC0485	-2,2772073	-799,52347	-2,6476808	-0,0007802	-2,6476808	-1,4873129	0,13693226
VC0810	1,34677076	6,40000053	1,26027927	6,2452E-06	1,26027927	1,48694649	0,137029
VC1543	1,87453643	9,2669664	1,7950105	9,0428E-06	1,7950105	1,48361521	0,1379111
VC1062	1,62618808	7,64798621	1,49003395	7,463E-06	1,49003395	1,4803959	0,13876769
pyrG	-1,2735393	-109,88359	-1,3244899	-0,0001072	-1,3244899	-1,4797277	0,138946
tpiA	-1,4128176	-212,95676	-1,6338184	-0,0002078	-1,6338184	-1,4778637	0,13944431
ilvM	2,62594703	16,0188313	2,52382839	1,5631E-05	2,52382839	1,47454562	0,14033484
VCA0302	-1,1798292	-5,991928	-1,2740898	-5,847E-06	-1,2740898	-1,471946	0,14103551
moaD	1,53228764	6,59716409	1,48497321	6,4376E-06	1,48497321	1,47067278	0,1413797
VC2099	1,22840818	31,2807609	1,26816129	3,0524E-05	1,26816129	1,47051282	0,141423
VCA0338	1,31526488	6,9690712	1,23036972	6,8005E-06	1,23036972	1,46953376	0,14168812
VC0199	1,69382746	4,14069573	1,57221151	4,0405E-06	1,57221151	1,46791733	0,14212674
VC2118	-1,1901697	-11,760208	-1,2098018	-1,148E-05	-1,2098018	-1,4671064	0,14234715
fliR	-1,2589516	-6,963414	-1,3387184	-6,795E-06	-1,3387184	-1,4668065	0,14242874
pgk	-1,6793732	-433,27498	-1,9178489	-0,0004228	-1,9178489	-1,4664703	0,14252027
rplT	1,37210376	256,912856	1,2479167	0,0002507	1,2479167	1,46516721	0,14287537
VCA0608	1,46200991	27,8962352	1,44736496	2,7221E-05	1,44736496	1,46271048	0,14354671
VC2213	-1,1262734	-247,34637	-1,3657093	-0,0002414	-1,3657093	-1,4617775	0,14380227
rpmJ (NC_002505 2754084..2754238)	1,7151991	170,804574	1,60531669	0,00016667	1,60531669	1,46083443	0,14406095
VC2086	-2,2666524	-95,386412	-2,2064167	-9,308E-05	-2,2064167	-1,4589496	0,14457907
VCA0730	1,28558279	10,4609548	1,24139628	1,0208E-05	1,24139628	1,45882086	0,14461446
VC2615	-3,4227639	-27,280187	-3,6434169	-2,662E-05	-3,6434169	-1,45263	0,1463266
VC2172	1,39687064	8,99181316	1,32116314	8,7743E-06	1,32116314	1,45135047	0,14668237
VCA0729	1,35257671	10,7779444	1,28539814	1,0517E-05	1,28539814	1,44877248	0,14740119
rpsO	1,62291949	620,582618	1,59667138	0,00060557	1,59667138	1,4472373	0,14783057
VC1406	1,35919076	6,40220539	1,30189186	6,2473E-06	1,30189186	1,44622656	0,14811372
VC0446	1,17929664	10,7229153	1,13903531	1,0464E-05	1,13903531	1,44606626	0,14815866
sucC	-2,4305736	-148,35649	-2,4037903	-0,0001448	-2,4037903	-1,4455606	0,14830057
VC0430	1,20302302	27,9459243	1,19179434	2,727E-05	1,19179434	1,44457473	0,14857745
VC1443	2,04651683	11,2658981	1,84207237	1,0993E-05	1,84207237	1,44173411	0,14937748
VC0524	1,22371575	8,87356562	1,18832353	8,6589E-06	1,18832353	1,43979147	0,14992645
VC0336	-2,3417585	-383,81185	-2,3584209	-0,0003745	-2,3584209	-1,4394049	0,15003591
VCA0907	2,02149188	36,9388016	2,03961004	3,6045E-05	2,03961004	1,43849902	0,15029259
VC2048	-1,250143	-9,769016	-1,285507	-9,533E-06	-1,285507	-1,4384438	0,15030825
VC1478	-1,5694939	-34,759567	-1,5988204	-3,392E-05	-1,5988204	-1,4376265	0,15054011
pntB	-1,3653262	-132,48359	-1,5022688	-0,0001293	-1,5022688	-1,4372085	0,1506588
rplW	2,12456368	257,347576	2,03896453	0,00025112	2,03896453	1,43703274	0,15070875
VC2654	1,23592316	18,3923933	1,21913349	1,7947E-05	1,21913349	1,43608049	0,15097951
VC1479	1,18702095	24,286227	1,19700294	2,3699E-05	1,19700294	1,43500793	0,1512849
VC0125	1,21642224	16,5619545	1,19619099	1,6161E-05	1,19619099	1,43493719	0,15130506
VCA0300	-1,2818743	-18,300174	-1,3231907	-1,786E-05	-1,3231907	-1,433694	0,15165969

VCA0911	3,20100548	1,91521797	2,88073029	1,8689E-06	2,88073029	1,43252201	0,15199455
VC0504	-1,1978988	-9,153477	-1,2105026	-8,932E-06	-1,2105026	-1,4320809	0,15212072
VC1182	1,34079993	15,2569565	1,29863768	1,4888E-05	1,29863768	1,43191128	0,15216929
rpsD	1,31645394	398,759187	1,30949592	0,00038911	1,30949592	1,43035912	0,15261406
VCA0921	-1,2586262	-26,214771	-1,3038546	-2,558E-05	-1,3038546	-1,4283184	0,15320031
VCA0653	-1,6285323	-21,840856	-1,9085611	-2,131E-05	-1,9085611	-1,4281618	0,15324537
VC2293	1,19274205	45,3054055	1,1737808	4,4209E-05	1,1737808	1,42487778	0,15419261
VC0013	-1,1055634	-12,091227	-1,1185529	-1,18E-05	-1,1185529	-1,4248044	0,15421379
VC0395	1,39233048	37,7503777	1,3692057	3,6837E-05	1,3692057	1,42434473	0,1543468
VC1021	1,23376989	7,67122241	1,19291478	7,4856E-06	1,19291478	1,42289909	0,15476546
VC1449	2,28502561	50,8381625	2,30609761	4,9608E-05	2,30609761	1,42281215	0,15479072
VCA0342	1,28177433	7,54177375	1,22228044	7,3593E-06	1,22228044	1,42188881	0,15505857
VC2062	-1,1350707	-13,01375	-1,1312036	-1,27E-05	-1,1312036	-1,4214209	0,15519446
VC0334	-1,1579331	-6,2173416	-1,2428256	-6,067E-06	-1,2428256	-1,4210804	0,15529345
VCA0094	-1,4503534	-3,0943933	-1,5559464	-3,02E-06	-1,5559464	-1,4209884	0,15532016
VC0171	1,50414211	17,7859793	1,47175116	1,7356E-05	1,47175116	1,41925583	0,15582451
VCA0433	1,506367	16,8593391	1,4741068	1,6451E-05	1,4741068	1,41713108	0,1564447
VC0607	-1,643158	-2,34297	-1,7989026	-2,286E-06	-1,7989026	-1,4164393	0,15664699
VC1456	1,33644234	6,36374205	1,28173327	6,2098E-06	1,28173327	1,41638524	0,1566628
VC1883	1,31321822	6,66318029	1,25560239	6,502E-06	1,25560239	1,41545318	0,15693572
VC2103	1,76266123	3,93747474	1,56923018	3,8422E-06	1,56923018	1,413907	0,15738927
VC1099	1,62738454	12,1298293	1,45488139	1,1836E-05	1,45488139	1,41332209	0,1575611
VC0786	1,87520833	2,79778342	1,6244781	2,7301E-06	1,6244781	1,41320811	0,15759459
VCA1039	1,31856289	8,2817979	1,24754692	8,0814E-06	1,24754692	1,41216973	0,15790004
nagB	-1,158386	-13,256877	-1,1727168	-1,294E-05	-1,1727168	-1,4120338	0,15794007
VC0652	-1,4080514	-22,560689	-1,4316415	-2,201E-05	-1,4316415	-1,4114034	0,15812577
VC2565	-1,3636731	-21,297114	-1,4488651	-2,078E-05	-1,4488651	-1,4113468	0,15814242
VCA0806	-1,2226043	-13,895921	-1,2690935	-1,356E-05	-1,2690935	-1,4108018	0,15830311
VCA0908	2,06515605	26,8229133	2,03587579	2,6174E-05	2,03587579	1,40540532	0,15990087
VCA0446	2,47463776	495,838358	2,72477335	0,00048384	2,72477335	1,4051101	0,15998864
VC1588	1,74995905	5,60859568	1,66521363	5,4729E-06	1,66521363	1,40497468	0,16002888
VCA0056	1,53168273	3,71839027	1,42848042	3,6284E-06	1,42848042	1,4029384	0,16063526
VC0458	1,25243307	7,18592827	1,20069092	7,0121E-06	1,20069092	1,4021511	0,16087017
VC2221	-1,9886949	-1148,7239	-1,6677315	-0,0011209	-1,6677315	-1,4004436	0,16138061
VC0927	1,35898381	4,67851143	1,32408598	4,5653E-06	1,32408598	1,3990142	0,16180878
VC2476	1,26370084	12,7066088	1,22194702	1,2399E-05	1,22194702	1,39868062	0,16190884
VC2598	-1,1799948	-13,825246	-1,1828089	-1,349E-05	-1,1828089	-1,3972641	0,16233424
VC0843	1,65689795	5,78392145	1,582627	5,644E-06	1,582627	1,39480161	0,16307575
tRNA-Leu-10	-1,4820537	-64,665933	-1,4512878	-6,31E-05	-1,4512878	-1,3939354	0,16333721
VC1964	1,32877675	20,3557962	1,3248462	1,9863E-05	1,3248462	1,39161486	0,16403913
VC1001	1,19696382	10,7575479	1,16291622	1,0497E-05	1,16291622	1,39142514	0,1640966
VC2061	-1,2008532	-17,704509	-1,2009856	-1,728E-05	-1,2009856	-1,3910252	0,16421785
VC0754	1,33515313	7,57577819	1,31609322	7,3925E-06	1,31609322	1,38928251	0,16474692
VC0419	-1,1087054	-9,8451494	-1,1413655	-9,607E-06	-1,1413655	-1,3856657	0,16584905
VC0666	1,37272202	11,8225695	1,30018591	1,1537E-05	1,30018591	1,38563575	0,16585823

VC1060	1,17213508	8,76935196	1,15739237	8,5572E-06	1,15739237	1,38542958	0,16592121
VCA0458	1,31636258	5,94271029	1,25760894	5,7989E-06	1,25760894	1,38541767	0,16592484
tRNA-Arg-4	-1,6491953	-13,39608	-1,6481355	-1,307E-05	-1,6481355	-1,3851549	0,16600516
VC0422	-1,1304919	-8,1399681	-1,1705192	-7,943E-06	-1,1705192	-1,3851214	0,16601541
clpS	1,36394225	43,2716134	1,36161039	4,2225E-05	1,36161039	1,38392463	0,16638162
VC1678	1,40893365	10,586383	1,35804808	1,033E-05	1,35804808	1,3820594	0,16695353
glmU	1,14196106	10,0137615	1,13743119	9,7715E-06	1,13743119	1,38100725	0,16727678
VCA0088	2,3452544	17,3529839	2,15543377	1,6933E-05	2,15543377	1,38092576	0,16730188
VCA0652	1,5842812	17,6531198	1,53559546	1,7226E-05	1,53559546	1,38008747	0,16755978
pntA	-1,3407711	-94,485399	-1,3913006	-9,22E-05	-1,3913006	-1,3800336	0,16757634
VC0356	-1,5833004	-146,68681	-1,5459129	-0,0001431	-1,5459129	-1,3797222	0,16767228
VCA0299	-1,2901242	-14,493607	-1,3321169	-1,414E-05	-1,3321169	-1,3773613	0,16840065
VCA0897	2,03305662	20,0639745	2,00728918	1,9579E-05	2,00728918	1,37364282	0,16955268
VC1791	1,47407963	4,51787991	1,4010111	4,4086E-06	1,4010111	1,37344556	0,16961392
VC2627	1,29050421	10,8705214	1,23778496	1,0608E-05	1,23778496	1,37300795	0,16974993
VC1864	2,04525176	5,51853183	1,80251791	5,385E-06	1,80251791	1,37290873	0,1697808
VC1314	-1,4629743	-28,792396	-1,6114126	-2,81E-05	-1,6114126	-1,3726182	0,16987116
VC2386	1,50129665	78,3753751	1,56671757	7,6479E-05	1,56671757	1,37254185	0,16989487
VCA0200	1,27712821	43,8280205	1,38265887	4,2768E-05	1,38265887	1,37146106	0,17023133
era	1,31283823	12,0086196	1,26967311	1,1718E-05	1,26967311	1,3709355	0,17039512
VC0557	1,32887104	44,5442794	1,36160392	4,3467E-05	1,36160392	1,36888407	0,17103559
serB	1,47169266	9,8651079	1,39966335	9,6265E-06	1,39966335	1,36769706	0,17140699
VC1859	1,84708591	6,73419137	1,63725539	6,5713E-06	1,63725539	1,36642714	0,17180501
VC2154	1,09392103	14,5642177	1,1057393	1,4212E-05	1,1057393	1,36532753	0,17215018
glpX	-1,4010457	-13,134664	-1,401629	-1,282E-05	-1,401629	-1,3619825	0,17320345
VC2420	1,25814126	13,4503855	1,24472644	1,3125E-05	1,24472644	1,36184943	0,17324548
VC0885	1,61529301	5,52729132	1,48424122	5,3936E-06	1,48424122	1,36165249	0,17330763
VC0850	1,3609312	8,27846889	1,29845715	8,0782E-06	1,29845715	1,36023315	0,17375622
VCA1031	-1,4935664	-18,83805	-1,4687316	-1,838E-05	-1,4687316	-1,3589812	0,17415261
VC1141	1,33254401	36,1303565	1,29250786	3,5256E-05	1,29250786	1,35841829	0,17433109
fliS	-1,3012155	-33,128664	-1,2886921	-3,233E-05	-1,2886921	-1,3581225	0,17442489
VCA0901	-1,3147888	-3,6006869	-1,4095835	-3,514E-06	-1,4095835	-1,3550516	0,17540115
VC0812	1,41445617	11,1640964	1,34053855	1,0894E-05	1,34053855	1,35459239	0,17554751
VC1087	-1,2129492	-10,355903	-1,2848418	-1,011E-05	-1,2848418	-1,3539741	0,1757447
VCA1097	-1,6933753	-3,4498312	-1,7361341	-3,366E-06	-1,7361341	-1,352863	0,17609945
VCA0489	1,20972729	18,4107775	1,21904356	1,7965E-05	1,21904356	1,35269093	0,17615446
VC0245	1,50306061	242,215837	1,41454461	0,00023636	1,41454461	1,3526312	0,17617353
VC1124	-1,1969623	-5,2129867	-1,264467	-5,087E-06	-1,264467	-1,3514858	0,17653992
tRNA-Ser-5	-1,1791155	-22,685952	-1,1626479	-2,214E-05	-1,1626479	-1,3513679	0,17657768
VC0156	1,76052029	55,338025	1,82829612	5,3999E-05	1,82829612	1,34899228	0,17733952
VC0181	1,31687622	32,353482	1,3749016	3,1571E-05	1,3749016	1,348192	0,17759669
tRNA-Ser-4	1,15172595	10,133213	1,14725729	9,8881E-06	1,14725729	1,34791553	0,1776856
VCA0314	1,21809895	12,5615573	1,20529193	1,2258E-05	1,20529193	1,34393046	0,17897093
flgH	-1,2725401	-19,603158	-1,3339505	-1,913E-05	-1,3339505	-1,342605	0,17939996
VC0832	2,08827601	6,80762397	1,96527528	6,6429E-06	1,96527528	1,34234229	0,17948509

VCA0910	2,81255497	1,63501855	2,42858232	1,5955E-06	2,42858232	1,34187704	0,17963593
VC0105	1,32613097	13,8695572	1,2533457	1,3534E-05	1,2533457	1,34175048	0,17967698
VCA0329a	1,33214513	5,51213488	1,24398739	5,3788E-06	1,24398739	1,34090949	0,17994989
VC2058	-1,2601753	-46,939521	-1,2366044	-4,58E-05	-1,2366044	-1,3374085	0,18108943
VC0054	1,36015524	5,78666726	1,29473039	5,6467E-06	1,29473039	1,33685791	0,18126907
VCA1079	1,21707207	23,3670996	1,22777326	2,2802E-05	1,22777326	1,33651999	0,18137944
VC1838	1,11987632	12,0317185	1,13703036	1,1741E-05	1,13703036	1,33643537	0,18140707
rbsC	-1,4512366	-4,5843185	-1,6905382	-4,473E-06	-1,6905382	-1,3352597	0,18179142
VC0833	1,89445816	11,2823519	1,86365583	1,1009E-05	1,86365583	1,3343705	0,18208253
VC0166	1,49052365	20,5479644	1,49155179	2,0051E-05	1,49155179	1,33407831	0,18217828
tRNA-Ala-5	-1,5949838	-2,4925935	-1,6227375	-2,432E-06	-1,6227375	-1,3324859	0,18270061
VC0039	1,27147856	6,09036266	1,21487096	5,943E-06	1,21487096	1,33138471	0,18306252
VC1823	-1,688044	-14,473223	-2,0167296	-1,412E-05	-2,0167296	-1,3308121	0,1832509
nrdB	1,74432034	45,0371619	1,74748394	4,3948E-05	1,74748394	1,3293928	0,18371849
ruvB	1,35758559	5,47969234	1,30095934	5,3471E-06	1,30095934	1,32934189	0,18373526
metQ	1,92086279	66,536508	1,99394888	6,4927E-05	1,99394888	1,32918992	0,1837854
VC1100	1,43824456	5,74640452	1,30989175	5,6074E-06	1,30989175	1,32650968	0,18467098
VC2206	-1,2236061	-63,723012	-1,2776712	-6,218E-05	-1,2776712	-1,3261825	0,18477933
VC1874	-1,3966028	-3,0131632	-1,4826162	-2,94E-06	-1,4826162	-1,3256223	0,18496488
VCA0592	-1,1524875	-5,6433008	-1,2299192	-5,507E-06	-1,2299192	-1,3212232	0,18642701
dnaA	-1,1662248	-17,430107	-1,1828059	-1,701E-05	-1,1828059	-1,3189462	0,18718718
VCA0675	2,12234337	20,7077343	2,01722397	2,0207E-05	2,01722397	1,31838893	0,18737357
VC0688	1,28463247	6,6840051	1,19295266	6,5223E-06	1,19295266	1,31648896	0,18801003
VC0776	2,95395435	1,25582203	2,69408732	1,2254E-06	2,69408732	1,3144357	0,18869967
VCA0334	1,4195225	10,3814023	1,35566218	1,013E-05	1,35566218	1,31331116	0,18907819
VCA0190	1,22293107	7,37221242	1,16887574	7,1939E-06	1,16887574	1,31230848	0,18941611
VC0137	-1,2031306	-12,445877	-1,2744539	-1,214E-05	-1,2744539	-1,3118782	0,1895613
VC1688	-1,5982437	-2,0388509	-1,7828603	-1,99E-06	-1,7828603	-1,3117486	0,18960502
VC1450	2,1188158	20,3702423	2,12290499	1,9877E-05	2,12290499	1,31121218	0,18978618
VC1863	1,7673599	7,10058235	1,59545298	6,9288E-06	1,59545298	1,30907006	0,1905107
VC0058	1,21575032	8,46023747	1,17284866	8,2556E-06	1,17284866	1,30849827	0,19070441
VC0876	1,37433749	4,69512526	1,27670154	4,5815E-06	1,27670154	1,30840173	0,19073714
rpsA	1,71848319	1137,53052	1,99114468	0,00111001	1,99114468	1,30768156	0,19098143
VCA0230	2,48582486	4,53503486	2,2818	4,4253E-06	2,2818	1,307124	0,19117068
murP	3,16729511	8,71970395	2,63575437	8,5088E-06	2,63575437	1,30709549	0,19118037
VC0121	1,50572905	10,1268344	1,44522056	9,8819E-06	1,44522056	1,30616108	0,19149784
VCA0082	1,67110908	2,73056698	1,52528208	2,6645E-06	1,52528208	1,30535862	0,19177081
aspS	1,20988488	11,154507	1,18875515	1,0885E-05	1,18875515	1,30258691	0,19271588
thrA	1,91412119	13,0148174	1,84498985	1,27E-05	1,84498985	1,30151526	0,19308223
VCA0477	1,35424029	14,5409747	1,32737252	1,4189E-05	1,32737252	1,30009546	0,19356833
VC1802	1,93787321	2,12440969	1,72011903	2,073E-06	1,72011903	1,29893932	0,1939648
VC1044	1,37436193	6,02776095	1,26443271	5,8819E-06	1,26443271	1,29801821	0,19428113
VC0026	1,18791745	7,91386293	1,1526262	7,7224E-06	1,1526262	1,29746908	0,19446988
VC1740	2,19820433	3,28519489	1,94542141	3,2057E-06	1,94542141	1,29745373	0,19447518
VCA0637	-1,3646818	-14,059644	-1,4090112	-1,372E-05	-1,4090112	-1,2964584	0,19481769

rplA	1,69246692	355,976693	1,53717968	0,00034737	1,53717968	1,2944926	0,19549539
VC0320	-1,1337272	-7,1870648	-1,1684559	-7,013E-06	-1,1684559	-1,2942378	0,19558334
VC1045	1,40993341	9,82719731	1,31838162	9,5895E-06	1,31838162	1,29140205	0,19656436
VC1625	2,91569446	8,73185351	2,80536499	8,5206E-06	2,80536499	1,29013023	0,19700553
VCA0883	1,36095584	4,23605906	1,30131759	4,1336E-06	1,30131759	1,28993872	0,19707198
VCA0627	1,71127588	2,52740556	1,5604089	2,4663E-06	1,5604089	1,28824132	0,19766202
VC0247	1,44469834	230,460233	1,36288169	0,00022489	1,36288169	1,28777475	0,19782446
VC0608	2,86508318	24,2987229	2,81734477	2,3711E-05	2,81734477	1,28730322	0,19798871
VCA0732	1,84601984	29,2431296	1,82986965	2,8536E-05	1,82986965	1,28708217	0,19806574
VCA0581	1,32856486	4,96135661	1,25044447	4,8413E-06	1,25044447	1,28702565	0,1980854
VC0891	1,49289418	7,35042553	1,39304349	7,1726E-06	1,39304349	1,28643028	0,198293
VC2492	1,36819345	6,59277129	1,31127861	6,4333E-06	1,31127861	1,2854176	0,19864645
VCA0113	1,61395087	3,57282569	1,46761062	3,4864E-06	1,46761062	1,28403715	0,19912901
uvrB	1,34285864	4,70343761	1,26429473	4,5897E-06	1,26429473	1,28336568	0,19936404
VC1454	-1,288449	-7,4235155	-1,3303791	-7,244E-06	-1,3303791	-1,2824817	0,19967378
VC0357	-1,6150321	-55,27196	-1,5347069	-5,393E-05	-1,5347069	-1,2817864	0,19991768
VC0459	1,24228312	5,88033211	1,20503663	5,7381E-06	1,20503663	1,28078472	0,20026934
VC0869	1,59968209	4,56698819	1,53835289	4,4565E-06	1,53835289	1,28018123	0,20048147
VC0836	1,76755598	5,66706358	1,707234	5,53E-06	1,707234	1,27825193	0,20116068
VC1899	1,48978678	6,12100598	1,37678084	5,9729E-06	1,37678084	1,27817875	0,20118647
VC2098	1,25645013	20,6749091	1,26225455	2,0175E-05	1,26225455	1,27806438	0,2012268
VCA0678	3,09801392	58,8322026	3,10873947	5,7409E-05	3,10873947	1,27804706	0,20123291
VC0029	1,96154107	10,0819148	1,83827809	9,838E-06	1,83827809	1,27686648	0,20164947
VC1912	-1,1712541	-20,38592	-1,1598139	-1,989E-05	-1,1598139	-1,2758549	0,2020069
VCA0482	1,41785243	4,84245528	1,37845704	4,7253E-06	1,37845704	1,26929932	0,20433438
acpS	1,29067209	29,1756535	1,29133806	2,847E-05	1,29133806	1,26904814	0,20442398
sspA	-1,1786618	-19,498816	-1,145103	-1,903E-05	-1,145103	-1,266627	0,20528875
VCA0938	-1,3345429	-5,1500786	-1,3723901	-5,025E-06	-1,3723901	-1,2659682	0,2055245
VC2266	-1,267562	-6,5657877	-1,2991832	-6,407E-06	-1,2991832	-1,2656898	0,20562419
VCr025	-1,1490794	-7758,7819	-1,3940015	-0,0075711	-1,3940015	-1,2645896	0,20601858
VC2297	1,50465089	12,0226237	1,3756495	1,1732E-05	1,3756495	1,26277847	0,20666888
VCA0610	-1,3110612	-27,450027	-1,4237151	-2,679E-05	-1,4237151	-1,2626689	0,20670829
VC2124	-1,1830725	-15,710766	-1,2117059	-1,533E-05	-1,2117059	-1,2624882	0,20677324
VC1095	1,30050414	6,43607624	1,22756627	6,2804E-06	1,22756627	1,26231774	0,20683452
VC1195	1,73387767	29,7609966	1,73028888	2,9041E-05	1,73028888	1,25900872	0,20802727
VCA1029	-1,4459742	-33,998173	-1,5787167	-3,318E-05	-1,5787167	-1,2583582	0,20826234
VC2282	-1,2777403	-3,5949093	-1,3438783	-3,508E-06	-1,3438783	-1,2578884	0,20843216
VC1578	1,60904423	499,833669	1,505837	0,00048774	1,505837	1,25667477	0,2088715
VC1137	1,73219867	9,76468079	1,71019272	9,5285E-06	1,71019272	1,2565494	0,20891693
VCA0913	2,29076888	2,1869483	2,15272211	2,134E-06	2,15272211	1,25602123	0,20910834
VC2491	1,45298257	4,93283828	1,34153863	4,8135E-06	1,34153863	1,25520359	0,20940492
VC0417	-1,1084986	-7,6975974	-1,146084	-7,511E-06	-1,146084	-1,2538001	0,20991473
VC1070	1,51925715	3,26685758	1,43748995	3,1878E-06	1,43748995	1,25352242	0,2100157
VC0373	-1,2078885	-7,533946	-1,2524723	-7,352E-06	-1,2524723	-1,2511408	0,21088315
VC2555	1,28852403	7,58882441	1,20648557	7,4052E-06	1,20648557	1,25003281	0,21128761

VC1373	1,60014681	2,72572518	1,47195157	2,6598E-06	1,47195157	1,24948765	0,21148683
truD	1,31799169	4,96572719	1,25836196	4,8456E-06	1,25836196	1,24900736	0,21166243
VCA0814	1,27855469	15,4345179	1,25608928	1,5061E-05	1,25608928	1,24809837	0,21199509
VC0025	1,28792159	8,79555356	1,21644925	8,5828E-06	1,21644925	1,24797177	0,21204145
VC0469	1,43014857	3,81847977	1,31451613	3,7261E-06	1,31451613	1,24767153	0,21215142
VC1254	1,59408699	29,9243637	1,57983579	2,92E-05	1,57983579	1,24646619	0,21259336
hisH	1,70173956	13,992467	1,67797811	1,3654E-05	1,67797811	1,24598496	0,21277
VC1985	1,51582375	13,7907522	1,39231842	1,3457E-05	1,39231842	1,2451731	0,2130682
hemH (NC_002505 1264415..1265562)	-1,383013	-22,17521	-1,4737439	-2,164E-05	-1,4737439	-1,2447	0,21324208
VC0055	1,37812794	4,2174981	1,29059977	4,1155E-06	1,29059977	1,24170423	0,21434575
VC1758	1,54991836	7,43941624	1,44318179	7,2594E-06	1,44318179	1,24112518	0,21455958
rplF	1,59896872	357,282165	1,42454612	0,00034864	1,42454612	1,24081861	0,21467285
VC1330	-1,2936301	-2,795528	-1,4469411	-2,728E-06	-1,4469411	-1,2376778	0,2158356
VCA1024	-1,1717379	-5,0157343	-1,2745556	-4,894E-06	-1,2745556	-1,2371229	0,21604151
VC2454	-1,17711	-4,2083973	-1,2756666	-4,107E-06	-1,2756666	-1,236689	0,21620261
VCA1113	1,63708406	7,72715329	1,58156366	7,5402E-06	1,58156366	1,23605545	0,21643802
rpmE2	1,40459126	4,91401722	1,31171382	4,7951E-06	1,31171382	1,23555525	0,21662399
VCA0172	1,2866673	5,69048766	1,19612432	5,5528E-06	1,19612432	1,23475116	0,21692317
VCA0238	-1,3643006	-4,0763681	-1,3829614	-3,978E-06	-1,3829614	-1,2344919	0,2170197
gltB	1,63133696	4,48353741	1,50973092	4,3751E-06	1,50973092	1,23390744	0,21723745
VC0765	-1,4700044	-55,347634	-1,4379755	-5,401E-05	-1,4379755	-1,2338561	0,21725659
VC1695	1,35203296	4,14755298	1,27868664	4,0472E-06	1,27868664	1,23360471	0,21735028
VC1844	1,54639368	86,614196	1,58378207	8,4519E-05	1,58378207	1,23354756	0,21737163
VC0771	1,76739046	2,23901985	1,58573844	2,1849E-06	1,58573844	1,23353157	0,21737756
VCA0332	1,39381669	79,1268438	1,23760144	7,7213E-05	1,23760144	1,23337346	0,21743651
VC2655	1,47695633	6,16813261	1,4351886	6,0189E-06	1,4351886	1,23332633	0,2174541
VC1837	1,16625769	17,7866077	1,18088178	1,7356E-05	1,18088178	1,23228277	0,21784354
VC0420	-1,1374295	-5,6974681	-1,1949549	-5,56E-06	-1,1949549	-1,2321476	0,217894
VC1025	1,53391386	7,33537978	1,48587148	7,1579E-06	1,48587148	1,23057447	0,21848212
vmrA	-1,2036679	-4,0582487	-1,2839071	-3,96E-06	-1,2839071	-1,2302189	0,2186152
VCA0588	-1,1459882	-4,7086813	-1,2398241	-4,595E-06	-1,2398241	-1,2298566	0,21875085
VC0719	1,35917806	8,66592675	1,30407694	8,4563E-06	1,30407694	1,22901354	0,21906679
VC1770	1,32591237	12,7749791	1,31099812	1,2466E-05	1,31099812	1,22704768	0,21980474
VC1987	1,21023805	6,5476542	1,16568217	6,3893E-06	1,16568217	1,22589463	0,22023837
VC2188	-1,2055713	-96,000644	-1,3110098	-9,368E-05	-1,3110098	-1,2254178	0,22041791
surE	1,28711845	5,91571282	1,23200085	5,7726E-06	1,23200085	1,2250443	0,22055859
dapA	1,26878322	46,0188567	1,28125238	4,4906E-05	1,28125238	1,22477809	0,22065892
hslO	1,61378304	7,49100955	1,47707426	7,3098E-06	1,47707426	1,22419399	0,22087912
VC1771	1,3379561	6,84100565	1,25690247	6,6755E-06	1,25690247	1,22337917	0,22118656
VC2507	-1,1926164	-5,4393026	-1,2998049	-5,308E-06	-1,2998049	-1,223115	0,2212863
VCA0396	1,20321474	11,7754597	1,15681947	1,1491E-05	1,15681947	1,22105865	0,22206387
VCA0058	1,19371196	7,23888073	1,14718708	7,0638E-06	1,14718708	1,22013386	0,22241418
VC2059	-1,1754104	-77,539389	-1,2134948	-7,566E-05	-1,2134948	-1,2184769	0,22304285
VC0552	1,20471965	7,03167677	1,15115973	6,8616E-06	1,15115973	1,21753781	0,2233997
VC2049	-1,5904991	-43,828043	-1,5265261	-4,277E-05	-1,5265261	-1,216989	0,22360845

tRNA-His-1	-1,3657278	-9,5084764	-1,3885706	-9,278E-06	-1,3885706	-1,2166872	0,22372332
leuD	1,32830562	9,80597137	1,27750043	9,5688E-06	1,27750043	1,21667762	0,22372696
VC1464	-1,1998172	-28,595113	-1,1716241	-2,79E-05	-1,1716241	-1,2158497	0,22404225
glnA	-1,3868243	-174,99045	-1,4651252	-0,0001708	-1,4651252	-1,2132173	0,2250468
VC1279	-1,4259369	-13,021091	-1,4652817	-1,271E-05	-1,4652817	-1,2078332	0,22711149
VC0238	1,2235571	41,6285943	1,32313804	4,0622E-05	1,32313804	1,20693657	0,22745662
VCA0347	1,21112713	6,28162738	1,16706287	6,1297E-06	1,16706287	1,2053401	0,22807208
VCA0372	-1,4489682	-23,455136	-1,4149875	-2,289E-05	-1,4149875	-1,2025745	0,22914109
tuf (NC_002505 335305..336530)	1,24016763	666,024964	1,40521774	0,00064991	1,40521774	1,19919212	0,2304533
VC0184	1,5595466	3,0945689	1,42305336	3,0197E-06	1,42305336	1,19839692	0,23076257
VC0170	1,67008136	5,34561831	1,47819526	5,2163E-06	1,47819526	1,19839644	0,23076279
VC2429	1,11563107	9,36440873	1,10763297	9,1379E-06	1,10763297	1,19783077	0,23098295
VC1457	1,27540774	7,53266452	1,22975467	7,3504E-06	1,22975467	1,19604711	0,23167823
VC0167	1,26717917	5,49253644	1,21121237	5,3597E-06	1,21121237	1,19572588	0,23180359
VCA0909	1,86255224	1,9894877	1,65410346	1,9414E-06	1,65410346	1,19493626	0,23211198
flgJ	-1,2514724	-25,517143	-1,2979291	-2,49E-05	-1,2979291	-1,194079	0,23244715
murQ	6,21657164	9,04667967	4,80139809	8,8278E-06	4,80139809	1,19283948	0,23293233
VCA0167	1,14925339	20,1270672	1,17186489	1,964E-05	1,17186489	1,19180989	0,23333584
nhaR	1,39186608	7,05845582	1,3157105	6,8877E-06	1,3157105	1,19137923	0,23350481
VCA0507	-1,4109011	-22,246246	-1,3858221	-2,171E-05	-1,3858221	-1,1908451	0,23371446
VCA0726	-1,2713963	-4,0785081	-1,306349	-3,98E-06	-1,306349	-1,1900931	0,23400983
VC1139	1,5490664	13,8338091	1,51036597	1,3499E-05	1,51036597	1,1898669	0,23409878
tRNA-Leu-4	-1,4112384	-12,682727	-1,4083589	-1,238E-05	-1,4083589	-1,1873442	0,23509196
mdoG	1,23650237	12,0202173	1,20351097	1,1729E-05	1,20351097	1,18718279	0,23515559
VCA1086	-1,2590239	-3,3413293	-1,3270829	-3,26E-06	-1,3270829	-1,1869866	0,23523295
VCA0258	1,62906452	2,6463003	1,4310353	2,5823E-06	1,4310353	1,18643134	0,23545206
VCA0298	-1,120272	-4,8181713	-1,2157375	-4,702E-06	-1,2157375	-1,1863441	0,23548647
tRNA-Gly-2	-1,5125354	-25,791458	-1,4757332	-2,517E-05	-1,4757332	-1,1855173	0,23581305
VC2019	-1,3137272	-49,305853	-1,3264072	-4,811E-05	-1,3264072	-1,1853762	0,23586879
VC0204	2,65416096	5,3222375	2,22746436	5,1935E-06	2,22746436	1,18220967	0,2371226
VCA0756	1,80868676	2,06321285	1,58307841	2,0133E-06	1,58307841	1,18202959	0,23719401
VCA0480	1,44282242	3,58341399	1,38603234	3,4967E-06	1,38603234	1,18187194	0,23725657
VC2412	1,50959075	104,319695	1,47434529	0,0001018	1,47434529	1,1815903	0,23736835
potA	1,31329013	17,1986712	1,31161698	1,6783E-05	1,31161698	1,18052237	0,23779259
VCA0229	2,37809677	1,34987744	2,23398182	1,3172E-06	2,23398182	1,17819063	0,23872067
16Sf	-1,2157726	-27,454571	-1,1921799	-2,679E-05	-1,1921799	-1,1781615	0,2387323
rnc	1,27256346	10,0070296	1,24251362	9,7649E-06	1,24251362	1,17717116	0,23912727
tRNA-Phe-3	-1,5460292	-8,3329878	-1,5926373	-8,131E-06	-1,5926373	-1,176909	0,23923191
VCA0835	-1,1645359	-4,5511695	-1,2257242	-4,441E-06	-1,2257242	-1,1767394	0,23929957
VC2060	-1,1820969	-24,751422	-1,1558564	-2,415E-05	-1,1558564	-1,1758217	0,2396662
VC0355	-1,5604713	-53,349894	-1,4670705	-5,206E-05	-1,4670705	-1,1753534	0,23985343
VCA0376	1,81546141	1,61555581	1,79296011	1,5765E-06	1,79296011	1,17304741	0,24077684
VCA0745	-2,1320243	-80,867471	-2,6113704	-7,891E-05	-2,6113704	-1,1717926	0,24128037
VC2723	-1,1413503	-5,1434009	-1,1951685	-5,019E-06	-1,1951685	-1,1712859	0,24148391
VC0421	-1,1100992	-6,8835564	-1,1419025	-6,717E-06	-1,1419025	-1,1696966	0,24212313

VCA0123	1,26705863	4,94129202	1,20887047	4,8218E-06	1,20887047	1,16922039	0,24231489
VC1101	1,40821737	4,04567144	1,27556446	3,9478E-06	1,27556446	1,16602837	0,24360302
VC0916	2,26695812	1,37393409	2,12958338	1,3407E-06	2,12958338	1,16578969	0,24369953
VC0089	-1,3601761	-2,2885544	-1,4922686	-2,233E-06	-1,4922686	-1,1645179	0,24421426
lpxD	1,22565552	37,4209498	1,21870767	3,6516E-05	1,21870767	1,16251338	0,24502704
VC1113	8,40583059	19,1450746	8,22933509	1,8682E-05	8,22933509	1,16109759	0,2456023
VCA0764	1,30557151	7,79747582	1,22002241	7,6088E-06	1,22002241	1,16086581	0,24569653
VCA0349	1,29100896	4,82102423	1,20530119	4,7044E-06	1,20530119	1,16037296	0,24589703
uppP	1,26131521	4,95361793	1,19887668	4,8338E-06	1,19887668	1,15936244	0,24630852
VC2083	-1,3351928	-2,5479181	-1,4246901	-2,486E-06	-1,4246901	-1,1570786	0,24724028
xseA	-1,3210058	-13,404725	-1,3507496	-1,308E-05	-1,3507496	-1,1568081	0,24735083
VCA0912	2,1785413	3,97472956	2,03255228	3,8786E-06	2,03255228	1,15665001	0,24741545
VC0734	2,07319032	1,76494971	1,80198442	1,7223E-06	1,80198442	1,15659562	0,24743766
VC0845	1,46037762	10,7815318	1,41905157	1,0521E-05	1,41905157	1,15623564	0,24758486
VCA0184	-1,4744769	-688,28444	-1,4049911	-0,0006716	-1,4049911	-1,1562131	0,24759406
VC1903	1,23698538	6,84469885	1,17764865	6,6791E-06	1,17764865	1,15570862	0,2478004
VCA0545	1,36242885	11,1670693	1,31289208	1,0897E-05	1,31289208	1,15415153	0,24843811
VC0730	-1,3688864	-4,4901322	-1,4250261	-4,382E-06	-1,4250261	-1,1539788	0,24850894
VCA0951	1,49145217	18,7317331	1,46359393	1,8279E-05	1,46359393	1,15333279	0,24877387
VCA0443	1,4344734	6,53793364	1,36018747	6,3798E-06	1,36018747	1,15319962	0,24882852
VCA0227	2,49424319	7,63203881	2,35338437	7,4474E-06	2,35338437	1,15297941	0,24891892
rplM	1,24483377	806,277974	1,50883334	0,00078677	1,50883334	1,15279988	0,24899262
VC0358	-1,4813247	-32,346252	-1,4261738	-3,156E-05	-1,4261738	-1,1473255	0,2512472
rplD	1,75245736	161,572094	1,69459585	0,00015766	1,69459585	1,14729355	0,25126037
VC2204	-1,2923119	-32,520605	-1,2650949	-3,173E-05	-1,2650949	-1,1464821	0,2515958
VCA1074	1,31672987	4,15160953	1,23592611	4,0512E-06	1,23592611	1,14638995	0,25163388
VC0961	1,20966796	10,4316491	1,18526155	1,0179E-05	1,18526155	1,1458957	0,25183836
aroB	1,30864605	10,3830197	1,25355015	1,0132E-05	1,25355015	1,14561437	0,25195482
VC2269	-1,1168009	-11,460415	-1,1805662	-1,118E-05	-1,1805662	-1,1446491	0,25235459
VC2159	1,18065236	25,3921163	1,19344909	2,4778E-05	1,19344909	1,14350341	0,25282969
hfq	-1,0637216	-97,853755	-1,2241267	-9,549E-05	-1,2241267	-1,1431728	0,25296694
VCA0444	1,36106749	7,32581015	1,29544663	7,1486E-06	1,29544663	1,14207251	0,25342394
VC2050	-1,5964674	-88,066726	-1,5211767	-8,594E-05	-1,5211767	-1,1408613	0,2539277
VC2116	1,38851442	16,2564695	1,33402882	1,5863E-05	1,33402882	1,14057809	0,25404561
VC0296	1,37788914	46,7555741	1,39828366	4,5624E-05	1,39828366	1,1393583	0,25455382
VC1149	1,32659094	9,88008899	1,24669515	9,6411E-06	1,24669515	1,13829995	0,25499531
VCA0163	-1,0977253	-5,8084235	-1,2034632	-5,668E-06	-1,2034632	-1,1379273	0,2551509
VC0604	1,34158759	23,9454287	1,28303729	2,3366E-05	1,28303729	1,13732678	0,25540178
VC0928	1,50436992	6,55224687	1,48482871	6,3937E-06	1,48482871	1,13658759	0,25571079
VC0365	1,36074815	15,8495338	1,35233004	1,5466E-05	1,35233004	1,13633417	0,2558168
VC2340	-1,1825872	-3,4551351	-1,2843184	-3,372E-06	-1,2843184	-1,1358482	0,25602015
VC0271	2,13233662	19,2230831	1,90234114	1,8758E-05	1,90234114	1,13535605	0,25622625
tRNA-Asn-4	-1,380469	-99,67388	-1,4242762	-9,726E-05	-1,4242762	-1,1337366	0,25690516
VCA0736	-1,115471	-5,8596969	-1,1575937	-5,718E-06	-1,1575937	-1,1331604	0,25714694
VC1037	1,44778067	5,68874055	1,3228934	5,5511E-06	1,3228934	1,13287455	0,257267

VC2520	1,30184548	5,68230631	1,23021552	5,5448E-06	1,23021552	1,13287015	0,25726884
frsA	1,36393857	3,79159526	1,25422488	3,6999E-06	1,25422488	1,13262365	0,25737238
VC2391	1,22860232	32,8699355	1,25425791	3,2075E-05	1,25425791	1,13162083	0,25779395
asnC	1,21344067	12,4483144	1,1649206	1,2147E-05	1,1649206	1,13089721	0,25809841
VC0838	1,89257258	7,29299722	1,85004949	7,1166E-06	1,85004949	1,12998123	0,25848421
VC2360	1,55499642	9,01238113	1,52847172	8,7944E-06	1,52847172	1,12984249	0,25854268
VCA0266	1,68572883	2,31397253	1,45064144	2,258E-06	1,45064144	1,12983957	0,25854387
VC1841	1,58306568	36,9351224	1,62921358	3,6042E-05	1,62921358	1,12962608	0,2586339
VC1886	1,34806173	3,41256054	1,2847213	3,33E-06	1,2847213	1,12952831	0,25867508
VC0823	1,80521248	50,7450614	1,84273815	4,9517E-05	1,84273815	1,12950879	0,25868334
potB	1,35028952	13,3399979	1,34214562	1,3017E-05	1,34214562	1,12712871	0,25968813
tRNA-Gly-1	-1,6139958	-29,180906	-1,5532246	-2,847E-05	-1,5532246	-1,126857	0,25980303
VC0097	1,14620618	7,23550497	1,12423065	7,0605E-06	1,12423065	1,12673491	0,25985462
VCA0479	1,31059466	14,2494598	1,2891819	1,3905E-05	1,2891819	1,12665907	0,25988672
VC1638	1,42853669	2,78608399	1,35689261	2,7187E-06	1,35689261	1,12501575	0,2605824
VC1043	-1,1563919	-146,12669	-1,3612217	-0,0001426	-1,3612217	-1,1249171	0,26062421
VC2514	1,11598897	11,7711852	1,09677187	1,1486E-05	1,09677187	1,12454165	0,26078336
tRNA-Pro-2	-1,4879052	-79,321208	-1,4811284	-7,74E-05	-1,4811284	-1,1232749	0,26132084
VC2352	-1,2540473	-82,414227	-1,3496562	-8,042E-05	-1,3496562	-1,1229521	0,26145787
VCA0448	2,03729755	44,3316832	2,00814348	4,3259E-05	2,00814348	1,12282996	0,2615098
VC1271	-1,2409648	-2,7893755	-1,3544266	-2,722E-06	-1,3544266	-1,1223716	0,26170451
minE	1,34929614	3,83633201	1,24553354	3,7435E-06	1,24553354	1,12180695	0,26194458
VCA0398	1,32832014	5,51901579	1,22596936	5,3855E-06	1,22596936	1,12151027	0,26207076
VC1177	1,31947659	6,68220614	1,23745116	6,5206E-06	1,23745116	1,12039567	0,26254524
VCA0204	-1,3420313	-9,7517019	-1,4412063	-9,516E-06	-1,4412063	-1,1144518	0,26508547
rpsN	1,41116047	127,352007	1,27861205	0,00012427	1,27861205	1,11377559	0,26537553
VC0048	1,52820976	22,847532	1,47772699	2,2295E-05	1,47772699	1,11311819	0,26565774
VC1233	1,39268865	4,58158155	1,31457409	4,4707E-06	1,31457409	1,11267004	0,26585022
VCA0124	1,15441283	13,9602842	1,16924686	1,3623E-05	1,16924686	1,11241305	0,26596066
VC1572	1,8350006	1,54778246	1,72378611	1,5103E-06	1,72378611	1,11079744	0,26665559
nhaA	1,34535621	3,67416955	1,28507587	3,5853E-06	1,28507587	1,11026817	0,26688353
rhtB	-1,1695695	-7,5977258	-1,2876173	-7,414E-06	-1,2876173	-1,1088408	0,26749894
VC2164	1,26597345	4,94135991	1,18083168	4,8218E-06	1,18083168	1,10870604	0,26755706
VC0483	1,33288977	16,3940362	1,31044984	1,5997E-05	1,31044984	1,10817793	0,26778503
VC0242	1,33377019	106,307781	1,27655411	0,00010374	1,27655411	1,10800467	0,26785987
VCA0475	1,13543114	25,4498813	1,13743375	2,4834E-05	1,13743375	1,10794996	0,26788348
VC0515	1,74154048	9,54143172	1,70870896	9,3106E-06	1,70870896	1,10657938	0,2684759
VC0150	1,13064555	28,3105406	1,08604935	2,7626E-05	1,08604935	1,10621082	0,26863534
VC0821	2,21073141	25,6236563	2,20936924	2,5004E-05	2,20936924	1,10619403	0,26864262
VCA0400	1,30487646	14,9266964	1,29731432	1,4566E-05	1,29731432	1,10440521	0,26941747
VC1112	9,68537801	39,2741517	9,43998623	3,8324E-05	9,43998623	1,10400227	0,26959224
yieM	1,36630049	5,3191887	1,32032	5,1905E-06	1,32032	1,10358866	0,26977167
rplS	1,5398522	129,721389	1,33988415	0,00012658	1,33988415	1,10283684	0,27009809
flgG	-1,1838783	-20,747406	-1,2138133	-2,025E-05	-1,2138133	-1,1028017	0,27011335
VCA0459	1,27768384	4,11730399	1,21776321	4,0177E-06	1,21776321	1,10130153	0,27076549

VCA0504	1,25192311	4,73011959	1,18671086	4,6157E-06	1,18671086	1,10075764	0,2710022
modB	-1,199094	-3,4081567	-1,2872735	-3,326E-06	-1,2872735	-1,0998848	0,27138237
trpA	-1,1047866	-4,9388893	-1,1775186	-4,819E-06	-1,1775186	-1,0990634	0,27174045
pheS	1,2958606	6,41212078	1,23614276	6,257E-06	1,23614276	1,09808767	0,27216625
VCA0432	1,31096135	14,040141	1,31028726	1,37E-05	1,31028726	1,09637345	0,27291545
VC2222	1,3399948	3,70930648	1,24183472	3,6196E-06	1,24183472	1,09564083	0,27323603
fliD	-1,1770248	-15,798684	-1,2059998	-1,542E-05	-1,2059998	-1,0934745	0,27418557
VC1509	-1,2160714	-3,2060952	-1,3194645	-3,129E-06	-1,3194645	-1,0922587	0,27471943
VC1983	-1,0842637	-7,0428282	-1,1196557	-6,872E-06	-1,1196557	-1,0921481	0,27476806
fur	-1,1513244	-121,53327	-1,3306122	-0,0001186	-1,3306122	-1,0914614	0,27507
VC0091	1,44458065	3,31667358	1,30669678	3,2364E-06	1,30669678	1,09097285	0,27528488
rplQ	1,3214016	75,0715681	1,16506705	7,3255E-05	1,16506705	1,090743	0,27538603
VC1769	1,36166807	17,3478383	1,36603216	1,6928E-05	1,36603216	1,0873087	0,27690044
VCA0933	-1,3470741	-379,56487	-1,5054463	-0,0003704	-1,5054463	-1,085985	0,2774857
VC0037	1,24360933	29,2290462	1,28292105	2,8522E-05	1,28292105	1,08593285	0,27750875
VC0840	1,71994939	14,3418985	1,70082149	1,3995E-05	1,70082149	1,0844276	0,27817533
VCA0421	1,2325977	5,02060078	1,16918899	4,8991E-06	1,16918899	1,08388371	0,27841641
VC1111	2,24788394	3,24702131	2,21939192	3,1685E-06	2,21939192	1,08317003	0,27873303
VC2294	1,11493592	26,7073059	1,13448856	2,6061E-05	1,13448856	1,08245588	0,27905007
VCA0199	1,29217974	30,9797985	1,33174132	3,023E-05	1,33174132	1,08225306	0,27914014
pspB	1,33266947	6,38003	1,28595266	6,2257E-06	1,28595266	1,08204777	0,27923136
VC0028	1,98899686	11,1991713	1,95351716	1,0928E-05	1,95351716	1,0799059	0,28018416
VC0951	1,21932459	5,43083895	1,153884	5,2995E-06	1,153884	1,07891631	0,28062508
VC1862	1,66713116	2,02942473	1,47223111	1,9803E-06	1,47223111	1,07840287	0,28085405
VC0980	1,44655746	4,39289591	1,31237038	4,2866E-06	1,31237038	1,07797998	0,28104274
VC1717	1,26728767	14,6608464	1,24664431	1,4306E-05	1,24664431	1,07774105	0,2811494
tRNA-Met-5	-1,2071324	-4,4168022	-1,2607326	-4,31E-06	-1,2607326	-1,0776858	0,28117405
VCA0955	-1,2300466	-9,946256	-1,3008569	-9,706E-06	-1,3008569	-1,0774499	0,28127936
VC0977	2,13555966	14,991466	1,91574639	1,4629E-05	1,91574639	1,07659992	0,2816591
VCA0171	1,3086424	4,04107135	1,21124101	3,9433E-06	1,21124101	1,07617893	0,28184728
VCA0205	-1,4741176	-186,62497	-1,7950998	-0,0001821	-1,7950998	-1,0756243	0,28209536
VCA1072	-3,184438	-43,623736	-3,354453	-4,257E-05	-3,354453	-1,0754801	0,28215991
VC2046	-1,1776646	-5,8090229	-1,2376297	-5,668E-06	-1,2376297	-1,0746033	0,28255242
VCA0201	1,22824124	34,3058656	1,29954125	3,3476E-05	1,29954125	1,07391454	0,28286103
VC1075	-1,1820184	-3,2165613	-1,2712001	-3,139E-06	-1,2712001	-1,0734372	0,28307504
VCA0051	-1,1760665	-15,116448	-1,2411998	-1,475E-05	-1,2411998	-1,0733104	0,28313191
VC0514	1,76642912	12,0001346	1,74820794	1,171E-05	1,74820794	1,072677	0,28341613
VC1686	1,19215542	11,6493175	1,19602752	1,1368E-05	1,19602752	1,07235276	0,28356168
oppB	1,33529029	8,2533111	1,24530612	8,0537E-06	1,24530612	1,06966957	0,28476812
VC0187	1,16648274	5,93027753	1,13729947	5,7868E-06	1,13729947	1,06907875	0,28503423
VC2385	1,28583252	44,5455935	1,33047982	4,3468E-05	1,33047982	1,06766672	0,28567093
tRNA-Thr-6	-1,4766974	-22,247622	-1,4500569	-2,171E-05	-1,4500569	-1,0675945	0,28570352
VCA0985	-1,3639123	-3,9461978	-1,5214912	-3,851E-06	-1,5214912	-1,0674933	0,28574921
VC1880	-1,167832	-6,1019533	-1,2267738	-5,954E-06	-1,2267738	-1,0660928	0,28638173
VC0399	-1,0736977	-5,3400411	-1,1525485	-5,211E-06	-1,1525485	-1,0655368	0,28663311

VC1963	1,21467734	8,26748412	1,18020711	8,0675E-06	1,18020711	1,06536262	0,28671191
VC1455	-1,2293032	-30,729107	-1,1802828	-2,999E-05	-1,1802828	-1,0653016	0,28673953
23Sh	1,06915797	1579,79609	1,24272814	0,00154158	1,24272814	1,06422331	0,28722761
rpsB	1,62524647	196,757134	1,33404626	0,000192	1,33404626	1,06340993	0,28759617
VC0976	1,30458878	22,7422852	1,25820474	2,2192E-05	1,25820474	1,06308262	0,28774456
VC0406	-1,1177195	-6,4604621	-1,1520172	-6,304E-06	-1,1520172	-1,0623258	0,28808785
VC1481	1,2881433	55,1932327	1,26205581	5,3858E-05	1,26205581	1,06214538	0,28816976
ksgA	1,2120309	14,2792907	1,17449801	1,3934E-05	1,17449801	1,06143879	0,28849058
VCA0551	1,2224081	18,3315418	1,20261009	1,7888E-05	1,20261009	1,06010825	0,28909542
VCA1071	-3,0842521	-53,332773	-3,1631736	-5,204E-05	-3,1631736	-1,0592626	0,28948027
VCA1022	1,38852897	2,64249951	1,3310716	2,5786E-06	1,3310716	1,05923286	0,28949379
VC1661	1,22298443	12,139765	1,22283276	1,1846E-05	1,22283276	1,05832277	0,28990838
VC1063	1,4652687	13,247062	1,42932554	1,2927E-05	1,42932554	1,05818558	0,28997091
VC0559	-1,1136175	-5,3381907	-1,1501268	-5,209E-06	-1,1501268	-1,0574574	0,29030294
VC0975	1,17882806	11,9512609	1,13907113	1,1662E-05	1,13907113	1,05711231	0,29046038
rpsH	1,53754338	400,091863	1,39952742	0,00039041	1,39952742	1,05678261	0,29061088
VC2363	1,71096654	20,7651656	1,69226963	2,0263E-05	1,69226963	1,05650049	0,2907397
VC0295	1,38597099	36,2684997	1,39895387	3,5391E-05	1,39895387	1,05639582	0,29078748
VCA0125	-1,1213732	-15,327827	-1,1053996	-1,496E-05	-1,1053996	-1,0549884	0,29143066
VC1374	1,61354216	3,57864895	1,49682056	3,4921E-06	1,49682056	1,05479191	0,29152056
tRNA-Thr-2	-1,1861389	-2,8380461	-1,3002995	-2,769E-06	-1,3002995	-1,0542844	0,29175275
VCA0636	-1,2568084	-10,841905	-1,2763043	-1,058E-05	-1,2763043	-1,0539057	0,29192612
VC1721	1,18861125	5,85558678	1,13476009	5,7139E-06	1,13476009	1,05308107	0,29230388
VC0820	1,80365381	6,22340867	1,69816086	6,0729E-06	1,69816086	1,05285882	0,29240576
VC1539a	-1,3478366	-21,903435	-1,4937962	-2,137E-05	-1,4937962	-1,050546	0,29346719
galM	-1,6432854	-155,75155	-1,9100844	-0,000152	-1,9100844	-1,0484325	0,29443945
VC0828	-1,3077858	-12,386839	-1,3186261	-1,209E-05	-1,3186261	-1,0470901	0,29505806
VCA0575	1,14714295	7,72032058	1,13727121	7,5336E-06	1,13727121	1,04705171	0,29507577
VC2000	-1,9478899	-1560,8708	-1,6185649	-0,0015231	-1,6185649	-1,0467763	0,29520283
VCA0336	1,37816166	2,75671266	1,30496522	2,69E-06	1,30496522	1,04604794	0,29553894
VC1839	1,12160534	7,83451238	1,12104191	7,645E-06	1,12104191	1,04505195	0,295999
VC2150	1,33734442	3,24603035	1,2522267	3,1675E-06	1,2522267	1,04431016	0,29634195
acpP	1,0618922	570,345142	1,26338121	0,00055655	1,26338121	1,04320033	0,29685556
VC1267	1,85696653	5,61541667	1,70035059	5,4796E-06	1,70035059	1,04261378	0,29712724
gltA	2,71058477	136,924114	2,42699184	0,00013361	2,42699184	1,04202678	0,29739934
VC1790	-1,370915	-20,267618	-1,3424371	-1,978E-05	-1,3424371	-1,0416622	0,29756837
VC2568	1,3396977	35,9980318	1,3486465	3,5127E-05	1,3486465	1,04110655	0,29782617
VC1866	-1,4951043	-751,24259	-1,4446331	-0,0007331	-1,4446331	-1,0397641	0,29844957
VC1354	-1,2108047	-2,9940303	-1,2733144	-2,922E-06	-1,2733144	-1,0391851	0,2987187
VC0439	-1,2483208	-8,9968118	-1,3252352	-8,779E-06	-1,3252352	-1,0391031	0,29875685
rimM	1,49320248	183,688256	1,24868877	0,00017924	1,24868877	1,03745872	0,29952217
VC0246	1,43016821	129,887346	1,35269726	0,00012675	1,35269726	1,03688425	0,29978985
VCA0655	-1,2771535	-8,1425571	-1,4957102	-7,946E-06	-1,4957102	-1,0367703	0,299843
cysB	1,30878471	25,7648097	1,26120843	2,5142E-05	1,26120843	1,03498689	0,30067509
VCA0087	2,41484869	12,9364594	2,36698006	1,2624E-05	2,36698006	1,03494937	0,30069264

radC	-1,4375863	-6,5448897	-1,5451141	-6,387E-06	-1,5451141	-1,0343166	0,30098824
VC2171	-1,2536445	-3,4951544	-1,3252198	-3,411E-06	-1,3252198	-1,0335379	0,30135228
VCA0585	1,33221157	3,24303152	1,24662751	3,1646E-06	1,24662751	1,03346222	0,30138768
VCA0584	1,32181257	3,86688929	1,20254089	3,7733E-06	1,20254089	1,03285737	0,30167069
VC2210	1,68057881	5,41216763	1,60202122	5,2812E-06	1,60202122	1,03283102	0,30168304
VC1064	1,13471875	29,507271	1,12894849	2,8793E-05	1,12894849	1,03187829	0,30212918
VC0880	2,11264313	2,2730487	2,00204718	2,2181E-06	2,00204718	1,03149999	0,30230647
VC1236	1,4070317	2,62756632	1,31136225	2,564E-06	1,31136225	1,03047757	0,30278592
VC2641	1,38693139	2,72515171	1,29851858	2,6592E-06	1,29851858	1,03043335	0,30280666
VC2224	-1,1657772	-2,9322494	-1,2744807	-2,861E-06	-1,2744807	-1,030335	0,30285283
VC0906	1,49787599	3,06484134	1,37698449	2,9907E-06	1,37698449	1,02988577	0,30306368
VC0959	1,23076009	57,7124938	1,19244557	5,6316E-05	1,19244557	1,02947083	0,30325852
prpB	1,67223394	2,22427214	1,43156347	2,1705E-06	1,43156347	1,02906934	0,30344713
VC0386	2,24453185	28,2980287	2,25743408	2,7613E-05	2,25743408	1,02754977	0,30416173
glmM	1,12771239	12,8912933	1,1275038	1,2579E-05	1,1275038	1,02384114	0,30591036
VCA0401	1,29890316	11,2732047	1,27391283	1,1E-05	1,27391283	1,02311166	0,30625511
VCA1044	-1,2750471	-33,55247	-1,3024154	-3,274E-05	-1,3024154	-1,0218187	0,30686678
fadI	1,56507746	2,86667326	1,392629	2,7973E-06	1,392629	1,02178935	0,30688066
VC2366	1,10760773	12,9210107	1,10813066	1,2608E-05	1,10813066	1,02098398	0,30726208
VC1362	-1,2674752	-2,1134957	-1,3934044	-2,062E-06	-1,3934044	-1,020879	0,3073118
glyS	1,27835099	10,4392272	1,25057034	1,0187E-05	1,25057034	1,02083919	0,30733067
VC1799	1,63541116	1,79049497	1,48103487	1,7472E-06	1,48103487	1,02051652	0,3074836
VC1260	1,41015477	2,82674944	1,29571556	2,7584E-06	1,29571556	1,01891512	0,30824331
VC1966	-1,1083666	-4,3755701	-1,1707325	-4,27E-06	-1,1707325	-1,0161055	0,30957921
VCA0922	-1,109971	-4,1339699	-1,1815608	-4,034E-06	-1,1815608	-1,0159609	0,30964804
tarA	1,54823761	3,23736227	1,42890286	3,159E-06	1,42890286	1,0134102	0,31086431
VCA0517	-2,3335049	-190,51346	-2,5813756	-0,0001859	-2,5813756	-1,0131827	0,31097296
VCA0057	1,30235904	3,16769924	1,24141854	3,0911E-06	1,24141854	1,01171823	0,31167284
VC1521	-1,1257741	-5,0152038	-1,2154502	-4,894E-06	-1,2154502	-1,0117103	0,31167663
VC0157	1,40361473	2,73132567	1,285234	2,6653E-06	1,285234	1,01131171	0,31186731
tRNA-Gly-7	-1,4163864	-7,6074654	-1,4196355	-7,423E-06	-1,4196355	-1,0112108	0,31191559
VC0040	1,20105997	12,1922703	1,19378563	1,1897E-05	1,19378563	1,01045127	0,31227919
VC1470	1,16434077	5,69967661	1,12692301	5,5618E-06	1,12692301	1,01016003	0,31241866
VC2369	-1,0941122	-4,7987884	-1,1518366	-4,683E-06	-1,1518366	-1,0078996	0,31350269
VCA0241	-1,6812725	-2,5555007	-1,8933491	-2,494E-06	-1,8933491	-1,0069516	0,31395808
ruvA	1,34926527	4,25694983	1,29973852	4,154E-06	1,29973852	1,00598293	0,31442383
VC1599	-1,1092735	-5,7927026	-1,2056218	-5,653E-06	-1,2056218	-1,0042754	0,31524591
VCA0465	1,21445196	6,85217834	1,15446419	6,6864E-06	1,15446419	1,00365137	0,31554673
VCA0576	1,91873244	8,05262376	1,82846607	7,8578E-06	1,82846607	1,00330067	0,31571589
VC1885	1,44075682	2,45003131	1,31733988	2,3908E-06	1,31733988	1,0032654	0,31573286
VC2139	-1,139571	-22,043977	-1,1400826	-2,151E-05	-1,1400826	-1,0030174	0,31585251
VC2177	-1,1141698	-5,1056585	-1,1796956	-4,982E-06	-1,1796956	-1,0024561	0,31612342
VC0811	1,41689733	39,4538967	1,42701454	3,8499E-05	1,42701454	1,00161933	0,31652755
VC2726	-1,1212776	-4,1674058	-1,1744298	-4,067E-06	-1,1744298	-1,0014671	0,31660108
moaC	1,37204066	3,44969314	1,31760665	3,3662E-06	1,31760665	1,00134533	0,31665993

metN	1,5336262	2,51552404	1,38109206	2,4547E-06	1,38109206	1,00120309	0,31672867
VC0533	1,37477637	18,7644582	1,3018961	1,8311E-05	1,3018961	1,00093646	0,3168576
23Se	1,03487991	12615,6156	1,09423534	0,01231043	1,09423534	0,99986855	0,3173742
VC0814	1,39892454	11,9501847	1,34864667	1,1661E-05	1,34864667	0,99895559	0,31781625
cadB	79,8664375	105,842743	77,9336526	0,00010328	77,9336526	0,99860529	0,31798602
VC0201	1,74895209	2,57157948	1,63053579	2,5094E-06	1,63053579	0,99825031	0,31815805
atpC	-1,0016612	-102,88813	-1,1626375	-0,0001004	-1,1626375	-0,9982377	0,31816415
VC2381	-1,1751453	-3,7198169	-1,2417597	-3,63E-06	-1,2417597	-0,9981348	0,31821403
VCA0966	1,38687104	6,68872324	1,27730106	6,5269E-06	1,27730106	0,99772343	0,31841354
VC2161	-1,2229129	-13,617937	-1,2625433	-1,329E-05	-1,2625433	-0,9963596	0,31907553
VC1138	1,58994541	8,39220769	1,57709696	8,1892E-06	1,57709696	0,9960893	0,31920682
VC0281	65,61349	69,1291103	61,9550509	6,7457E-05	61,9550509	0,99538132	0,31955092
VC0493	1,513257	29,718619	1,46574692	2,9E-05	1,46574692	0,99537286	0,319555
16Se	1,0844878	9839,64627	1,1830185	0,00960161	1,1830185	0,9953495	0,31956636
mukB	1,20401841	4,5405581	1,15587313	4,4307E-06	1,15587313	0,99242103	0,32099222
VCA0010	-1,3545699	-5,6562512	-1,3790651	-5,519E-06	-1,3790651	-0,9923049	0,32104884
VC0162	-1,6893232	-34,984839	-1,6220275	-3,414E-05	-1,6220275	-0,9912427	0,32156716
VCA0692	1,7117762	10,0695924	1,603459	9,826E-06	1,603459	0,98959858	0,32237041
VCA0792	-1,3501515	-19,511777	-1,3240405	-1,904E-05	-1,3240405	-0,9878963	0,32320349
VC1067	-1,1271894	-5,7613052	-1,1749591	-5,622E-06	-1,1749591	-0,9867223	0,32377885
VC1098	-1,4108235	-75,560874	-1,3367932	-7,373E-05	-1,3367932	-0,9861193	0,32407463
VCA0807	-1,233564	-10,223833	-1,3682042	-9,977E-06	-1,3682042	-0,983515	0,32535406
VC0637	1,11579972	23,4059468	1,10050709	2,284E-05	1,10050709	0,98311682	0,32555001
VCA0368	1,29735351	2,97511464	1,25797243	2,9031E-06	1,25797243	0,9829296	0,32564213
VCA0569	-1,1658735	-14,717862	-1,180488	-1,436E-05	-1,180488	-0,9828557	0,32567849
VC2725	-1,1044422	-5,3807909	-1,1406413	-5,251E-06	-1,1406413	-0,9825225	0,32584254
VC0057	1,24861644	4,16543977	1,16641392	4,0647E-06	1,16641392	0,9797628	0,32720326
VC2371	-2,1958345	-39,038209	-1,944854	-3,809E-05	-1,944854	-0,979496	0,32733498
VC1163	1,50507882	1,91687376	1,3991248	1,8705E-06	1,3991248	0,97810747	0,32802121
VC0351	1,21445967	4,69276483	1,1765933	4,5792E-06	1,1765933	0,97785703	0,32814508
VC2700	-6,4095216	-23,110127	-6,783909	-2,255E-05	-6,783909	-0,9778249	0,32816101
VCA0491	1,34845922	5,67299826	1,29425186	5,5358E-06	1,29425186	0,97732214	0,32840973
VC1512	-1,6570595	-20,497979	-1,7113671	-2E-05	-1,7113671	-0,9765364	0,32879879
VC0288	-1,581715	-5,4907433	-1,9119742	-5,358E-06	-1,9119742	-0,9759419	0,32909331
VC1487	1,27824956	6,72326085	1,2522701	6,5606E-06	1,2522701	0,97520976	0,32945628
VC1413	1,33221034	3,63461046	1,22355528	3,5467E-06	1,22355528	0,97414568	0,32998426
VC2184	-1,2884002	-18,453812	-1,2930064	-1,801E-05	-1,2930064	-0,9728922	0,33060693
VC0925	1,36650851	2,47030467	1,29274443	2,4105E-06	1,29274443	0,97275723	0,33067401
VC1183	1,90121634	6,85885598	1,66988391	6,6929E-06	1,66988391	0,97101827	0,33153924
VC1216	-1,2997712	-10,911541	-1,3232254	-1,065E-05	-1,3232254	-0,9704451	0,33182474
VC2392	-1,0582406	-5,4486156	-1,1221948	-5,317E-06	-1,1221948	-0,9701693	0,33196215
VC0353	1,17732539	5,1307705	1,1300893	5,0067E-06	1,1300893	0,96957796	0,33225693
VC1800	1,8793305	1,22081449	1,6906332	1,1913E-06	1,6906332	0,96957739	0,33225722
tRNA-Leu-7	-1,3597859	-12,069528	-1,3891457	-1,178E-05	-1,3891457	-0,9690719	0,33250939
VCA0143	-1,1764311	-2,6596072	-1,2665105	-2,595E-06	-1,2665105	-0,9686126	0,33273853

VCA0521	-1,3732296	-13,199908	-1,3967462	-1,288E-05	-1,3967462	-0,9674543	0,33331705
VC1051	1,2605785	18,1507266	1,23857071	1,7712E-05	1,23857071	0,96700074	0,33354371
VC2041	-1,1104878	-7,8576092	-1,1020987	-7,668E-06	-1,1020987	-0,9668494	0,33361936
VC0831	1,6336747	4,55128933	1,55163207	4,4412E-06	1,55163207	0,96655717	0,3337655
VC0839	1,85416494	7,17626526	1,78613085	7,0027E-06	1,78613085	0,96576211	0,3341633
valS	1,26514079	7,81157064	1,21342488	7,6226E-06	1,21342488	0,96481893	0,33463557
VCA0294	-1,1801762	-8,2808768	-1,2700168	-8,081E-06	-1,2700168	-0,9647386	0,33467579
VCA0621	1,58539947	10,0286187	1,4731735	9,786E-06	1,4731735	0,96281821	0,3356388
VC2607	1,54794377	7,43893855	1,38981437	7,259E-06	1,38981437	0,96124562	0,33642875
VC2643	1,74213817	1,38610521	1,56912105	1,3526E-06	1,56912105	0,95977506	0,33716847
VCA0920	-1,0803748	-4,9954048	-1,1307435	-4,875E-06	-1,1307435	-0,9589568	0,33758056
VC2149	-1,1418463	-41,166415	-1,1744584	-4,017E-05	-1,1744584	-0,9588323	0,33764327
lolA	1,19768769	7,41801071	1,16132321	7,2386E-06	1,16132321	0,9581941	0,33796494
VCA1043	1,27663897	44,1720394	1,29261566	4,3103E-05	1,29261566	0,95779817	0,3381646
VC0461	1,21116758	4,53736518	1,14451675	4,4276E-06	1,14451675	0,95777618	0,33817567
VC2549	1,32297032	4,2010046	1,21616309	4,0994E-06	1,21616309	0,95618788	0,33897736
VC1262	1,50629254	69,3387995	1,4151381	6,7661E-05	1,4151381	0,95433054	0,33991641
VCA0498	1,17913188	28,8497747	1,19781463	2,8152E-05	1,19781463	0,95413394	0,34001591
VC0117	-1,1768664	-6,6477647	-1,2649275	-6,487E-06	-1,2649275	-0,9534482	0,34036305
VC0638	1,14095891	5,61924124	1,1139806	5,4833E-06	1,1139806	0,9534008	0,34038707
VC1256	1,55620223	24,9912467	1,54052786	2,4387E-05	1,54052786	0,95334519	0,34041526
VC2100	-2,0960884	-11,856781	-2,0473411	-1,157E-05	-2,0473411	-0,9525408	0,34082284
VCA0520	-1,4969378	-20,538666	-1,5000606	-2,004E-05	-1,5000606	-0,9524399	0,34087397
VCA0866	2,6417968	0,93638165	2,10390483	9,1373E-07	2,10390483	0,95228051	0,34095478
VC2036	1,49231221	30,868724	1,43366816	3,0122E-05	1,43366816	0,95220814	0,34099151
VC0610	1,26621771	3,60248758	1,21168037	3,5153E-06	1,21168037	0,95107766	0,34156499
VCA0460	1,34621322	2,26896027	1,30649548	2,2141E-06	1,30649548	0,95106816	0,34156981
VC0139	-1,1300229	-6,3308803	-1,2132728	-6,178E-06	-1,2132728	-0,9507669	0,34172276
VCA0309	1,16696804	16,5170955	1,19615879	1,6118E-05	1,19615879	0,94930961	0,34246322
VC1189	-1,5384652	-88,850309	-1,5825535	-8,67E-05	-1,5825535	-0,9488529	0,34269548
VCA0044	1,24664314	3,5832227	1,18262759	3,4965E-06	1,18262759	0,94840857	0,34292154
VCA0540	-2,1850432	-358,76025	-2,1029397	-0,0003501	-2,1029397	-0,9481831	0,34303629
VC0952	1,167887	11,2972713	1,14714836	1,1024E-05	1,14714836	0,9468526	0,34371393
VC0275	1,49803062	3,00461295	1,40396928	2,9319E-06	1,40396928	0,94646528	0,34391137
VCA0997	1,34314851	2,44048468	1,27865832	2,3814E-06	1,27865832	0,94623111	0,34403076
VCA0817	1,34492134	4,62956531	1,23706732	4,5176E-06	1,23706732	0,94589938	0,34419996
VC2044	1,26085094	29,7917035	1,27351715	2,9071E-05	1,27351715	0,94587273	0,34421356
VCA0679	2,10231012	78,6822202	2,17184018	7,6779E-05	2,17184018	0,94509283	0,34461153
rpmF	1,27326814	39,8851147	1,27123003	3,892E-05	1,27123003	0,944937	0,34469111
VC2295	1,12159501	33,2845893	1,09714456	3,2479E-05	1,09714456	0,94430352	0,34501463
VC1639	1,33892023	3,24158807	1,28682401	3,1632E-06	1,28682401	0,94306651	0,34564693
VCA0168	1,1890877	6,54362692	1,149193	6,3853E-06	1,149193	0,94232658	0,3460255
VCA0940	-1,2474421	-18,622679	-1,4044164	-1,817E-05	-1,4044164	-0,9419661	0,34621005
VCA1035	1,99186677	6,58893953	1,89064563	6,4295E-06	1,89064563	0,94175203	0,34631967
VC0202	1,87758319	2,5668851	1,76025402	2,5048E-06	1,76025402	0,94164964	0,3463721

VC2361	1,41022548	567,626473	1,45943854	0,00055389	1,45943854	0,94109945	0,34665395
VC1568	1,41863592	2,09046894	1,32878199	2,0399E-06	1,32878199	0,94096546	0,3467226
VC2261	1,20756075	10,0146615	1,18718575	9,7724E-06	1,18718575	0,93850583	0,34798457
fliF	-1,0689297	-7,1174141	-1,1033047	-6,945E-06	-1,1033047	-0,9381876	0,34814805
VCA0586	-1,1951509	-2,6642085	-1,2549064	-2,6E-06	-1,2549064	-0,9363108	0,34911323
VC2769	1,22947122	76,3845321	1,18346635	7,4537E-05	1,18346635	0,93530019	0,34963369
VC1200	1,1981783	4,96268281	1,12484351	4,8426E-06	1,12484351	0,93529232	0,34963771
fumC	1,56111182	5,32445058	1,48072387	5,1956E-06	1,48072387	0,93507585	0,34974925
16Sh	1,21288012	1570,29384	1,62979901	0,00153231	1,62979901	0,93501636	0,34977994
VC1181	1,26640791	3,59572901	1,17853749	3,5087E-06	1,17853749	0,93451867	0,35003645
VCA1102	-1,2238673	-2,0284837	-1,3349625	-1,979E-06	-1,3349625	-0,9343424	0,35012736
VC2370	-1,6397432	-13,004777	-1,5820313	-1,269E-05	-1,5820313	-0,9343071	0,35014555
flgD	-1,142471	-34,394521	-1,1598407	-3,356E-05	-1,1598407	-0,9339249	0,35034271
VC0249	1,39977229	337,11208	1,34504263	0,00032896	1,34504263	0,93303278	0,35080308
VC0385	2,6121295	16,6264574	2,66330487	1,6224E-05	2,66330487	0,93282952	0,35090806
VC0027	1,75862389	16,2440824	1,74654244	1,5851E-05	1,74654244	0,9326826	0,35098394
VC1201	-1,2121385	-28,733886	-1,1712714	-2,804E-05	-1,1712714	-0,93258	0,35103695
nusG	1,18783257	36,3279917	1,19952785	3,5449E-05	1,19952785	0,93239074	0,35113468
VC1948	2,6228052	0,66174064	2,5575043	6,4573E-07	2,5575043	0,93228057	0,35119159
VC0789	7,54232871	328,142614	7,7996639	0,0003202	7,7996639	0,93170856	0,35148723
VC2241	-1,3997387	-69,984235	-1,4514572	-6,829E-05	-1,4514572	-0,9315507	0,35156879
VC1904	1,32868548	66,0794375	1,29526212	6,4481E-05	1,29526212	0,93012398	0,35230696
VC1126	1,28904453	15,4052717	1,29736031	1,5033E-05	1,29736031	0,93009006	0,35232451
VCA0721	1,45968442	2,66089172	1,36455018	2,5965E-06	1,36455018	0,92730252	0,35376953
VC0175	1,30594284	43,3025352	1,32730676	4,2255E-05	1,32730676	0,92600436	0,35444376
VC1384	-1,1607887	-152,38011	-1,2825112	-0,0001487	-1,2825112	-0,9257869	0,35455678
VCA0467	1,47319985	7,50789225	1,47970941	7,3263E-06	1,47970941	0,92573903	0,35458168
VC1420	1,34178632	2,55520685	1,25147433	2,4934E-06	1,25147433	0,92531466	0,35480229
rfaD	1,16409789	16,1722493	1,17782333	1,5781E-05	1,17782333	0,92523861	0,35484184
VCA0524	-1,2219361	-2,2413319	-1,2909097	-2,187E-06	-1,2909097	-0,9240399	0,35546556
VCA1037	1,39752978	3,37038534	1,26055143	3,2889E-06	1,26055143	0,92320678	0,35589949
VCA0055	1,47859878	2,01053357	1,32880097	1,9619E-06	1,32880097	0,92282253	0,35609973
tRNA-Gly-5	-1,1879442	-7,1573255	-1,2046356	-6,984E-06	-1,2046356	-0,9213923	0,35684571
VC0727	1,28967439	5,57608102	1,19986457	5,4412E-06	1,19986457	0,92038727	0,35737046
VC0516	1,47626351	42,8307047	1,56318296	4,1795E-05	1,56318296	0,91971282	0,3577229
VC0908	1,20619311	8,08410988	1,16592107	7,8885E-06	1,16592107	0,91970923	0,35772478
VCA0366	1,27299432	3,21999533	1,19163848	3,1421E-06	1,19163848	0,91907103	0,35805845
VC0168	1,51964336	96,063524	1,40409681	9,374E-05	1,40409681	0,91777068	0,35873899
VC2766	1,21068651	63,2066922	1,14327239	6,1678E-05	1,14327239	0,91747968	0,3588914
VCA0560	-1,0732587	-4,4553376	-1,13432	-4,348E-06	-1,13432	-0,9171683	0,3590545
VC2694	-1,0550291	-4,0412476	-1,1490024	-3,943E-06	-1,1490024	-0,9168595	0,35921629
VCA0248	-1,2125742	-8,2796729	-1,3202215	-8,079E-06	-1,3202215	-0,9167465	0,35927552
flgK	-1,1024572	-12,457808	-1,1409283	-1,216E-05	-1,1409283	-0,9166635	0,35931905
VC1054	1,18781947	5,36666176	1,11418132	5,2368E-06	1,11418132	0,91650646	0,35940136
VC0343	1,16509221	5,23821776	1,11272348	5,1115E-06	1,11272348	0,91569049	0,35982929

VC1575	1,42501058	31,8005966	1,37468544	3,1031E-05	1,37468544	0,91428404	0,36056765
VC2518	1,23395286	9,15223819	1,21537615	8,9308E-06	1,21537615	0,91364457	0,36090369
VCA0643	2,04548893	0,88599724	1,91542998	8,6456E-07	1,91542998	0,91356278	0,36094667
flhF	-1,0745738	-7,8346249	-1,1306206	-7,645E-06	-1,1306206	-0,9132234	0,3611251
maf (NC_002505 444542..445200)	-1,0997256	-13,100448	-1,1163134	-1,278E-05	-1,1163134	-0,9127755	0,36136069
VC0284	1,61267637	1,38300898	1,50157856	1,3496E-06	1,50157856	0,91208706	0,36172293
VC1205	2,98079391	3,93895421	2,42620246	3,8437E-06	2,42620246	0,91049433	0,36256193
VC0523	-1,0678981	-5,4038146	-1,1156743	-5,273E-06	-1,1156743	-0,9086445	0,36353785
VCA0536	-1,3633991	-62,624234	-1,3613039	-6,111E-05	-1,3613039	-0,9069341	0,36444167
VCA0183	-1,1469479	-2,5795759	-1,2378439	-2,517E-06	-1,2378439	-0,906919	0,36444968
VC0161	1,39689374	4,16418429	1,29997667	4,0634E-06	1,29997667	0,90671243	0,36455894
VCA0900	-1,1651208	-2,6380948	-1,2317894	-2,574E-06	-1,2317894	-0,9066272	0,364604
rplE	1,35491733	89,5222286	1,12800705	8,7357E-05	1,12800705	0,90648048	0,36468166
sdhC	2,68753349	69,0894933	2,44254972	6,7418E-05	2,44254972	0,90521442	0,36535187
trmD	1,56246861	126,367566	1,23296147	0,00012331	1,23296147	0,90508511	0,36542034
aroK	1,28961033	22,9016825	1,23824664	2,2348E-05	1,23824664	0,90492055	0,36550752
VC0715	1,38131822	25,2882411	1,39406211	2,4676E-05	1,39406211	0,90482589	0,36555769
VC1417	1,2980814	2,83819134	1,21655439	2,7695E-06	1,21655439	0,90312758	0,36645822
VC1103	1,43126076	3,72807221	1,28445063	3,6379E-06	1,28445063	0,9007007	0,36774752
VCA0843	1,58765112	9,7616711	1,4352512	9,5255E-06	1,4352512	0,89965944	0,36830158
sbcB	1,39495361	2,70091943	1,3014648	2,6356E-06	1,3014648	0,89952656	0,36837229
VC0470	1,37576859	2,14857869	1,28688673	2,0966E-06	1,28688673	0,89923045	0,36852996
VC1854	-1,4767564	-765,94822	-1,3165796	-0,0007474	-1,3165796	-0,8977741	0,36930607
VC0035	1,25955846	3,41547153	1,17029241	3,3328E-06	1,17029241	0,89666274	0,36989894
VC0746	-1,2703567	-9,1202934	-1,2682371	-8,9E-06	-1,2682371	-0,8961096	0,3701943
VC2296	1,20129303	5,09082629	1,12796014	4,9677E-06	1,12796014	0,8956736	0,37042715
VCA0101	1,22202784	4,794163	1,1270116	4,6782E-06	1,1270116	0,89489069	0,37084556
VC1651	1,28989141	2,62055131	1,22678909	2,5572E-06	1,22678909	0,89481087	0,37088823
tRNA-Gln-3	-1,4570069	-5,5826655	-1,4996377	-5,448E-06	-1,4996377	-0,8945196	0,37104402
VC1860	1,20653863	25,3583356	1,2276588	2,4745E-05	1,2276588	0,89406003	0,37128983
VC1393	-1,0963129	-3,556506	-1,1617524	-3,47E-06	-1,1617524	-0,8935108	0,37158374
VC1437	-1,2313913	-12,411947	-1,2667929	-1,211E-05	-1,2667929	-0,8933156	0,37168819
VC1979	1,40157186	2,17131727	1,27836444	2,1188E-06	1,27836444	0,89211217	0,37233285
VCA0278	1,613779	43,8446777	1,54925102	4,2784E-05	1,54925102	0,89191379	0,37243921
VC1798	1,33745034	2,22721098	1,27000033	2,1733E-06	1,27000033	0,89148186	0,37267076
tnaA	2,07919848	3,48203865	1,76159069	3,3978E-06	1,76159069	0,89129597	0,37277046
VCA0315	-1,3374597	-3,7720668	-1,4347419	-3,681E-06	-1,4347419	-0,891294	0,37277154
VCA1104	-1,2783844	-7,1315056	-1,3772439	-6,959E-06	-1,3772439	-0,8880309	0,37452416
ushA	-1,2544423	-87,920698	-1,3686441	-8,579E-05	-1,3686441	-0,8878765	0,37460727
VC0225	1,27879384	2,9087664	1,19804887	2,8384E-06	1,19804887	0,88671898	0,37523025
VC1452	-1,1191788	-6,9983	-1,1190709	-6,829E-06	-1,1190709	-0,8863575	0,37542497
lysS	1,2680681	17,5009193	1,24739969	1,7078E-05	1,24739969	0,8861558	0,37553362
VCA0674	1,51766387	18,9201609	1,49910406	1,8462E-05	1,49910406	0,88523622	0,37602928
VC1071	1,54348884	1,44303433	1,51613033	1,4081E-06	1,51613033	0,88412213	0,37663032
fis	1,38858858	38,6391985	1,42595849	3,7704E-05	1,42595849	0,88362167	0,37690055

VCA0218	1,61782674	2,62899653	1,39072165	2,5654E-06	1,39072165	0,88291874	0,37728022
VC2653	1,29060645	56,1191397	1,27946418	5,4762E-05	1,27946418	0,88246795	0,37752387
VC0223	-1,0869525	-3,5904372	-1,1556373	-3,504E-06	-1,1556373	-0,8818782	0,37784273
VC0541	1,6338817	12,8758916	1,64148868	1,2564E-05	1,64148868	0,88157545	0,3780065
tmk	1,23991138	2,97295543	1,19043941	2,901E-06	1,19043941	0,88058498	0,37854253
ulaD	-1,527143	-2,5870343	-1,6754377	-2,524E-06	-1,6754377	-0,8797647	0,37898683
VC1716	1,2849667	5,02071373	1,20560999	4,8993E-06	1,20560999	0,87966587	0,37904038
VC0567	-1,0137422	-30,227782	-1,0821423	-2,95E-05	-1,0821423	-0,87932	0,37922782
VC0849	1,23966161	10,2211481	1,22047393	9,9739E-06	1,22047393	0,87926329	0,37925859
VCA0565	-1,1356944	-9,1170379	-1,1311384	-8,896E-06	-1,1311384	-0,8788503	0,37948247
VCA0582	1,26206422	4,79035603	1,2072661	4,6745E-06	1,2072661	0,87811355	0,37988214
VC1510	-1,6524177	-38,523537	-1,6878461	-3,759E-05	-1,6878461	-0,8776689	0,38012344
VC0248	1,42408353	163,294777	1,3046684	0,00015934	1,3046684	0,87681629	0,38058646
VC0451	-1,0803537	-5,3521166	-1,100566	-5,223E-06	-1,100566	-0,8767841	0,38060397
VC1672	-1,1862899	-2,1243821	-1,2739564	-2,073E-06	-1,2739564	-0,8762509	0,38089369
VC1285	1,25993078	3,29748221	1,16735917	3,2177E-06	1,16735917	0,87400864	0,38211358
VC1962	1,24945737	5,93140851	1,17762286	5,7879E-06	1,17762286	0,87203951	0,38318686
VCA0986	-1,3574836	-10,993738	-1,4273817	-1,073E-05	-1,4273817	-0,8716985	0,38337294
gltD (NC_002505 2542113..2543569)	1,56539916	5,47322849	1,5155686	5,3408E-06	1,5155686	0,86862049	0,3850548
VC1278	-1,1043458	-3,2145045	-1,1707328	-3,137E-06	-1,1707328	-0,8684214	0,38516373
rpoZ	1,13236027	19,4463444	1,14292693	1,8976E-05	1,14292693	0,86820907	0,38527993
proA	1,26613388	5,11295995	1,20687379	4,9893E-06	1,20687379	0,86606439	0,3864549
flhB	-1,1630039	-5,926856	-1,2373404	-5,783E-06	-1,2373404	-0,8651563	0,38695303
VC0270	1,13236293	14,7891328	1,13017695	1,4431E-05	1,13017695	0,86425232	0,38744934
VC2402	-1,126369	-3,3421351	-1,1830556	-3,261E-06	-1,1830556	-0,864062	0,38755386
VC0256	-1,3558672	-18,611719	-1,3102625	-1,816E-05	-1,3102625	-0,8634353	0,38789823
dapF	1,12012866	6,39708169	1,09419714	6,2423E-06	1,09419714	0,86316403	0,38804732
VCA0629	-1,2696328	-1,5468991	-1,3823682	-1,509E-06	-1,3823682	-0,8630364	0,38811751
VC2107	1,2761001	9,69401794	1,2504577	9,4595E-06	1,2504577	0,86298892	0,38814361
VC2625	1,19344684	4,23577894	1,12445161	4,1333E-06	1,12445161	0,86280329	0,38824566
grxA	1,2240142	34,8508644	1,23551446	3,4008E-05	1,23551446	0,86225938	0,38854484
panC	1,20098896	12,2375134	1,18347823	1,1941E-05	1,18347823	0,86192635	0,38872809
VC1308	1,2196808	3,61571175	1,14696091	3,5282E-06	1,14696091	0,86169188	0,38885713
VC0882	1,25860479	8,29554199	1,1960045	8,0949E-06	1,1960045	0,86058533	0,38946652
VC2706	1,65668265	12,0750592	1,61904696	1,1783E-05	1,61904696	0,86020195	0,38967779
tRNA-Met-4	-1,3164737	-8,649703	-1,3380839	-8,44E-06	-1,3380839	-0,8596863	0,38996202
VCA0112	1,60019419	1,37564707	1,43606297	1,3424E-06	1,43606297	0,85949891	0,39006536
VCA0867	1,85846808	182,193068	1,8777929	0,00017779	1,8777929	0,85867514	0,39051981
rplI	1,29399794	77,4674666	1,26550621	7,5593E-05	1,26550621	0,85832259	0,39071441
VC2558	1,86452144	5,47686251	1,80012086	5,3444E-06	1,80012086	0,85807005	0,39085385
VCA0743	-1,3513328	-21,476542	-1,3631355	-2,096E-05	-1,3631355	-0,857155	0,39135928
VC0667	1,25366129	11,4829174	1,21111654	1,1205E-05	1,21111654	0,85694848	0,39147341
VC1150	1,58003054	20,154279	1,46689539	1,9667E-05	1,46689539	0,85620412	0,39188494
VC0255	1,20034326	62,2072307	1,28069191	6,0702E-05	1,28069191	0,85598808	0,39200441
VC0203	1,56357537	1,55879976	1,42392876	1,5211E-06	1,42392876	0,85594538	0,39202802

tolB	1,46520579	116,525982	1,31237132	0,00011371	1,31237132	0,85530185	0,39238409
VC1474	1,45470805	1,64196494	1,34876602	1,6022E-06	1,34876602	0,85524584	0,3924151
VC2022	1,24751661	18,7234515	1,23390994	1,8271E-05	1,23390994	0,85441399	0,3928757
pheT	1,28008583	7,20703247	1,2103147	7,0327E-06	1,2103147	0,85301855	0,39364904
VC1889	1,29453675	2,39356758	1,22498845	2,3357E-06	1,22498845	0,8521229	0,39414592
aroE	1,23900321	6,41585718	1,18638932	6,2606E-06	1,18638932	0,85167594	0,39439402
VC1086	-1,2776124	-6,135244	-1,3379712	-5,987E-06	-1,3379712	-0,851531	0,39447449
VCA0195	-1,2194168	-9,6915877	-1,3563604	-9,457E-06	-1,3563604	-0,8514408	0,3945246
VCA0518	-1,8683808	-277,57304	-2,252276	-0,0002709	-2,252276	-0,8508783	0,394837
VC0431	-1,0920828	-10,682885	-1,0546082	-1,042E-05	-1,0546082	-0,8503729	0,39511784
fliI	-1,0715953	-3,77267	-1,1364861	-3,681E-06	-1,1364861	-0,8503262	0,3951438
VC1617	2,09432741	14,3903892	1,84790971	1,4042E-05	1,84790971	0,85030662	0,3951547
VCA0765	1,29587592	7,23770241	1,20446687	7,0626E-06	1,20446687	0,85029879	0,39515904
VC2119	-1,0759061	-5,513304	-1,1301649	-5,38E-06	-1,1301649	-0,8500782	0,39528162
VC0827	-1,2511434	-274,95367	-1,2975704	-0,0002683	-1,2975704	-0,8491952	0,39577276
VC1518	-1,2089831	-6,0729691	-1,2970656	-5,926E-06	-1,2970656	-0,8483639	0,3962354
VCA0476	1,30646943	11,0366789	1,27523003	1,077E-05	1,27523003	0,84804175	0,39641477
ribA	1,35299834	26,2272566	1,35546757	2,5593E-05	1,35546757	0,84770891	0,39660015
VC1490	1,24481004	9,28173769	1,17909914	9,0572E-06	1,17909914	0,84764377	0,39663644
VC0692	1,20097811	3,55049654	1,1445666	3,4646E-06	1,1445666	0,84737373	0,39678688
VC0784	2,37877366	10,7704999	2,00716686	1,051E-05	2,00716686	0,84710407	0,39693717
VCA0340	1,2429655	2,98020744	1,17402831	2,9081E-06	1,17402831	0,84598994	0,39755838
VCA0982	1,97329485	3,17579831	1,65030994	3,099E-06	1,65030994	0,84581676	0,39765502
VC0617	-2,1305573	-7,2645978	-2,2610342	-7,089E-06	-2,2610342	-0,84573	0,39770345
uvrA	1,26033966	2,84731976	1,18214181	2,7784E-06	1,18214181	0,84439514	0,39844867
VC1029	1,33922411	2,17885293	1,24466497	2,1261E-06	1,24466497	0,84408631	0,3986212
VC0881	1,3000683	2,59743385	1,20095994	2,5346E-06	1,20095994	0,84349905	0,39894943
VC0649	-1,1304369	-4,246649	-1,1864721	-4,144E-06	-1,1864721	-0,8434363	0,39898451
tRNA-Arg-2	-1,578537	-1,6103368	-1,5911404	-1,571E-06	-1,5911404	-0,8428636	0,39930476
VC1936	1,49975225	1,78990382	1,30450249	1,7466E-06	1,30450249	0,84233292	0,39960167
VC0981	1,06938477	6,77842393	1,07960413	6,6144E-06	1,07960413	0,84190545	0,39984092
VC1445	1,111781	10,7833867	1,1278599	1,0523E-05	1,1278599	0,8418796	0,39985539
rplC	1,53995054	194,39359	1,4169466	0,00018969	1,4169466	0,84185733	0,39986784
VC2090	2,18188058	78,7392608	2,06868106	7,6834E-05	2,06868106	0,84182326	0,39988694
VCA0666	-1,1132213	-2,6272248	-1,1973051	-2,564E-06	-1,1973051	-0,8412719	0,40019567
VC1561	1,28468339	2,60212481	1,19806791	2,5392E-06	1,19806791	0,83871475	0,40162944
VC1371	-1,1145282	-3,5880664	-1,1759946	-3,501E-06	-1,1759946	-0,8378073	0,402139
VC1235	2,33365027	14,3343223	2,40665715	1,3988E-05	2,40665715	0,83700984	0,40258709
VC1123	-1,2065989	-5,120055	-1,2669715	-4,996E-06	-1,2669715	-0,8369635	0,40261309
rdgC	1,32149038	2,57435599	1,1993362	2,5121E-06	1,1993362	0,83665274	0,40278783
VC1496	1,12013165	32,1405155	1,1668158	3,1363E-05	1,1668158	0,83664848	0,40279023
VC1616	1,88536808	18,4442907	1,6619443	1,7998E-05	1,6619443	0,83660741	0,40281334
VC1140	-1,0806394	-4,0450178	-1,1223925	-3,947E-06	-1,1223925	-0,8365517	0,40284464
alr (NC_002505 1393705..1394969)	-1,0964089	-3,3071898	-1,1672592	-3,227E-06	-1,1672592	-0,8365202	0,40286234
araD	-1,2721823	-1,1487249	-1,5090702	-1,121E-06	-1,5090702	-0,8361837	0,4030516

VC0291	1,34465869	18,8890202	1,35772338	1,8432E-05	1,35772338	0,83605118	0,40312617
VC0513	1,63930135	5,2878535	1,58845979	5,1599E-06	1,58845979	0,835746	0,40329786
VC0460	1,23702866	2,95350722	1,17098612	2,8821E-06	1,17098612	0,83538283	0,40350223
guaA	1,37569533	23,4761911	1,418793	2,2908E-05	1,418793	0,83447354	0,40401424
VC2724	-1,0959838	-3,4456833	-1,1439922	-3,362E-06	-1,1439922	-0,8332238	0,40471856
pgi	-1,5323073	-52,255703	-1,4376825	-5,099E-05	-1,4376825	-0,8327788	0,40496951
VC0714	1,43233865	40,4624969	1,49941348	3,9484E-05	1,49941348	0,83234832	0,40521242
VC2244	-1,0846459	-8,3088524	-1,1056618	-8,108E-06	-1,1056618	-0,8323339	0,40522052
rpsP	1,3993814	85,1603472	1,19777968	8,31E-05	1,19777968	0,82921336	0,40698372
VC0792	5,93616744	34,2323529	5,74218292	3,3404E-05	5,74218292	0,82888467	0,40716973
VC2051	-1,4078092	-30,138831	-1,3555371	-2,941E-05	-1,3555371	-0,8284588	0,40741075
bcp	1,1230369	25,8523623	1,14203912	2,5227E-05	1,14203912	0,82814096	0,40759072
VC2152	1,30101245	2,61454027	1,22604793	2,5513E-06	1,22604793	0,82772886	0,40782411
VC0781	-1,0656305	-3,7429309	-1,1296218	-3,652E-06	-1,1296218	-0,8267246	0,40839317
VC1563	1,36503025	1,84564147	1,28160444	1,801E-06	1,28160444	0,82667628	0,40842061
VCA0323	1,1379877	13,5047081	1,13691264	1,3178E-05	1,13691264	0,82592402	0,40884722
VC1436	-1,2341862	-27,340152	-1,1794618	-2,668E-05	-1,1794618	-0,8252633	0,40922217
VC0997	1,09162111	19,9061119	1,11468134	1,9425E-05	1,11468134	0,82512911	0,40929835
VC1566	1,46629466	1,37614593	1,39479594	1,3429E-06	1,39479594	0,82498464	0,40938034
VC1434	-1,1405029	-11,275104	-1,1254924	-1,1E-05	-1,1254924	-0,8243103	0,40976329
VC1448	1,71033508	1,94341223	1,70599706	1,8964E-06	1,70599706	0,8204013	0,41198742
VCA0186	1,63722074	22,710245	1,48240475	2,2161E-05	1,48240475	0,81978901	0,41233645
prfA	1,14968465	6,40794536	1,11234492	6,2529E-06	1,11234492	0,81960793	0,41243968
ubiE	1,12815032	5,00829925	1,0934793	4,8871E-06	1,0934793	0,81910781	0,41272494
VC1938	1,38934283	2,92786623	1,26702253	2,857E-06	1,26702253	0,81882285	0,41288753
VCA0329	-1,0649936	-3,252611	-1,1474501	-3,174E-06	-1,1474501	-0,8185454	0,41304584
VC2532	-1,0618901	-5,8641058	-1,144302	-5,722E-06	-1,144302	-0,8183628	0,41315009
VC1364	1,13303878	6,09576169	1,11393526	5,9483E-06	1,11393526	0,81830087	0,41318544
VC0548	-1,0657434	-129,51768	-1,1930915	-0,0001264	-1,1930915	-0,8180404	0,41333413
VC2271	-1,0621734	-5,6248148	-1,1278234	-5,489E-06	-1,1278234	-0,8180386	0,41333515
VCA0245	-1,2237388	-1,4610277	-1,4805	-1,426E-06	-1,4805	-0,8178645	0,41343459
VC1353	-1,153956	-4,3476138	-1,2528706	-4,242E-06	-1,2528706	-0,8174031	0,41369816
VCA0996	1,22323287	2,9154959	1,16484524	2,845E-06	1,16484524	0,81610436	0,41444049
clpA	1,24474526	23,8198127	1,23546363	2,3244E-05	1,23546363	0,81502829	0,41505617
cysM	1,31065227	2,40058018	1,2146768	2,3425E-06	1,2146768	0,81470229	0,41524277
VC0049	1,41740854	45,1189365	1,38276886	4,4027E-05	1,38276886	0,81398178	0,41565544
VC0445	1,22645245	12,9386813	1,18795913	1,2626E-05	1,18795913	0,8122601	0,41664244
VC2416	-1,2428437	-20,164162	-1,2957119	-1,968E-05	-1,2957119	-0,8103815	0,41772101
VC1919	-1,4489675	-828,17499	-1,3152266	-0,0008081	-1,3152266	-0,8096916	0,41811751
VCA0363	1,50248625	1,3463846	1,38705329	1,3138E-06	1,38705329	0,80928301	0,41835241
VC1002	1,15496356	4,44322381	1,10302159	4,3357E-06	1,10302159	0,80809349	0,4190368
VC1764	-1,3635635	-19,486938	-1,3151488	-1,902E-05	-1,3151488	-0,8069245	0,41970999
VCA0566	1,10928709	19,3986488	1,13649108	1,8929E-05	1,13649108	0,80674873	0,41981131
VCA1036	1,36597713	3,26076439	1,23625825	3,1819E-06	1,23625825	0,80662798	0,41988088
VC2039	1,09911672	7,24848853	1,10908858	7,0731E-06	1,10908858	0,80591162	0,42029384

VC1865	-1,6713551	-92,730032	-1,5501288	-9,049E-05	-1,5501288	-0,805803	0,42035646
VCA0478	1,22742379	10,1336676	1,20206492	9,8885E-06	1,20206492	0,80548686	0,42053881
VC2078	1,40416133	20,3253432	1,41325973	1,9834E-05	1,41325973	0,80484896	0,42090688
VC1873	-1,2993755	-1,3226499	-1,3901728	-1,291E-06	-1,3901728	-0,8048182	0,42092463
VC1620	1,17048669	8,78948595	1,17407601	8,5769E-06	1,17407601	0,8032256	0,42184439
VCA0192	1,28332183	8,60157299	1,26345971	8,3935E-06	1,26345971	0,8030206	0,42196287
VCA0840	1,2425242	24,9517668	1,27853601	2,4348E-05	1,27853601	0,80259715	0,42220766
sucA	-1,4913784	-40,377998	-1,4828066	-3,94E-05	-1,4828066	-0,801522	0,42282957
VC0232	1,155183	4,09829641	1,11017666	3,9992E-06	1,11017666	0,80122786	0,42299977
VC2040	1,1168004	19,0662225	1,12420331	1,8605E-05	1,12420331	0,80094698	0,42316237
VC2441	1,15663992	13,7069152	1,14814313	1,3375E-05	1,14814313	0,79973169	0,42386632
VC1565	1,33125802	1,68404559	1,3029044	1,6433E-06	1,3029044	0,79955693	0,42396758
VC1595	-1,3937399	-74,625973	-1,4659254	-7,282E-05	-1,4659254	-0,7993047	0,42411378
VC2314	-1,2701774	-1,3905904	-1,3610194	-1,357E-06	-1,3610194	-0,7986872	0,42447187
VC2362	1,41714496	12,8807079	1,40004781	1,2569E-05	1,40004781	0,79867211	0,42448062
VCA0902	-1,0689799	-2,6554709	-1,1779038	-2,591E-06	-1,1779038	-0,7979403	0,42490517
VC1696	-1,2204562	-16,689345	-1,3727283	-1,629E-05	-1,3727283	-0,7972025	0,42533347
VC0914	-1,0680098	-3,094893	-1,1627337	-3,02E-06	-1,1627337	-0,7969407	0,42548548
VC2722	-1,0738297	-3,1616825	-1,1434037	-3,085E-06	-1,1434037	-0,7966233	0,42566984
VC0573	1,61006356	105,612933	1,53491722	0,00010306	1,53491722	0,79616235	0,42593773
VCA0492	1,22540899	3,33758266	1,18895712	3,2568E-06	1,18895712	0,79473597	0,42676714
VC1272	1,384139	4,19355312	1,30281728	4,0921E-06	1,30281728	0,79344222	0,42752026
cysN	1,98326982	5,11068097	1,93994104	4,987E-06	1,93994104	0,79234276	0,42816089
VC1516	-1,2267729	-17,602333	-1,2858952	-1,718E-05	-1,2858952	-0,7923372	0,42816416
VC1713	1,28484166	3,20285615	1,22002338	3,1254E-06	1,22002338	0,79193322	0,42839965
VC2144	-1,2077685	-4,606702	-1,2478467	-4,495E-06	-1,2478467	-0,7905529	0,42920498
VC2624	1,2138358	3,51574976	1,12565884	3,4307E-06	1,12565884	0,78963368	0,42974176
VC1152	1,14101747	8,17452803	1,13837865	7,9768E-06	1,13837865	0,78935842	0,42990259
VCA0378	1,95139555	0,73166194	1,78992817	7,1396E-07	1,78992817	0,78833978	0,43049802
VC1480	1,15154855	24,5713993	1,15719563	2,3977E-05	1,15719563	0,78783955	0,43079061
VC1068	1,37952553	1,85063484	1,30693857	1,8059E-06	1,30693857	0,78617791	0,4317633
VCA0953	1,35175436	7,97487345	1,29971736	7,782E-06	1,29971736	0,7856232	0,43208832
VC1621	1,39793939	21,1908326	1,34939901	2,0678E-05	1,34939901	0,7855497	0,43213138
VC1676	1,28420292	4,10543882	1,22159557	4,0061E-06	1,22159557	0,78543129	0,43220077
oadA-2	5,0745192	60,0580729	4,85355961	5,8605E-05	4,85355961	0,78540169	0,43221815
qrr2	-1,1479403	-2,4407351	-1,2174784	-2,382E-06	-1,2174784	-0,7853705	0,43223643
ubiA	-1,0650783	-2,9165076	-1,1514468	-2,846E-06	-1,1514468	-0,7848042	0,43256843
VC2501	1,20992303	6,96282055	1,14387901	6,7944E-06	1,14387901	0,78459481	0,4326912
5Sf	-1,0814986	-9260,6815	-1,0802818	-0,0090367	-1,0802818	-0,7845208	0,43273462
VC1431	1,10364488	7,25275708	1,10984803	7,0773E-06	1,10984803	0,78408837	0,43298829
VC1215	-1,0896886	-10,066517	-1,1043484	-9,823E-06	-1,1043484	-0,7840239	0,43302615
VC2727	-1,0855511	-4,4953654	-1,14531	-4,387E-06	-1,14531	-0,7836056	0,43327159
VC2286	1,13241648	6,98312014	1,11834204	6,8142E-06	1,11834204	0,78270507	0,43380036
VC0732	1,2163961	3,97929223	1,15701342	3,883E-06	1,15701342	0,78246349	0,43394227
VC0816	1,47533549	1,51089934	1,31183495	1,4743E-06	1,31183495	0,78192228	0,43426028

hemE	1,10928036	5,3440808	1,07924147	5,2148E-06	1,07924147	0,78169225	0,43439549
VC0800	5,09681895	139,524818	4,82421181	0,00013615	4,82421181	0,78161109	0,43444322
cysS	1,08273494	8,87957672	1,07462238	8,6648E-06	1,07462238	0,78147062	0,43452579
VCA0243	-1,2387187	-1,2004176	-1,4265245	-1,171E-06	-1,4265245	-0,7802498	0,43524386
VC0694	-1,1023819	-2,6389471	-1,166273	-2,575E-06	-1,166273	-0,7795318	0,43566653
VC1268	1,26417696	2,78894507	1,20526153	2,7215E-06	1,20526153	0,7793598	0,43576783
VC1649	1,54806517	76,6104382	1,61056663	7,4757E-05	1,61056663	0,77908538	0,43592946
VCA1011	-1,1851487	-1,6918088	-1,2714591	-1,651E-06	-1,2714591	-0,7788208	0,43608533
VC0259	1,09406115	21,3314522	1,1589561	2,0815E-05	1,1589561	0,77770263	0,43674439
VC1513	-1,4779742	-31,46035	-1,4893818	-3,07E-05	-1,4893818	-0,7776036	0,43680277
VC1584	1,5698741	1,22740655	1,39268781	1,1977E-06	1,39268781	0,77738535	0,43693148
VC1365	1,44458539	4,70403211	1,39447889	4,5902E-06	1,39447889	0,77590804	0,43780333
VC2453	-1,1144746	-6,1487813	-1,2214292	-6E-06	-1,2214292	-0,7755803	0,4379969
VC1370	1,33480197	1,91762083	1,23331544	1,8712E-06	1,23331544	0,77524802	0,43819314
VC0578	1,04393682	8,43347085	1,06432118	8,2295E-06	1,06432118	0,77503547	0,43831873
VC1615	2,08006873	9,68599421	1,79292741	9,4517E-06	1,79292741	0,77482365	0,43844393
potC	1,2991631	8,78298268	1,26460443	8,5705E-06	1,26460443	0,77413095	0,43885339
VC0797	5,05209317	159,678324	4,74698195	0,00015582	4,74698195	0,77353661	0,43920492
VC1319	1,372209	104,646986	1,31750696	0,00010212	1,31750696	0,77304554	0,43949546
VC1198	1,35977262	4,80962091	1,28571421	4,6933E-06	1,28571421	0,77276174	0,43966344
VCA0225	1,18021145	5,72326839	1,12471758	5,5848E-06	1,12471758	0,77216667	0,44001575
VC0241	1,18959987	68,5450431	1,15060921	6,6887E-05	1,15060921	0,77201667	0,44010461
VC2661	-1,0904064	-4,13211	-1,1226297	-4,032E-06	-1,1226297	-0,7718319	0,440214
VCA0427	1,29050153	12,6174342	1,25267898	1,2312E-05	1,25267898	0,77158132	0,44036247
VC0616	-2,1467499	-5,5425076	-2,2460727	-5,408E-06	-2,2460727	-0,7708112	0,44081887
VC1184	1,54369079	4,29835712	1,37655719	4,1944E-06	1,37655719	0,77061351	0,44093609
VC2329	1,15396936	6,69880242	1,11800647	6,5367E-06	1,11800647	0,77053956	0,44097993
VC2097	1,1537725	4,29608907	1,11646339	4,1922E-06	1,11646339	0,76923234	0,44175543
VC2442	1,19335387	10,2417393	1,18682506	9,994E-06	1,18682506	0,76768893	0,44267205
VC1792	1,50062053	1,29705937	1,35614036	1,2657E-06	1,35614036	0,76692254	0,44312761
VC2236	1,25384558	2,47851444	1,17174713	2,4186E-06	1,17174713	0,76683125	0,44318188
VC0846	1,34657574	1,61334255	1,27562295	1,5743E-06	1,27562295	0,76565459	0,44388189
VC0794	4,7137795	133,021721	4,42271087	0,0001298	4,42271087	0,76531281	0,44408535
VCA0283	1,17239491	20,8733162	1,17480112	2,0368E-05	1,17480112	0,76371053	0,4450398
VC0289	1,40998891	3,08055313	1,2705974	3,006E-06	1,2705974	0,76302952	0,44544583
VC2323	1,30389496	2,01478937	1,24552212	1,966E-06	1,24552212	0,76294501	0,44549622
VC0889	1,30399593	7,8660348	1,23695009	7,6757E-06	1,23695009	0,76121394	0,44652933
VC1872	-1,1637956	-1,7843899	-1,2426808	-1,741E-06	-1,2426808	-0,7610992	0,44659782
cpxA	-1,0432597	-3,9486813	-1,1292548	-3,853E-06	-1,1292548	-0,7610977	0,44659872
VCA0029	2,25705709	53,9868974	2,14197393	5,2681E-05	2,14197393	0,76107447	0,44661264
VC2316	-1,2191151	-1,3659729	-1,3287543	-1,333E-06	-1,3287543	-0,7606005	0,44689572
fadB	1,70285488	1,28323996	1,44559214	1,2522E-06	1,44559214	0,75906725	0,44781236
rpmJ (NC_002505 940858..941024)	1,34269389	5,35675573	1,27000008	5,2272E-06	1,27000008	0,75895733	0,44787812
VC1808	-1,179775	-7,6161528	-1,3124436	-7,432E-06	-1,3124436	-0,7581083	0,44838621
VCA0020	1,34192787	1,81466755	1,23571544	1,7708E-06	1,23571544	0,75761185	0,44868341

VC1403	1,43480062	1,2538358	1,36002294	1,2235E-06	1,36002294	0,7575108	0,44874393
VC1186	-1,0641632	-4,4551701	-1,1474586	-4,347E-06	-1,1474586	-0,7573688	0,44882894
VC0795	4,53718952	168,068879	4,40528742	0,000164	4,40528742	0,75725166	0,44889916
VC0668	1,39202043	2,2337293	1,24933633	2,1797E-06	1,24933633	0,75721136	0,44892328
VCA0705	-1,2449831	-1,234528	-1,3659545	-1,205E-06	-1,3659545	-0,7568718	0,44912668
VC0713	1,39804007	1106,72743	1,65866764	0,00107995	1,65866764	0,75670794	0,44922491
VC0796	4,43161803	380,288887	4,43085037	0,00037109	4,43085037	0,75611449	0,44958061
rpmG	1,09440936	778,126843	1,35818059	0,0007593	1,35818059	0,75556375	0,44991085
VC2745	1,31833044	1,75454101	1,24329689	1,7121E-06	1,24329689	0,75556011	0,44991301
VC2668	1,31762231	6,88662363	1,2075136	6,72E-06	1,2075136	0,75482357	0,45035489
VC0798	4,6899084	189,633707	4,37711831	0,00018505	4,37711831	0,75477271	0,45038542
VCA0030	1,83774504	12,1620007	1,82354391	1,1868E-05	1,82354391	0,75370698	0,45102525
VC0871	-1,3527492	-32,128738	-1,2972344	-3,135E-05	-1,2972344	-0,7522986	0,45187153
gidB	1,16590669	7,96241738	1,15522817	7,7698E-06	1,15522817	0,75209634	0,45199317
VC1511	-1,5406735	-29,78586	-1,562204	-2,907E-05	-1,562204	-0,7520313	0,45203226
VCA0715	1,27750603	2,07219158	1,20014101	2,0221E-06	1,20014101	0,75200512	0,45204801
VC0801	4,70833461	77,9907342	4,26746816	7,6104E-05	4,26746816	0,75193403	0,45209078
rpsU	-1,0923951	-111,3344	-1,2231504	-0,0001086	-1,2231504	-0,7507695	0,45279143
VC1252	-1,0627882	-4,9431326	-1,1242687	-4,824E-06	-1,1242687	-0,7492931	0,45368061
VC1852	-1,0775833	-7,1299113	-1,0817765	-6,957E-06	-1,0817765	-0,7479806	0,45447186
VCA0312	-1,0383593	-2,9386862	-1,1354583	-2,868E-06	-1,1354583	-0,7478241	0,45456628
VC0440	-1,1384931	-8,5435649	-1,177112	-8,337E-06	-1,177112	-0,7472861	0,45489091
VC0945	1,24425631	4,63689604	1,20634902	4,5247E-06	1,20634902	0,74692766	0,45510726
rbn	1,2390756	2,26536885	1,17869545	2,2106E-06	1,17869545	0,74660608	0,4553014
VC0799	4,73118625	102,429609	4,237863	9,9952E-05	4,237863	0,74657171	0,45532218
fmt	1,12812766	4,39008604	1,08821128	4,2839E-06	1,08821128	0,74590414	0,45572535
VC1250	1,17259925	4,22683191	1,10906779	4,1246E-06	1,10906779	0,74559213	0,45591387
VC1765	-1,2960566	-28,472973	-1,2352863	-2,778E-05	-1,2352863	-0,745148	0,45618228
rumA	1,16810765	3,14466933	1,12483341	3,0686E-06	1,12483341	0,74448643	0,45658228
VC0239	1,11493763	13,8073903	1,14294647	1,3473E-05	1,14294647	0,74413083	0,45679736
VC1317	1,18176624	36,9062021	1,16100648	3,6013E-05	1,16100648	0,74396131	0,45689992
VCA0673	1,33336978	8,58047408	1,28256129	8,3729E-06	1,28256129	0,74323282	0,45734077
tRNA-His-2	-1,3928847	-17,706689	-1,3418412	-1,728E-05	-1,3418412	-0,7430713	0,45743855
pyrE	-1,2050254	-15,085492	-1,1561821	-1,472E-05	-1,1561821	-0,7429847	0,45749099
VC0216	-1,2536932	-81,658241	-1,3697404	-7,968E-05	-1,3697404	-0,7420812	0,45803815
lpxB	1,26657504	1,97802059	1,20337308	1,9302E-06	1,20337308	0,74008403	0,45924904
VC0263	1,1748064	3,42200828	1,11808378	3,3392E-06	1,11808378	0,73982426	0,45940666
VC2274	1,25957009	4,25598268	1,21528353	4,153E-06	1,21528353	0,73965081	0,45951194
VCA1096	-1,2525584	-1,2354901	-1,3461372	-1,206E-06	-1,3461372	-0,7394837	0,45961337
VC2333	-1,1676467	-19,923117	-1,2028784	-1,944E-05	-1,2028784	-0,7386488	0,46012031
VC1248	-1,1928729	-2,4848022	-1,3112793	-2,425E-06	-1,3112793	-0,7378432	0,46060979
VC1514	-1,4176788	-37,500134	-1,4160475	-3,659E-05	-1,4160475	-0,7378275	0,46061935
VC0337	-1,0892675	-2,3310948	-1,1687122	-2,275E-06	-1,1687122	-0,7375919	0,46076253
VCA1045	-1,2056925	-4,1515889	-1,3149922	-4,051E-06	-1,3149922	-0,737408	0,46087427
VC0074	1,23846219	2,68866866	1,18492192	2,6236E-06	1,18492192	0,73648858	0,46143344

VC1819	-1,2953405	-0,9331086	-1,4792803	-9,105E-07	-1,4792803	-0,7356296	0,46195615
VC1424	-1,1073976	-10,345073	-1,1267948	-1,009E-05	-1,1267948	-0,7348585	0,46242568
VC1850	1,0826239	4,97302971	1,07499318	4,8527E-06	1,07499318	0,73432496	0,46275074
cyaY	1,13443186	7,35068763	1,12548103	7,1729E-06	1,12548103	0,73355267	0,46322145
VCA0086	1,29518836	2,7862286	1,20008755	2,7188E-06	1,20008755	0,73338374	0,46332444
VC1537	-1,1520717	-24,580868	-1,1912482	-2,399E-05	-1,1912482	-0,7331583	0,46346189
VC0185	1,31419959	2,50822535	1,23010398	2,4475E-06	1,23010398	0,73285015	0,46364986
VC0502	1,14803345	11,7375667	1,12452304	1,1454E-05	1,12452304	0,73158831	0,46441992
VC1534	1,32749911	2,00015949	1,20110371	1,9518E-06	1,20110371	0,73155319	0,46444136
VC2765	1,23475782	56,4688018	1,08311299	5,5103E-05	1,08311299	0,73101036	0,46477288
VC1309	1,19815349	2,65344356	1,14376444	2,5893E-06	1,14376444	0,73064595	0,46499547
VC2348	-1,2880994	-14,671102	-1,3228303	-1,432E-05	-1,3228303	-0,7304584	0,46511008
VC1298	1,82639449	16,748918	1,62179904	1,6344E-05	1,62179904	0,73011053	0,46532267
csrD	-1,1668514	-2213,1391	-1,2826502	-0,0021596	-1,2826502	-0,7300375	0,46536727
VC0614	-1,6080429	-1,7580107	-1,7666064	-1,715E-06	-1,7666064	-0,7293819	0,46576815
VCA0308	1,16900704	20,5271927	1,21587477	2,0031E-05	1,21587477	0,72852875	0,46629002
VCA0497	1,17689633	19,1418448	1,17964098	1,8679E-05	1,17964098	0,72837471	0,46638429
rpsJ	1,4491177	157,807618	1,25637312	0,00015399	1,25637312	0,72815176	0,46652072
VCA0906	-1,188999	-1,504026	-1,2664206	-1,468E-06	-1,2664206	-0,7275838	0,46686842
VC2298	1,27801942	18,9200816	1,21904137	1,8462E-05	1,21904137	0,7274274	0,46696423
VC1951	1,39113924	51,1391058	1,42667766	4,9902E-05	1,42667766	0,72734617	0,46701397
VC2215	1,23701998	2,16364143	1,18671883	2,1113E-06	1,18671883	0,72649367	0,46753622
VC2380	-1,1226491	-2,3015781	-1,1813897	-2,246E-06	-1,1813897	-0,7260939	0,46778121
VCA0658	1,59652357	7,97931139	1,40903473	7,7863E-06	1,40903473	0,72489658	0,46851553
VC0367	1,25671214	209,555251	1,18718848	0,00020449	1,18718848	0,72428967	0,46888796
VC1485	1,17112156	17,3218723	1,13716296	1,6903E-05	1,13716296	0,7242556	0,46890886
VC1011	1,20613753	2,54713138	1,1473908	2,4855E-06	1,1473908	0,72421898	0,46893134
VCA0053	-1,4128978	-106,44818	-1,5674129	-0,0001039	-1,5674129	-0,724106	0,46900071
VC2105	1,45676247	7,2099002	1,31103541	7,0355E-06	1,31103541	0,72390343	0,46912507
rpmE	1,43106652	110,721764	1,18016529	0,00010804	1,18016529	0,72246108	0,47001108
VCA0231	1,63634538	0,89151635	1,4846817	8,6995E-07	1,4846817	0,72230183	0,47010896
VC2524	1,1530233	3,92851942	1,0960221	3,8335E-06	1,0960221	0,72200684	0,4702903
VC0631	1,14336911	20,3704466	1,16487243	1,9878E-05	1,16487243	0,72174269	0,47045273
VCA0362	1,20705856	5,47511647	1,14515725	5,3427E-06	1,14515725	0,72068404	0,47110396
VCA0972	1,19523938	2,63341466	1,1406774	2,5697E-06	1,1406774	0,72054493	0,47118958
VC2738	-1,4229774	-170,67088	-1,5834491	-0,0001665	-1,5834491	-0,7205362	0,47119493
VC2749	-1,0736202	-2,3369239	-1,1597035	-2,28E-06	-1,1597035	-0,7200231	0,47151077
rnhA	-1,0504706	-2,6511372	-1,1393449	-2,587E-06	-1,1393449	-0,7197576	0,47167427
VC2093	1,2853224	1,9721024	1,19139975	1,9244E-06	1,19139975	0,71884896	0,47223401
VC1499	1,24258056	2,11558193	1,17719111	2,0644E-06	1,17719111	0,71870557	0,47232237
VC2673	1,20044052	4,39823397	1,15653139	4,2918E-06	1,15653139	0,71822487	0,47261868
VC0983	1,09758409	11,1872397	1,1145173	1,0917E-05	1,1145173	0,71795132	0,47278733
tyrA	1,25317455	3,52800988	1,14815524	3,4427E-06	1,14815524	0,71792762	0,47280195
VC0924	1,24068595	2,16760024	1,17213809	2,1152E-06	1,17213809	0,71787349	0,47283532
VC1835	1,37930901	194,601366	1,24343269	0,00018989	1,24343269	0,71716593	0,47327175

VC1085	-1,2326666	-3,4213227	-1,2965909	-3,339E-06	-1,2965909	-0,7162926	0,47381072
VCA0235	-1,4550589	-132,53877	-1,3549014	-0,0001293	-1,3549014	-0,7161194	0,47391764
VC2319	-1,1557384	-1,8668322	-1,2011063	-1,822E-06	-1,2011063	-0,7153322	0,47440384
VC0700	1,22072281	2,16477662	1,1707138	2,1124E-06	1,1707138	0,71466603	0,47481545
VC0551	-1,3190461	-25,528377	-1,3909516	-2,491E-05	-1,3909516	-0,7132483	0,47569215
cysE	1,13435122	6,08632216	1,09679792	5,9391E-06	1,09679792	0,71311439	0,475775
VC2650	1,26657785	9,32057012	1,18066646	9,0951E-06	1,18066646	0,71278894	0,47597641
motB	-1,0881725	-5,8409454	-1,1286329	-5,7E-06	-1,1286329	-0,7126955	0,47603421
VCA1087	-1,1685881	-1,5133643	-1,2518317	-1,477E-06	-1,2518317	-0,7125593	0,47611851
VCA0753	1,5563583	1,28376814	1,5362468	1,2527E-06	1,5362468	0,71167573	0,47666563
VC1871	-1,3609562	-168,41567	-1,4587001	-0,0001643	-1,4587001	-0,7113982	0,47683755
VC2569	1,37293451	9,65353595	1,3356533	9,42E-06	1,3356533	0,71103091	0,47706513
VC0710	-1,0979178	-5,8291199	-1,1174696	-5,688E-06	-1,1174696	-0,7108269	0,47719153
VC2201	-1,0650357	-7,3830371	-1,0465751	-7,204E-06	-1,0465751	-0,7100284	0,47768652
VCA0388	1,14892076	3,44731853	1,10216295	3,3639E-06	1,10216295	0,70896109	0,47834865
VC0613	-1,5412824	-1,7937489	-1,6985649	-1,75E-06	-1,6985649	-0,7085484	0,47860479
ccrB	1,1177501	11,8374452	1,10455379	1,1551E-05	1,10455379	0,70834142	0,47873329
VC2667	1,4266768	5,705579	1,29687338	5,5676E-06	1,29687338	0,70815501	0,47884904
flgI	-1,1242362	-11,831065	-1,1891147	-1,154E-05	-1,1891147	-0,7080613	0,4789072
VC1249	-1,3528284	-33,134635	-1,4423864	-3,233E-05	-1,4423864	-0,7078768	0,47902178
VCA0659	-1,2240517	-9,6052284	-1,2784787	-9,373E-06	-1,2784787	-0,7077826	0,47908029
VC2077	1,4505466	24,4983973	1,46035318	2,3906E-05	1,46035318	0,70717895	0,47945531
5Sh	-1,0723161	-3354,9342	-1,0299555	-0,0032738	-1,0299555	-0,7071068	0,47950015
VC1756	1,19845997	2,62173236	1,13570661	2,5583E-06	1,13570661	0,70695033	0,47959737
metH	1,21714453	2,29297633	1,15598977	2,2375E-06	1,15598977	0,70548616	0,48050777
VC0106	1,23851332	2,04070381	1,17678428	1,9913E-06	1,17678428	0,70512712	0,48073116
VC1574	1,42946055	51,4925971	1,35724072	5,0247E-05	1,35724072	0,70510579	0,48074443
VC0429	1,25193327	2,04927417	1,17595245	1,9997E-06	1,17595245	0,70507663	0,48076258
VCA0571	1,06510035	16,6752555	1,0739858	1,6272E-05	1,0739858	0,70473188	0,48097713
VCA0767	1,21180258	2,9852291	1,11737164	2,913E-06	1,11737164	0,70459284	0,48106368
VCA0501	1,18550906	5,13327696	1,14226667	5,0091E-06	1,14226667	0,70440142	0,48118285
VC1882	1,23554739	2,14881401	1,16673183	2,0968E-06	1,16673183	0,70431947	0,48123387
VC2433	-1,3044418	-24,722595	-1,2412974	-2,412E-05	-1,2412974	-0,7041328	0,48135014
tRNA-Gly-6	-1,3416324	-33,423215	-1,2702599	-3,261E-05	-1,2702599	-0,7041278	0,48135322
VC2470	-1,1359306	-13,817939	-1,1717201	-1,348E-05	-1,1717201	-0,703657	0,48164646
VC1926	-1,1941061	-1,608424	-1,3585892	-1,57E-06	-1,3585892	-0,7030041	0,48205322
VC2552	1,24942486	4,02277495	1,16206952	3,9255E-06	1,16206952	0,70069	0,48349653
carB	1,24431928	4,49553196	1,22401414	4,3868E-06	1,22401414	0,69974593	0,48408602
VC0630	-1,0189588	-3,0563921	-1,1145422	-2,982E-06	-1,1145422	-0,6970947	0,48574358
cmk	-1,1448462	-6,9612485	-1,1598399	-6,793E-06	-1,1598399	-0,6950636	0,4870155
VC0389	1,15442095	4,8809875	1,10448367	4,7629E-06	1,10448367	0,69494229	0,4870915
VC2608	1,20696445	2,54900719	1,13471006	2,4873E-06	1,13471006	0,69467397	0,48725967
VC2136	-1,0364695	-3,7598502	-1,0893666	-3,669E-06	-1,0893666	-0,6946011	0,48730536
VC2080	-1,044935	-3,0793824	-1,1101568	-3,005E-06	-1,1101568	-0,6944597	0,48739401
VC2255	-1,0519283	-4,7294027	-1,0979557	-4,615E-06	-1,0979557	-0,6942361	0,48753419

VCA0359	1,18512254	2,56404248	1,13361443	2,502E-06	1,13361443	0,69405885	0,48764533
VC0068	1,63781554	1,39220508	1,36875363	1,3585E-06	1,36875363	0,69349565	0,48799858
VC1698	-1,0595743	-4,2864834	-1,1973191	-4,183E-06	-1,1973191	-0,69223	0,48879292
VCA0662	1,20720899	5,69423119	1,13929582	5,5565E-06	1,13929582	0,69169847	0,48912673
mgsA	-1,0587802	-6,1999128	-1,1528346	-6,05E-06	-1,1528346	-0,6916436	0,48916122
VC1906	1,24423506	2,49574386	1,14480677	2,4354E-06	1,14480677	0,69151568	0,48924156
VC0547	-1,1439316	-18,110171	-1,1236256	-1,767E-05	-1,1236256	-0,6908298	0,48967253
VC2747	-1,1113692	-1,7564442	-1,1987625	-1,714E-06	-1,1987625	-0,6901729	0,49008548
VC1293	1,34521444	14,6594956	1,34178994	1,4305E-05	1,34178994	0,69016657	0,49008949
VC2472	1,16330133	3,6837534	1,1180007	3,5946E-06	1,1180007	0,68986634	0,49027827
lolB	-1,0632463	-2,7967198	-1,1202375	-2,729E-06	-1,1202375	-0,6897918	0,49032519
VC1035	-1,1256658	-1,5595452	-1,2261605	-1,522E-06	-1,2261605	-0,6894319	0,49055156
VC1719	1,69738642	55,2485296	1,76974634	5,3912E-05	1,76974634	0,68930736	0,49062991
VC0456	1,14033075	3,33473336	1,10683155	3,2541E-06	1,10683155	0,6885521	0,4911052
VC1668	1,21347379	2,7053437	1,14312956	2,6399E-06	1,14312956	0,68800381	0,49145041
glyQ	1,19926899	6,76452168	1,15578444	6,6009E-06	1,15578444	0,68794178	0,49148947
tRNA-Asp-5	1,2725227	8,37386422	1,21905655	8,1713E-06	1,21905655	0,68704379	0,49205517
VC2207	-1,0866179	-36,743407	-1,1378426	-3,585E-05	-1,1378426	-0,6870433	0,49205547
VC1666	-1,1512902	-1,4422069	-1,2448605	-1,407E-06	-1,2448605	-0,6869743	0,49209895
rpsM	1,17770018	77,6330795	1,06838056	7,5755E-05	1,06838056	0,68682975	0,49219004
VC2365	1,1506143	4,69747056	1,1099764	4,5838E-06	1,1099764	0,68656022	0,49235993
VCA0468	1,18916884	9,3494772	1,16693821	9,1233E-06	1,16693821	0,68626644	0,49254515
VC0818	1,25366481	1,88686597	1,1814187	1,8412E-06	1,1814187	0,68612791	0,49263248
VCA0470	1,2327295	4,83040202	1,16992545	4,7135E-06	1,16992545	0,68604455	0,49268505
16Sd	1,04983716	456,318872	1,23482004	0,00044528	1,23482004	0,68588033	0,49278861
VC1294	1,48645733	1,29382003	1,39219272	1,2625E-06	1,39219272	0,68584261	0,49281239
tRNA-Ser-1	1,80461042	39,272826	1,91748133	3,8323E-05	1,91748133	0,6851942	0,49322144
flgA	-1,0394796	-4,3120726	-1,1014826	-4,208E-06	-1,1014826	-0,6849899	0,4933503
infA	-1,2569805	-42,708389	-1,2525892	-4,168E-05	-1,2525892	-0,6844126	0,4937147
VC1102	1,33640244	1,9305086	1,20429969	1,8838E-06	1,20429969	0,68415131	0,49387965
VC0145	-1,0684458	-6,0398582	-1,0984446	-5,894E-06	-1,0984446	-0,6835079	0,49428601
VCA0506	1,15025346	4,06045201	1,10439696	3,9622E-06	1,10439696	0,68309578	0,49454635
VC0114	1,27930087	5,73274645	1,22959344	5,5941E-06	1,22959344	0,6819474	0,49527225
VCA0680	1,62809375	43,6926243	1,68685784	4,2636E-05	1,68685784	0,68049433	0,49619153
VC1299	1,33308687	7,14876872	1,32190406	6,9758E-06	1,32190406	0,68041912	0,49623916
VC1577	1,50121563	181,215745	1,3424404	0,00017683	1,3424404	0,67960315	0,4967558
VCA0532	1,22916367	3,02965399	1,17115761	2,9564E-06	1,17115761	0,67909635	0,49707685
VC0432	-1,233232	-69,175421	-1,3099807	-6,75E-05	-1,3099807	-0,6789323	0,49718082
VC0079	-1,1127014	-10,592637	-1,1325679	-1,034E-05	-1,1325679	-0,6780005	0,49777139
VC2252	1,09541066	8,13161457	1,07763276	7,9349E-06	1,07763276	0,67785941	0,49786087
VC1210	1,22665989	2,98830955	1,13164182	2,916E-06	1,13164182	0,67780973	0,49789237
VC0574	1,41547404	52,8138528	1,38799019	5,1536E-05	1,38799019	0,67770624	0,49795801
VC0790	2,00857801	54,9110584	2,01978031	5,3583E-05	2,01978031	0,67633751	0,49882643
VC1751	1,12379552	4,25864977	1,0925925	4,1556E-06	1,0925925	0,67630048	0,49884991
tRNA-Leu-5	-1,1518402	-3,670646	-1,1887896	-3,582E-06	-1,1887896	-0,6758121	0,49915995

VC1155	-1,1723386	-9,7792667	-1,2532495	-9,543E-06	-1,2532495	-0,6749325	0,49971869
VC0112	1,3085287	79,2884497	1,20996809	7,737E-05	1,20996809	0,67479745	0,49980449
rfaH	-1,0669262	-5,3914058	-1,1163893	-5,261E-06	-1,1163893	-0,6746545	0,49989531
VC0837	1,1250743	12,5502522	1,13971193	1,2247E-05	1,13971193	0,67392935	0,50035626
VCA0580	1,15190941	3,27841018	1,09674278	3,1991E-06	1,09674278	0,67365343	0,5005317
VC2183	-1,181827	-11,531575	-1,2147373	-1,125E-05	-1,2147373	-0,6735008	0,50062874
VC0260	1,07779086	6,42855261	1,08999155	6,273E-06	1,08999155	0,67318262	0,50083115
VCA0829	1,38748183	1,40807351	1,23983313	1,374E-06	1,23983313	0,67254516	0,50123672
minC	1,25625397	3,50917638	1,16928039	3,4243E-06	1,16928039	0,67250175	0,50126436
VC1282	1,37677426	1,52552612	1,21929705	1,4886E-06	1,21929705	0,67248217	0,50127681
VC0200	1,63707443	12,3973802	1,59315562	1,2097E-05	1,59315562	0,67122394	0,50207791
VC1745	1,2272361	2,10666209	1,15343511	2,0557E-06	1,15343511	0,67105435	0,50218593
VC2645	1,32380189	2,91894427	1,20062383	2,8483E-06	1,20062383	0,67093128	0,50226434
ulaA	-1,1197994	-1,5386792	-1,3555654	-1,501E-06	-1,3555654	-0,6708763	0,50229936
VCA0264	1,41272223	1,43820692	1,23292292	1,4034E-06	1,23292292	0,67087566	0,50229977
VC0194	1,5890762	0,90115372	1,3984245	8,7935E-07	1,3984245	0,67014788	0,50276355
VC0381	-1,1245257	-9,0289266	-1,13371	-8,811E-06	-1,13371	-0,6692956	0,50330699
flgC	-1,1121559	-26,685921	-1,121537	-2,604E-05	-1,121537	-0,6692477	0,50333749
VCA0595	1,21469629	2,46690583	1,12871431	2,4072E-06	1,12871431	0,66895357	0,50352512
VC0618	-1,8361624	-5,7469774	-1,9518426	-5,608E-06	-1,9518426	-0,6676129	0,50438076
VCA0558	-1,149255	-3,2652401	-1,2301299	-3,186E-06	-1,2301299	-0,6673696	0,50453609
VC2128	-1,0911765	-7,9815701	-1,1514385	-7,788E-06	-1,1514385	-0,6672991	0,5045811
VC2511	1,19863762	2,17739388	1,14574115	2,1247E-06	1,14574115	0,66609315	0,50535158
VC2185	-1,1520283	-8,9827025	-1,1678047	-8,765E-06	-1,1678047	-0,6659239	0,5054598
VC0391	1,26718723	4,17378139	1,20360985	4,0728E-06	1,20360985	0,6658535	0,50550478
VCA0402	1,28210845	2,13395342	1,22492706	2,0823E-06	1,22492706	0,6655843	0,50567687
VC2229	1,09203785	4,68871446	1,06629894	4,5753E-06	1,06629894	0,66540189	0,5057935
VC1978	1,46294137	1,25316983	1,26683034	1,2229E-06	1,26683034	0,66523461	0,50590048
VC1794	1,27730611	1,43762323	1,22859985	1,4028E-06	1,22859985	0,66513008	0,50596732
astD	-1,3595405	-3,6076254	-1,5534121	-3,52E-06	-1,5534121	-0,6640112	0,5066832
VCA0905	1,56562045	5,33898831	1,37487244	5,2098E-06	1,37487244	0,66302904	0,507312
fliJ	-1,0385333	-2,8965764	-1,1191853	-2,827E-06	-1,1191853	-0,6624709	0,50766953
VC1917	-1,1193195	-1,1684693	-1,2860808	-1,14E-06	-1,2860808	-0,6623215	0,50776523
VC1363	1,12335741	3,50603456	1,08681298	3,4212E-06	1,08681298	0,66149754	0,50829332
VC1614	1,76911618	11,7243843	1,56363513	1,1441E-05	1,56363513	0,66011124	0,50918249
VCA1066	-1,2128898	-2,4391582	-1,2970486	-2,38E-06	-1,2970486	-0,6598831	0,50932888
VC0212	-1,1113544	-7,4789494	-1,1247838	-7,298E-06	-1,1247838	-0,6595203	0,50956174
VCA0100	1,18233423	2,83246014	1,10779438	2,7639E-06	1,10779438	0,659223	0,5097526
glpE	-1,0375241	-3,2500719	-1,0977877	-3,171E-06	-1,0977877	-0,6584551	0,51024579
flgF	-1,1112728	-19,566544	-1,1422198	-1,909E-05	-1,1422198	-0,6583061	0,51034146
VC2095	-1,1674537	-18,651409	-1,2801143	-1,82E-05	-1,2801143	-0,6582827	0,51035651
VC1744	1,18715109	2,64611917	1,11536827	2,5821E-06	1,11536827	0,65799209	0,51054324
VCA0892	1,40404071	27,3985918	1,33832338	2,6736E-05	1,33832338	0,65777391	0,51068346
VC1644	1,35229402	29,7586867	1,33510738	2,9039E-05	1,33510738	0,656561	0,51146327
VC0581	1,14008819	4,6630428	1,09217775	4,5502E-06	1,09217775	0,65653794	0,51147809

fre	1,14349998	3,423351	1,10070436	3,3405E-06	1,10070436	0,65636688	0,51158812
VCA0877	1,21255828	2,34451934	1,14328398	2,2878E-06	1,14328398	0,6563311	0,51161113
VC1515	-1,2206084	-12,008041	-1,2665291	-1,172E-05	-1,2665291	-0,6563154	0,51162125
VC0676	-1,1164383	-1,3981524	-1,2286591	-1,364E-06	-1,2286591	-0,6560119	0,51181646
VC1622	1,29550946	12,2576396	1,24250456	1,1961E-05	1,24250456	0,65555855	0,51210823
flgB	-1,120987	-157,81392	-1,1965164	-0,000154	-1,1965164	-0,6551028	0,51240161
VC0377	-1,0763848	-28,260411	-1,1054178	-2,758E-05	-1,1054178	-0,6540901	0,51305382
VC2421	1,1943995	2,43622832	1,12433746	2,3773E-06	1,12433746	0,65405351	0,51307736
VC0962	1,33243285	5,0950789	1,31097441	4,9718E-06	1,31097441	0,65329485	0,51356625
VCA0417	1,19927358	2,28993341	1,14672079	2,2345E-06	1,14672079	0,65268142	0,51396172
ribH	-1,1377554	-50,627182	-1,1801191	-4,94E-05	-1,1801191	-0,6525566	0,51404222
VC0115	1,2207999	4,36744032	1,18857068	4,2618E-06	1,18857068	0,65112485	0,51496593
VC0535	1,29631784	1,64933869	1,1870183	1,6094E-06	1,1870183	0,65047926	0,51538272
VC0352	1,18453773	2,72419998	1,10919652	2,6583E-06	1,10919652	0,65047647	0,51538452
VC1303	1,22295322	2,18394355	1,13790426	2,1311E-06	1,13790426	0,65009922	0,51562815
VC1375	1,33265213	2,86596547	1,25523736	2,7966E-06	1,25523736	0,64977599	0,51583697
VC1898	-1,1698191	-23,175429	-1,2569865	-2,261E-05	-1,2569865	-0,6482578	0,51681828
secF	-1,0647825	-10,680751	-1,1004053	-1,042E-05	-1,1004053	-0,6482243	0,5168399
VC2334	1,20549445	2,40088867	1,12366259	2,3428E-06	1,12366259	0,6476307	0,51722386
tRNA-Thr-4	-1,3082735	-36,258521	-1,2644844	-3,538E-05	-1,2644844	-0,6474377	0,51734877
VC1522	-1,0748166	-2,5845129	-1,1619175	-2,522E-06	-1,1619175	-0,6473603	0,51739881
tRNA-Thr-5	-1,3763333	-48,299178	-1,3439699	-4,713E-05	-1,3439699	-0,6470737	0,5175843
VC1928	1,90352852	1,96275694	1,78141142	1,9153E-06	1,78141142	0,64681296	0,51775305
ispD	1,20158915	1,96549765	1,1526279	1,9179E-06	1,1526279	0,64659497	0,51789413
modC	-1,0946089	-2,0325542	-1,1479066	-1,983E-06	-1,1479066	-0,646287	0,51809353
VC0198	1,49907656	1,18091773	1,29699759	1,1523E-06	1,29699759	0,64544286	0,51864026
VC1508	1,21430881	7,5239194	1,17571346	7,3419E-06	1,17571346	0,64476055	0,5190824
trpD	-1,0943899	-1,6355872	-1,185063	-1,596E-06	-1,185063	-0,644655	0,5191508
VC1567	1,28491144	1,26934365	1,24466001	1,2386E-06	1,24466001	0,64425484	0,51941022
VCA0939	-1,0774598	-2,0419801	-1,1453142	-1,993E-06	-1,1453142	-0,6441666	0,51946742
VCA0077	1,18572584	2,20846299	1,13319973	2,155E-06	1,13319973	0,64319901	0,520095
VC0234	1,12034664	6,4229833	1,10883012	6,2676E-06	1,10883012	0,64249967	0,52054883
ipk	-1,1383016	-8,0135649	-1,1602449	-7,82E-06	-1,1602449	-0,6417416	0,52104099
VC2622	-1,1354119	-1,0723788	-1,293346	-1,046E-06	-1,293346	-0,6414915	0,52120343
VCA0923	-1,0762362	-4,2600926	-1,1991956	-4,157E-06	-1,1991956	-0,6408935	0,52159189
argC	1,43557514	1,05712358	1,29753519	1,0316E-06	1,29753519	0,64085898	0,52161434
VC0250	1,28087957	112,782364	1,21834086	0,00011005	1,21834086	0,6404859	0,52185679
mscL	1,20952149	2,64686921	1,11620031	2,5828E-06	1,11620031	0,64028627	0,52198653
VC2671	-1,123077	-1,3886741	-1,2182678	-1,355E-06	-1,2182678	-0,6402509	0,52200955
VC1833	1,12418048	5,57007846	1,11586734	5,4353E-06	1,11586734	0,63853711	0,52312414
VCA0019	1,45017673	1,08485164	1,28609837	1,0586E-06	1,28609837	0,63820025	0,52334336
VCA0645	1,43163533	0,98851248	1,31818325	9,646E-07	1,31818325	0,63799498	0,52347697
VC2052	-1,2842486	-20,922818	-1,2590266	-2,042E-05	-1,2590266	-0,6375283	0,52378083
VC1712	1,45112095	1,35312268	1,29101588	1,3204E-06	1,29101588	0,63719426	0,52399834
VC2635	1,17600085	3,44225436	1,1087926	3,359E-06	1,1087926	0,63699231	0,52412988

VC1701	1,53257596	2,35473272	1,39843542	2,2978E-06	1,39843542	0,63676086	0,52428066
ompR	1,24309248	6,13999723	1,1614405	5,9915E-06	1,1614405	0,63588948	0,52484849
VC2278	1,22739342	5,80190161	1,20449902	5,6615E-06	1,20449902	0,63530007	0,52523276
VCA0474	1,16681581	2,25498182	1,12688528	2,2004E-06	1,12688528	0,6352867	0,52524147
VC2012	-1,1091443	-1,3577642	-1,2190872	-1,325E-06	-1,2190872	-0,6341553	0,52597951
VCA0095	1,76812608	0,79902636	1,43445669	7,797E-07	1,43445669	0,63385029	0,52617856
dnaG	1,13256167	5,26068678	1,11801377	5,1334E-06	1,11801377	0,63382115	0,52619758
VC1491	-1,2017158	-18,688689	-1,1980879	-1,824E-05	-1,1980879	-0,6326764	0,526945
VCA0066	1,6362912	0,67964446	1,48848913	6,632E-07	1,48848913	0,63264704	0,52696418
VC0008	1,36212912	3,34517929	1,3168812	3,2643E-06	1,3168812	0,63190968	0,52744593
VCA0166	-1,4754827	-833,58124	-1,1754851	-0,0008134	-1,1754851	-0,6303903	0,5284393
VC1497	1,16585946	42,3413808	1,15577035	4,1317E-05	1,15577035	0,63019537	0,52856681
VC1853	1,08645712	15,143414	1,05338582	1,4777E-05	1,05338582	0,62988027	0,52877296
VC0660	1,26395752	5,31513752	1,23090828	5,1866E-06	1,23090828	0,62966898	0,52891122
VC2277	1,17132934	11,0827039	1,15307871	1,0815E-05	1,15307871	0,62902243	0,5293344
VCA0193	-1,1045427	-1,4635398	-1,197891	-1,428E-06	-1,197891	-0,628745	0,52951601
VC0603	1,13450625	3,25259421	1,08435471	3,1739E-06	1,08435471	0,62842428	0,52972605
VC0257	-1,3567611	-8,7691603	-1,344446	-8,557E-06	-1,344446	-0,6282017	0,52987185
VCA0356a	1,21236694	7,71048324	1,17486122	7,524E-06	1,17486122	0,6274534	0,53036209
VC1754	1,12467236	3,13830006	1,08727315	3,0624E-06	1,08727315	0,62743177	0,53037628
glgA	-1,1168146	-17,398195	-1,1619555	-1,698E-05	-1,1619555	-0,626273	0,53113592
VC2533	-1,1197754	-1,4102385	-1,2041785	-1,376E-06	-1,2041785	-0,6260228	0,5313
VC1957	1,10059123	10,6307111	1,08099856	1,0374E-05	1,08099856	0,62592096	0,5313668
VC1711	1,34515567	1,18586852	1,25949052	1,1572E-06	1,25949052	0,62576887	0,53146658
VC2382	-1,1087917	-1,5580687	-1,1825397	-1,52E-06	-1,1825397	-0,6252498	0,53180716
VCA1105	-1,1809489	-3,8408278	-1,2659882	-3,748E-06	-1,2659882	-0,6251527	0,53187085
VCA0815	1,21031894	5,54784589	1,15610097	5,4136E-06	1,15610097	0,62503305	0,5319494
rimO	1,27012766	1,36948454	1,21665936	1,3364E-06	1,21665936	0,62447605	0,53231502
VC0229	1,07088103	8,41927785	1,07579742	8,2156E-06	1,07579742	0,62441043	0,53235811
VC0132	-1,0527188	-2,2381083	-1,122903	-2,184E-06	-1,122903	-0,6234784	0,53297023
VC0036	1,35967754	20,7637919	1,34824632	2,0261E-05	1,34824632	0,62315373	0,53318355
VCA0896	1,27681171	2,60065291	1,21607811	2,5377E-06	1,21607811	0,62307631	0,5332344
VCA0284	-1,017805	-2,9115089	-1,0929509	-2,841E-06	-1,0929509	-0,6228363	0,53339212
VC2768	1,25318955	94,9075245	1,13525476	9,2612E-05	1,13525476	0,62279598	0,53341864
tRNA-Gly-4	1,29331502	1,34613026	1,21225408	1,3136E-06	1,21225408	0,6224668	0,53363499
VCA0018	-1,4533131	-0,5721785	-1,5819313	-5,583E-07	-1,5819313	-0,6219997	0,53394206
VC2032	-1,1887589	-1,5637643	-1,2441195	-1,526E-06	-1,2441195	-0,6216794	0,53415273
phhA	1,5598716	1,45378677	1,31511896	1,4186E-06	1,31511896	0,6208533	0,53469618
VC0896	1,32484631	1,35147065	1,210105	1,3188E-06	1,210105	0,62083643	0,53470727
thiE	1,42979668	0,99143401	1,29671683	9,6745E-07	1,29671683	0,61988443	0,5353339
VC1580	1,36206566	1,30926425	1,21685736	1,2776E-06	1,21685736	0,61986044	0,53534969
VC1517	-1,1407004	-12,717296	-1,2003224	-1,241E-05	-1,2003224	-0,6190048	0,53591327
VC1927	1,63661171	0,99894342	1,52484074	9,7478E-07	1,52484074	0,61784992	0,5366743
VCA0110	1,42519633	1,08129251	1,26672002	1,0551E-06	1,26672002	0,61782016	0,53669391
VC1628	1,2422471	4,57581214	1,23527355	4,4651E-06	1,23527355	0,61779054	0,53671346

VC0568	1,24815924	4,73350063	1,14464648	4,619E-06	1,14464648	0,61713781	0,53714386
VC0393	-1,0464866	-2,2003124	-1,1218508	-2,147E-06	-1,1218508	-0,6156923	0,53809765
VC0330	-1,0966935	-14,433266	-1,1379046	-1,408E-05	-1,1379046	-0,6152411	0,53839553
VCA0092	1,20750168	2,16191101	1,12356165	2,1096E-06	1,12356165	0,61431751	0,53900555
VC1147	-1,1102467	-8,1666361	-1,1919002	-7,969E-06	-1,1919002	-0,6140454	0,53918539
VC1993	1,79610672	1,46339317	1,47494247	1,428E-06	1,47494247	0,61395759	0,53924339
VC2211	1,33265354	1,29541715	1,21481897	1,2641E-06	1,21481897	0,61395151	0,53924739
VC0426	-1,0535681	-2,6084213	-1,1129196	-2,545E-06	-1,1129196	-0,6133229	0,53966285
VCA0561	1,1574507	5,52330301	1,10895898	5,3897E-06	1,10895898	0,61318759	0,53975233
tRNA-Gln-5	-1,362288	-2,0578499	-1,367443	-2,008E-06	-1,367443	-0,613032	0,53985524
VC2540	1,16806155	7,44710238	1,15276391	7,2669E-06	1,15276391	0,61156156	0,54082791
VC0615	-1,8054796	-7,8582915	-1,8681353	-7,668E-06	-1,8681353	-0,6114542	0,54089893
VC2168	1,20395461	2,19365158	1,12042078	2,1406E-06	1,12042078	0,61134765	0,54096948
fliQ	-1,1039415	-5,946923	-1,138148	-5,803E-06	-1,138148	-0,6113332	0,54097902
VC0237	1,07109307	15,7638149	1,12989948	1,5382E-05	1,12989948	0,60961646	0,54211593
VC1789	-1,3675974	-9,0009295	-1,3484981	-8,783E-06	-1,3484981	-0,6094918	0,54219855
VC1884	1,21743558	1,81453914	1,14641199	1,7706E-06	1,14641199	0,60936627	0,54228172
VCA0809	-1,111839	-1,7766769	-1,2036005	-1,734E-06	-1,2036005	-0,6086883	0,54273112
VC0397	-1,0340803	-73,743903	-1,2094584	-7,196E-05	-1,2094584	-0,6084234	0,54290671
VC2142	-1,2127956	-37,826052	-1,23335	-3,691E-05	-1,23335	-0,608081	0,54313381
VC1840	1,13223889	6,68754376	1,10597788	6,5258E-06	1,10597788	0,60796826	0,54320855
VC1411	1,25255436	1,54073911	1,17340103	1,5035E-06	1,17340103	0,60727184	0,54367055
tRNA-Glu-4	-1,5665082	-0,4679144	-1,7106368	-4,566E-07	-1,7106368	-0,6066416	0,54408879
VCA0628	1,45189794	42,7485136	1,40438421	4,1714E-05	1,40438421	0,6064974	0,54418454
VC0269	1,15581029	13,9943424	1,16725522	1,3656E-05	1,16725522	0,60571444	0,54470442
VC2202	1,01366272	6,52188176	1,0381431	6,3641E-06	1,0381431	0,60516623	0,54506857
VC1204	1,88248807	2,45094356	1,56706886	2,3917E-06	1,56706886	0,60498788	0,54518708
VC0266	-1,0607982	-1,615978	-1,1735741	-1,577E-06	-1,1735741	-0,6036552	0,54607294
VC2153	1,17610292	2,99268064	1,11052984	2,9203E-06	1,11052984	0,60341019	0,54623587
VCA1088	-1,1325197	-1,4118358	-1,1880727	-1,378E-06	-1,1880727	-0,6033738	0,54626005
VCA1057	1,52872411	0,69571303	1,42229826	6,7888E-07	1,42229826	0,60321478	0,54636585
mltA	1,16179333	2,99414879	1,10067938	2,9217E-06	1,10067938	0,60277086	0,54666116
VC2682	-1,1196691	-7,7428327	-1,1427969	-7,556E-06	-1,1427969	-0,6025787	0,54678902
VC2686	1,16284617	39,3153959	1,18585162	3,8364E-05	1,18585162	0,60229951	0,54697482
VC2397	-1,0796693	-33,21547	-1,119285	-3,241E-05	-1,119285	-0,6013377	0,54761511
avtA	1,15807884	2,81192436	1,08947535	2,7439E-06	1,08947535	0,60103878	0,54781417
argS	1,22902475	6,4055314	1,20388643	6,2506E-06	1,20388643	0,60087582	0,54792273
VC2666	1,32855956	1,28267569	1,20685059	1,2516E-06	1,20685059	0,60056835	0,54812755
rluD	-1,1203349	-11,273976	-1,1370433	-1,1E-05	-1,1370433	-0,6000236	0,54849051
VC1258	-1,1170963	-21,800976	-1,1350196	-2,127E-05	-1,1350196	-0,5998835	0,54858392
VCA0329b	1,21628176	1,59530201	1,16198084	1,5567E-06	1,16198084	0,59881166	0,54929851
secE	1,12703791	29,874403	1,11529366	2,9152E-05	1,11529366	0,59822493	0,54968988
VC2320	-1,1210684	-1,5124885	-1,1711934	-1,476E-06	-1,1711934	-0,5981402	0,54974639
VCA0041	1,16675116	3,16801861	1,10640274	3,0914E-06	1,10640274	0,59728032	0,55032026
VC1209	1,13873732	34,1722521	1,13394244	3,3346E-05	1,13394244	0,59605508	0,55113845

VC0948	1,09354056	9,17842532	1,08396543	8,9564E-06	1,08396543	0,59580454	0,55130584
VC1671	1,1818037	2,49630014	1,09938282	2,4359E-06	1,09938282	0,59542143	0,55156182
VC1080	1,388928	7,54656839	1,2968503	7,364E-06	1,2968503	0,59476158	0,55200289
VC2338	-1,2228592	-36,674807	-1,2998942	-3,579E-05	-1,2998942	-0,5944338	0,55222204
VC0579	1,07548814	6,73716889	1,06761115	6,5742E-06	1,06761115	0,59440918	0,55223849
VCA0689	1,3612446	1,12691497	1,23314002	1,0997E-06	1,23314002	0,59409759	0,55244686
VC1562	1,36765613	1,13372787	1,23134111	1,1063E-06	1,23134111	0,59382658	0,55262813
VC0423	1,52058666	4,30292179	1,31528667	4,1988E-06	1,31528667	0,59357067	0,55279934
VCA0991	1,38222571	1,12062943	1,2336246	1,0935E-06	1,2336246	0,59298947	0,55318822
VC2600	-1,0798467	-1,8277404	-1,1370096	-1,784E-06	-1,1370096	-0,5929159	0,55323742
VC2147	-1,1002369	-5,3700045	-1,1459118	-5,24E-06	-1,1459118	-0,5927913	0,55332086
VC2434	-1,3545492	-24,94138	-1,2620673	-2,434E-05	-1,2620673	-0,5926008	0,55344835
VC0658	-1,025515	-2,210223	-1,1170244	-2,157E-06	-1,1170244	-0,5918663	0,55394015
VC0077	-1,1242539	-1,0058885	-1,2624657	-9,816E-07	-1,2624657	-0,591673	0,55406959
VC0153	1,20508247	2,12747548	1,11595107	2,076E-06	1,11595107	0,59140039	0,5542522
tRNA-Asp-4	1,40504182	34,2308637	1,47074696	3,3403E-05	1,47074696	0,5908691	0,55460818
VC1759	1,1728359	2,47763041	1,09839033	2,4177E-06	1,09839033	0,59036087	0,55494876
VC0213	-1,0507523	-4,0472848	-1,103292	-3,949E-06	-1,103292	-0,588409	0,55625785
VC2305	1,16279936	14,3466534	1,17072105	1,4E-05	1,17072105	0,58800344	0,55653001
VC1910	-1,0793426	-8,2102462	-1,0632796	-8,012E-06	-1,0632796	-0,5874978	0,55686947
tRNA-Phe-1	1,74614453	0,51166179	1,6030737	4,9928E-07	1,6030737	0,58697313	0,55722178
tRNA-Asn-3	-1,3116152	-27,061044	-1,2757303	-2,641E-05	-1,2757303	-0,5866951	0,55740855
VC0627	1,24943137	15,9688741	1,21230695	1,5583E-05	1,21230695	0,58655982	0,55749941
VC0936	1,38597254	0,98123395	1,26446578	9,575E-07	1,26446578	0,58634021	0,55764694
VC1158	-1,0672944	-8,6564556	-1,0828486	-8,447E-06	-1,0828486	-0,5860595	0,55783557
VCA0849	1,2819561	1,2712826	1,19776901	1,2405E-06	1,19776901	0,58582957	0,55799008
fliN	-1,1083118	-10,225457	-1,1087793	-9,978E-06	-1,1087793	-0,585522	0,55819684
VC1633	1,19292507	7,97365557	1,17022575	7,7808E-06	1,17022575	0,58471359	0,55874034
VC0118	-1,0910413	-3,7793964	-1,1858058	-3,688E-06	-1,1858058	-0,5846595	0,55877671
VCA0217	1,3679301	1,21573303	1,2271159	1,1863E-06	1,2271159	0,58453936	0,55885751
VCA0311	-1,0953218	-10,345928	-1,1085414	-1,01E-05	-1,1085414	-0,5838988	0,55928843
23Sg	-1,1201222	-189,39501	-1,1500032	-0,0001848	-1,1500032	-0,5838328	0,55933285
VC1484	1,5815919	544,744678	1,77915494	0,00053157	1,77915494	0,58372476	0,55940554
VC1306	-1,0518618	-3,5713063	-1,1284755	-3,485E-06	-1,1284755	-0,5832435	0,55972945
VCA0527	1,1620425	2,53069296	1,09367564	2,4695E-06	1,09367564	0,58283415	0,56000498
VC2731	1,15301498	2,86412926	1,09242314	2,7948E-06	1,09242314	0,58255206	0,56019491
VCA1046	-1,1038787	-1,9565941	-1,1816052	-1,909E-06	-1,1816052	-0,5825417	0,56020185
VCA0011	-1,3165827	-31,661493	-1,3741844	-3,09E-05	-1,3741844	-0,5822617	0,56039043
VCA0075	1,22001741	2,39176437	1,18142056	2,3339E-06	1,18142056	0,58224955	0,56039862
VCA0067	1,39538315	1,11290038	1,2255772	1,086E-06	1,2255772	0,58172298	0,56075331
VCA0590	1,00045884	-2,5181219	-1,0935902	-2,457E-06	-1,0935902	-0,5811314	0,56115189
VC0813	1,31790775	3,23447006	1,18472316	3,1562E-06	1,18472316	0,58106493	0,56119672
VC2096	-1,0767657	-7,6486883	-1,0724584	-7,464E-06	-1,0724584	-0,579678	0,56213179
VC1079	1,19645281	3,91162193	1,15510611	3,817E-06	1,15510611	0,57946751	0,56227378
VC0227	1,13036061	11,030273	1,16754854	1,0763E-05	1,16754854	0,57921361	0,56244507

VCA0279	1,58886479	46,5528817	1,46331075	4,5427E-05	1,46331075	0,57913639	0,56249719
secA	1,16113771	16,8656904	1,13900047	1,6458E-05	1,13900047	0,5791195	0,56250858
VCA0494	1,33424937	1,59145089	1,23477612	1,553E-06	1,23477612	0,57894434	0,56262676
VC1408	1,17178015	2,11423117	1,11145543	2,0631E-06	1,11145543	0,57862978	0,56283903
VC2440	1,13178339	20,8661203	1,16115777	2,0361E-05	1,16115777	0,57850136	0,56292571
VCA0382	-1,1131552	-8,4261441	-1,1979569	-8,222E-06	-1,1979569	-0,5777155	0,56345621
thiP	1,39886928	0,78051486	1,33241086	7,6163E-07	1,33241086	0,5776794	0,56348061
VC2602	1,00356573	-21,753159	-1,0568684	-2,123E-05	-1,0568684	-0,5774685	0,56362304
VCA0156	-1,2012126	-0,8143317	-1,315991	-7,946E-07	-1,315991	-0,5773389	0,56371054
VC2324	1,29413778	1,24753184	1,1953723	1,2174E-06	1,1953723	0,57711897	0,56385911
VC2767	1,22769103	79,5756788	1,11009792	7,7651E-05	1,11009792	0,57684549	0,56404387
ileS	1,1337233	7,51538292	1,12081054	7,3336E-06	1,12081054	0,57677704	0,56409011
VCA0286	1,14969228	3,16100107	1,08922692	3,0845E-06	1,08922692	0,57674175	0,56411395
mglC	-1,2220067	-51,628852	-1,317428	-5,038E-05	-1,317428	-0,5765053	0,56427373
VC1257	1,13705113	3,67738088	1,09667678	3,5884E-06	1,09667678	0,57647475	0,56429436
VC0538	1,52972657	13,9331687	1,58795482	1,3596E-05	1,58795482	0,57630271	0,56441062
VC1048	1,18305226	6,9477762	1,16659476	6,7797E-06	1,16659476	0,57618488	0,56449025
gltD (NC_002505 2535637..2537147)	1,67957073	4,65450722	1,60576236	4,5419E-06	1,60576236	0,57508352	0,56523484
VC2502	1,29170871	1,34522594	1,17815694	1,3127E-06	1,17815694	0,5745354	0,56560558
VCA0919	1,06704941	18,6036376	1,09445649	1,8154E-05	1,09445649	0,57379443	0,56610695
ligA	1,19377432	2,13058882	1,11620547	2,079E-06	1,11620547	0,57352819	0,56628714
VCA0878	-1,0584873	-1,9324224	-1,1201976	-1,886E-06	-1,1201976	-0,5732909	0,56644777
VC2009	1,3218797	34,696495	1,25697533	3,3857E-05	1,25697533	0,57298365	0,5666558
VC2672	1,07799631	13,3205156	1,05003076	1,2998E-05	1,05003076	0,57276542	0,56680355
VCA0609	-1,1306723	-18,609773	-1,1712751	-1,816E-05	-1,1712751	-0,5727475	0,56681573
VC0002	1,05679392	6,81630451	1,06213445	6,6514E-06	1,06213445	0,57232137	0,5671043
nudC	1,24086601	1,52344753	1,15438522	1,4866E-06	1,15438522	0,57229219	0,56712406
VCA0341	1,20050246	1,54709291	1,16365872	1,5097E-06	1,16365872	0,57153562	0,56763664
VCA0003	-1,2843816	-12,173121	-1,257285	-1,188E-05	-1,257285	-0,5711845	0,56787465
VC1652	1,17679687	1,78585737	1,1295183	1,7427E-06	1,1295183	0,57083686	0,56811025
VC0466	1,20270445	1,69413129	1,13699734	1,6531E-06	1,13699734	0,57080932	0,56812893
tRNA-Thr-1	1,51623312	1,15926166	1,45624891	1,1312E-06	1,45624891	0,5707943	0,56813911
VC0767	-1,2141375	-12,913163	-1,170568	-1,26E-05	-1,170568	-0,5707722	0,56815408
VCA1080	1,16476326	2,44914851	1,09275525	2,3899E-06	1,09275525	0,57066875	0,56822422
VCA0936	1,27016949	1,33673449	1,17675307	1,3044E-06	1,17675307	0,57064216	0,56824226
VC1579	1,24774191	116,193449	1,13311684	0,00011338	1,13311684	0,57026695	0,56849668
VC0619	-1,4535211	-2,6814108	-1,5387015	-2,617E-06	-1,5387015	-0,5698126	0,56880484
VCA0469	1,14855431	4,25180426	1,09041328	4,1489E-06	1,09041328	0,56894014	0,56939679
VCA0624	1,14609956	9,58904211	1,14600925	9,3571E-06	1,14600925	0,56887585	0,56944042
VCA0799	1,16346929	2,90347569	1,07708657	2,8332E-06	1,07708657	0,56857805	0,56964255
VC2232	-1,0155605	-2,801071	-1,0798959	-2,733E-06	-1,0798959	-0,5681617	0,56992521
VC1658	1,29898847	1,21182648	1,19469677	1,1825E-06	1,19469677	0,56790333	0,57010063
tdh	-1,2264908	-16,694185	-1,1862497	-1,629E-05	-1,1862497	-0,5675365	0,57034978
VCA0651	-1,237308	-0,5107433	-1,5320007	-4,984E-07	-1,5320007	-0,5673962	0,57044503
VC1662	1,18357458	1,95319431	1,11617277	1,9059E-06	1,11617277	0,56717064	0,57059828

VC0605	1,13723056	3,3425363	1,09710166	3,2617E-06	1,09710166	0,56694345	0,57075262
emrD	-1,0609434	-1,7707587	-1,1283868	-1,728E-06	-1,1283868	-0,5660808	0,57133889
VC0475	1,48818809	2,98705977	1,40048231	2,9148E-06	1,40048231	0,56569053	0,5716042
VC2606	1,25174487	2,70709497	1,14310558	2,6416E-06	1,14310558	0,56485553	0,57217205
VC0624	1,19660778	2,06461132	1,1083383	2,0147E-06	1,1083383	0,5641634	0,57264295
VC0297	1,10709335	11,9260126	1,1173163	1,1638E-05	1,1173163	0,56369755	0,57296002
VCA0441	1,21179941	8,15679558	1,16868751	7,9595E-06	1,16868751	0,56350738	0,57308947
VC0113	1,17705726	2,44508507	1,14433072	2,3859E-06	1,14433072	0,56239725	0,57384542
VC2541	-1,0752451	-1,4674614	-1,1539683	-1,432E-06	-1,1539683	-0,5609732	0,57481582
VC1942	1,18263987	1,6918734	1,13166767	1,6509E-06	1,13166767	0,5599217	0,57553286
VCA0522	-1,1404246	-8,9942266	-1,1331519	-8,777E-06	-1,1331519	-0,5588413	0,57627004
VCA0436	1,23102499	3,34812624	1,14208856	3,2671E-06	1,14208856	0,55881491	0,57628807
VCA0695	1,34943753	1,51433237	1,24318003	1,4777E-06	1,24318003	0,55876739	0,57632051
VC0061	1,34673976	1,06366879	1,21621489	1,0379E-06	1,21621489	0,55795846	0,57687277
tRNA-Val-1	-1,1327306	-3,9666141	-1,1772475	-3,871E-06	-1,1772475	-0,5578127	0,5769723
VC2390	-1,1704343	-5,8494846	-1,170391	-5,708E-06	-1,170391	-0,5575081	0,57718034
VCA0831	1,18235822	2,07821192	1,10461911	2,0279E-06	1,10461911	0,55670965	0,57772585
VCA0787	-1,1346154	-1,4674874	-1,2762437	-1,432E-06	-1,2762437	-0,5561833	0,57808556
VC2519	1,15451581	2,0615414	1,11427834	2,0117E-06	1,11427834	0,55617951	0,57808817
nhaB	-1,0776107	-8,8113497	-1,1171034	-8,598E-06	-1,1171034	-0,5560474	0,57817849
VC0540	1,4074404	4,41171047	1,38586224	4,305E-06	1,38586224	0,55578942	0,57835485
VC0376	-1,0907593	-1,3695006	-1,1694012	-1,336E-06	-1,1694012	-0,5555739	0,57850221
VC1673	-1,0763716	-1,2224255	-1,1835738	-1,193E-06	-1,1835738	-0,555258	0,57871826
VC1786	-1,1265029	-0,9613958	-1,2393389	-9,381E-07	-1,2393389	-0,5552127	0,57874917
VC2417	1,1635408	2,60056246	1,08890071	2,5377E-06	1,08890071	0,55465874	0,57912812
VC0050	1,33084666	10,9283287	1,25246064	1,0664E-05	1,25246064	0,55431018	0,57936661
VCA0348	1,16037743	2,55379802	1,11438959	2,492E-06	1,11438959	0,55416096	0,57946872
VC2717	-1,2950657	-48,142576	-1,3287756	-4,698E-05	-1,3287756	-0,5538174	0,57970381
VCA0005	1,16514006	29,692961	1,19687276	2,8975E-05	1,19687276	0,55291887	0,580319
VC2669	1,36165998	2,58317356	1,23569859	2,5207E-06	1,23569859	0,5523793	0,58068854
VCA0570	-1,0098888	-38,522112	-1,1058416	-3,759E-05	-1,1058416	-0,5522742	0,58076057
VCA1070	1,36424029	40,1145983	1,30677563	3,9144E-05	1,30677563	0,55203979	0,58092112
VCA0793	1,21525327	1,52717459	1,14162271	1,4902E-06	1,14162271	0,55043039	0,58202423
VCA0904	1,54832664	2,44645499	1,29598585	2,3873E-06	1,29598585	0,54987994	0,58240175
VC2642	1,32352934	1,03014057	1,21656235	1,0052E-06	1,21656235	0,54949216	0,58266776
VC0278	-1,2179802	-14,54602	-1,2804241	-1,419E-05	-1,2804241	-0,5491789	0,5828827
tRNA-Pro-1	-1,3251637	-40,103168	-1,2926093	-3,913E-05	-1,2926093	-0,5488311	0,58312137
VCA0646	1,41997534	0,70714125	1,33092174	6,9003E-07	1,33092174	0,54879867	0,58314364
23Sa	-1,4494092	-27,810457	-1,5082188	-2,714E-05	-1,5082188	-0,5482038	0,58355198
VC2226	-1,1871329	-5,0587875	-1,2064249	-4,936E-06	-1,2064249	-0,5477202	0,58388403
VC1348	-1,0920604	-1,1349928	-1,1930062	-1,108E-06	-1,1930062	-0,5474246	0,58408711
VCA0370	1,20889023	1,50403436	1,14222641	1,4676E-06	1,14222641	0,54733013	0,58415197
VC2753	1,79631176	146,398115	1,5651558	0,00014286	1,5651558	0,54711117	0,5843024
trmB	1,2301016	5,04402027	1,19386647	4,922E-06	1,19386647	0,54695701	0,5844083
VCA0128	-1,1701954	-4,589578	-1,4332214	-4,479E-06	-1,4332214	-0,5468619	0,58447363

VC0364	1,26903579	4,72617609	1,20019369	4,6118E-06	1,20019369	0,54650805	0,58471678
nusA	1,1300124	23,8427666	1,1579937	2,3266E-05	1,1579937	0,54562588	0,58532317
VC1977	1,20453095	1,79383738	1,11687648	1,7504E-06	1,11687648	0,54509402	0,58568887
rbgA	1,05366296	4,74217128	1,05848619	4,6275E-06	1,05848619	0,54493394	0,58579897
VC0705	1,1701738	10,9461961	1,15611726	1,0681E-05	1,15611726	0,54445583	0,58612786
VC0273	1,09497718	370,174688	1,20890868	0,00036122	1,20890868	0,54380178	0,58657791
VC0774	2,05822384	0,40286242	1,64226763	3,9312E-07	1,64226763	0,54201189	0,58781034
VC0080	-1,0852563	-1,4197542	-1,1478613	-1,385E-06	-1,1478613	-0,5414939	0,58816722
VC2535	1,09856399	5,10976003	1,06579364	4,9861E-06	1,06579364	0,54110514	0,58843514
VCA0981	1,34369173	1,07223127	1,19859808	1,0463E-06	1,19859808	0,53903968	0,5898595
VCA0439	-1,1270911	-10,142853	-1,1824361	-9,897E-06	-1,1824361	-0,5387233	0,59007782
VC0384	1,56558273	7,04029261	1,63958937	6,87E-06	1,63958937	0,5380933	0,59051267
VCA0614	1,24328001	1,18976407	1,19150295	1,161E-06	1,19150295	0,53808012	0,59052175
panB	1,15062132	5,99147138	1,12817539	5,8465E-06	1,12817539	0,53805597	0,59053843
VCA0587	-1,0849243	-1,6875311	-1,1429538	-1,647E-06	-1,1429538	-0,5376862	0,59079376
VCA0140	-1,0719926	-1,3906039	-1,1483294	-1,357E-06	-1,1483294	-0,5366952	0,59147817
VC0379a	-1,1414477	-1,4887753	-1,1823341	-1,453E-06	-1,1823341	-0,5361067	0,5918848
VCA0371	-1,3228743	-7,8554522	-1,2999369	-7,665E-06	-1,2999369	-0,5357134	0,5921567
VC2014	1,2065325	1,52134525	1,1341064	1,4845E-06	1,1341064	0,53554234	0,59227491
VC0580	1,08569297	6,07699399	1,07289096	5,93E-06	1,07289096	0,53506302	0,59260631
VC1984	1,28552619	3,68039889	1,16061365	3,5914E-06	1,16061365	0,53492848	0,59269934
rnhB	1,21256039	1,27383027	1,16172665	1,243E-06	1,16172665	0,53469809	0,59285866
VC2739	-1,0117701	-1,8992795	-1,1055821	-1,853E-06	-1,1055821	-0,5345251	0,5929783
VC2757	-1,0170002	-1,9539643	-1,1018956	-1,907E-06	-1,1018956	-0,5330837	0,59397568
VCA0848	-1,0908338	-1,496053	-1,1349293	-1,46E-06	-1,1349293	-0,5325962	0,59431316
VCA0519	-1,5102621	-34,639835	-1,463899	-3,38E-05	-1,463899	-0,531771	0,59488466
VC2141	-1,0847912	-65,502033	-1,1966816	-6,392E-05	-1,1966816	-0,5312105	0,59527295
VC1601	-1,0745072	-6,849042	-1,1332898	-6,683E-06	-1,1332898	-0,5306053	0,59569234
VC1164	1,34555378	0,99484797	1,22457351	9,7078E-07	1,22457351	0,53006479	0,59606703
VC0716	1,16306066	4,33733638	1,13380104	4,2324E-06	1,13380104	0,52993992	0,59615361
VC1533	-1,0788778	-1,134761	-1,1795801	-1,107E-06	-1,1795801	-0,5296109	0,59638172
VC1066	-1,0876522	-7,2756583	-1,1652283	-7,1E-06	-1,1652283	-0,529405	0,59652457
VC0455	1,14731495	1,97170833	1,09933795	1,924E-06	1,09933795	0,52905741	0,59676564
VC0769	-1,2011315	-2,3209836	-1,3131253	-2,265E-06	-1,3131253	-0,5289705	0,59682596
VCA0970	1,18288973	8,26621735	1,11527206	8,0662E-06	1,11527206	0,52871579	0,59700264
VCA0805	1,15092149	2,52572391	1,08576661	2,4646E-06	1,08576661	0,52798825	0,59750751
VC2112	1,19010198	2,86640448	1,12859132	2,7971E-06	1,12859132	0,52737301	0,59793461
VC2002	-1,0158596	-2,3423883	-1,0821862	-2,286E-06	-1,0821862	-0,5266669	0,59842496
VC0251	1,20753357	76,5079864	1,15680813	7,4657E-05	1,15680813	0,52664421	0,59844072
hutH	-1,1232138	-1,7162431	-1,2681243	-1,675E-06	-1,2681243	-0,5263943	0,59861431
VCA0144	-1,0705729	-1,7198521	-1,169803	-1,678E-06	-1,169803	-0,5253858	0,59931505
VC1402	1,36594278	0,87118914	1,23599181	8,5011E-07	1,23599181	0,52520772	0,59943882
VCA0837	-1,021629	-2,2805429	-1,083639	-2,225E-06	-1,083639	-0,5240574	0,60023865
VC1949	-1,2044104	-0,7854702	-1,2631093	-7,665E-07	-1,2631093	-0,5234094	0,60068941
gltX	1,11424893	9,23377745	1,14255564	9,0104E-06	1,14255564	0,52330218	0,60076402

VC0503	-1,0617672	-3,6501827	-1,0943	-3,562E-06	-1,0943	-0,5228937	0,60104824
VC2438	-1,026409	-1,9987909	-1,1078891	-1,95E-06	-1,1078891	-0,5228562	0,60107432
VC2335	1,0080738	-2,3570886	-1,0803359	-2,3E-06	-1,0803359	-0,5225685	0,60127459
VC1631	-1,1850151	-4,5621294	-1,2639016	-4,452E-06	-1,2639016	-0,5220297	0,60164968
VCA0473	1,20194344	2,06207888	1,09354519	2,0122E-06	1,09354519	0,52193318	0,60171688
rlmL	1,14052016	3,14771739	1,09668259	3,0716E-06	1,09668259	0,5219224	0,60172438
VC1956	1,09971207	4,83550867	1,06099299	4,7185E-06	1,06099299	0,52163533	0,60192428
VC2683	1,32427236	1,02176044	1,19467989	9,9704E-07	1,19467989	0,52144842	0,60205445
VCA0822	-1,1411483	-1,1795563	-1,1990913	-1,151E-06	-1,1990913	-0,5209172	0,6024245
VC0371	1,12029557	3,00071321	1,06210971	2,9281E-06	1,06210971	0,52072366	0,60255931
VC1973	-1,1997116	-56,804449	-1,2185553	-5,543E-05	-1,2185553	-0,5204018	0,60278356
VCA0738	-1,0775975	-1,5486753	-1,1235015	-1,511E-06	-1,1235015	-0,5198205	0,60318869
VC0934	1,75763975	0,40683695	1,56811638	3,97E-07	1,56811638	0,51961578	0,60333141
VC2301	-1,2372465	-18,193329	-1,2386661	-1,775E-05	-1,2386661	-0,5193096	0,60354487
VCA0931	1,28813167	1,08407829	1,18048993	1,0579E-06	1,18048993	0,51884996	0,60386539
VC1106	1,1626645	1,56997958	1,12086777	1,532E-06	1,12086777	0,51809454	0,60439233
VCA0339	1,1928937	1,63551102	1,11561455	1,5959E-06	1,11561455	0,51781964	0,60458413
VC1108	1,1667224	2,64547629	1,10600658	2,5815E-06	1,10600658	0,51758769	0,604746
VC0163	1,14751758	9,42643721	1,17162828	9,1984E-06	1,17162828	0,51677375	0,60531414
VCA0768	1,24958741	6,8932312	1,18072934	6,7265E-06	1,18072934	0,5166425	0,60540577
VC1890	1,25882079	9,10614131	1,26802908	8,8859E-06	1,26802908	0,51660215	0,60543395
VC0609	1,26144055	1,54850742	1,17657216	1,511E-06	1,17657216	0,51639645	0,60557757
VC1320	1,36567341	162,078762	1,29688027	0,00015816	1,29688027	0,51631458	0,60563474
mazG	1,09027279	3,80499191	1,07294656	3,7129E-06	1,07294656	0,51626885	0,60566667
VC1998	1,08429895	4,18733752	1,08185109	4,086E-06	1,08185109	0,51592958	0,60590362
VC1224	1,22377162	11,0100574	1,15988255	1,0744E-05	1,15988255	0,51579582	0,60599705
VC2101	-1,5375376	-4,6877914	-1,4807288	-4,574E-06	-1,4807288	-0,5153319	0,60632114
VCA0952	-1,1027853	-5,3547005	-1,129132	-5,225E-06	-1,129132	-0,5153159	0,60633232
VCA0671	1,23927459	1,26484944	1,15031334	1,2343E-06	1,15031334	0,51502684	0,60653428
VC2523	1,16957652	2,4745798	1,10786588	2,4147E-06	1,10786588	0,51497013	0,60657392
VC0662	1,2827213	3,17317085	1,23296572	3,0964E-06	1,23296572	0,51418159	0,60712507
VCA0591	-1,0017911	-3,4729749	-1,0847347	-3,389E-06	-1,0847347	-0,5141502	0,60714699
VC0517	-1,0516966	-8,2059159	-1,0365453	-8,007E-06	-1,0365453	-0,5134004	0,60767127
VCA0978	1,23233897	1,3228566	1,14220545	1,2909E-06	1,14220545	0,51327095	0,60776182
VC1280	1,32633508	1,61040319	1,17294117	1,5714E-06	1,17294117	0,51284447	0,60806014
VC0597	1,38199735	0,90121916	1,21549946	8,7942E-07	1,21549946	0,51281898	0,60807797
thiH	1,27437258	1,09964056	1,17297753	1,073E-06	1,17297753	0,51245378	0,60833348
VC0690	-1,0388205	-3,8158669	-1,1282096	-3,724E-06	-1,1282096	-0,5123579	0,60840061
VC0904	1,22890546	1,29739813	1,14407508	1,266E-06	1,14407508	0,51141535	0,60906027
VCA0846	1,17513479	2,01491315	1,09041763	1,9662E-06	1,09041763	0,51133294	0,60911796
VC2311	1,27132791	2,1243374	1,19793871	2,0729E-06	1,19793871	0,5111834	0,60922267
VC0626	1,15742164	4,8997408	1,11803161	4,7812E-06	1,11803161	0,51107866	0,609296
VC1022	1,14868634	2,3777124	1,07594391	2,3202E-06	1,07594391	0,51084111	0,60946234
VC1530	-1,0786059	-1,5539095	-1,1615777	-1,516E-06	-1,1615777	-0,5100558	0,61001239
VC1398	1,36558798	0,63312767	1,31729641	6,1781E-07	1,31729641	0,50997461	0,61006927

VCA0424	1,16256855	2,50259013	1,09929144	2,442E-06	1,09929144	0,50986173	0,61014835
VCA0291	1,17724094	2,25683308	1,11534723	2,2022E-06	1,11534723	0,50985798	0,61015098
VCA0176	-1,1250795	-7,4781873	-1,1921762	-7,297E-06	-1,1921762	-0,5098353	0,61016691
lldD	-1,2046369	-2,3590684	-1,3566461	-2,302E-06	-1,3566461	-0,5096195	0,61031806
VC1240	-1,1124016	-1,9182758	-1,1833171	-1,872E-06	-1,1833171	-0,5090385	0,61072524
VC0405	-1,0360651	-3,2700889	-1,0542326	-3,191E-06	-1,0542326	-0,5089311	0,61080055
VC0923	1,20768794	1,49980624	1,1220962	1,4635E-06	1,1220962	0,50880283	0,61089046
VCA0925	1,16453773	1,8716331	1,0965926	1,8264E-06	1,0965926	0,5086162	0,61102128
VC1193	-1,2691569	-11,724329	-1,3385392	-1,144E-05	-1,3385392	-0,5085468	0,61106997
VC2350	-1,2317285	-24,460645	-1,2389021	-2,387E-05	-1,2389021	-0,5084772	0,61111877
VC1088	-1,0567391	-1,1178402	-1,1665311	-1,091E-06	-1,1665311	-0,5077118	0,61165547
VCA0202	-1,2741536	-0,5069238	-1,4062658	-4,947E-07	-1,4062658	-0,5067172	0,61235329
VC0830	1,30224159	3,15543055	1,26219033	3,0791E-06	1,26219033	0,5065738	0,61245392
VCA0035	1,25301892	1,15576745	1,1598328	1,1278E-06	1,1598328	0,50654777	0,61247218
VCA1053	1,22213212	1,38486297	1,13164682	1,3514E-06	1,13164682	0,50654027	0,61247744
VC1061	1,42358877	0,69967773	1,27807962	6,8275E-07	1,27807962	0,50618632	0,61272588
argD	1,77439135	7,95439589	1,52528739	7,762E-06	1,52528739	0,50609658	0,61278887
VCA0115	1,42842272	0,7269339	1,26590217	7,0935E-07	1,26590217	0,50588187	0,6129396
VC0135	1,29736466	2,64579829	1,15511342	2,5818E-06	1,15511342	0,50489057	0,61363572
VC1495	-1,0888104	-0,9119341	-1,2052801	-8,899E-07	-1,2052801	-0,5046439	0,61380901
ihfB	1,03231446	-82,35587	-1,0897609	-8,036E-05	-1,0897609	-0,5043741	0,61399853
VC1762	-1,1855989	-9,5657936	-1,1558064	-9,334E-06	-1,1558064	-0,5035357	0,61458772
VC1929	1,72201663	2,64210564	1,663657	2,5782E-06	1,663657	0,50334723	0,6147202
sdhA	1,53790308	33,1196621	1,45974544	3,2318E-05	1,45974544	0,50315308	0,61485668
treR	1,1391213	2,86891596	1,08428318	2,7995E-06	1,08428318	0,50240625	0,6153818
VC0075	-1,1072995	-8,9976665	-1,1525175	-8,78E-06	-1,1525175	-0,5022296	0,61550607
VC0361	1,10013284	21,8852483	1,13721912	2,1356E-05	1,13721912	0,50218122	0,61554007
VC1893	-1,0375064	-3,1795216	-1,0542315	-3,103E-06	-1,0542315	-0,5018288	0,61578797
VC2240	1,37408557	14,184791	1,25188798	1,3842E-05	1,25188798	0,50139114	0,61609591
VC2761	1,45668041	7,31760938	1,47663518	7,1406E-06	1,47663518	0,50119005	0,61623741
VC2612	1,2328617	1,14308289	1,15753156	1,1154E-06	1,15753156	0,50038741	0,61680234
murG	-1,0354416	-2,2730776	-1,0916347	-2,218E-06	-1,0916347	-0,4991357	0,61768385
VCA0318	1,17452428	3,10058128	1,12109147	3,0256E-06	1,12109147	0,49905137	0,61774322
VC2108	1,19849529	3,985866	1,15720561	3,8894E-06	1,15720561	0,49895183	0,61781334
VC0071	1,12043466	8,64737184	1,10388584	8,4382E-06	1,10388584	0,49855263	0,61809461
VC0010	1,37409237	20,5382804	1,42702482	2,0041E-05	1,42702482	0,49835538	0,61823361
VC0747	1,12105023	5,28430606	1,10659989	5,1565E-06	1,10659989	0,49825264	0,61830601
VCA0083	1,25074688	1,08171894	1,16550745	1,0556E-06	1,16550745	0,49802172	0,61846875
VC1091	1,11928667	21,4104845	1,06816301	2,0893E-05	1,06816301	0,49798177	0,61849692
VCA0116	1,32876987	0,76608196	1,24141169	7,4755E-07	1,24141169	0,49752779	0,61881693
VC1127	1,20768092	2,63035845	1,15441942	2,5667E-06	1,15441942	0,49693363	0,61923588
VC2480	1,11708227	2,65650814	1,06393902	2,5922E-06	1,06393902	0,49688956	0,61926696
VC0004	1,10174688	10,6195105	1,08904069	1,0363E-05	1,08904069	0,49688301	0,61927157
VC0090	-1,0692174	-1,1193279	-1,1586149	-1,092E-06	-1,1586149	-0,4967356	0,61937553
VCA0881	1,42991135	0,71674245	1,25905808	6,994E-07	1,25905808	0,49656686	0,61949454

VCA0853	1,48191396	0,48249043	1,40965218	4,7082E-07	1,40965218	0,49606175	0,61985086
VCA0111	1,45731859	0,59357915	1,31947802	5,7922E-07	1,31947802	0,49525147	0,62042264
VC1232	1,1682379	1,50573214	1,11482681	1,4693E-06	1,11482681	0,49524679	0,62042594
rseB	1,0861122	16,8491395	1,06954948	1,6442E-05	1,06954948	0,49489356	0,62067529
VC1286	-1,0312093	-5,3655988	-1,1257055	-5,236E-06	-1,1257055	-0,4948719	0,62069061
VC1697	-1,0530242	-6,325184	-1,1860217	-6,172E-06	-1,1860217	-0,4946982	0,62081317
VCA0379	1,24641725	1,17924741	1,14856293	1,1507E-06	1,14856293	0,49459088	0,62088896
VC1008	-1,0558742	-8,1217489	-1,1012321	-7,925E-06	-1,1012321	-0,4939169	0,62136491
VCA0157	-1,0728208	-0,9515399	-1,1868378	-9,285E-07	-1,1868378	-0,493855	0,62140861
VC0076	-1,2102105	-74,924226	-1,2563315	-7,311E-05	-1,2563315	-0,493749	0,62148351
VCA0862	-1,0314268	-1,1444885	-1,1932749	-1,117E-06	-1,1932749	-0,4937361	0,62149257
VCA0454	1,3800653	1,72216792	1,37501486	1,6805E-06	1,37501486	0,493447	0,6216968
VCA0064	1,51313331	0,4819646	1,40485642	4,7031E-07	1,40485642	0,49337194	0,62174982
VCA0132	-1,065846	-2,4100069	-1,1494497	-2,352E-06	-1,1494497	-0,4933101	0,62179349
rph	-1,108632	-10,134042	-1,0982148	-9,889E-06	-1,0982148	-0,4933023	0,62179907
VCA0661	1,00610793	-2,4762664	-1,0675823	-2,416E-06	-1,0675823	-0,4927792	0,62216863
prfC	1,11843414	3,10413342	1,08854723	3,029E-06	1,08854723	0,49206023	0,62267678
tRNA-Glu-2	1,1771338	23,4658176	1,23670935	2,2898E-05	1,23670935	0,49180304	0,62285862
VC1447	1,38124071	0,61218761	1,30320483	5,9738E-07	1,30320483	0,49170562	0,62292748
VC1892	1,03324029	5,62156297	1,03771896	5,4856E-06	1,03771896	0,49145758	0,62310287
VC1950	1,21008879	15,9105588	1,23409564	1,5526E-05	1,23409564	0,49135698	0,62317401
VC1283	1,39933818	0,91789429	1,19588208	8,9569E-07	1,19588208	0,49049529	0,62378348
VC2264	-1,0732304	-6,6639259	-1,1044243	-6,503E-06	-1,1044243	-0,490442	0,62382121
VC0158	1,07052643	4,07726566	1,05673264	3,9786E-06	1,05673264	0,49011488	0,62405263
VC0073	-1,0457803	-1,9037631	-1,0915949	-1,858E-06	-1,0915949	-0,4899594	0,62416265
VC0152	1,14739936	2,27300725	1,07360522	2,218E-06	1,07360522	0,48981906	0,62426196
VCA0838	-1,043064	-3,4524946	-1,0785524	-3,369E-06	-1,0785524	-0,48923	0,62467887
VC1482	1,11765284	2,40365485	1,06839861	2,3455E-06	1,06839861	0,4883272	0,62531812
VC0063	1,31261756	0,71760383	1,24828624	7,0024E-07	1,24828624	0,48758936	0,62584075
VC1888	1,18626685	1,4939318	1,1119526	1,4578E-06	1,1119526	0,48742076	0,6259602
VC2239	1,11098036	5,35037889	1,0617512	5,2209E-06	1,0617512	0,48677721	0,62641625
VC1386	1,3678315	0,76756908	1,22931881	7,49E-07	1,22931881	0,48669166	0,62647688
VC1945	1,37010619	0,80033083	1,21829855	7,8097E-07	1,21829855	0,48608441	0,62690733
VCA0022	1,18479303	1,29823998	1,1426018	1,2668E-06	1,1426018	0,48606442	0,62692151
ihfA	1,02087108	-60,590614	-1,1257412	-5,912E-05	-1,1257412	-0,4856731	0,62719896
mobA	-1,0620104	-3,5290045	-1,1463366	-3,444E-06	-1,1463366	-0,4854995	0,62732213
VC0866	1,15479471	2,11823741	1,07703267	2,067E-06	1,07703267	0,48547829	0,62733713
VC2065	1,11771258	28,9559141	1,05882445	2,8255E-05	1,05882445	0,48505627	0,62763646
VC2431	-1,0090475	-2,8210589	-1,0767523	-2,753E-06	-1,0767523	-0,4844488	0,6280674
nusB	-1,1646506	-31,610923	-1,1516081	-3,085E-05	-1,1516081	-0,4840675	0,628338
VCA0644	-1,0351641	-0,8070621	-1,2136635	-7,875E-07	-1,2136635	-0,4834197	0,62879776
VC1242	1,32633768	11,6445588	1,22295494	1,1363E-05	1,22295494	0,4833257	0,62886453
moaA	1,14667264	2,20381048	1,09628272	2,1505E-06	1,09628272	0,48206383	0,62976062
VC1763	-1,217015	-12,110657	-1,1748442	-1,182E-05	-1,1748442	-0,4820293	0,62978515
VC2528	-1,0824164	-7,6991261	-1,1063014	-7,513E-06	-1,1063014	-0,4819629	0,62983232

holA	1,14511935	2,37844982	1,06720107	2,3209E-06	1,06720107	0,48162842	0,63006995
VC0173	1,2231402	1,99293694	1,1617035	1,9447E-06	1,1617035	0,48155138	0,63012469
VCA0533	1,18407749	6,55086887	1,1514961	6,3924E-06	1,1514961	0,48136219	0,63025913
VC1208	1,12717608	26,2853164	1,12409068	2,5649E-05	1,12409068	0,48086907	0,63060957
VCA0568	-1,0578275	-7,3940691	-1,0555538	-7,215E-06	-1,0555538	-0,4806148	0,63079032
tRNA-Gln-4	-1,2435659	-3,3099263	-1,2965371	-3,23E-06	-1,2965371	-0,4805882	0,63080925
VCA0503	1,14267654	1,91750001	1,08360791	1,8711E-06	1,08360791	0,48045096	0,63090678
VC1000	1,08232429	9,09445189	1,08772923	8,8744E-06	1,08772923	0,48020548	0,63108132
VC2640	1,08896908	5,35874195	1,06398447	5,2291E-06	1,06398447	0,47970999	0,63143364
rpmH	1,11194778	23,4743196	1,09482132	2,2906E-05	1,09482132	0,47938517	0,63166467
VC1346	1,26684568	1,04823405	1,15735214	1,0229E-06	1,15735214	0,47892455	0,63199232
VCA0270	1,00103855	-2,6766905	-1,1218253	-2,612E-06	-1,1218253	-0,478884	0,63202114
VC2513	1,15485666	1,95209489	1,08137607	1,9049E-06	1,08137607	0,47850828	0,6322885
VC1785	1,45150325	0,62201502	1,2788142	6,0697E-07	1,2788142	0,4778203	0,63277813
VCA1078	-1,0594311	-4,0730728	-1,0781192	-3,975E-06	-1,0781192	-0,4778111	0,63278469
pdxA	1,14214404	3,53129313	1,08346982	3,4459E-06	1,08346982	0,47674866	0,63354113
VC1805	1,29283708	0,95161659	1,17226635	9,286E-07	1,17226635	0,47581332	0,63420739
VC1974	-1,1323819	-9,1319445	-1,1448404	-8,911E-06	-1,1448404	-0,4746364	0,63504617
VCA0006	1,16227684	34,1153262	1,15695105	3,329E-05	1,15695105	0,4733252	0,63598121
VC0183	1,20552467	2,71997797	1,1405921	2,6542E-06	1,1405921	0,47314944	0,63610659
VC1121	-1,0153382	-1,540004	-1,122388	-1,503E-06	-1,122388	-0,4729453	0,6362522
VC0900	1,13483597	9,18065029	1,09212398	8,9586E-06	1,09212398	0,47294493	0,6362525
VC2554	1,27075037	1,04435826	1,15345242	1,0191E-06	1,15345242	0,47250474	0,63656658
VC2368	-1,0096791	-157,58987	-1,2105013	-0,0001538	-1,2105013	-0,471666	0,63716523
VC2187	-1,4074653	-198,89521	-1,3555544	-0,0001941	-1,3555544	-0,4715825	0,63722486
16Sb	1,26242606	5,97313521	1,20751806	5,8286E-06	1,20751806	0,47095496	0,63767292
fliE	-1,0804483	-17,158557	-1,0841568	-1,674E-05	-1,0841568	-0,4707357	0,63782951
VC1506	-1,0209033	-0,8922967	-1,1802824	-8,707E-07	-1,1802824	-0,4704752	0,63801558
VC0491	1,27860791	50,0320812	1,26759751	4,8822E-05	1,26759751	0,47029941	0,63814113
VCA0344	1,3966742	123,291547	1,31248344	0,00012031	1,31248344	0,47017893	0,63822721
VC1089	1,34865334	0,70369895	1,23360089	6,8668E-07	1,23360089	0,46988249	0,63843899
VC1733	1,62302553	0,43833587	1,40325043	4,2773E-07	1,40325043	0,46973435	0,63854484
VCA1041	-1,1618386	-10,673974	-1,2221435	-1,042E-05	-1,2221435	-0,4694514	0,63874702
VC1939	1,20446498	1,7769085	1,08991543	1,7339E-06	1,08991543	0,46801191	0,63977609
VC2610	1,17095052	1,6071785	1,09514698	1,5683E-06	1,09514698	0,46793625	0,63983019
rpsT	1,11460057	207,699508	1,16060898	0,00020267	1,16060898	0,46672347	0,64069776
VC1492	-1,2926123	-20,661779	-1,3106929	-2,016E-05	-1,3106929	-0,4666266	0,64076709
VC1708	1,28954578	0,83583798	1,1900582	8,1562E-07	1,1900582	0,46648554	0,64086801
VC2265	1,01003464	-2,1889658	-1,0683932	-2,136E-06	-1,0683932	-0,4659906	0,64122226
VCA0893	1,09771915	2,78491562	1,05303059	2,7175E-06	1,05303059	0,46456192	0,64224522
VC0415	-1,0625478	-10,555119	-1,0876126	-1,03E-05	-1,0876126	-0,4643594	0,64239032
VC2028	1,23069516	5,58847727	1,19655731	5,4533E-06	1,19655731	0,46426853	0,6424554
uvrD	1,14412757	2,01605449	1,073831	1,9673E-06	1,073831	0,46403471	0,64262289
VC1345	1,28443601	0,86682761	1,18041307	8,4586E-07	1,18041307	0,4638662	0,64274362
VC1702	1,28256816	3,33132513	1,1379815	3,2507E-06	1,1379815	0,46328969	0,64315676

VCA1002	-1,135609	-1,557575	-1,2209691	-1,52E-06	-1,2209691	-0,4630723	0,64331253
VCA0407	-1,2097606	-0,5664882	-1,2885679	-5,528E-07	-1,2885679	-0,4629123	0,64342726
VC1611	1,3938181	0,72670677	1,21731046	7,0913E-07	1,21731046	0,4622406	0,64390881
VCA0496	1,15213342	5,25559577	1,13578883	5,1285E-06	1,13578883	0,46169681	0,64429878
VC0130	-1,0561446	-3,8336709	-1,1550476	-3,741E-06	-1,1550476	-0,4616116	0,64435992
VC2424	-1,0811767	-1,0379315	-1,1467946	-1,013E-06	-1,1467946	-0,4614306	0,64448973
VCA0105	1,23054206	2,23375781	1,1198467	2,1797E-06	1,1198467	0,46139211	0,64451733
VC1986	1,15568491	1,49596817	1,09949302	1,4598E-06	1,09949302	0,46117339	0,64467423
tppB	1,25759946	7,55553077	1,26517437	7,3728E-06	1,26517437	0,46094898	0,64483524
VCA0154	-1,0697177	-0,8045033	-1,1919777	-7,85E-07	-1,1919777	-0,4597618	0,64568724
pyrB	1,16739715	2,75310055	1,13009572	2,6865E-06	1,13009572	0,4596076	0,64579792
VC1861	1,3664517	0,72422955	1,21499933	7,0671E-07	1,21499933	0,45923111	0,64606823
VC1900	-1,08331	-5,9197653	-1,0569694	-5,777E-06	-1,0569694	-0,4590401	0,64620536
VC0108	1,14559921	1,79726182	1,08123739	1,7538E-06	1,08123739	0,45876297	0,64640441
VC1796	1,29037282	1,33926092	1,21930901	1,3069E-06	1,21930901	0,45869232	0,64645515
VC2346	1,17371126	11,1984745	1,16774183	1,0928E-05	1,16774183	0,45838263	0,6466776
VC0647	1,06090561	14,2293467	1,07489612	1,3885E-05	1,07489612	0,4581263	0,64686172
VC1423	-1,0288591	-4,736064	-1,0476844	-4,621E-06	-1,0476844	-0,4574663	0,64733593
VC1400	-1,0811922	-1,0349879	-1,1439945	-1,01E-06	-1,1439945	-0,456658	0,64791692
VCA0834	-1,0421837	-1,3939249	-1,1048733	-1,36E-06	-1,1048733	-0,4564602	0,64805914
VC0635	-1,0013546	-60,811542	-1,1316949	-5,934E-05	-1,1316949	-0,4564119	0,64809383
VCA1052	-1,0128884	-0,931375	-1,1609901	-9,088E-07	-1,1609901	-0,4562442	0,64821437
VCA0759	1,58919304	5,83826401	1,47078592	5,697E-06	1,47078592	0,45545754	0,64878013
VC0600	1,46249387	0,50183005	1,31949076	4,8969E-07	1,31949076	0,45537814	0,64883723
VC0022	1,11835771	5,12705919	1,09855893	5,003E-06	1,09855893	0,4543707	0,64956206
VC1296	-1,0372641	-1,6271095	-1,0982283	-1,588E-06	-1,0982283	-0,4541599	0,64971374
VC1397	1,2501137	0,88541429	1,1679616	8,6399E-07	1,1679616	0,45364311	0,65008574
VC1748	1,31901491	0,6050863	1,25559534	5,9045E-07	1,25559534	0,45354014	0,65015986
tRNA-Asn-1	1,05935363	9,69132706	1,09410468	9,4569E-06	1,09410468	0,45320786	0,65039909
VC2451	-1,028409	-3,0541156	-1,0457058	-2,98E-06	-1,0457058	-0,4524616	0,65093652
VC2426	1,25862708	0,96077532	1,15282107	9,3753E-07	1,15282107	0,45233547	0,65102735
tRNA-Pro-3	-1,1212066	-5,0369084	-1,1316	-4,915E-06	-1,1316	-0,4521173	0,65118449
VCA0247	1,27755797	3,43858986	1,15526585	3,3554E-06	1,15526585	0,4514704	0,65165057
VC0917	1,44567651	0,46902031	1,33794773	4,5767E-07	1,33794773	0,45098715	0,65199883
VCA0357	1,20236039	1,89837274	1,13290336	1,8524E-06	1,13290336	0,45043687	0,65239548
VC1905	1,1120672	9,12301397	1,08871043	8,9023E-06	1,08871043	0,45032688	0,65247478
VCA0888	1,20599293	1,16212586	1,12302167	1,134E-06	1,12302167	0,44947231	0,65309099
VCA0453	1,19279469	1,15810114	1,1314655	1,1301E-06	1,1314655	0,44931289	0,65320598
VC1738	1,0860125	14,6531917	1,07397319	1,4299E-05	1,07397319	0,44905934	0,65338887
VC1558	-1,2078007	-17,65307	-1,2388349	-1,723E-05	-1,2388349	-0,4490061	0,65342727
VC0632	1,005515	-1,8967141	-1,0734374	-1,851E-06	-1,0734374	-0,4489317	0,65348094
VCA0681	1,21056139	1,1410708	1,12506825	1,1135E-06	1,12506825	0,44885501	0,65353627
recX	-1,0513138	-0,9384481	-1,1540727	-9,157E-07	-1,1540727	-0,4487452	0,65361549
VCA0114	1,39430523	0,64008784	1,23271167	6,246E-07	1,23271167	0,44737719	0,65460276
VC0964	1,09490235	-35,585156	-1,0713926	-3,472E-05	-1,0713926	-0,445922	0,65565363

VCA0965	1,18325184	1,52987558	1,09050716	1,4929E-06	1,09050716	0,44576717	0,65576546
VC0852	1,16464137	4,92319653	1,10266012	4,8041E-06	1,10266012	0,44551898	0,65594477
VCA0874	-1,0772742	-11,007375	-1,1002177	-1,074E-05	-1,1002177	-0,4452032	0,65617289
VC1757	1,14515134	2,22323342	1,11223059	2,1695E-06	1,11223059	0,44511951	0,65623341
VC1576	1,37863826	93,4237426	1,18264694	9,1164E-05	1,18264694	0,44474025	0,6565075
VC2567	1,01002955	-1,9141331	-1,0713438	-1,868E-06	-1,0713438	-0,4447384	0,65650885
VC1527	-1,0518454	-3,3463716	-1,138094	-3,265E-06	-1,138094	-0,4444962	0,65668392
VC2289	1,07555444	8,46209054	1,08876074	8,2574E-06	1,08876074	0,44409818	0,65697163
VC2405	1,08226249	2,84019586	1,04737531	2,7715E-06	1,04737531	0,44404304	0,65701147
VCA1106	-1,1136667	-6,1840643	-1,199318	-6,034E-06	-1,199318	-0,4435574	0,65736262
VCA0009	-1,1312869	-0,9031222	-1,1739337	-8,813E-07	-1,1739337	-0,4427756	0,65792809
VCA1018	1,36716918	0,71091806	1,20222676	6,9372E-07	1,20222676	0,44254713	0,65809335
5Sg	1,01155115	1019,63477	1,21234534	0,00099497	1,21234534	0,44211433	0,65840651
VC0081	1,18718847	1,26874467	1,10823654	1,2381E-06	1,10823654	0,44205637	0,65844843
nagA	1,21118078	11,5853751	1,14688426	1,1305E-05	1,14688426	0,44187933	0,65857656
fliG	1,04762745	3,66106783	1,03595147	3,5725E-06	1,03595147	0,44041396	0,65963733
VCA0133	-1,0460687	-1,231233	-1,115344	-1,201E-06	-1,115344	-0,4402343	0,65976745
VCA0472	-1,0070913	-1,9690599	-1,076005	-1,921E-06	-1,076005	-0,4401641	0,65981828
VCA0423	1,06862002	5,34943638	1,05665673	5,22E-06	1,05665673	0,43967438	0,66017297
VC1369	1,2895419	0,85827661	1,16230946	8,3751E-07	1,16230946	0,43963117	0,66020428
VC1660	-1,0221677	-0,9538769	-1,1439862	-9,308E-07	-1,1439862	-0,438387	0,66110576
VCA0065	1,41399378	0,5167857	1,2824618	5,0428E-07	1,2824618	0,43802049	0,66137145
VCA0694	-1,0851485	-12,113695	-1,1032024	-1,182E-05	-1,1032024	-0,4369035	0,66218134
tRNA-Lys-2	1,14431239	2,04028065	1,1265173	1,9909E-06	1,1265173	0,43672512	0,66231073
fadA	-1,0767244	-1,3694239	-1,1901007	-1,336E-06	-1,1901007	-0,4366674	0,66235257
VC1597	1,2850413	0,86523732	1,15832837	8,4431E-07	1,15832837	0,43636521	0,66257179
VCA0331	-1,006292	-1,6616822	-1,0818264	-1,621E-06	-1,0818264	-0,4359535	0,66287049
VC0911	-1,256557	-75,633788	-1,4290635	-7,38E-05	-1,4290635	-0,4350765	0,66350691
VCA0812	1,33871076	0,56925911	1,24882211	5,5549E-07	1,24882211	0,43469346	0,66378496
VC1980	1,19063227	1,18168615	1,11235895	1,1531E-06	1,11235895	0,43424422	0,66411112
VCA0989	1,3153943	0,79832404	1,17087384	7,7901E-07	1,17087384	0,43418199	0,66415631
VCA0004	-1,1253063	-18,186583	-1,1097243	-1,775E-05	-1,1097243	-0,4341126	0,66420668
VC0001	-1,1011621	-1,0057648	-1,1397411	-9,814E-07	-1,1397411	-0,4339725	0,66430845
VC1333	-1,0630621	-0,5777138	-1,2436448	-5,637E-07	-1,2436448	-0,4338296	0,66441224
mnmC	1,21092442	1,02666564	1,12999477	1,0018E-06	1,12999477	0,43356139	0,66460699
VC2006	-1,1084575	-17,686196	-1,1130861	-1,726E-05	-1,1130861	-0,4333417	0,66476659
tRNA-Met-8	-1,4267969	-0,5257375	-1,4416513	-5,13E-07	-1,4416513	-0,4327948	0,66516384
VC1812	-1,0536999	-0,8904746	-1,1549734	-8,689E-07	-1,1549734	-0,4321947	0,66559991
VC2384	1,16391519	1,55030224	1,10716591	1,5128E-06	1,10716591	0,43180539	0,66588287
VC2770	1,15072129	45,033808	1,1062278	4,3944E-05	1,1062278	0,43151013	0,66609751
VC1377	-1,0516022	-8,2915118	-1,1074324	-8,091E-06	-1,1074324	-0,4313913	0,66618392
deoA	-1,1057183	-6,5564149	-1,2248265	-6,398E-06	-1,2248265	-0,4313342	0,66622539
VC0778	1,54378819	0,35998339	1,41541362	3,5127E-07	1,41541362	0,43096966	0,66649045
VC1096	1,22561521	1,01143442	1,12997041	9,8697E-07	1,12997041	0,4302954	0,66698079
VCA0395	-1,0427538	-6,897041	-1,0992459	-6,73E-06	-1,0992459	-0,4302201	0,66703553

VC0492	1,23014643	55,8033399	1,18100607	5,4453E-05	1,18100607	0,42975434	0,66737437
VC0506	-1,011453	-2,2009669	-1,0574016	-2,148E-06	-1,0574016	-0,4292182	0,66776443
VCA0737	1,13334594	2,19150666	1,06277433	2,1385E-06	1,06277433	0,42843312	0,66833583
VCA1112	1,19944803	1,47520371	1,1539081	1,4395E-06	1,1539081	0,42772821	0,66884903
tRNA-Ala-1	1,27119184	1,58226766	1,18778153	1,544E-06	1,18778153	0,42752775	0,668995
VC1685	-1,0066097	-1,8065502	-1,0697517	-1,763E-06	-1,0697517	-0,4273755	0,66910587
VC1361	-1,0571317	-0,7196788	-1,1847439	-7,023E-07	-1,1847439	-0,427283	0,6691732
VC0267	-1,0125015	-1,7557535	-1,1277069	-1,713E-06	-1,1277069	-0,4255998	0,67039951
VC2652	1,00476579	-0,8522067	-1,1524518	-8,316E-07	-1,1524518	-0,4255336	0,67044773
cpxP	1,2057601	0,96181856	1,13372124	9,3855E-07	1,13372124	0,42524596	0,67065739
VC0950	1,13716801	3,26484251	1,09330768	3,1859E-06	1,09330768	0,4250671	0,67078778
VCA0425	1,15608691	4,75971372	1,11263176	4,6446E-06	1,11263176	0,42506126	0,67079203
rpsR	1,31582874	65,9747795	1,09973891	6,4379E-05	1,09973891	0,42503219	0,67081323
VCA0553	1,17586244	1,12557784	1,11280373	1,0983E-06	1,11280373	0,42460273	0,67112632
VC0476	-1,2080123	-15,949058	-1,1504528	-1,556E-05	-1,1504528	-0,4239128	0,67162942
VC0894	1,21497608	1,94796232	1,17583508	1,9008E-06	1,17583508	0,42299622	0,67229804
VCA0702	1,33981267	10,5163387	1,28192296	1,0262E-05	1,28192296	0,42236162	0,67276112
VC0586	-1,1429939	-10,069396	-1,1397026	-9,826E-06	-1,1397026	-0,4217125	0,67323493
VC2487	1,27305036	0,81976975	1,15582303	7,9994E-07	1,15582303	0,42161597	0,67330536
VCA0937	1,21708494	0,95232329	1,13256014	9,2929E-07	1,13256014	0,42141529	0,67345187
VCA0716	1,22108964	0,90532177	1,13983128	8,8342E-07	1,13983128	0,42128499	0,673547
VC2716	1,1687497	1,56197519	1,09841647	1,5242E-06	1,09841647	0,4212067	0,67360416
VCA0034	-1,1390597	-0,5661905	-1,2329683	-5,525E-07	-1,2329683	-0,4209685	0,67377808
VC2478	1,10687837	-71,85564	-1,0818944	-7,012E-05	-1,0818944	-0,4209442	0,67379586
VC0148	-1,0107001	-2,5143424	-1,0608164	-2,454E-06	-1,0608164	-0,4205093	0,67411345
VC1281	1,37926094	1,9546325	1,19336191	1,9073E-06	1,19336191	0,42035075	0,67422926
VCA0403	1,14615574	1,6062381	1,08314297	1,5674E-06	1,08314297	0,42018623	0,67434943
VCA0078	1,2783086	5,93382156	1,21186329	5,7903E-06	1,21186329	0,42007532	0,67443046
VCA0385	-1,0703693	-8,2758115	-1,087555	-8,076E-06	-1,087555	-0,4199445	0,67452602
VCA0638	-1,0761895	-0,6635144	-1,1941378	-6,475E-07	-1,1941378	-0,4196717	0,67472533
VC1955	1,30766062	0,67723639	1,18893674	6,6085E-07	1,18893674	0,41876773	0,6753859
VC1868	-1,0902548	-0,6367195	-1,2015753	-6,213E-07	-1,2015753	-0,4182032	0,67579859
VCA0700	-1,1167169	-0,5855276	-1,2206173	-5,714E-07	-1,2206173	-0,4177514	0,67612893
tRNA-Glu-3	1,12593096	19,8928192	1,18550774	1,9412E-05	1,18550774	0,41693449	0,67672635
VC2026	1,26505998	56,0684716	1,07098823	5,4712E-05	1,07098823	0,41669827	0,67689913
VC1194	1,17441791	1,26023477	1,10518701	1,2297E-06	1,10518701	0,41597344	0,67742945
VCA0723	-1,043416	-1,1760622	-1,103108	-1,148E-06	-1,103108	-0,4159047	0,67747978
VCA1058	1,36415603	0,66838396	1,18864025	6,5221E-07	1,18864025	0,41572339	0,67761244
VCA0250	-1,5174405	-31,801041	-1,688713	-3,103E-05	-1,688713	-0,4151796	0,67801044
VCA0654	-1,0347924	-1,8340283	-1,1364139	-1,79E-06	-1,1364139	-0,4146025	0,67843291
hemD	-1,0319107	-1,8451893	-1,1146922	-1,801E-06	-1,1146922	-0,4144482	0,67854594
VC2428	1,10937955	3,72944314	1,07801521	3,6392E-06	1,07801521	0,41440375	0,67857846
tRNA-Ala-4	1,28088431	1,1967189	1,27432152	1,1678E-06	1,27432152	0,41400115	0,67887328
VC1170	-1,029988	-2,2700528	-1,0701921	-2,215E-06	-1,0701921	-0,413953	0,67890857
VCA0769	1,193115	23,8664796	1,138442	2,3289E-05	1,138442	0,41377414	0,67903955

VC0233	1,07205114	2,36781744	1,05165233	2,3105E-06	1,05165233	0,41330395	0,67938395
VC0583	1,09734395	23,4778861	1,08637959	2,291E-05	1,08637959	0,41318261	0,67947284
VC1925	-1,0258598	-1,189067	-1,1384851	-1,16E-06	-1,1384851	-0,4124514	0,68000858
VC2212	-1,0184661	-2,4302711	-1,0593054	-2,371E-06	-1,0593054	-0,4122117	0,68018426
VC1168	1,18281159	3,21571404	1,13028436	3,1379E-06	1,13028436	0,41205771	0,68029714
VC2057	1,01180866	-2,2852169	-1,050744	-2,23E-06	-1,050744	-0,4118802	0,68042724
VC2560	1,44622165	1,96596631	1,48276257	1,9184E-06	1,48276257	0,41178013	0,68050061
VCA0158	-1,0301643	-0,9373186	-1,1274936	-9,146E-07	-1,1274936	-0,4105026	0,68143731
VC0913	-1,0226984	-1,158233	-1,101907	-1,13E-06	-1,101907	-0,4104463	0,68147863
VCA0873	-1,0097146	-1,8516023	-1,0624689	-1,807E-06	-1,0624689	-0,4101844	0,68167072
VC0802	1,17896421	1,88948642	1,12360348	1,8438E-06	1,12360348	0,40968573	0,68203652
VCA0706	-1,0262784	-0,8964094	-1,1330523	-8,747E-07	-1,1330523	-0,4095678	0,68212306
VC0299	1,30825775	0,57727383	1,21434587	5,6331E-07	1,21434587	0,40943694	0,68221905
flhA	-1,0296665	-4,162909	-1,0758965	-4,062E-06	-1,0758965	-0,409242	0,68236212
VCA0868	1,18845253	3,85315333	1,12039268	3,7599E-06	1,12039268	0,40904525	0,68250647
VC1700	1,13300785	2,19488613	1,08913157	2,1418E-06	1,08913157	0,408774	0,68270554
VCA0890	-1,017314	-0,8580787	-1,1384562	-8,373E-07	-1,1384562	-0,4082552	0,68308637
hmuV	-1,0314703	-3,0234104	-1,1380065	-2,95E-06	-1,1380065	-0,4073282	0,683767
VC0438	-1,1498306	-6,1864881	-1,1479632	-6,037E-06	-1,1479632	-0,4071259	0,68391554
VC2692	1,22177312	1,42568994	1,10967002	1,3912E-06	1,10967002	0,40619797	0,68459716
VCA0024	1,2227677	0,92340417	1,12616655	9,0107E-07	1,12616655	0,40544474	0,68515064
VC0875	1,10229376	5,86922598	1,08395036	5,7272E-06	1,08395036	0,40512778	0,6853836
VC1223	1,24983203	8,54909601	1,17500779	8,3423E-06	1,17500779	0,40436164	0,68594683
VCA0593	1,1432135	1,47424961	1,07645886	1,4386E-06	1,07645886	0,40355491	0,68654007
tRNA-Met-9	-1,0373721	-2,8069462	-1,050024	-2,739E-06	-1,050024	-0,4035204	0,68656545
rpsF	1,24118658	80,3992112	1,08143523	7,8454E-05	1,08143523	0,40213281	0,68758631
VCA1005	1,22117498	0,5864503	1,20235063	5,7226E-07	1,20235063	0,40205546	0,68764322
VC2299	1,15954574	6,61250064	1,1019104	6,4525E-06	1,1019104	0,40193657	0,68773073
upp	1,09314222	5,84586945	1,09332713	5,7044E-06	1,09332713	0,4015647	0,68800443
VC2542	1,15923191	1,42916629	1,07801768	1,3946E-06	1,07801768	0,40121571	0,68826133
VC0131	-1,0428765	-2,9213196	-1,1281515	-2,851E-06	-1,1281515	-0,4003536	0,68889611
VCA0139	-1,009166	-1,4667092	-1,0755336	-1,431E-06	-1,0755336	-0,4001678	0,68903291
csrC	1,00606362	1012,1188	1,06398605	0,00098763	1,06398605	0,39988633	0,68924027
VC2464	-1,0139618	-3,1987287	-1,0337891	-3,121E-06	-1,0337891	-0,3993051	0,68966844
VC1583	1,26373919	0,79853063	1,14245099	7,7921E-07	1,14245099	0,39910299	0,68981734
VC2751	1,12220507	4,77509898	1,07726153	4,6596E-06	1,07726153	0,39869356	0,69011903
VC1290	-1,0234031	-1,0551787	-1,1057278	-1,03E-06	-1,1057278	-0,3986754	0,69013241
VC1679	1,2620135	0,89040963	1,12651877	8,6887E-07	1,12651877	0,39865756	0,69014555
VC1078	-1,0516779	-0,9124443	-1,123002	-8,904E-07	-1,123002	-0,3982414	0,69045228
VC2729	1,09010121	4,30112191	1,0732213	4,1971E-06	1,0732213	0,3981442	0,69052391
VC2309	1,19890462	0,9105673	1,12312769	8,8854E-07	1,12312769	0,39802296	0,69061327
tRNA-Leu-8	-1,169629	-39,991096	-1,1792408	-3,902E-05	-1,1792408	-0,3977443	0,69081872
VC1724	1,00111004	-1,0212329	-1,1085999	-9,965E-07	-1,1085999	-0,3972307	0,69119737
VC1832	1,07258219	2,35428352	1,04569962	2,2973E-06	1,04569962	0,39722496	0,69120159
VCA1050	1,35239705	1,89412333	1,18076523	1,8483E-06	1,18076523	0,39704898	0,69133136

VCA1001	-1,0916616	-0,5572798	-1,2077647	-5,438E-07	-1,2077647	-0,3966499	0,69162566
VC0870	-1,2436853	-1,3121016	-1,2058556	-1,28E-06	-1,2058556	-0,3964096	0,69180292
VCA0381	-1,0018863	-1,0463093	-1,1053666	-1,021E-06	-1,1053666	-0,3963516	0,69184571
VCA0059	-1,2659122	-403,79591	-1,1303153	-0,000394	-1,1303153	-0,3962231	0,69194047
coaD	1,14845634	1,45466778	1,07424492	1,4195E-06	1,07424492	0,39523027	0,69267297
VC0620	-1,1738385	-7,9490119	-1,2985553	-7,757E-06	-1,2985553	-0,394931	0,69289384
VC1016	1,13107743	1,68027278	1,06384135	1,6396E-06	1,06384135	0,39488365	0,69292877
VC2730	1,08616435	8,04333576	1,08681384	7,8488E-06	1,08681384	0,39475981	0,69302017
VCA0856	-1,1668144	-0,3037131	-1,4097399	-2,964E-07	-1,4097399	-0,3936059	0,69387202
VCA0280	1,33322151	30,2606007	1,27109103	2,9529E-05	1,27109103	0,39342619	0,69400475
VC0857	1,19957605	1,71282118	1,13504664	1,6714E-06	1,13504664	0,39294502	0,6943601
VCA0418	1,21746607	0,94325055	1,13664721	9,2043E-07	1,13664721	0,39283688	0,69443998
rplU	-1,0194823	274,775076	1,17018809	0,00026813	1,17018809	0,39277613	0,69448486
VCA0431	1,24134012	5,9741423	1,1734908	5,8296E-06	1,1734908	0,39255319	0,69464954
VC2175	-1,0561203	-7,5277305	-1,1161671	-7,346E-06	-1,1161671	-0,3921811	0,69492443
VCA0530	-1,158571	-0,5329873	-1,2124723	-5,201E-07	-1,2124723	-0,3918609	0,69516104
rho	1,07420129	17,9524348	1,06384007	1,7518E-05	1,06384007	0,39148495	0,69543882
VCA0031	1,1678038	0,94904736	1,11378185	9,2609E-07	1,11378185	0,39148296	0,6954403
VC0151	1,38241494	6,67248523	1,23159558	6,5111E-06	1,23159558	0,39118242	0,69566243
mutL	1,11044692	2,0807223	1,05021873	2,0304E-06	1,05021873	0,39102945	0,69577548
VC0508	-1,031898	-1,2773055	-1,1246139	-1,246E-06	-1,1246139	-0,3908836	0,69588327
VC0575	1,14634727	24,2424485	1,14405084	2,3656E-05	1,14405084	0,39044079	0,69621065
VCA0639	-1,0463419	-0,5959199	-1,1863475	-5,815E-07	-1,1863475	-0,390353	0,69627553
VC1600	-1,0291686	-4,243301	-1,0837569	-4,141E-06	-1,0837569	-0,3899147	0,69659966
artP	1,37849693	3,25597866	1,29551476	3,1772E-06	1,29551476	0,38989204	0,69661641
VC1531	1,07431385	4,86635138	1,07480637	4,7486E-06	1,07480637	0,38981493	0,69667343
VCA0691	1,51978345	0,35311588	1,33343478	3,4457E-07	1,33343478	0,38906945	0,69722478
VC0809	1,21708654	1,01494121	1,10460658	9,9039E-07	1,10460658	0,3890248	0,69725782
VC1405	1,16163994	1,0702067	1,09879989	1,0443E-06	1,09879989	0,38876718	0,69744839
VC0205	2,09847039	0,18885985	1,72731932	1,8429E-07	1,72731932	0,38870944	0,69749111
VCA0894	1,11651814	1,83111115	1,05656023	1,7868E-06	1,05656023	0,38869585	0,69750116
VC2018	1,11016473	1,86976838	1,05533284	1,8245E-06	1,05533284	0,38860851	0,69756578
VC1709	1,16664812	1,97873407	1,10387098	1,9309E-06	1,10387098	0,38858578	0,6975826
VC1555	-1,0581134	-5,2203713	-1,1442901	-5,094E-06	-1,1442901	-0,3883827	0,69773285
VC0398	1,01737668	-1,6857173	-1,0614742	-1,645E-06	-1,0614742	-0,388344	0,69776148
VC1076	-1,0076023	-1,0736739	-1,0980518	-1,048E-06	-1,0980518	-0,3879885	0,69802454
rumB	1,18133488	1,01237305	1,10364719	9,8788E-07	1,10364719	0,38683469	0,69887861
VC0807	1,15588429	1,35597321	1,09060268	1,3232E-06	1,09060268	0,38666368	0,69900522
VCA0641	1,41638826	0,38864652	1,29382495	3,7924E-07	1,29382495	0,38645822	0,69915736
ftsA	-1,0521551	-6,8287532	-1,0449079	-6,664E-06	-1,0449079	-0,3864548	0,69915989
VC2163	-1,0229421	-1,8103188	-1,0564193	-1,767E-06	-1,0564193	-0,3860141	0,69948624
VC1165	1,28387994	0,70485874	1,15093831	6,8781E-07	1,15093831	0,38521084	0,70008122
VC1783	-1,0702701	-4,8887115	-1,2063938	-4,77E-06	-1,2063938	-0,3850556	0,70019627
csrB	-1,0291799	-5010,3194	-1,0931925	-0,0048891	-1,0931925	-0,3849075	0,70030594
VC1596	-1,1225415	-31,585191	-1,1910024	-3,082E-05	-1,1910024	-0,3845985	0,70053491

VC1012	1,14304453	1,1639193	1,08840034	1,1358E-06	1,08840034	0,38445462	0,70064154
VC0127	-1,0139191	-2,0219042	-1,0499275	-1,973E-06	-1,0499275	-0,3843708	0,70070363
VC2617	1,47706318	2,40246762	1,27181645	2,3443E-06	1,27181645	0,38418274	0,70084303
VC0262	1,08782473	2,64936778	1,05554035	2,5853E-06	1,05554035	0,38409109	0,70091095
VC1206	1,4109616	2,44933481	1,22892074	2,3901E-06	1,22892074	0,38405354	0,70093878
VCA0260	1,28104838	0,97784968	1,18117548	9,5419E-07	1,18117548	0,38335015	0,70146018
cls	1,00190185	-1,4660358	-1,0691091	-1,431E-06	-1,0691091	-0,3832778	0,70151384
VCA0420	1,13755052	2,80924013	1,07518023	2,7413E-06	1,07518023	0,38297037	0,70174175
VC1199	1,20244593	0,86132482	1,13245566	8,4049E-07	1,13245566	0,3826259	0,70199718
VCA0880	-1,2097864	-0,2634281	-1,45385	-2,571E-07	-1,45385	-0,3823174	0,70222599
tRNA-Met-3	-1,1589537	-25,669377	-1,1581797	-2,505E-05	-1,1581797	-0,3820272	0,70244118
VCA0708	-1,1135095	-0,5005223	-1,2151349	-4,884E-07	-1,2151349	-0,3818812	0,70254951
VC2023	1,12269975	6,69557793	1,10433673	6,5336E-06	1,10433673	0,38174351	0,70265164
VCA0103	1,13913151	1,37636181	1,07316464	1,3431E-06	1,07316464	0,38173746	0,70265612
VCA0758	1,43411703	3,05336296	1,35513967	2,9795E-06	1,35513967	0,38165213	0,70271943
VCA1063	1,56292313	4,04579459	1,53601994	3,9479E-06	1,53601994	0,38158043	0,70277263
VC1736	1,26103832	0,71090352	1,14634367	6,9371E-07	1,14634367	0,38133302	0,70295617
VCA0358	-1,0089047	-2,1919918	-1,1098044	-2,139E-06	-1,1098044	-0,3809223	0,70326091
VC0308	-1,0224493	-1,5446082	-1,0787741	-1,507E-06	-1,0787741	-0,3806363	0,70347312
VCA0179	1,35727424	1,16314451	1,31038032	1,135E-06	1,31038032	0,38031732	0,7037099
VC1416	1,27869547	0,52146325	1,20371002	5,0885E-07	1,20371002	0,38027875	0,70373852
VCA0267	1,26958289	0,5080987	1,20835636	4,9581E-07	1,20835636	0,37923119	0,70451621
VC1613	-1,0323695	-4,0905706	-1,1443518	-3,992E-06	-1,1443518	-0,3789351	0,70473605
VCA0884	1,13389309	1,27769404	1,0775572	1,2468E-06	1,0775572	0,37827901	0,70522335
VCA0549	1,1456155	9,38484335	1,11418661	9,1578E-06	1,11418661	0,37818447	0,70529358
VC0972	-1,0951784	-45,566242	-1,1986198	-4,446E-05	-1,1986198	-0,3766707	0,70641834
lipB	1,11138858	2,19739343	1,08358236	2,1442E-06	1,08358236	0,37654205	0,70651398
VC2711	1,16879615	1,38234776	1,07073373	1,3489E-06	1,07073373	0,37637843	0,70663559
VC1212	1,2159754	0,82114358	1,12176602	8,0128E-07	1,12176602	0,37599841	0,70691809
VC1390	1,41006243	0,44376531	1,23760454	4,3303E-07	1,23760454	0,37598754	0,70692617
VCA0160	1,2916912	0,99463041	1,16773434	9,7057E-07	1,16773434	0,37594015	0,70696141
VC1967	-1,0199657	-1,0750823	-1,0914661	-1,049E-06	-1,0914661	-0,3755682	0,70723798
VC1681	-1,042165	-1,0453942	-1,0969567	-1,02E-06	-1,0969567	-0,3754653	0,70731449
VC1430	1,05547933	2,57897458	1,0502686	2,5166E-06	1,0502686	0,37519491	0,70751553
23Sc	-1,042861	-0,8428757	-1,1178038	-8,225E-07	-1,1178038	-0,3750428	0,70762863
queF	1,12948446	1,37734104	1,07029331	1,344E-06	1,07029331	0,37456459	0,70798432
VC1994	1,14117124	1,68529331	1,06751113	1,6445E-06	1,06751113	0,37429141	0,70818753
VCA0557	-1,0041633	-1,4040998	-1,0736756	-1,37E-06	-1,0736756	-0,3730664	0,70909901
potE	1,45636425	2,45937608	1,45137226	2,3999E-06	1,45137226	0,3728389	0,70926837
VCA0285	1,12320517	1,43496865	1,06671694	1,4003E-06	1,06671694	0,37278986	0,70930486
tRNA-Met-2	-1,2166501	-16,900796	-1,1610901	-1,649E-05	-1,1610901	-0,3719924	0,70989855
VC2660	1,10973246	25,1372396	1,11387672	2,4529E-05	1,11387672	0,37134066	0,71038383
VCA0709	-1,0366166	-0,8773456	-1,1105281	-8,561E-07	-1,1105281	-0,3712689	0,71043726
deoD	-1,0871409	-10,922126	-1,0625943	-1,066E-05	-1,0625943	-0,3698316	0,71150797
VCA0620	1,1834416	2,61238523	1,09309004	2,5492E-06	1,09309004	0,36977714	0,71154856

VC1981	-1,0004135	-1,1224803	-1,0843521	-1,095E-06	-1,0843521	-0,3691606	0,71200806
tRNA-Tyr-1	-1,2181037	-16,794377	-1,1535596	-1,639E-05	-1,1535596	-0,3683652	0,71260099
VC1519	-1,0079626	-3,0989848	-1,1082386	-3,024E-06	-1,1082386	-0,3681407	0,71276834
VC0542	1,16502628	1,29070556	1,07239786	1,2595E-06	1,07239786	0,36779382	0,71302699
tRNA-Leu-9	-1,2239382	-11,495984	-1,205957	-1,122E-05	-1,205957	-0,3667722	0,71378895
VCA1084	-1,0079505	-1,4155672	-1,0651168	-1,381E-06	-1,0651168	-0,3659358	0,71441298
VC1582	1,3502087	0,40141652	1,24934976	3,9171E-07	1,24934976	0,36537146	0,71483415
VC2109	-1,0551013	-10,054605	-1,0795186	-9,811E-06	-1,0795186	-0,3652583	0,71491859
VC0304	-1,0442753	-2,8323409	-1,0545055	-2,764E-06	-1,0545055	-0,3652339	0,71493681
VC2383	1,17474957	1,01199305	1,09167574	9,8751E-07	1,09167574	0,36478071	0,71527512
VC0534	1,20858791	2,94389505	1,10812861	2,8727E-06	1,10812861	0,36471356	0,71532526
VC0587	1,1842757	0,8389925	1,11155343	8,187E-07	1,11155343	0,36465467	0,71536921
VC0829	-1,1642579	-3,0161417	-1,1703382	-2,943E-06	-1,1703382	-0,3644286	0,71553799
VC1023	1,154175	1,53124047	1,059433	1,4942E-06	1,059433	0,36410611	0,71577879
tRNA-Val-4	1,13470698	7,62557352	1,167456	7,4411E-06	1,167456	0,36286873	0,71670296
VC0396	-1,0647114	-0,5375785	-1,177806	-5,246E-07	-1,177806	-0,3628657	0,71670524
VC2011	1,17551769	1,81179423	1,08609714	1,768E-06	1,08609714	0,36243035	0,71703048
VC0825	1,16651341	1,63571967	1,11435549	1,5961E-06	1,11435549	0,36223666	0,7171752
VC1896	-1,0403963	-3,1633266	-1,047487	-3,087E-06	-1,047487	-0,3618598	0,71745678
VC0648	-1,0117708	-4,4499669	-1,0742606	-4,342E-06	-1,0742606	-0,3616867	0,71758615
VC0965	1,32142005	71,6875851	1,23362498	6,9953E-05	1,23362498	0,36133712	0,71784746
VC2462	1,08691785	3,37559213	1,06541879	3,2939E-06	1,06541879	0,36111498	0,71801351
hemG	1,16649616	1,49716014	1,05977771	1,4609E-06	1,05977771	0,36104363	0,71806684
VC1542	1,26482201	0,50338446	1,1888406	4,9121E-07	1,1888406	0,36095393	0,7181339
purN	-1,0886786	-3,2398176	-1,1086592	-3,161E-06	-1,1086592	-0,3605738	0,71841807
VC0452	1,28207807	0,66565153	1,13920148	6,4955E-07	1,13920148	0,36048008	0,71848816
VC1656	1,20614759	0,81200227	1,11269825	7,9236E-07	1,11269825	0,36047962	0,71848851
VCA1095	-1,1174392	-0,4917287	-1,1931564	-4,798E-07	-1,1931564	-0,3604489	0,71851146
VC1645	1,36405241	15,6320924	1,28475418	1,5254E-05	1,28475418	0,36032107	0,71860706
VCA0063	1,35776671	0,41653134	1,23168607	4,0645E-07	1,23168607	0,36017893	0,71871334
vrrA	1,28466237	48,840604	1,13222636	4,7659E-05	1,13222636	0,36002158	0,71883101
VC1725	1,00667327	-1,2927323	-1,0691304	-1,261E-06	-1,0691304	-0,3599645	0,71887372
hemA	-1,0124341	-3,7214577	-1,0389997	-3,631E-06	-1,0389997	-0,359351	0,71933253
VCA0510	1,17327732	1,51166105	1,09451508	1,4751E-06	1,09451508	0,35882538	0,71972574
VC2388	1,13197691	2,43063231	1,11924829	2,3718E-06	1,11924829	0,3585088	0,7199626
VCA0567	1,02969706	4,91112817	1,05862751	4,7923E-06	1,05862751	0,35716519	0,72096816
VC2435	-1,2385166	-34,880064	-1,180279	-3,404E-05	-1,180279	-0,3562233	0,72167339
VCA0734	1,03826092	-1,4213462	-1,0612429	-1,387E-06	-1,0612429	-0,3559418	0,72188416
VC1042	-1,0208053	-0,8279192	-1,1073807	-8,079E-07	-1,1073807	-0,3557525	0,72202594
VC0761	1,1029966	18,2409406	1,07210042	1,78E-05	1,07210042	0,35560156	0,72213899
VC1682	-1,0544515	-1,2830494	-1,1044998	-1,252E-06	-1,1044998	-0,3555791	0,72215584
VC0897	1,14351144	1,16119044	1,07509911	1,1331E-06	1,07509911	0,35506981	0,72253731
VC2178	-1,0378584	-5,8730654	-1,0798042	-5,731E-06	-1,0798042	-0,3548964	0,72266724
VCA0667	1,00019855	-0,6512837	-1,1377407	-6,355E-07	-1,1377407	-0,3548138	0,72272911
ispF	1,13019137	1,36985711	1,06320098	1,3367E-06	1,06320098	0,3548086	0,722733

VC2756	1,0162384	-1,2435959	-1,0697454	-1,214E-06	-1,0697454	-0,3545712	0,72291087
VC2637	1,27012798	0,58918225	1,15307459	5,7493E-07	1,15307459	0,35449357	0,72296904
VC2743	1,22832932	0,62604079	1,17346907	6,109E-07	1,17346907	0,35440785	0,72303326
VC1084	-1,0415708	-2,7266314	-1,1245561	-2,661E-06	-1,1245561	-0,3543498	0,72307675
VC2262	1,16944306	0,94354594	1,09279177	9,2072E-07	1,09279177	0,35427143	0,72313549
VC1935	1,39720621	0,60769347	1,1563563	5,9299E-07	1,1563563	0,35308612	0,72402389
VC1410	1,19358086	1,01258362	1,11256635	9,8809E-07	1,11256635	0,3528399	0,72420848
VC2534	1,01863252	-1,5694906	-1,0542029	-1,532E-06	-1,0542029	-0,352481	0,72447755
mnmA	1,1312659	8,52380111	1,14611713	8,3176E-06	1,14611713	0,35190419	0,72491014
VC2422	1,03063893	-1,5379323	-1,0642335	-1,501E-06	-1,0642335	-0,3516451	0,72510444
VCA0317	1,11945904	1,58968565	1,07681928	1,5512E-06	1,07681928	0,35142471	0,72526976
VCA0039	1,18057691	3,4702373	1,08653858	3,3863E-06	1,08653858	0,35136881	0,72531169
VC1059	1,22189757	10,2186775	1,12956166	9,9715E-06	1,12956166	0,35127538	0,72538179
VC1804	-1,0553849	-0,6821098	-1,1277074	-6,656E-07	-1,1277074	-0,3504621	0,72599194
VC0899	-1,1152061	-12,909571	-1,1871409	-1,26E-05	-1,1871409	-0,3502184	0,72617484
VC1937	1,31390881	0,59008299	1,14888371	5,7581E-07	1,14888371	0,35021538	0,72617708
VC1760	1,06302052	5,46274467	1,08210176	5,3306E-06	1,08210176	0,34987271	0,72643424
VCA0026	-1,0390347	-7,3147221	-1,0672986	-7,138E-06	-1,0672986	-0,3494441	0,72675597
VC1795	1,18138576	0,78205533	1,10978763	7,6314E-07	1,10978763	0,34941239	0,72677974
VC2233	1,09189726	1,96549555	1,04227579	1,9179E-06	1,04227579	0,34937789	0,72680564
VC1180	1,01842065	-1,6478429	-1,0542814	-1,608E-06	-1,0542814	-0,3492615	0,72689302
VC1344	1,26910479	0,48792228	1,18169899	4,7612E-07	1,18169899	0,34915271	0,72697467
VC1116	1,17662206	1,54067436	1,12373067	1,5034E-06	1,12373067	0,34899945	0,72708973
VCA0382a	-1,0359114	-2,8188916	-1,1317167	-2,751E-06	-1,1317167	-0,3482378	0,72766163
adk	-1,0094349	-55,333467	-1,1797627	-5,399E-05	-1,1797627	-0,3481792	0,72770565
VC0220	-1,0139274	-1,2595211	-1,0838592	-1,229E-06	-1,0838592	-0,347906	0,72791081
VCA0033	1,25336687	0,61033731	1,14152044	5,9557E-07	1,14152044	0,34785243	0,72795102
VCA1033	-1,0288738	-0,6335939	-1,1358093	-6,183E-07	-1,1358093	-0,3476568	0,72809795
VC0143	1,32704079	0,56847473	1,16284353	5,5472E-07	1,16284353	0,34731936	0,72835142
VCA0008	-1,0776274	-0,7170825	-1,1283791	-6,997E-07	-1,1283791	-0,3471858	0,72845174
VC2156	-1,0159202	-5,5447964	-1,0538627	-5,411E-06	-1,0538627	-0,3469537	0,72862615
VC2437	-1,0519068	-5,3068798	-1,0938178	-5,178E-06	-1,0938178	-0,3467987	0,72874258
VC2466	1,05644533	-23,148388	-1,0549143	-2,259E-05	-1,0549143	-0,3467644	0,72876834
VCA0090	1,01112868	-0,8027424	-1,1050477	-7,833E-07	-1,1050477	-0,3466672	0,72884134
VC1118	1,00132525	-1,7493481	-1,1142566	-1,707E-06	-1,1142566	-0,3466324	0,7288675
ispG	1,08061367	2,85199746	1,03753142	2,783E-06	1,03753142	0,3464198	0,72902726
VCA0419	1,02522128	-1,4908408	-1,065368	-1,455E-06	-1,065368	-0,3457938	0,7294977
VC0033	1,23346029	1,0096006	1,15846799	9,8518E-07	1,15846799	0,34578508	0,72950425
VC1350	1,09987532	6,51073649	1,11065638	6,3532E-06	1,11065638	0,34560803	0,72963733
VC0403	1,04749739	2,37873696	1,03624805	2,3212E-06	1,03624805	0,34528414	0,72988078
VCA0740	1,11187869	1,54208091	1,05282129	1,5048E-06	1,05282129	0,34502416	0,73007622
VC0550	-1,0937199	-13,36561	-1,1546665	-1,304E-05	-1,1546665	-0,3444675	0,73049477
VC0612	-1,0350704	-0,8441032	-1,115063	-8,237E-07	-1,115063	-0,344299	0,73062146
23Sd	-1,0350452	-0,7676316	-1,1085275	-7,491E-07	-1,1085275	-0,3442857	0,73063148
VC1253	1,02354378	-1,7943216	-1,0542019	-1,751E-06	-1,0542019	-0,343378	0,73131412

VC0788	1,24833585	1,47768786	1,15179008	1,4419E-06	1,15179008	0,34337684	0,73131499
VC2337	-1,0823388	-11,392977	-1,1465712	-1,112E-05	-1,1465712	-0,3431584	0,73147934
VC0044	1,11769431	1,52515872	1,05278749	1,4883E-06	1,05278749	0,34301883	0,73158431
VC0672	1,00067078	-2,3718216	-1,0367666	-2,314E-06	-1,0367666	-0,3425215	0,73195844
VCA1004	-1,0682973	-5,0903291	-1,1741195	-4,967E-06	-1,1741195	-0,3424647	0,73200124
purH	1,20681847	1,42929164	1,16390642	1,3947E-06	1,16390642	0,34224736	0,73216476
VCA0249	1,23360857	5,06349925	1,18475718	4,941E-06	1,18475718	0,34181077	0,73249332
VC1723	-1,0278251	-0,9675548	-1,0837274	-9,441E-07	-1,0837274	-0,3415185	0,7327133
VCA0683	-1,0848014	-1,7255621	-1,1657177	-1,684E-06	-1,1657177	-0,3412778	0,73289448
VC0859	1,20887815	0,52059968	1,15934811	5,0801E-07	1,15934811	0,3394888	0,73424154
VCA0975	1,13066464	7,44079436	1,09857167	7,2608E-06	1,09857167	0,33913585	0,73450741
VCA0949	-1,012644	-0,8773929	-1,0913713	-8,562E-07	-1,0913713	-0,3391168	0,73452178
VCA1081	1,11992322	1,50966089	1,05945533	1,4731E-06	1,05945533	0,33818347	0,73522494
VC1731	-1,1607809	-6,5932103	-1,1447708	-6,434E-06	-1,1447708	-0,3381206	0,7352723
VC1525	-1,0471097	-3,2737282	-1,1396841	-3,195E-06	-1,1396841	-0,3379153	0,73542706
VCA0618	1,16283414	0,93226297	1,08505709	9,0971E-07	1,08505709	0,33777582	0,73553215
VC1587	1,32470695	0,41689671	1,20033521	4,0681E-07	1,20033521	0,33745011	0,73577763
VC2113	1,25063915	0,7341323	1,13969531	7,1637E-07	1,13969531	0,3373137	0,73588045
5Sc	1,01150901	742,907109	1,16035841	0,00072494	1,16035841	0,3372799	0,73590593
VC0047	1,25718199	0,66023019	1,12178004	6,4426E-07	1,12178004	0,33716883	0,73598965
VC0633	-1,0289859	248,66608	1,05634374	0,00024265	1,05634374	0,33684961	0,73623029
VC2475	1,08655455	1,73557694	1,04453786	1,6936E-06	1,04453786	0,33678916	0,73627585
VC0344	1,10173491	1,85467488	1,04146366	1,8098E-06	1,04146366	0,33617637	0,73673788
VC1246	1,11098232	3,66332543	1,09895701	3,5747E-06	1,09895701	0,33579463	0,73702576
VC1302	1,19654216	3,9977387	1,1172252	3,901E-06	1,1172252	0,33531727	0,73738578
VC1129	1,13035594	2,78501842	1,12828281	2,7176E-06	1,12828281	0,33515198	0,73751046
VC0140	1,03059233	-1,5910576	-1,0780398	-1,553E-06	-1,0780398	-0,3351492	0,73751254
VC1743	1,00884574	-3,0437891	-1,0763675	-2,97E-06	-1,0763675	-0,3349035	0,73769789
VC2332	1,01068788	-2,3466951	-1,0715616	-2,29E-06	-1,0715616	-0,3336844	0,73861777
VC1797	-1,0421905	-0,7980883	-1,0971556	-7,788E-07	-1,0971556	-0,3330478	0,73909822
VCA0603	1,25305436	0,6273613	1,12523767	6,1218E-07	1,12523767	0,33303076	0,73911107
VC0287	-1,0912835	-1,9205655	-1,259809	-1,874E-06	-1,259809	-0,3324306	0,73956414
VC0641	-1,0860124	-12,765556	-1,066728	-1,246E-05	-1,066728	-0,3324043	0,73958402
VC0064	1,25651966	0,64864966	1,12020317	6,3296E-07	1,12020317	0,33215146	0,7397749
VC1473	-1,0482183	-0,8292026	-1,0927894	-8,091E-07	-1,0927894	-0,332108	0,73980772
VCA0164	1,00892311	-1,2578058	-1,060149	-1,227E-06	-1,060149	-0,3319228	0,73994758
VC2704	-1,0853914	-0,3159176	-1,2624708	-3,083E-07	-1,2624708	-0,3315864	0,74020156
VC0593	1,095932	1,99121578	1,06318353	1,943E-06	1,06318353	0,3310273	0,74062388
VCA0833	1,0026618	-0,9290845	-1,0816768	-9,066E-07	-1,0816768	-0,3306994	0,74087156
VC0177	1,18875	1,35643298	1,10855699	1,3236E-06	1,10855699	0,33013609	0,74129715
rnpA	-1,0928629	-23,727385	-1,0996071	-2,315E-05	-1,0996071	-0,3300976	0,74132624
VC1335	1,36466943	0,5606246	1,15048236	5,4706E-07	1,15048236	0,32988207	0,74148908
VCA0789	-1,0423336	-0,689966	-1,1106531	-6,733E-07	-1,1106531	-0,3294194	0,7418387
VCA1017	1,15892684	1,17143383	1,10391809	1,1431E-06	1,10391809	0,32894663	0,74219604
VCA0198	-1,0290826	-9,9373912	-1,0404109	-9,697E-06	-1,0404109	-0,3278237	0,74304498

VC1710	1,23672159	1,66472962	1,14206128	1,6245E-06	1,14206128	0,32780282	0,74306077
VC1693	1,23880813	0,51084116	1,15055819	4,9848E-07	1,15055819	0,32755251	0,74325004
VC0601	1,15801083	0,78592855	1,09510141	7,6692E-07	1,09510141	0,32714852	0,74355557
VC1295	1,26376117	0,55198524	1,13774084	5,3863E-07	1,13774084	0,32664732	0,74393466
VC0687	-1,0982748	-41,747908	-1,3606916	-4,074E-05	-1,3606916	-0,3266311	0,74394696
VC1755	1,09517261	2,12566867	1,06048712	2,0742E-06	1,06048712	0,32631787	0,74418388
VC0338	-1,0529937	-4,6761948	-1,1508767	-4,563E-06	-1,1508767	-0,3261565	0,74430598
VC2719	-1,0794662	-0,4313917	-1,1789668	-4,21E-07	-1,1789668	-0,3260303	0,74440144
vibF	1,31238159	0,40971709	1,18865665	3,9981E-07	1,18865665	0,32549957	0,74480302
pbpG	1,15717498	0,9683307	1,07557812	9,4491E-07	1,07557812	0,32523988	0,74499954
VC0717	1,12642528	2,43564065	1,08950017	2,3767E-06	1,08950017	0,32506697	0,7451304
VC1013	1,12294048	1,26455165	1,0602579	1,234E-06	1,0602579	0,32454147	0,74552815
VCA0559	1,01839238	-1,4892885	-1,0480803	-1,453E-06	-1,0480803	-0,323867	0,74603875
VCA0836	1,07595717	1,74556106	1,04087425	1,7033E-06	1,04087425	0,32385764	0,74604583
VC1680	-1,0126195	-1,1361654	-1,0673157	-1,109E-06	-1,0673157	-0,3237367	0,7461374
VC2254	-1,0123264	-2,5788335	-1,0629994	-2,516E-06	-1,0629994	-0,3235885	0,74624961
VC0560	-1,0243075	-2,755545	-1,0256308	-2,689E-06	-1,0256308	-0,3234362	0,7463649
VCA0647	-1,0906546	-0,4089076	-1,1862629	-3,99E-07	-1,1862629	-0,3232851	0,74647933
VC1650	1,25761952	0,4953307	1,15106424	4,8335E-07	1,15106424	0,32304509	0,7466611
VC2287	1,21521939	0,65675124	1,11162955	6,4086E-07	1,11162955	0,32273282	0,74689761
VCA0927	1,18198193	0,58904294	1,12524076	5,7479E-07	1,12524076	0,3227037	0,74691966
thiG	1,29217187	0,49600047	1,15027959	4,84E-07	1,15027959	0,32248162	0,74708787
VC0622	-1,0069052	-3,4891522	-1,1310813	-3,405E-06	-1,1310813	-0,3224201	0,74713448
VCA0405	1,13111416	8,89661529	1,10252833	8,6814E-06	1,10252833	0,32193585	0,74750131
VC1553	1,23269931	0,49520129	1,14947	4,8322E-07	1,14947	0,32141311	0,74789736
VC1851	1,02163415	-1,405345	-1,0501955	-1,371E-06	-1,0501955	-0,3212867	0,74799311
VCA0002	1,09083462	3,53516366	1,04962349	3,4496E-06	1,04962349	0,32113063	0,74811141
VCA0818	1,22607652	0,80792395	1,10003204	7,8838E-07	1,10003204	0,32106895	0,74815815
truA	1,13591085	1,0308024	1,06894044	1,0059E-06	1,06894044	0,32100711	0,74820502
VCA0811	1,28211424	0,50941579	1,14452832	4,9709E-07	1,14452832	0,32092845	0,74826463
VCA0369	1,1235791	0,98262944	1,07233314	9,5886E-07	1,07233314	0,32077269	0,74838267
VC2238	1,14119298	0,97233095	1,07283843	9,4881E-07	1,07283843	0,32016081	0,74884644
VC2551	1,19305728	1,49195973	1,08866973	1,4559E-06	1,08866973	0,32004899	0,7489312
VC1422	1,23007369	4,47701578	1,23511488	4,3687E-06	1,23511488	0,31968909	0,74920405
VC2626	1,08829303	2,75197387	1,06396022	2,6854E-06	1,06396022	0,31816267	0,75036156
VCA0177	-1,0369663	-5,2794288	-1,0459474	-5,152E-06	-1,0459474	-0,3179053	0,75055677
VC1311	-1,0288761	-0,6512601	-1,1088896	-6,355E-07	-1,1088896	-0,3176183	0,75077453
VC2373	1,28985826	5,05667514	1,33541854	4,9343E-06	1,33541854	0,31679212	0,75140136
VCA0325	1,15301315	1,07514688	1,11568186	1,0491E-06	1,11568186	0,31646555	0,75164919
VCA0481	1,13758021	1,88164567	1,07479705	1,8361E-06	1,07479705	0,31621855	0,75183664
VC1528	1,071272	2,06383726	1,04084579	2,0139E-06	1,04084579	0,31614262	0,75189427
smpB	1,05862597	3,27270749	1,05071762	3,1935E-06	1,05071762	0,31496272	0,75278997
VC1459	-1,1606245	-0,2961925	-1,2513003	-2,89E-07	-1,2513003	-0,3149399	0,75280728
VC1811	-1,0099696	-1,5313651	-1,0440651	-1,494E-06	-1,0440651	-0,3147078	0,75298352
trmE	-1,0057321	-1,2199763	-1,0554573	-1,19E-06	-1,0554573	-0,3142435	0,75333615

VC1605	1,31130528	1,75697028	1,14649654	1,7145E-06	1,14649654	0,31403347	0,75349563
VCA0330	1,14875979	1,08951587	1,0622082	1,0632E-06	1,0622082	0,31400663	0,75351601
VC1394	-1,0172465	-1,0641423	-1,0637235	-1,038E-06	-1,0637235	-0,31397	0,75354381
VC0706	1,07665498	1062,35326	1,22165954	0,00103665	1,22165954	0,31391569	0,75358509
VC1776	-1,177441	-17,034693	-1,2698811	-1,662E-05	-1,2698811	-0,313208	0,75412263
VC0305	1,04250634	2,55680954	1,02589301	2,495E-06	1,02589301	0,31312234	0,75418773
VC0471	1,28538882	0,56136369	1,12303297	5,4778E-07	1,12303297	0,31240372	0,75473373
VC2353	1,01084555	-1,0407776	-1,0644571	-1,016E-06	-1,0644571	-0,3122306	0,75486528
VC2176	-1,0437592	-6,6374737	-1,0739607	-6,477E-06	-1,0739607	-0,3121873	0,7548982
VCA0523	-1,0941609	-4,4169994	-1,1297249	-4,31E-06	-1,1297249	-0,3117818	0,75520638
VC1887	1,08743075	3,90536224	1,0565011	3,8109E-06	1,0565011	0,31153573	0,7553934
VCA1085	1,001122	-4,0076576	-1,1213149	-3,911E-06	-1,1213149	-0,3114395	0,75546655
tRNA-Glu-1	1,32641813	0,23237512	1,32289156	2,2675E-07	1,32289156	0,31129311	0,75557782
VC1807	1,36208059	0,39622606	1,17618941	3,8664E-07	1,17618941	0,31022316	0,75639127
VCA0631	1,01150161	-0,771008	-1,0867269	-7,524E-07	-1,0867269	-0,3100532	0,7565205
VCA1109	1,3716651	0,32208374	1,22088005	3,1429E-07	1,22088005	0,30999955	0,75656131
VC2634	1,18197818	0,66166551	1,10168521	6,4566E-07	1,10168521	0,30990344	0,7566344
VCA0537	1,22310623	3,23431775	1,10398122	3,1561E-06	1,10398122	0,30982386	0,75669492
VC0052	1,12792201	1,43590248	1,10487669	1,4012E-06	1,10487669	0,3098061	0,75670843
VCA0182	1,2341383	0,64056005	1,10486767	6,2506E-07	1,10486767	0,30942138	0,75700102
helD	1,00831776	-1,1730831	-1,055868	-1,145E-06	-1,055868	-0,3092528	0,75712927
VC1960	1,15089877	1,3930712	1,05548108	1,3594E-06	1,05548108	0,30909762	0,75724728
VC2399	1,03485574	7,60053065	1,03910267	7,4167E-06	1,03910267	0,30901868	0,75730733
VCA0777	1,00186477	-0,8670923	-1,0761432	-8,461E-07	-1,0761432	-0,3088749	0,75741667
VC1990	1,12117979	0,9165536	1,07165933	8,9438E-07	1,07165933	0,3084037	0,75777518
VC0757	-1,0235912	-1,4239122	-1,0720819	-1,389E-06	-1,0720819	-0,3082895	0,75786204
VC1196	1,09791367	3,04323578	1,06980369	2,9696E-06	1,06980369	0,30815058	0,75796777
VC0777	1,42156544	0,25361448	1,28423531	2,4748E-07	1,28423531	0,30769218	0,75831658
VC2063	1,01394722	5,82142494	1,033128	5,6806E-06	1,033128	0,30701566	0,75883146
VC1674	1,0297692	-0,5108115	-1,1310034	-4,985E-07	-1,1310034	-0,3069312	0,75889572
VC0689	1,14571325	1,00950693	1,06400693	9,8509E-07	1,06400693	0,30646202	0,7592529
metF	1,01610302	-1,4967685	-1,0873344	-1,461E-06	-1,0873344	-0,3055141	0,75997463
VC1439	-1,0515867	-7,0084356	-1,0599091	-6,839E-06	-1,0599091	-0,3048042	0,76051528
VC1606	1,20532887	1,50280033	1,12653212	1,4664E-06	1,12653212	0,3032744	0,76168077
VCA0485	1,15017037	2,86918212	1,06909949	2,7998E-06	1,06909949	0,30298592	0,7619006
VC2676	-1,0000885	-3,6476927	-1,0614165	-3,559E-06	-1,0614165	-0,3029313	0,76194225
VC1780	1,3788869	6,34834778	1,19142598	6,1948E-06	1,19142598	0,30292168	0,76194956
VC1876	1,11668116	5,54289704	1,08306535	5,4088E-06	1,08306535	0,3028398	0,76201196
VCA0354	-1,0254611	-1,3836423	-1,0898016	-1,35E-06	-1,0898016	-0,3026141	0,76218395
VC1466	1,12018008	1,14440536	1,10256385	1,1167E-06	1,10256385	0,30256086	0,76222456
VCA0104	1,14710367	1,53311595	1,0614244	1,496E-06	1,0614244	0,30244686	0,76231145
VC2432	-1,1576216	-10,056226	-1,1147757	-9,813E-06	-1,1147757	-0,3019149	0,76271694
VC0473	-1,0348275	-4,6421861	-1,1069418	-4,53E-06	-1,1069418	-0,3018216	0,7627881
thyA	1,02272208	3,79853613	1,02956811	3,7066E-06	1,02956811	0,30145975	0,76306395
VC1469	-1,103109	-0,2763485	-1,2461903	-2,697E-07	-1,2461903	-0,3014405	0,76307859

engB	-1,0416467	-3,0851803	-1,031474	-3,011E-06	-1,031474	-0,300732	0,7636189
VC0454	1,14548326	0,90493979	1,0747665	8,8305E-07	1,0747665	0,30034127	0,76391687
VC2075	-1,0846922	-3,6094154	-1,1081528	-3,522E-06	-1,1081528	-0,300269	0,76397201
VC2358	1,12063085	6,20610805	1,08947206	6,056E-06	1,08947206	0,30008095	0,76411543
VC0230	-1,0168368	-1,9926803	-1,0402053	-1,944E-06	-1,0402053	-0,2995923	0,76448815
VCA0061	-1,1400242	-6,9828316	-1,098617	-6,814E-06	-1,098617	-0,2995701	0,76450509
VC2604	-1,0143951	-5,4010962	-1,0224453	-5,27E-06	-1,0224453	-0,299478	0,76457536
VCA0871	1,12772367	1,07175814	1,05732914	1,0458E-06	1,05732914	0,2993291	0,76468897
rrmA	1,12132562	1,05996633	1,05798511	1,0343E-06	1,05798511	0,29932831	0,76468957
VC1722	1,00823852	-23,059358	-1,068557	-2,25E-05	-1,068557	-0,2987127	0,76515924
VCA0889	1,19511941	0,69939814	1,08868677	6,8248E-07	1,08868677	0,29848146	0,76533573
mraW	1,16476363	1,11619721	1,05830805	1,0892E-06	1,05830805	0,298463	0,76534982
VC0701	1,08860271	1,32458894	1,04826478	1,2925E-06	1,04826478	0,29820768	0,76554467
VC1772	1,17571197	1,60150529	1,10424785	1,5628E-06	1,10424785	0,29812221	0,7656099
VCA0962	1,17397764	0,8990111	1,06814479	8,7726E-07	1,06814479	0,29810681	0,76562166
VC1471	1,11727764	1,041197	1,07646861	1,016E-06	1,07646861	0,29798141	0,76571736
VC1391	1,13727333	0,59877148	1,10639851	5,8429E-07	1,10639851	0,29791659	0,76576683
VCA1077	-1,0021343	-2,4087372	-1,0565854	-2,35E-06	-1,0565854	-0,2974162	0,76614882
VC2621	1,00979226	-1,4717725	-1,1095792	-1,436E-06	-1,1095792	-0,2963431	0,76696812
VCA0208	1,01580299	-1,0318559	-1,0582962	-1,007E-06	-1,0582962	-0,2961011	0,76715286
VC1028	1,10286411	1,3479502	1,0666913	1,3153E-06	1,0666913	0,29605477	0,76718827
VC1675	1,02452305	-0,5927823	-1,1034997	-5,784E-07	-1,1034997	-0,2958066	0,76737777
VC2710	1,08080157	2,46991123	1,05193811	2,4102E-06	1,05193811	0,29529265	0,76777034
VC0096	1,15204694	0,43232345	1,14399738	4,2186E-07	1,14399738	0,29514191	0,76788548
VC0069	1,1213823	0,88430197	1,06788902	8,6291E-07	1,06788902	0,29512087	0,76790156
pyrH	1,08546616	3,69049455	1,07345523	3,6012E-06	1,07345523	0,29418035	0,76862011
VC1395	1,11354104	0,69298482	1,08642218	6,7622E-07	1,08642218	0,29345112	0,76917737
VCA0773	-1,0357928	-4,9968851	-1,1039317	-4,876E-06	-1,1039317	-0,2930841	0,76945788
VC2720	1,24234697	5,48143485	1,11160612	5,3488E-06	1,11160612	0,29238863	0,76998952
secD (NC_002506 631616..633495)	1,19352659	2,19004653	1,12947287	2,1371E-06	1,12947287	0,29228596	0,770068
VCA0416	1,15015405	3,60007352	1,08705235	3,513E-06	1,08705235	0,29215585	0,77016748
VCA0178	1,14450468	1,1243227	1,05764374	1,0971E-06	1,05764374	0,29207233	0,77023133
VCA0070	1,22769229	0,5131167	1,1172793	5,007E-07	1,1172793	0,29200567	0,77028229
VCA0832	1,02888141	4,38753059	1,0366141	4,2814E-06	1,0366141	0,29192484	0,77034411
VC0817	1,14431078	1,00038347	1,05844381	9,7618E-07	1,05844381	0,29190892	0,77035627
VC1380	1,02571849	-1,0660287	-1,0547438	-1,04E-06	-1,0547438	-0,2919026	0,77036107
VCA0731	1,31319833	0,27423584	1,23079559	2,676E-07	1,23079559	0,29174732	0,77047983
VC1366	1,09090449	1,65216956	1,05317614	1,6122E-06	1,05317614	0,2917341	0,77048995
VCA0212	1,10706735	0,81076869	1,07365683	7,9116E-07	1,07365683	0,29169776	0,77051773
VC1858	1,0211532	-1,1945147	-1,0486055	-1,166E-06	-1,0486055	-0,2915918	0,77059878
VC2070	1,1657236	10,3367495	1,12962638	1,0087E-05	1,12962638	0,29158464	0,77060424
VCA0976	1,30415079	0,36537946	1,16787476	3,5654E-07	1,16787476	0,29134583	0,77078685
VC1607	1,23597918	1,12941318	1,16300982	1,1021E-06	1,16300982	0,29107002	0,77099778
murC	1,07164346	1,63124589	1,03520213	1,5918E-06	1,03520213	0,29094507	0,77109334
VC1636	1,18596741	0,61255646	1,09638129	5,9774E-07	1,09638129	0,2906674	0,77130571

VCA1006	1,00306002	-0,4805991	-1,1243905	-4,69E-07	-1,1243905	-0,2905557	0,77139114
VC1356	1,15411369	0,80008165	1,07263552	7,8073E-07	1,07263552	0,29003022	0,77179313
VC1944	1,22087944	0,58595223	1,10048604	5,7178E-07	1,10048604	0,28999199	0,77182237
VC1541	1,20713234	0,49208447	1,12049814	4,8018E-07	1,12049814	0,28963603	0,77209471
VC1632	1,24977115	0,53350618	1,1105368	5,206E-07	1,1105368	0,28952657	0,77217846
VC1031	1,21492301	0,52536976	1,11232682	5,1266E-07	1,11232682	0,28950454	0,77219532
VCA0712	1,00553932	-0,7210616	-1,0803751	-7,036E-07	-1,0803751	-0,2890938	0,77250959
VC0892	1,03170124	-1,6548528	-1,0506772	-1,615E-06	-1,0506772	-0,2889083	0,77265157
nrdR	1,13458965	1,81678231	1,04739332	1,7728E-06	1,04739332	0,28869068	0,7728181
VC0433	1,14983259	0,8428206	1,0788773	8,2243E-07	1,0788773	0,28857134	0,77290944
VC1556	-1,0081994	-2,7025592	-1,1138701	-2,637E-06	-1,1138701	-0,2884622	0,77299294
VCA0159	1,26171875	0,44184563	1,13345121	4,3116E-07	1,13345121	0,28794966	0,77338529
VC1407	1,10675112	1,70431773	1,04928919	1,6631E-06	1,04928919	0,28755303	0,77368892
phhB	-1,0147868	-2,4293708	-1,0864599	-2,371E-06	-1,0864599	-0,2872499	0,77392099
VC1277	1,03124527	-1,1133347	-1,0773186	-1,086E-06	-1,0773186	-0,2868913	0,77419558
VCA0219	-1,1514772	-162,93983	-1,1532034	-0,000159	-1,1532034	-0,2868292	0,77424314
xni	1,01252231	-1,1843202	-1,0473618	-1,156E-06	-1,0473618	-0,2866932	0,77434723
VC0724	-1,0093286	-0,8884753	-1,0634965	-8,67E-07	-1,0634965	-0,286391	0,77457866
VC1571	-1,0697501	-0,6424025	-1,1538329	-6,269E-07	-1,1538329	-0,2856525	0,77514427
VC0342	1,11192233	2,22362428	1,08935892	2,1698E-06	1,08935892	0,28560596	0,77517995
VCA0987	1,25288907	9,2335568	1,29101414	9,0102E-06	1,29101414	0,28554516	0,77522653
VCA0239	1,11486491	0,81442219	1,06897432	7,9472E-07	1,06897432	0,28540013	0,77533761
VC1816	1,176457	0,59448687	1,09566885	5,8011E-07	1,09566885	0,28533635	0,77538648
VC1211	1,15202131	0,71966584	1,07765409	7,0226E-07	1,07765409	0,28406887	0,77635761
VCA0028	-1,0458632	-0,3064889	-1,1920509	-2,991E-07	-1,1920509	-0,2838254	0,77654421
VC2772	-1,0470004	-6,9586749	-1,0429531	-6,79E-06	-1,0429531	-0,2835959	0,77672013
VCA0579	1,12791682	1,13957338	1,04811508	1,112E-06	1,04811508	0,28340033	0,77686998
VCA0165	-1,0137045	-1,5221225	-1,1019241	-1,485E-06	-1,1019241	-0,2830905	0,77710751
VC1342	-1,015198	-0,8375221	-1,0763742	-8,173E-07	-1,0763742	-0,2826108	0,77747518
VC2081	1,16934248	1,25595552	1,10425132	1,2256E-06	1,10425132	0,28232591	0,77769363
VC0725	-1,0037923	-1,0450916	-1,0521545	-1,02E-06	-1,0521545	-0,2822819	0,77772737
VCA0355	-1,0095449	-0,8496381	-1,0644726	-8,291E-07	-1,0644726	-0,2821392	0,77783678
tRNA-Gly-8	-1,1173887	-5,255273	-1,1181518	-5,128E-06	-1,1181518	-0,2818277	0,77807563
VCA0253	1,14829425	1,27827804	1,1037473	1,2474E-06	1,1037473	0,28175788	0,77812918
VC0940	1,11237622	2,41868715	1,08768313	2,3602E-06	1,08768313	0,28108449	0,77864561
VCA0999	1,10123665	0,91960937	1,05894821	8,9736E-07	1,05894821	0,28104661	0,77867466
VC0673	1,0137283	-1,5238357	-1,0350561	-1,487E-06	-1,0350561	-0,280629	0,77899498
VCA0412	1,28421565	0,50220316	1,11134299	4,9005E-07	1,11134299	0,27971601	0,77969541
VC1441	1,10079082	16,9621582	1,08515386	1,6552E-05	1,08515386	0,27971215	0,77969837
VCA0191	1,12370686	1,25303565	1,04232938	1,2227E-06	1,04232938	0,27913018	0,78014493
fliM	-1,003309	-2,6257088	-1,0411981	-2,562E-06	-1,0411981	-0,2787633	0,7804265
tRNA-Tyr-4	-1,0961427	-0,9506586	-1,1797913	-9,277E-07	-1,1797913	-0,2785478	0,78059187
VCA0511	1,21556433	17,991614	1,18447124	1,7556E-05	1,18447124	0,27830725	0,78077652
VCA0203	1,02031994	13,0637911	1,05021572	1,2748E-05	1,05021572	0,27818494	0,78087041
VC0474	1,25857497	0,35545258	1,15581448	3,4685E-07	1,15581448	0,27761965	0,78130436

VCA0073	1,16160208	0,62600831	1,08545302	6,1086E-07	1,08545302	0,27740597	0,78146841
VC2024	1,06426934	3,8230044	1,06603803	3,7305E-06	1,06603803	0,2765206	0,78214826
VC0758	1,06833527	1,65721608	1,03115456	1,6171E-06	1,03115456	0,27615377	0,78242997
VCA0630	1,27083648	0,51892029	1,10292133	5,0637E-07	1,10292133	0,27602807	0,78252651
VCA0554	-1,0472223	-1,0114318	-1,0825906	-9,87E-07	-1,0825906	-0,2759633	0,78257628
VC2479	-1,0510758	-4,1064946	-1,0739718	-4,007E-06	-1,0739718	-0,275421	0,7829928
VC1718	1,02890535	-2,3671826	-1,0633643	-2,31E-06	-1,0633643	-0,2753474	0,78304937
VCA0971	1,00577219	-3,6248869	-1,0642054	-3,537E-06	-1,0642054	-0,2752897	0,78309372
fliA	1,00799786	4,45121647	1,02560841	4,3435E-06	1,02560841	0,27528887	0,78309433
VC0500	1,14803082	0,75740577	1,06892823	7,3908E-07	1,06892823	0,27513967	0,78320895
VC1591	1,18628185	1,92213313	1,14380372	1,8756E-06	1,14380372	0,27471021	0,7835389
VCA0386	1,00794691	-1,1352637	-1,0494766	-1,108E-06	-1,0494766	-0,2745837	0,78363614
ansA	1,21314347	3,74806552	1,1008437	3,6574E-06	1,1008437	0,27408366	0,78402035
VC1965	1,0331311	2,23766451	1,03759284	2,1835E-06	1,03759284	0,27403849	0,78405505
VCA0282	1,01790111	-1,2304377	-1,0461504	-1,201E-06	-1,0461504	-0,2733258	0,78460281
VC2378	1,30563909	2,18400421	1,1326553	2,1312E-06	1,1326553	0,27298533	0,78486451
VC1409	-1,0136106	-0,8286424	-1,1001965	-8,086E-07	-1,1001965	-0,2727308	0,78506013
tRNA-Leu-6	-1,0491856	-2,3579081	-1,0910276	-2,301E-06	-1,0910276	-0,2724244	0,78529576
VC0174	1,04582628	-1,3702192	-1,0492264	-1,337E-06	-1,0492264	-0,2723888	0,78532313
tRNA-Ile-1	1,12988457	28,7879745	1,12918647	2,8092E-05	1,12918647	0,27215869	0,78550002
trkA	1,02324809	-1,2775849	-1,0392749	-1,247E-06	-1,0392749	-0,2716955	0,7858562
VC0378	1,03210653	-8,5652651	-1,0247287	-8,358E-06	-1,0247287	-0,2716295	0,78590695
VC1605a	1,20636021	17,4508406	1,17529522	1,7029E-05	1,17529522	0,27146753	0,78603148
VC0973	-1,0627984	-17,546347	-1,1219745	-1,712E-05	-1,1219745	-0,2713599	0,78611425
VCA0313	1,09138917	1,60935076	1,04637261	1,5704E-06	1,04637261	0,27126909	0,78618409
VC2733	1,1313786	1,19112717	1,04192926	1,1623E-06	1,04192926	0,27088446	0,7864799
tRNA-Trp-1	1,1405099	12,1874487	1,19354317	1,1893E-05	1,19354317	0,27079253	0,78655062
VCA0346	1,11642889	3,20215794	1,07102223	3,1247E-06	1,07102223	0,2707771	0,78656249
VC2015	1,12216863	0,915743	1,05477665	8,9359E-07	1,05477665	0,27062413	0,78668015
ruvC	1,12621133	0,71723567	1,07039531	6,9988E-07	1,07039531	0,2704824	0,78678916
VCA1008	1,21899582	0,37644348	1,13754494	3,6734E-07	1,13754494	0,26957264	0,78748906
VC1119	1,25197688	0,8872305	1,12179564	8,6577E-07	1,12179564	0,26827184	0,78849009
murB	1,00555679	-1,5034797	-1,0323592	-1,467E-06	-1,0323592	-0,2679896	0,78870734
VC2242	-1,0145193	-0,67249	-1,0733867	-6,562E-07	-1,0733867	-0,2672232	0,78929731
dipZ	1,12865508	0,84406627	1,05791929	8,2365E-07	1,05791929	0,26696205	0,78949839
pflA	1,04001984	4,67022389	1,06004389	4,5572E-06	1,06004389	0,2667742	0,78964303
VC1604	1,23472915	1,6966353	1,09865049	1,6556E-06	1,09865049	0,26539792	0,79070296
VC1694	1,41835459	0,22023576	1,23787002	2,1491E-07	1,23787002	0,2650069	0,79100415
VC0791	1,20627735	4,10480727	1,18360524	4,0055E-06	1,18360524	0,26492208	0,7910695
ispH	1,08051343	1,43620825	1,03817738	1,4015E-06	1,03817738	0,26458006	0,79133299
VC2536	-1,0435811	-4,6013408	-1,0365726	-4,49E-06	-1,0365726	-0,2644589	0,79142636
ftsB	1,08806139	1,41983272	1,04557571	1,3855E-06	1,04557571	0,26422548	0,79160619
VC0596	1,12691659	17,3073767	1,07136114	1,6889E-05	1,07136114	0,26421204	0,79161654
VCA0197	1,11200904	0,95277661	1,05513127	9,2973E-07	1,05513127	0,26397457	0,79179952
VC2564	1,19871932	0,55340224	1,09820963	5,4001E-07	1,09820963	0,26345347	0,79220109

coaE	1,13655165	0,84164059	1,05650935	8,2128E-07	1,05650935	0,26340379	0,79223938
VC0970	1,06328692	6,1615862	1,03995065	6,0125E-06	1,03995065	0,2630395	0,79252015
VC0103	1,0118055	-0,9734727	-1,0484868	-9,499E-07	-1,0484868	-0,2629189	0,79261308
VC1379	1,15629111	0,73073766	1,06500893	7,1306E-07	1,06500893	0,26270562	0,79277749
VC0764	1,14170136	1,63191067	1,08660418	1,5924E-06	1,08660418	0,26203827	0,79329195
VC0221	1,11632112	0,86606122	1,05427363	8,4511E-07	1,05427363	0,26200144	0,79332034
VC2553	1,21884538	0,50386472	1,09467118	4,9168E-07	1,09467118	0,26137844	0,79380069
VC1347	1,00948966	-0,7225606	-1,064522	-7,051E-07	-1,064522	-0,2602822	0,79464612
VCA0254	1,24841891	0,28738616	1,16829808	2,8043E-07	1,16829808	0,25868666	0,79587702
uvrC	1,04479867	-1,6667615	-1,0399389	-1,626E-06	-1,0399389	-0,2583976	0,79610004
VCA0316	1,10886518	0,88309016	1,0539862	8,6173E-07	1,0539862	0,25753729	0,79676404
leuO	1,10161019	0,85217483	1,05322305	8,3156E-07	1,05322305	0,25743067	0,79684633
VC1524	-1,0443865	-1,8647856	-1,1071816	-1,82E-06	-1,1071816	-0,2573645	0,79689745
VCA0466	1,20013592	1,04852297	1,07626332	1,0232E-06	1,07626332	0,25727408	0,79696721
VC0968	1,07333901	29,4383201	1,14051447	2,8726E-05	1,14051447	0,25721407	0,79701353
VC2343	1,10541667	1,16014709	1,03862151	1,1321E-06	1,03862151	0,25678519	0,79734461
tRNA-Val-3	-1,0933161	-14,383877	-1,0694286	-1,404E-05	-1,0694286	-0,2565652	0,79751445
VCA0606	1,06905744	-0,3780153	-1,1221943	-3,689E-07	-1,1221943	-0,2555341	0,79831064
VCA0779	1,14182156	0,7765096	1,08105037	7,5772E-07	1,08105037	0,255498	0,7983385
VC1538	1,04195173	8,04233045	1,03746421	7,8478E-06	1,03746421	0,2550799	0,7986614
purU	1,10362501	3,67332152	1,07807146	3,5845E-06	1,07807146	0,25463222	0,79900719
VC0919	1,02560618	-0,488926	-1,0924402	-4,771E-07	-1,0924402	-0,2545583	0,79906425
VC1440	1,06034394	8,05827122	1,05981859	7,8633E-06	1,05981859	0,25445059	0,79914748
VCA0804	-1,2597365	-17,630205	-1,1818113	-1,72E-05	-1,1818113	-0,2538789	0,79958913
VC1732	1,1006744	3,06075106	1,07032861	2,9867E-06	1,07032861	0,25356033	0,79983526
VC2341	1,24590959	0,65774109	1,08997929	6,4183E-07	1,08997929	0,25276847	0,80044714
VC1775	-1,0330137	-6,3726286	-1,1344398	-6,218E-06	-1,1344398	-0,2521292	0,80094124
VC2326	1,00503697	-22,265067	-1,0926784	-2,173E-05	-1,0926784	-0,2520539	0,8009994
VCA0589	1,03617326	-0,9619371	-1,0449963	-9,387E-07	-1,0449963	-0,2519884	0,80105002
VCA1034	1,03114466	-0,4830686	-1,0914723	-4,714E-07	-1,0914723	-0,251759	0,80122736
VC1934	-1,0137106	-0,5089271	-1,0866018	-4,966E-07	-1,0866018	-0,2517291	0,80125046
pcm	1,03873242	-1,2424336	-1,0417458	-1,212E-06	-1,0417458	-0,2514121	0,80149549
VCA0442	1,13136508	0,97715801	1,08012684	9,5352E-07	1,08012684	0,25136008	0,80153574
VC1040	1,11382365	1,42699742	1,07907929	1,3925E-06	1,07907929	0,25047292	0,80222165
VC2630	1,14564252	0,60006051	1,07221172	5,8554E-07	1,07221172	0,25046484	0,8022279
VC2638	1,22762861	0,46569395	1,0938409	4,5443E-07	1,0938409	0,25022822	0,80241087
VCA0106	1,14978683	0,62948993	1,06848735	6,1426E-07	1,06848735	0,25005513	0,80254472
VC1947	1,19981833	0,4764919	1,09134406	4,6496E-07	1,09134406	0,24987163	0,80268663
metL	1,15944138	0,6078132	1,0707345	5,9311E-07	1,0707345	0,24957496	0,80291607
VC2076	1,13015019	6,37031974	1,14621329	6,2162E-06	1,14621329	0,24917214	0,80322764
VC2407	1,12058007	0,8776027	1,04820501	8,5637E-07	1,04820501	0,24892752	0,80341685
VC1399	1,187817	0,42241162	1,1025708	4,1219E-07	1,1025708	0,24863716	0,80364147
VC2609	1,14813455	2,54478745	1,06978048	2,4832E-06	1,06978048	0,2486369	0,80364167
VCA0119	1,13016396	0,46261508	1,09308167	4,5142E-07	1,09308167	0,24843377	0,80379881
VC1004	1,01520105	-1,1102287	-1,0377143	-1,083E-06	-1,0377143	-0,2482872	0,80391217

VCA0992	1,33087154	0,22595005	1,19993929	2,2048E-07	1,19993929	0,24820517	0,80397567
VC0864	1,00080755	-1,230053	-1,052846	-1,2E-06	-1,052846	-0,2481153	0,80404518
VC0920	1,226384	0,44126912	1,09751797	4,3059E-07	1,09751797	0,24808636	0,8040676
VC2005	-1,0769773	-13,820806	-1,1156524	-1,349E-05	-1,1156524	-0,2479095	0,80420447
VC1038	1,07256654	10,0873404	1,13080634	9,8433E-06	1,13080634	0,24776322	0,80431762
VC0539	1,18264635	1,52449114	1,16984125	1,4876E-06	1,16984125	0,24735662	0,80463226
yebU	-1,0122053	-0,6637118	-1,0687135	-6,477E-07	-1,0687135	-0,2470564	0,80486458
VC1643	1,0335502	-0,4515931	-1,0943007	-4,407E-07	-1,0943007	-0,2469865	0,80491864
murD	1,01508818	-1,1606281	-1,0442072	-1,133E-06	-1,0442072	-0,2469162	0,80497306
VC2508	1,04996113	-0,3952245	-1,1083659	-3,857E-07	-1,1083659	-0,2468634	0,80501393
VCA0210	1,00230506	-0,6005317	-1,0699036	-5,86E-07	-1,0699036	-0,2466637	0,80516855
VC0949	1,09282931	1,62657777	1,04592085	1,5872E-06	1,04592085	0,24660545	0,8052136
VC0231	1,067028	1,38252036	1,02969955	1,3491E-06	1,02969955	0,24635733	0,80540564
VC2497	1,09611439	4,18828094	1,05461161	4,087E-06	1,05461161	0,24619739	0,80552945
VC1421	1,01433377	-0,7617695	-1,0543698	-7,433E-07	-1,0543698	-0,2459319	0,80573495
VCA0550	1,0239274	-1,2298976	-1,0333051	-1,2E-06	-1,0333051	-0,2458427	0,80580399
VC0966	1,26319949	86,4921118	1,12515229	8,44E-05	1,12515229	0,24570583	0,80590997
VC0144	1,04553845	-2,500881	-1,0627704	-2,44E-06	-1,0627704	-0,2453068	0,80621892
VC0169	1,21341479	0,41043517	1,10274555	4,0051E-07	1,10274555	0,24528555	0,80623535
VCA1068	-1,0079258	-6,0646251	-1,1077982	-5,918E-06	-1,1077982	-0,2452718	0,80624598
VC2473	-1,07369	-14,627691	-1,0973864	-1,427E-05	-1,0973864	-0,2447593	0,8066428
VC0963	1,10516206	0,81395051	1,05011886	7,9426E-07	1,05011886	0,24432856	0,80697637
VCA0262	-1,0045852	-0,6253336	-1,0657214	-6,102E-07	-1,0657214	-0,2443071	0,80699296
fbpC	1,37287321	3,98082175	1,29286245	3,8845E-06	1,29286245	0,24364384	0,80750668
VCA0542	1,14595196	10,8878504	1,12428335	1,0624E-05	1,12428335	0,24294391	0,80804886
VC1585	1,01981142	-0,8251314	-1,0507239	-8,052E-07	-1,0507239	-0,2426454	0,8082801
VCA1003	1,01748057	-2,4183351	-1,095569	-2,36E-06	-1,095569	-0,2416484	0,80905263
VC0146	-1,0194602	-3,3259063	-1,0356092	-3,245E-06	-1,0356092	-0,2411564	0,80943392
VC2067	-1,0287408	-4,3084888	-1,0287797	-4,204E-06	-1,0287797	-0,2409428	0,80959942
VCA1059	1,10819704	0,81270472	1,05775866	7,9304E-07	1,05775866	0,24073099	0,80976363
artM	1,25857355	1,26229186	1,20250943	1,2318E-06	1,20250943	0,24063443	0,80983847
VCA0194	1,05254751	-0,9828722	-1,0430869	-9,591E-07	-1,0430869	-0,2402971	0,81009993
asnB	1,09105402	-43,908497	-1,0855186	-4,285E-05	-1,0855186	-0,2400216	0,81031353
VC2439	-1,009952	-4,7761959	-1,0737032	-4,661E-06	-1,0737032	-0,2398755	0,81042679
VCA0852	1,01540193	-0,4650836	-1,0859936	-4,538E-07	-1,0859936	-0,2398302	0,81046191
VC1806	1,23761652	0,36673465	1,11005099	3,5786E-07	1,11005099	0,23954541	0,8106827
VC2546	-1,0238453	-2,184204	-1,0236591	-2,131E-06	-1,0236591	-0,2393696	0,81081904
VCA0415	1,12130484	0,75324624	1,06999538	7,3502E-07	1,06999538	0,239339	0,81084274
VCA0600	1,12843756	0,63474442	1,06133029	6,1939E-07	1,06133029	0,23802691	0,81186023
VCA0529	-1,0406862	-0,2502185	-1,1630676	-2,442E-07	-1,1630676	-0,2378863	0,8119693
VC0585	-1,0699207	-5,5361819	-1,0440982	-5,402E-06	-1,0440982	-0,2377394	0,81208322
VC0783	1,20054948	0,40420887	1,09740816	3,9443E-07	1,09740816	0,23731253	0,81241435
VC0665	1,04770366	-1,1273041	-1,0524768	-1,1E-06	-1,0524768	-0,2369063	0,8127295
VC1130	1,01508246	-7,2019368	-1,0223402	-7,028E-06	-1,0223402	-0,2367925	0,81281779
VC0041	1,07335011	1,36117511	1,02775925	1,3282E-06	1,02775925	0,23644159	0,81309003

VC0921	1,18404728	0,32701925	1,1206968	3,1911E-07	1,1206968	0,23629606	0,81320295
tarB	-1,0232695	-1,624687	-1,0583282	-1,585E-06	-1,0583282	-0,2361024	0,81335324
VCA0688	1,20086541	0,35571832	1,1096554	3,4711E-07	1,1096554	0,23551779	0,81380688
VCA0574	1,00836639	-1,4996238	-1,0844227	-1,463E-06	-1,0844227	-0,2354308	0,8138744
VC2115	1,00642416	-0,8488037	-1,0444816	-8,283E-07	-1,0444816	-0,2353787	0,81391482
VCA1090	-1,0685602	-0,3440159	-1,1133775	-3,357E-07	-1,1133775	-0,235302	0,81397438
VC1551	1,25586405	0,26959046	1,14666722	2,6307E-07	1,14666722	0,23507014	0,8141543
VC1933	1,13212056	0,54206505	1,07026845	5,2895E-07	1,07026845	0,23493888	0,81425618
VC1581	1,4072428	0,17790145	1,23040396	1,736E-07	1,23040396	0,23480306	0,8143616
VCA0960	1,07702958	1,26644394	1,02938396	1,2358E-06	1,02938396	0,23455027	0,81455782
VCA0361a	-1,8E+308	-0,0567154	-1,9542881	-5,534E-08	-1,9542881	-0,2344361	0,81464648
torD	1,21683981	0,38048596	1,10116797	3,7128E-07	1,10116797	0,23443471	0,81464752
VC0093	-1,0355345	-0,847796	-1,0609413	-8,273E-07	-1,0609413	-0,2339326	0,81503733
VCA0257	1,23029049	0,40531754	1,09393559	3,9551E-07	1,09393559	0,2335569	0,815329
VCA0601	1,26389848	0,26754367	1,14576311	2,6107E-07	1,14576311	0,23350238	0,81537133
VC0265	-1,0046306	-0,7306934	-1,0508663	-7,13E-07	-1,0508663	-0,2331726	0,81562737
VC2771	1,19450931	31,3899879	1,06008584	3,0631E-05	1,06008584	0,23293719	0,81581019
VC1608	1,14930886	0,80173682	1,09228764	7,8234E-07	1,09228764	0,23272266	0,81597678
VC2031	-1,0310441	-0,4473501	-1,1015669	-4,365E-07	-1,1015669	-0,2323538	0,81626321
VC1968	1,032215	-1,4347506	-1,0305206	-1,4E-06	-1,0305206	-0,2323521	0,81626455
VC1894	-1,0073813	-7,264565	-1,0242456	-7,089E-06	-1,0242456	-0,232281	0,81631975
tRNA-Met-6	-1,0178958	-0,5989225	-1,0617199	-5,844E-07	-1,0617199	-0,2319241	0,81659701
VC0693	-1,0009058	-0,7019778	-1,0524171	-6,85E-07	-1,0524171	-0,2319151	0,81660399
VCA0042	1,08061617	1,47452383	1,03208812	1,4389E-06	1,03208812	0,23146102	0,81695667
VC0147	-1,010547	-2,8244652	-1,0369536	-2,756E-06	-1,0369536	-0,2309847	0,81732667
fliL	-1,0046041	-1,7103141	-1,0305864	-1,669E-06	-1,0305864	-0,2309326	0,81736715
tRNA-Asp-1	1,11722477	2,27864798	1,11777805	2,2235E-06	1,11777805	0,23022019	0,81792069
VC1005	-1,0324995	-1,4211915	-1,077903	-1,387E-06	-1,077903	-0,2300825	0,81802771
VC0196	1,11081947	0,71991545	1,05131837	7,025E-07	1,05131837	0,22941626	0,81854541
VC1015	1,09671344	1,01625328	1,03475879	9,9167E-07	1,03475879	0,22821479	0,81947927
VC0496	1,16612561	0,52658641	1,06799637	5,1385E-07	1,06799637	0,22791092	0,8197155
VC0922	1,13386925	0,53824302	1,06636839	5,2522E-07	1,06636839	0,22773426	0,81985284
VC0072	1,09101415	1,06443158	1,05913016	1,0387E-06	1,05913016	0,22727545	0,82020957
VC1389	-1,0338191	-0,3549052	-1,1019362	-3,463E-07	-1,1019362	-0,2272333	0,82024233
VC2270	1,01417907	-2,0004789	-1,0359718	-1,952E-06	-1,0359718	-0,2272054	0,82026406
VCA0343	1,0396062	-0,8996372	-1,0389792	-8,779E-07	-1,0389792	-0,2271484	0,82030839
VCA0786	1,00935329	-0,7531798	-1,0465793	-7,35E-07	-1,0465793	-0,2267751	0,8205986
VC1911	1,00644181	2,89871986	1,03135818	2,8286E-06	1,03135818	0,22661989	0,82071933
VC1247	1,03998407	11,1432549	1,04369593	1,0874E-05	1,04369593	0,22590738	0,82127346
VCA0380	1,2194315	0,3943673	1,08951329	3,8483E-07	1,08951329	0,22512993	0,8218782
VC0903	1,12260641	0,73418867	1,04685433	7,1643E-07	1,04685433	0,22454286	0,82233493
VCA0096	1,15476471	0,56092491	1,06172293	5,4736E-07	1,06172293	0,2244518	0,82240577
VC0808	1,13484503	0,55818211	1,06166633	5,4468E-07	1,06166633	0,22380275	0,8229108
fadE	1,05499359	-0,3815691	-1,0913448	-3,723E-07	-1,0913448	-0,2236028	0,82306637
VCA0958	1,2396971	0,38173121	1,09124618	3,725E-07	1,09124618	0,22353481	0,8231193

VCA0754	1,16841643	3,29751124	1,17246082	3,2177E-06	1,17246082	0,22297469	0,82355521
VC1746	1,17350468	0,45532238	1,07549844	4,4431E-07	1,07549844	0,22290984	0,82360569
VC0938	1,18534056	0,37541039	1,09184779	3,6633E-07	1,09184779	0,222374	0,82402276
VC1931	1,20628424	0,35108275	1,09817381	3,4259E-07	1,09817381	0,22199504	0,82431775
VCA0720	1,00367114	-0,3521	-1,0972085	-3,436E-07	-1,0972085	-0,2212716	0,82488096
VC2500	1,00362704	-1,1222601	-1,0307784	-1,095E-06	-1,0307784	-0,2211216	0,82499777
VCA0141	1,00317596	-1,0104397	-1,0888171	-9,86E-07	-1,0888171	-0,2206696	0,82534968
fadJ	1,18502651	0,95489538	1,07956728	9,318E-07	1,07956728	0,22013761	0,82576399
VC1766	1,0632076	2,8193129	1,05434644	2,7511E-06	1,05434644	0,219886	0,82595995
ppnK	1,11668523	1,10224772	1,03399836	1,0756E-06	1,03399836	0,21908686	0,8265824
VC1162	-1,0055598	-0,6018212	-1,0544268	-5,873E-07	-1,0544268	-0,2187045	0,82688027
VC0722	-1,018313	-3,8965532	-1,0768928	-3,802E-06	-1,0768928	-0,2186649	0,82691109
VC1213	1,07789692	8,05246632	1,04475248	7,8577E-06	1,04475248	0,21801227	0,82741956
VC1730	1,08857738	4,6950586	1,04753763	4,5815E-06	1,04753763	0,21798179	0,82744331
VCA0146	-1,0307536	-0,2155755	-1,1583052	-2,104E-07	-1,1583052	-0,2177969	0,82758735
VC1179	-1,0015161	-1,5530665	-1,038111	-1,515E-06	-1,038111	-0,2176981	0,82766433
VC1465	1,00537445	-0,7198673	-1,0447091	-7,025E-07	-1,0447091	-0,2173064	0,82796961
VC1429	1,09006038	0,81761558	1,03924047	7,9784E-07	1,03924047	0,21725641	0,82800853
VCA0776	1,15758218	0,59986109	1,05385222	5,8535E-07	1,05385222	0,2172229	0,82803465
VC1856	1,0089186	-1,3941233	-1,0358931	-1,36E-06	-1,0358931	-0,2170618	0,82816022
VC1647	1,2346427	0,35449237	1,09263392	3,4592E-07	1,09263392	0,21697181	0,82823032
VC0595	1,20394775	0,55190653	1,06864241	5,3856E-07	1,06864241	0,21689661	0,82828892
VCA0926	1,14793086	0,4752444	1,06775047	4,6375E-07	1,06775047	0,21613632	0,8288815
VC0317	1,12898072	0,62635729	1,05098189	6,112E-07	1,05098189	0,21612321	0,82889172
cpdB	1,01482976	-3,1434856	-1,0922714	-3,067E-06	-1,0922714	-0,2160086	0,82898107
VCA0085	1,23198142	0,35963099	1,08995515	3,5093E-07	1,08995515	0,21549371	0,82938242
tRNA-Met-7	-1,0802881	-3,385983	-1,063794	-3,304E-06	-1,063794	-0,2153871	0,82946554
VCA0935	1,1759352	1,20991162	1,12132208	1,1806E-06	1,12132208	0,21532986	0,82951016
VCA0632	1,16425532	0,67634746	1,05519531	6,5999E-07	1,05519531	0,21529509	0,82953727
VC0995	1,14381856	10,9429088	1,08920616	1,0678E-05	1,08920616	0,21516578	0,82963808
tRNA-Ala-3	-1,056794	-1,2102147	-1,1207169	-1,181E-06	-1,1207169	-0,2146841	0,83001365
VC1269	1,1112723	5,35789245	1,07711858	5,2283E-06	1,07711858	0,21453482	0,83013003
VC0847	1,15452883	1,42985607	1,06734239	1,3953E-06	1,06734239	0,21449641	0,83015997
VC1777	-1,0015674	-6,923622	-1,1729238	-6,756E-06	-1,1729238	-0,2143339	0,83028666
VC1788	1,10931833	0,58089397	1,05395622	5,6684E-07	1,05395622	0,21396196	0,83057673
dinG	1,09096826	1,52229603	1,04945034	1,4855E-06	1,04945034	0,2137992	0,83070366
VC0498	-1,0202586	-0,3388434	-1,0941451	-3,306E-07	-1,0941451	-0,2137748	0,83072267
VC1909	-1,0236843	-2,691216	-1,0191079	-2,626E-06	-1,0191079	-0,213146	0,83121308
VCA0895	1,1932165	0,44937618	1,06963975	4,3851E-07	1,06963975	0,21298465	0,83133895
VC1081	1,17985261	3,48202873	1,09662253	3,3978E-06	1,09662253	0,21209733	0,83203112
VCA0074	1,09880283	0,79882007	1,0601886	7,795E-07	1,0601886	0,21201355	0,83209647
VCA1015	1,12578758	0,7253053	1,05795327	7,0776E-07	1,05795327	0,21163808	0,8323894
VCA0727	1,00394598	-0,5556954	-1,0546757	-5,423E-07	-1,0546757	-0,2106235	0,83318106
VCA1054	1,13240257	0,3468403	1,08904229	3,3845E-07	1,08904229	0,21059634	0,83320227
VC1077	1,09135328	13,7850064	1,06200594	1,3452E-05	1,06200594	0,21021116	0,83350288

VC0910	-1,0681526	-63,315412	-1,2281453	-6,178E-05	-1,2281453	-0,2101304	0,83356588
VCA0690	1,26635338	0,19827384	1,1602095	1,9348E-07	1,1602095	0,2100341	0,83364106
VC2303	-1,0792471	-3,1653667	-1,0932535	-3,089E-06	-1,0932535	-0,2100288	0,83364524
prmA	1,09104513	0,82690604	1,05834588	8,069E-07	1,05834588	0,2098449	0,83378873
VCA0961	1,16688835	0,45395177	1,06595936	4,4297E-07	1,06595936	0,20851837	0,83482425
apaG	1,08847964	1,34770694	1,03368837	1,3151E-06	1,03368837	0,20743639	0,83566907
VC2208	1,04809045	-1,1826647	-1,0438756	-1,154E-06	-1,0438756	-0,2071452	0,83589647
VCA0080	1,10801545	0,72991078	1,03993233	7,1225E-07	1,03993233	0,20703983	0,83597876
VC0084	1,02807955	-1,0260791	-1,0281628	-1,001E-06	-1,0281628	-0,2067499	0,8362052
qrr4	1,02916476	-1,6394345	-1,054573	-1,6E-06	-1,054573	-0,2066327	0,83629676
VCA0863	1,03485873	-0,5739468	-1,0671228	-5,601E-07	-1,0671228	-0,2066216	0,83630539
VC2504	1,24021682	2,09201871	1,09768276	2,0414E-06	1,09768276	0,20655638	0,83635634
VC2094	-1,0105683	-4,0953702	-1,0702937	-3,996E-06	-1,0702937	-0,2064998	0,83640055
VC1325	1,23855287	62,039306	1,1437537	6,0538E-05	1,1437537	0,20642595	0,83645822
VC2557	1,18713901	1,35537525	1,05773015	1,3226E-06	1,05773015	0,20638662	0,83648893
VC0819	1,1894073	0,35560847	1,08295933	3,4701E-07	1,08295933	0,20583642	0,8369187
VC0463	1,0368637	-0,5887202	-1,048451	-5,745E-07	-1,048451	-0,2043884	0,83805003
VC2082	1,12540511	0,57834431	1,04932655	5,6435E-07	1,04932655	0,20435779	0,83807392
VC1197	-1,0331247	-7,4385864	-1,0697832	-7,259E-06	-1,0697832	-0,2043233	0,83810086
VC0129	-1,0031534	-1,1429507	-1,0246107	-1,115E-06	-1,0246107	-0,2041624	0,83822662
VC1017	1,11512968	0,75594642	1,03723378	7,3766E-07	1,03723378	0,20359104	0,83867311
VC1305	1,00911874	-1,7325801	-1,0476074	-1,691E-06	-1,0476074	-0,2031396	0,83902592
VC0678	1,25669663	17,6372009	1,04102428	1,7211E-05	1,04102428	0,20310646	0,83905184
VCA0813	1,15212218	0,41846304	1,06779648	4,0834E-07	1,06779648	0,20288045	0,83922848
tRNA-Ile-3	1,09188816	19,4596677	1,08294893	1,8989E-05	1,08294893	0,20258675	0,83945806
VC2489	1,22246351	0,40865117	1,06921535	3,9877E-07	1,06921535	0,20250542	0,83952162
VCA0426	1,06342329	2,3311096	1,04659751	2,2747E-06	1,04659751	0,20217949	0,83977641
VC1442	1,12790525	7,26855894	1,07011846	7,0927E-06	1,07011846	0,20200342	0,83991407
VC1153	1,13381558	14,8357791	1,10025135	1,4477E-05	1,10025135	0,20144817	0,84034816
VC1083	1,01303534	-1,9744801	-1,0769273	-1,927E-06	-1,0769273	-0,2010238	0,84067998
VC1507	1,12673001	1,98044326	1,05308368	1,9325E-06	1,05308368	0,20057805	0,84102853
VCA0788	1,01583771	-2,8890559	-1,0538095	-2,819E-06	-1,0538095	-0,2005569	0,84104504
VC2599	-1,0226596	-2,5771654	-1,0288867	-2,515E-06	-1,0288867	-0,1998977	0,84156062
VC1120	1,0495648	-0,9271377	-1,0643969	-9,047E-07	-1,0643969	-0,1998344	0,84161012
ugpC	1,37766536	0,16563027	1,17476926	1,6162E-07	1,17476926	0,19982813	0,841615
VC0957	-1,0535892	-22,084593	-1,1037333	-2,155E-05	-1,1037333	-0,1992363	0,84207788
lplA	1,11062453	0,49977741	1,06071638	4,8769E-07	1,06071638	0,19895213	0,8423002
VCA0222	1,21630232	0,26319854	1,10520408	2,5683E-07	1,10520408	0,19864247	0,84254243
VC0887	1,0211596	-0,4961911	-1,0542521	-4,842E-07	-1,0542521	-0,1982753	0,84282968
VC1664	1,00882379	-0,5966456	-1,0448252	-5,822E-07	-1,0448252	-0,1980865	0,84297738
VCA1020	1,02611929	-1,175428	-1,0223736	-1,147E-06	-1,0223736	-0,1975183	0,84342195
VC1560	-1,0043598	-0,9595732	-1,0560516	-9,364E-07	-1,0560516	-0,197509	0,84342924
VC1782	1,03296903	-2,769009	-1,1182384	-2,702E-06	-1,1182384	-0,1973998	0,84351473
VC1660a	1,03305997	-0,5288	-1,0527624	-5,16E-07	-1,0527624	-0,1972064	0,84366606
VC1897	-1,0366493	-3,1695158	-1,0467875	-3,093E-06	-1,0467875	-0,197021	0,84381111

envZ	1,04882864	-0,7064887	-1,0372421	-6,894E-07	-1,0372421	-0,1968399	0,84395284
cyaA	1,04377338	13,3769431	1,12432929	1,3053E-05	1,12432929	0,19656562	0,84416749
VC1870	1,02348255	-0,4069124	-1,0651245	-3,971E-07	-1,0651245	-0,1962057	0,84444916
VCA0007	1,09351381	1,82945043	1,05648274	1,7852E-06	1,05648274	0,19603369	0,8445838
VC1156	1,02621276	-3,0740796	-1,0868656	-3E-06	-1,0868656	-0,1957603	0,8447978
VCA0988	-1,0609283	-0,2352499	-1,1146457	-2,296E-07	-1,1146457	-0,1956077	0,84491725
VC1422a	1,07256426	1,07469648	1,02389496	1,0487E-06	1,02389496	0,19510678	0,84530936
VCA1100	1,22291482	0,20645342	1,13084228	2,0146E-07	1,13084228	0,19501608	0,84538037
VC0099	1,09105429	0,92902157	1,02762212	9,0655E-07	1,02762212	0,19485689	0,84550499
VCA0544	1,19963729	1,27325762	1,06773314	1,2425E-06	1,06773314	0,1947541	0,84558546
VCA0297	1,10553894	0,48713746	1,0537879	4,7535E-07	1,0537879	0,19424462	0,84598435
VCA0649	1,36282582	0,12794008	1,21800303	1,2485E-07	1,21800303	0,19422909	0,84599651
VCA0854	1,03871532	-0,1503692	-1,181659	-1,467E-07	-1,181659	-0,1938096	0,84632497
VC0095	1,12232068	0,78469677	1,0321284	7,6571E-07	1,0321284	0,19292379	0,84701866
VC1229	1,25228176	0,23233452	1,11246679	2,2671E-07	1,11246679	0,19263499	0,84724485
VC1598	1,1347298	0,64395101	1,03916711	6,2837E-07	1,03916711	0,19263045	0,8472484
purT	-1,0251359	-0,9393093	-1,0698711	-9,166E-07	-1,0698711	-0,1926121	0,8472628
VCA1092	-1,0519843	-0,2723947	-1,0951129	-2,658E-07	-1,0951129	-0,192609	0,84726517
lspA	-1,0099776	-2,040003	-1,0432503	-1,991E-06	-1,0432503	-0,1925313	0,84732606
cobS	1,00922463	-1,1930502	-1,0767243	-1,164E-06	-1,0767243	-0,1922264	0,84756487
VC1006	1,09980941	2,14552994	1,06768391	2,0936E-06	1,06768391	0,19107196	0,84846923
VC2318	-1,0234868	-1,0074072	-1,047328	-9,83E-07	-1,047328	-0,1906995	0,84876101
alr (NC_002505 387951..389119)	1,02870496	-0,9652087	-1,0254223	-9,419E-07	-1,0254223	-0,1906466	0,84880245
VCA0102	1,00207836	-0,9421495	-1,0260729	-9,194E-07	-1,0260729	-0,1906351	0,84881149
VC2162	1,01363094	-0,9603057	-1,0254535	-9,371E-07	-1,0254535	-0,1902771	0,84909203
VC0728	1,04863811	-0,9734418	-1,0391553	-9,499E-07	-1,0391553	-0,190188	0,84916184
VCA0505	1,02088302	-1,6642658	-1,0387852	-1,624E-06	-1,0387852	-0,1899018	0,84938608
VC2111	-1,0059936	-1,2293466	-1,0314795	-1,2E-06	-1,0314795	-0,1898012	0,84946491
VCA1010	1,05290833	-0,3617054	-1,0683557	-3,53E-07	-1,0683557	-0,1893713	0,84980186
VC2310	1,16886065	0,57800914	1,0893421	5,6403E-07	1,0893421	0,18934438	0,84982292
VC0851	1,07574878	4,29258671	1,03718095	4,1887E-06	1,03718095	0,18928457	0,84986979
VC1997	1,05084059	-1,8901315	-1,048532	-1,844E-06	-1,048532	-0,188757	0,85028332
VCA0464	1,0738725	0,84706203	1,02833877	8,2657E-07	1,02833877	0,18842776	0,85054135
VC1310	1,07018249	1,01293564	1,02778923	9,8843E-07	1,02778923	0,18824721	0,85068287
VC0992	1,02117934	-0,6185694	-1,0389216	-6,036E-07	-1,0389216	-0,1882145	0,8507085
VC0868	-1,0548642	-0,2215879	-1,1122188	-2,162E-07	-1,1122188	-0,1879306	0,85093106
VC1953	1,20334631	0,22232959	1,11178542	2,1695E-07	1,11178542	0,18790027	0,85095484
VC2550	1,0830616	3,23277131	1,05983096	3,1546E-06	1,05983096	0,18787878	0,85097169
VCA1019	1,23549765	0,27139686	1,09043831	2,6483E-07	1,09043831	0,1876814	0,85112642
VC1918	1,04068207	5,17278133	1,03539048	5,0476E-06	1,03539048	0,18755388	0,85122639
VC1122	-1,0411371	-2,8609923	-1,0646644	-2,792E-06	-1,0646644	-0,1874878	0,8512782
VC2455	1,02387995	-0,435227	-1,0552641	-4,247E-07	-1,0552641	-0,1873735	0,85136782
mraY	1,01437587	-1,0207317	-1,0231254	-9,96E-07	-1,0231254	-0,1870935	0,85158731
VC2677	1,02557275	-6,3536084	-1,0740335	-6,2E-06	-1,0740335	-0,1869374	0,85170971
VC0303	-1,0055273	-0,8646634	-1,0730391	-8,437E-07	-1,0730391	-0,1867281	0,85187381

VCA0766	1,01517471	-0,4955817	-1,0479864	-4,836E-07	-1,0479864	-0,1866449	0,85193905
VC0779	1,01751053	-0,3482103	-1,0687867	-3,398E-07	-1,0687867	-0,1863704	0,85215435
VC1003	1,06945462	1,29199684	1,03289917	1,2607E-06	1,03289917	0,18602533	0,8524249
ectC	-1,0063159	-0,4245026	-1,0637236	-4,142E-07	-1,0637236	-0,1855295	0,85281372
VCA0990	1,22175677	0,33956107	1,06987877	3,3135E-07	1,06987877	0,18544743	0,85287812
VC0038	1,07381666	7,24900306	1,05712222	7,0736E-06	1,05712222	0,18486237	0,85333699
VC0481	1,07557407	2,17913371	1,06089594	2,1264E-06	1,06089594	0,18453229	0,8535959
VCA0930	1,09133625	0,67628901	1,03409136	6,5993E-07	1,03409136	0,18440301	0,85369732
VC0235	1,07541464	1,79080595	1,03735536	1,7475E-06	1,03735536	0,18433707	0,85374904
VCA0025	1,15554684	5,86854593	1,1073906	5,7266E-06	1,1073906	0,18421645	0,85384366
zntA	1,06807989	1,87693097	1,04391745	1,8315E-06	1,04391745	0,18408509	0,85394671
VC2695	1,02423289	-2,0224743	-1,0430848	-1,974E-06	-1,0430848	-0,1838676	0,85411733
VCA0615	1,04919509	-0,3505139	-1,0662691	-3,42E-07	-1,0662691	-0,183644	0,85429278
glgC (NC_002505 1865312..1866570)	-1,0060319	-5,876606	-1,0587399	-5,734E-06	-1,0587399	-0,183461	0,85443635
VCA0484	1,03989538	-1,3490184	-1,0567694	-1,316E-06	-1,0567694	-0,1832844	0,85457488
VC1475	-1,027444	-0,1681044	-1,1421207	-1,64E-07	-1,1421207	-0,1829183	0,85486215
VC1433	-1,027226	-4,653088	-1,0576601	-4,541E-06	-1,0576601	-0,1827978	0,85495665
VC2300	1,05234003	-1,0727664	-1,0275147	-1,047E-06	-1,0275147	-0,1825207	0,85517412
VC2463	-1,0226144	-3,2928043	-1,0248548	-3,213E-06	-1,0248548	-0,1824459	0,85523281
VC1867	1,0724246	-1,1399126	-1,0493553	-1,112E-06	-1,0493553	-0,18222	0,85541009
VC1875	1,09668024	2,69640918	1,05745567	2,6312E-06	1,05745567	0,18154755	0,85593782
VC0755	1,02917193	-3,8758841	-1,0474337	-3,782E-06	-1,0474337	-0,1814906	0,85598256
tRNA-Gly-3	-1,0767309	-1,8883733	-1,0632858	-1,843E-06	-1,0632858	-0,1809837	0,85638035
VC2631	-1,007335	-0,2821796	-1,0803526	-2,754E-07	-1,0803526	-0,1808239	0,85650583
obgE	1,06157936	3,86742867	1,06510241	3,7739E-06	1,06510241	0,1806522	0,85664059
VC1057	-1,0012345	-0,472146	-1,0470958	-4,607E-07	-1,0470958	-0,1805191	0,85674508
ubiB	1,06254761	0,99231233	1,02464166	9,6831E-07	1,02464166	0,18033086	0,85689283
VCA0770	1,15375277	16,4725941	1,05467187	1,6074E-05	1,05467187	0,18017261	0,85701707
tRNA-Leu-2	-1,1657615	-4,243009	-1,1242803	-4,14E-06	-1,1242803	-0,179908	0,85722484
VC2017	1,08050997	0,69061164	1,03169919	6,739E-07	1,03169919	0,17979461	0,85731382
VCA0594	1,06227964	22,7262581	1,08986263	2,2176E-05	1,08986263	0,17976435	0,85733759
VCA0422	1,07403966	0,69978055	1,03280547	6,8285E-07	1,03280547	0,17940938	0,85761628
VC0582	1,00825927	-1,1437446	-1,0235089	-1,116E-06	-1,0235089	-0,1790098	0,85792997
VCA0685	1,3106434	9,80748366	1,24162143	9,5702E-06	1,24162143	0,1786536	0,85820971
VCA0604	1,02629685	-0,143705	-1,1595102	-1,402E-07	-1,1595102	-0,1784487	0,85837062
VCA0027	1,16955182	0,43227064	1,07102187	4,2181E-07	1,07102187	0,1778552	0,8588367
VC2355	1,06748912	1,53001038	1,02351539	1,493E-06	1,02351539	0,17728958	0,85928094
VCA0265	1,08229177	4,99372025	1,04741255	4,8729E-06	1,04741255	0,17682146	0,85964864
VCA0703	1,02253484	-0,3644725	-1,058359	-3,557E-07	-1,058359	-0,176071	0,86023817
VC0930	1,11717174	0,40938328	1,05120124	3,9948E-07	1,05120124	0,17509088	0,86100823
VC1188	1,13814715	2,53870899	1,06106737	2,4773E-06	1,06106737	0,17398715	0,86187557
VCA0733	1,2101321	0,25985577	1,08002966	2,5357E-07	1,08002966	0,17318819	0,86250352
VCA0275	1,28074482	0,16387834	1,12977191	1,5991E-07	1,12977191	0,17307944	0,86258899
VCA0886	-1,0713401	-5,0421087	-1,0591282	-4,92E-06	-1,0591282	-0,1730619	0,8626028
VC0772	-1,0133384	-0,2288404	-1,0906933	-2,233E-07	-1,0906933	-0,1725718	0,862988

VCA1016	1,12651199	0,52460951	1,05578997	5,1192E-07	1,05578997	0,17242078	0,86310674
VC2357	1,02364906	-5,8853496	-1,0676444	-5,743E-06	-1,0676444	-0,1724099	0,86311533
VCA0455	1,10383573	1,17207	1,04212496	1,1437E-06	1,04212496	0,17228949	0,86320996
VC1750	1,03479674	-0,6997767	-1,0286377	-6,828E-07	-1,0286377	-0,1721522	0,86331789
16Sc	1,0175049	7,34154833	1,07211283	7,1639E-06	1,07211283	0,17210345	0,86335621
VC2527	-1,0434552	-6,5981085	-1,0399703	-6,438E-06	-1,0399703	-0,1720411	0,8634052
VC1341	-1,0121232	-2,3348716	-1,0437905	-2,278E-06	-1,0437905	-0,1714943	0,86383512
VC0404	1,05357505	1,24400693	1,02501246	1,2139E-06	1,02501246	0,17128465	0,86399996
VC1665	1,04753105	-0,3217486	-1,0626438	-3,14E-07	-1,0626438	-0,1712171	0,86405306
VCA0684	-1,1454543	-4,3493307	-1,2023684	-4,244E-06	-1,2023684	-0,170786	0,86439206
VCA1099	1,41013427	0,12084917	1,17431074	1,1793E-07	1,17431074	0,17048411	0,86462944
VC1438	-1,0232228	-2,9492457	-1,0576467	-2,878E-06	-1,0576467	-0,170271	0,86479699
VCA0977	1,16650629	0,33270724	1,0598267	3,2466E-07	1,0598267	0,17026491	0,86480181
VCA0774	-1,0123428	-10,570813	-1,0799308	-1,032E-05	-1,0799308	-0,1702368	0,86482391
VC0349	1,04560037	-6,2886728	-1,0169195	-6,137E-06	-1,0169195	-0,1697253	0,86522618
VC0490	1,03677005	15,571902	1,06065995	1,5195E-05	1,06065995	0,16927549	0,86557996
VCA1094	-1,0577141	-0,2899554	-1,0859538	-2,829E-07	-1,0859538	-0,1685964	0,86611413
VC0937	1,03429551	-0,3655651	-1,0530438	-3,567E-07	-1,0530438	-0,1683305	0,86632329
VC1289	1,08115828	0,97404044	1,01951587	9,5048E-07	1,01951587	0,16804674	0,86654651
tRNA-Ile-2	1,09600204	18,1635081	1,0768361	1,7724E-05	1,0768361	0,16800759	0,86657732
VC2563	1,12583967	0,43328886	1,04415541	4,2281E-07	1,04415541	0,16756624	0,86692454
VC2288	1,0701276	1,67927099	1,03511494	1,6386E-06	1,03511494	0,16717043	0,86723596
VC2613	1,08126982	-0,8253198	-1,0302967	-8,054E-07	-1,0302967	-0,1668847	0,86746077
VCA0714	1,10458439	0,43940376	1,04311976	4,2877E-07	1,04311976	0,16679613	0,86753047
VC0032	1,23555132	0,15387651	1,12680044	1,5015E-07	1,12680044	0,16589912	0,86823635
hemC	1,05571655	-0,631877	-1,0293961	-6,166E-07	-1,0293961	-0,1657078	0,86838692
VC0704	1,14103314	0,41334158	1,04528049	4,0334E-07	1,04528049	0,16569	0,86840092
VCA0353	1,08613652	0,41749393	1,04478191	4,0739E-07	1,04478191	0,16562106	0,86845518
VC0979	1,1567512	0,65759182	1,04531564	6,4168E-07	1,04531564	0,16546367	0,86857905
VC1097	-1,0013439	11,7935135	1,04888627	1,1508E-05	1,04888627	0,16479087	0,86910859
VC1991	1,16405913	3,47310233	1,08674021	3,3891E-06	1,08674021	0,16438255	0,86943001
VC0070	1,05034831	2,98421526	1,03599481	2,912E-06	1,03599481	0,16433813	0,86946497
hemH (NC_002505 1051232..1052247)	1,11892174	0,75148768	1,02418901	7,3331E-07	1,02418901	0,16413899	0,86962173
VC1612	1,05400338	-1,1416215	-1,0588921	-1,114E-06	-1,0588921	-0,1641207	0,8696361
VCA0255	-1,0012279	-0,1798231	-1,1051035	-1,755E-07	-1,1051035	-0,1641178	0,86963845
VCA0830	1,1572868	0,36276354	1,05065516	3,5399E-07	1,05065516	0,16396062	0,86976215
gpsA	1,12597679	0,60201249	1,02992459	5,8745E-07	1,02992459	0,16317057	0,87038414
gcp	1,04182948	1,46547119	1,02389365	1,43E-06	1,02389365	0,16283516	0,87064823
VC1337	1,05713255	-0,3636325	-1,0918124	-3,548E-07	-1,0918124	-0,1627571	0,87070968
VCA0375	1,15304463	0,2389874	1,07669709	2,3321E-07	1,07669709	0,16272403	0,87073573
VC1171	1,0392876	-0,4896229	-1,0365391	-4,778E-07	-1,0365391	-0,1623406	0,87103768
VC2467	1,13603183	-21,352275	-1,0268176	-2,084E-05	-1,0268176	-0,1620292	0,87128284
recF	-1,0127494	-3,5597431	-1,0346244	-3,474E-06	-1,0346244	-0,1619939	0,87131065
VCA0271	-1,0124286	-2,1183029	-1,0239346	-2,067E-06	-1,0239346	-0,1617651	0,87149084
VC0268	1,05021174	2,19375945	1,03872944	2,1407E-06	1,03872944	0,16134402	0,87182247

VC0947	1,04607591	4,22588243	1,01419815	4,1237E-06	1,01419815	0,16055835	0,87244127
VCA0211	1,04374134	-0,3089261	-1,0570497	-3,015E-07	-1,0570497	-0,1603221	0,87262733
VC1550	1,04409096	-0,1637574	-1,1101694	-1,598E-07	-1,1101694	-0,1601524	0,87276106
VC1603	1,00482824	-3,6655205	-1,043947	-3,577E-06	-1,043947	-0,1601027	0,8728002
VCA0751	1,14769808	1,79608924	1,06474321	1,7526E-06	1,06474321	0,15972842	0,87309502
VC0379	1,13352289	0,47839525	1,04229779	4,6682E-07	1,04229779	0,15971289	0,87310725
VCA0153	1,2056028	0,2758877	1,0635987	2,6921E-07	1,0635987	0,15971263	0,87310745
VC2531	1,04776417	5,61381941	1,03645359	5,478E-06	1,03645359	0,15862764	0,87396226
VC1976	1,12457655	0,49609352	1,0342524	4,8409E-07	1,0342524	0,15830281	0,8742182
VCA0847	1,15144283	0,26396614	1,06522201	2,5758E-07	1,06522201	0,15814277	0,8743443
VCA0865	1,19815597	0,23906542	1,07223109	2,3328E-07	1,07223109	0,15811124	0,87436915
ribB	-1,0166907	-1,0879535	-1,0463672	-1,062E-06	-1,0463672	-0,1579476	0,87449811
VC0142	1,02801574	-3,8237036	-1,0489192	-3,731E-06	-1,0489192	-0,1579184	0,87452113
VCA0772	1,16726687	0,24720704	1,0695144	2,4123E-07	1,0695144	0,15783197	0,87458921
VC0392	1,13369757	0,64547179	1,03985427	6,2986E-07	1,03985427	0,15775021	0,87465364
VC1360	1,02750506	-0,2306844	-1,0742511	-2,251E-07	-1,0742511	-0,1573951	0,87493349
VC2339	1,21176953	0,22445807	1,07634814	2,1903E-07	1,07634814	0,15735409	0,8749658
VC0861	1,05315494	-0,272505	-1,0623526	-2,659E-07	-1,0623526	-0,1572153	0,8750752
VC2395	1,03052909	-0,4668481	-1,035826	-4,556E-07	-1,035826	-0,156993	0,87525036
VC0770	-1,1613843	-13,66746	-1,0890284	-1,334E-05	-1,0890284	-0,1565309	0,87561461
VCA0228	1,15132865	0,33306191	1,0498169	3,25E-07	1,0498169	0,15583155	0,87616581
recC	1,11726624	0,45665346	1,03564983	4,4561E-07	1,03564983	0,15489377	0,87690507
VC1784	-1,0328942	-2,6859476	-1,1038285	-2,621E-06	-1,1038285	-0,154614	0,87712567
folE	1,07001789	0,62842742	1,02679271	6,1322E-07	1,02679271	0,15451907	0,87720048
tRNA-Leu-12	1,05593562	5,71983358	1,08064543	5,5815E-06	1,08064543	0,15390868	0,87768175
VC2064	1,08199927	7,68181197	1,0157057	7,496E-06	1,0157057	0,15370236	0,87784443
VC0301	1,08063482	-0,1318567	-1,1268078	-1,287E-07	-1,1268078	-0,1535751	0,87794477
VC0943	1,06358871	0,75926605	1,03830312	7,409E-07	1,03830312	0,15351268	0,877994
VC1779	1,02091306	-8,1276844	-1,1065145	-7,931E-06	-1,1065145	-0,1534826	0,87801773
VC0628	1,10745388	0,74081735	1,03788183	7,229E-07	1,03788183	0,15252275	0,87877465
VC0721	1,09486676	0,39739005	1,03976902	3,8778E-07	1,03976902	0,15245906	0,87882488
VC1359	1,02005914	-0,312944	-1,0505704	-3,054E-07	-1,0505704	-0,1521619	0,87905923
tRNA-Leu-3	-1,0879455	-2,2756567	-1,0793344	-2,221E-06	-1,0793344	-0,1518779	0,87928325
VC0723	1,05011014	-0,9339006	-1,0352452	-9,113E-07	-1,0352452	-0,1517467	0,87938675
rpmA	-1,0622017	-51,077168	-1,0359105	-4,984E-05	-1,0359105	-0,1516713	0,87944621
VCA0640	1,51374775	0,10867267	1,14952694	1,0604E-07	1,14952694	0,15059454	0,88029557
VC1284	1,18006653	0,32046994	1,04795877	3,1272E-07	1,04795877	0,15004762	0,88072705
VCA0739	1,01306465	-0,4759267	-1,0356984	-4,644E-07	-1,0356984	-0,1497911	0,88092944
VC0051	1,09097435	0,444017	1,03816926	4,3328E-07	1,03816926	0,14896492	0,88158132
VCA0993	1,04773995	-0,1619686	-1,0956417	-1,581E-07	-1,0956417	-0,1489161	0,88161986
VC1161	1,01875292	-0,4800816	-1,0312328	-4,685E-07	-1,0312328	-0,148815	0,88169959
VC1663	1,01179698	-5,2017281	-1,0171392	-5,076E-06	-1,0171392	-0,1483394	0,88207492
VCA0619	1,05414896	-0,8387715	-1,034098	-8,185E-07	-1,034098	-0,148121	0,88224725
VC2223	1,06839539	0,57719821	1,02558064	5,6323E-07	1,02558064	0,14787982	0,88243762
VC0680	1,00261342	-0,6506599	-1,0334093	-6,349E-07	-1,0334093	-0,1478094	0,88249317

VCA0274	-1,0362596	-2,8107697	-1,0814662	-2,743E-06	-1,0814662	-0,1477068	0,88257415
VCA0562	1,05370363	4,29946809	1,02574086	4,1955E-06	1,02574086	0,14758839	0,88266762
recA	1,04885668	5,23117596	1,01435581	5,1046E-06	1,01435581	0,14754094	0,88270507
VC2646	1,03646949	7,60203649	1,11755093	7,4181E-06	1,11755093	0,14710409	0,88304987
VCA0791	-1,1561883	-2,8887399	-1,0757242	-2,819E-06	-1,0757242	-0,1466078	0,8834416
VC1203	1,05430062	-0,7902801	-1,0907981	-7,712E-07	-1,0907981	-0,1465773	0,88346567
VC0501	1,15023955	0,58211302	1,04569767	5,6803E-07	1,04569767	0,14615268	0,88380087
VC2603	1,13080321	2,86809553	1,06344734	2,7987E-06	1,06344734	0,14553786	0,88428623
VC0901	1,07369157	0,56011623	1,0254504	5,4657E-07	1,0254504	0,14530847	0,88446734
VC0858	1,13306495	0,2234098	1,06470237	2,1801E-07	1,06470237	0,14492519	0,88476995
VCA0663	1,09968338	1,07889285	1,03393481	1,0528E-06	1,03393481	0,1446733	0,88496883
VC2702	1,02278999	-2,8639806	-1,0499577	-2,795E-06	-1,0499577	-0,1446703	0,88497121
VCA0037	1,06789761	3,17707684	1,04860895	3,1002E-06	1,04860895	0,14417081	0,8853656
VC2256	-1,0081543	-1,4224219	-1,0269227	-1,388E-06	-1,0269227	-0,144166	0,88536942
VC1590	1,06442722	0,83172067	1,08863684	8,116E-07	1,08863684	0,14406559	0,88544869
VC0522	1,04630868	2,89243531	1,02854533	2,8225E-06	1,02854533	0,14373101	0,88571289
VCA1067	1,11808805	0,63512538	1,04342207	6,1976E-07	1,04342207	0,14345802	0,88592847
VC2525	-1,0386655	-6,5276626	-1,034981	-6,37E-06	-1,034981	-0,1431189	0,8861963
VC0023	1,02336458	-2,5298171	-1,048692	-2,469E-06	-1,048692	-0,1423862	0,88677499
VCA0526	-1,0182952	-0,403894	-1,0512407	-3,941E-07	-1,0512407	-0,1422497	0,88688274
VCA0903	1,15737392	0,74206305	1,05653893	7,2411E-07	1,05653893	0,14214135	0,88696836
VC0929	1,1093423	0,28338432	1,05165866	2,7653E-07	1,05165866	0,14202888	0,8870572
VCA0825	1,22311317	0,20158769	1,06899309	1,9671E-07	1,06899309	0,14200922	0,88707273
VC0510	1,01637235	-0,3247331	-1,0419529	-3,169E-07	-1,0419529	-0,1414764	0,88749364
VCA1098	1,2585972	0,13705271	1,10197589	1,3374E-07	1,10197589	0,14123412	0,887685
VC2444	1,02530736	-0,4642717	-1,0333241	-4,53E-07	-1,0333241	-0,1406779	0,88812443
VC1074	1,14242194	0,3197514	1,05501685	3,1202E-07	1,05501685	0,1406032	0,88818343
tRNA-Lys-1	1,01560439	-0,5016561	-1,0330113	-4,895E-07	-1,0330113	-0,1403307	0,88839875
nudE	1,08781796	0,75126439	1,02454557	7,3309E-07	1,02454557	0,14000175	0,88865861
VCA0233	1,05394473	-0,2098073	-1,0636186	-2,047E-07	-1,0636186	-0,1392993	0,88921363
VCA0322	1,01089999	-2,1399363	-1,0388969	-2,088E-06	-1,0388969	-0,1392778	0,88923061
VCA0276	-1,0272639	-3,680673	-1,0479087	-3,592E-06	-1,0479087	-0,1384174	0,88991055
wecC	1,16043668	0,17296676	1,07586036	1,6878E-07	1,07586036	0,13770535	0,89047329
VC1191	1,11567801	0,72774724	1,04453217	7,1014E-07	1,04453217	0,13747673	0,890654
VC0525	1,08470564	0,45264731	1,02822171	4,417E-07	1,02822171	0,13746053	0,89066679
queA	1,10492771	0,62097571	1,04196239	6,0595E-07	1,04196239	0,13715885	0,89090524
VCA0406	1,12120025	1,424669	1,04437383	1,3902E-06	1,04437383	0,13709965	0,89095204
VC2045	1,17089133	20,8494206	1,03562723	2,0345E-05	1,03562723	0,1368782	0,89112707
VCA0704	1,13558748	0,25637596	1,04983373	2,5017E-07	1,04983373	0,13674227	0,89123452
VC1778	1,05267791	-3,23578	-1,0706025	-3,158E-06	-1,0706025	-0,1367163	0,89125504
VC2712	-1,0020152	-0,4558764	-1,0502541	-4,448E-07	-1,0502541	-0,1365573	0,89138073
VC1392	1,07306208	0,3713014	1,03366869	3,6232E-07	1,03366869	0,13579986	0,89197951
VC0024	1,02490453	-0,4531606	-1,027368	-4,422E-07	-1,027368	-0,1354707	0,89223971
fieF	1,09748371	0,58412514	1,02089069	5,6999E-07	1,02089069	0,13459363	0,89293319
VC0224	1,08715916	0,54273767	1,02230358	5,2961E-07	1,02230358	0,13400621	0,89339766

VCA0345	1,05827959	4,68171772	1,04918649	4,5685E-06	1,04918649	0,13387151	0,89350418
tRNA-Thr-3	-1,0924213	-8,1531847	-1,0467388	-7,956E-06	-1,0467388	-0,1338491	0,8935219
VC2043	1,11093132	0,32623116	1,03701923	3,1834E-07	1,03701923	0,13336485	0,89390485
VCA0209	1,10011863	0,34152823	1,03519042	3,3327E-07	1,03519042	0,13310236	0,89411243
hisS	1,00906446	-1,7467667	-1,0209191	-1,705E-06	-1,0209191	-0,1326541	0,89446694
VCA0707	1,04686685	-0,0768725	-1,1646395	-7,501E-08	-1,1646395	-0,1324404	0,89463599
VC0182	1,03622389	1,70655422	1,0306538	1,6653E-06	1,0306538	0,13235777	0,89470132
VC2647	1,1597242	28,1049565	1,05128395	2,7425E-05	1,05128395	0,13226625	0,89477371
VC0088	1,07508108	0,58769592	1,01977805	5,7348E-07	1,01977805	0,13139634	0,89546179
tRNA-Gln-1	-1,1248187	-12,12429	-1,0840453	-1,183E-05	-1,0840453	-0,1308885	0,89586351
VCA0350	1,14948373	1,21103483	1,05165025	1,1817E-06	1,05165025	0,13085537	0,89588972
VCA0408	1,1393611	0,72576203	1,04069524	7,082E-07	1,04069524	0,13065007	0,89605213
tRNA-Ala-2	-1,0503112	-2,1392915	-1,0436371	-2,088E-06	-1,0436371	-0,1304501	0,89621038
VCA0942	1,08650146	0,78585883	1,02756231	7,6685E-07	1,02756231	0,12964156	0,89685003
VC1687	1,10971457	1,5841644	1,05666937	1,5458E-06	1,05666937	0,12875557	0,89755107
VC2548	1,06187671	0,67074802	1,01644183	6,5452E-07	1,01644183	0,12809474	0,89807401
VCA0534	1,07543678	0,32582276	1,03412814	3,1794E-07	1,03412814	0,12806208	0,89809985
VC2344	1,03036095	-0,8774127	-1,0192405	-8,562E-07	-1,0192405	-0,1278615	0,89825862
VC0780	1,03423623	-0,1723702	-1,0651582	-1,682E-07	-1,0651582	-0,1277321	0,89836101
VCA0188	1,3056707	0,17801518	1,06252673	1,7371E-07	1,06252673	0,12723976	0,89875064
VC0954	1,02336883	-0,8430202	-1,0184668	-8,226E-07	-1,0184668	-0,1271561	0,89881683
VC1554	1,02891792	1,00394793	1,01740608	9,7966E-07	1,01740608	0,12713531	0,89883331
VC1602	-1,0280696	-4,5907765	-1,0386979	-4,48E-06	-1,0386979	-0,1271027	0,89885908
VC0400	1,04454491	-0,6509559	-1,0166597	-6,352E-07	-1,0166597	-0,1270171	0,8989269
VCA1111	1,17752187	0,17770991	1,0615992	1,7341E-07	1,0615992	0,12621254	0,89956369
VC1340	1,00286419	-0,2156381	-1,0503759	-2,104E-07	-1,0503759	-0,1260721	0,89967488
VC0726	1,02153205	-0,6997428	-1,0199388	-6,828E-07	-1,0199388	-0,1259965	0,89973469
VC1332	1,0340954	-0,1691584	-1,0640313	-1,651E-07	-1,0640313	-0,1254717	0,9001501
VCA0876	1,11122192	0,41526844	1,02547508	4,0522E-07	1,02547508	0,12517652	0,90038381
VCA1056	1,01186322	-0,1822682	-1,0585215	-1,779E-07	-1,0585215	-0,12468	0,9007769
VC1989	1,0406486	-0,9749483	-1,0295641	-9,514E-07	-1,0295641	-0,1245036	0,90091654
tRNA-Val-2	-1,0540913	-2,2767754	-1,0541646	-2,222E-06	-1,0541646	-0,1243278	0,90105577
VC1105	1,09428089	0,29770829	1,03511331	2,9051E-07	1,03511331	0,12413651	0,9012072
tpx	1,03475627	4,14299	1,01382196	4,0428E-06	1,01382196	0,12393471	0,90136697
VCA0543	1,06873097	1,39773176	1,02404162	1,3639E-06	1,02404162	0,12390326	0,90139188
zntR	-1,0343852	-1,4617591	-1,0314075	-1,426E-06	-1,0314075	-0,1232964	0,90187238
VC2342	1,16156245	-8,272616	-1,0156158	-8,072E-06	-1,0156158	-0,1227424	0,9023111
VC1261	1,14900996	0,22413776	1,04562818	2,1872E-07	1,04562818	0,12246795	0,90252843
VC0009	-1,0023975	-0,7161772	-1,0382581	-6,989E-07	-1,0382581	-0,1221913	0,90274755
VCA0802	1,04779063	-0,8685744	-1,0179446	-8,476E-07	-1,0179446	-0,1218163	0,90304453
rpmB	1,11324149	-37,480701	-1,0384027	-3,657E-05	-1,0384027	-0,1216783	0,90315381
VCA0023	1,04778102	-0,2415295	-1,0414666	-2,357E-07	-1,0414666	-0,1213179	0,90343926
VC0005	-1,0592577	-4,0605791	-1,0385253	-3,962E-06	-1,0385253	-0,1210524	0,90364958
VC2030	1,05056488	2,70077742	1,02932627	2,6354E-06	1,02932627	0,12076937	0,90387372
VCA0040	1,03930253	-0,5030127	-1,0194843	-4,908E-07	-1,0194843	-0,120664	0,90395717

VC0258	1,01215204	13,753381	1,0373832	1,3421E-05	1,0373832	0,12060749	0,90400194
VC1895	-1,0154302	-1,132255	-1,0105619	-1,105E-06	-1,0105619	-0,1203639	0,90419487
23Sf	1,03275144	-0,2732135	-1,0359064	-2,666E-07	-1,0359064	-0,120232	0,90429938
VCA0256	1,14023672	0,21612332	1,0454859	2,1089E-07	1,0454859	0,12007501	0,90442373
VC1493	1,06307248	-0,466618	-1,020769	-4,553E-07	-1,020769	-0,1199488	0,90452373
VC2678	1,05455414	-0,4141968	-1,0229242	-4,042E-07	-1,0229242	-0,1186657	0,90554025
VCA0778	1,01242886	-0,2633483	-1,0361292	-2,57E-07	-1,0361292	-0,1184006	0,90575028
VCA0440	1,10348531	0,61413576	1,03180462	5,9928E-07	1,03180462	0,11831296	0,90581969
VC2280	1,02231923	-0,4503723	-1,0208335	-4,395E-07	-1,0208335	-0,1180232	0,9060493
VC1467	1,01250073	-0,2846333	-1,0329817	-2,777E-07	-1,0329817	-0,1176996	0,90630571
cca	1,03567718	-0,2896809	-1,0322183	-2,827E-07	-1,0322183	-0,1173785	0,90656016
VCA0956	1,07653038	0,46310924	1,01989225	4,5191E-07	1,01989225	0,11697288	0,90688154
xerC	1,01272324	0,84016276	1,01090399	8,1984E-07	1,01090399	0,11691113	0,90693048
tRNA-Met-1	-1,1141261	-2,5683658	-1,0724586	-2,506E-06	-1,0724586	-0,1168273	0,9069969
rpsI	1,19078876	8,05564296	1,01512474	7,8608E-06	1,01512474	0,11681893	0,90700355
VC1707	1,05131493	-0,940854	-1,0374645	-9,181E-07	-1,0374645	-0,1166896	0,90710604
VC0298	1,08577105	-0,1634089	-1,0568139	-1,595E-07	-1,0568139	-0,1163668	0,90736187
VC0465	1,2088853	0,20250607	1,04555125	1,9761E-07	1,04555125	0,11631228	0,90740506
VC1355	1,03616882	-0,3483347	-1,0260126	-3,399E-07	-1,0260126	-0,1158332	0,90778478
VC2053	-1,0630359	-9,3281716	-1,0444171	-9,103E-06	-1,0444171	-0,1155916	0,90797623
VC0254	-1,0230405	8,30171878	1,03169543	8,1009E-06	1,03169543	0,11553362	0,90802218
VC2718	1,12699069	0,24737892	1,03599857	2,4139E-07	1,03599857	0,11455059	0,90880135
VC1245	1,03053708	-0,3626353	-1,024286	-3,539E-07	-1,024286	-0,114246	0,90904276
VC2445	1,07750011	-0,2476522	-1,0350703	-2,417E-07	-1,0350703	-0,1131522	0,90990988
gyrB	1,0649752	2,57896624	1,02674184	2,5166E-06	1,02674184	0,11311608	0,90993853
VC0736	1,05488223	-0,5378393	-1,0341688	-5,248E-07	-1,0341688	-0,1125141	0,91041581
VC2703	1,11040104	0,30176857	1,02822834	2,9447E-07	1,02822834	0,11224944	0,91062563
16Sa	1,02317603	-0,2545822	-1,0334968	-2,484E-07	-1,0334968	-0,1121646	0,91069286
VCA0269	1,03733463	-0,1666908	-1,0515836	-1,627E-07	-1,0515836	-0,1121317	0,91071899
VC0931	1,13636448	0,16481405	1,05167704	1,6083E-07	1,05167704	0,11159706	0,91114291
VCA0803	1,02436852	-0,1742367	-1,0486774	-1,7E-07	-1,0486774	-0,1114444	0,91126398
VC1815	1,19196957	0,18077023	1,04686797	1,764E-07	1,04686797	0,11143409	0,91127213
VC2307	1,13265822	0,22781483	1,03672481	2,223E-07	1,03672481	0,11101114	0,91160751
VCA0914	1,17350418	0,2366877	1,03492533	2,3096E-07	1,03492533	0,11039412	0,91209682
VCA0240	1,04531011	-0,3705911	-1,0220871	-3,616E-07	-1,0220871	-0,1101998	0,91225091
VCA0068	1,09183417	-0,2270607	-1,0361693	-2,216E-07	-1,0361693	-0,1100008	0,91240871
VC0984	-1,0095936	1,76183766	1,01309256	1,7192E-06	1,01309256	0,10991907	0,91247357
VC1276	1,05333945	-0,450888	-1,0389618	-4,4E-07	-1,0389618	-0,1099003	0,91248846
VC0505	1,03766182	-0,5672368	-1,0270325	-5,535E-07	-1,0270325	-0,1097432	0,91261307
VCA0794	1,02918476	-0,3230935	-1,0305195	-3,153E-07	-1,0305195	-0,1096611	0,91267812
VC0450	1,12418129	0,83986571	1,02827131	8,1955E-07	1,02827131	0,10951245	0,91279605
VC1774	1,13671391	1,74945731	1,06436989	1,7071E-06	1,06436989	0,10948426	0,91281841
VC0762	1,00172256	-2,1222227	-1,0228243	-2,071E-06	-1,0228243	-0,1093291	0,91294148
VC0884	1,03263532	0,83564934	1,02377047	8,1543E-07	1,02377047	0,10859351	0,91352491
VCA0974	1,04791665	1,37229544	1,05714995	1,3391E-06	1,05714995	0,10851604	0,91358636

VC1659	1,18422369	0,21338438	1,03745663	2,0822E-07	1,03745663	0,10848341	0,91361224
VCA0963	1,0429031	-0,3052156	-1,025959	-2,978E-07	-1,025959	-0,1083166	0,91374457
VC2705	1,02011423	-0,0984943	-1,0820496	-9,611E-08	-1,0820496	-0,1079094	0,91406759
VCA1091	1,01412276	-0,1860781	-1,0424964	-1,816E-07	-1,0424964	-0,1077716	0,91417685
VC2217	1,21120521	0,19446472	1,04037466	1,8976E-07	1,04037466	0,10744385	0,91443687
VC0623	1,04420898	-0,3083903	-1,025267	-3,009E-07	-1,025267	-0,1074359	0,91444317
VCA0310	1,03501007	0,66488764	1,01464293	6,488E-07	1,01464293	0,10739699	0,91447404
VC1913	1,01187596	0,90087038	1,01250873	8,7908E-07	1,01250873	0,10729197	0,91455736
VCA0277	1,06458787	10,9708988	1,03437864	1,0705E-05	1,03437864	0,10712743	0,9146879
VC0775	1,14778941	0,15974031	1,04906142	1,5588E-07	1,04906142	0,10711769	0,91469562
5Sd	1,00322127	204,481457	1,04208871	0,00019953	1,04208871	0,10634982	0,91530481
VC1432	1,05611117	1,00205836	1,04573562	9,7782E-07	1,04573562	0,10611868	0,91548819
VCA0824	1,03153067	-0,1493023	-1,0514551	-1,457E-07	-1,0514551	-0,1059932	0,91558778
VCA1038	1,10813067	0,28845725	1,02620439	2,8148E-07	1,02620439	0,1057911	0,9157481
VCA0495	-1,0398914	-3,9438449	-1,0279457	-3,848E-06	-1,0279457	-0,1054008	0,91605777
VCA0882	1,11119353	0,28132789	1,02645029	2,7452E-07	1,02645029	0,10495825	0,91640894
murE	1,05485765	0,73257448	1,01458999	7,1485E-07	1,01458999	0,10495697	0,91640996
VCA0634	1,16759913	0,2024371	1,03664523	1,9754E-07	1,03664523	0,10453408	0,91674553
VC2697	1,0798158	-0,6456876	-1,0310204	-6,301E-07	-1,0310204	-0,1043152	0,91691918
VCA0917	1,0247655	-10,265099	-1,0416801	-1,002E-05	-1,0416801	-0,1042428	0,91697666
VC1187	1,04491623	-0,3618565	-1,0257064	-3,531E-07	-1,0257064	-0,1026669	0,91822732
dnaE	1,0591382	0,5967596	1,01679317	5,8232E-07	1,01679317	0,10253323	0,91833344
VCA0351	1,10890992	2,24708771	1,0374059	2,1927E-06	1,0374059	0,10226339	0,91854762
VC0942	1,05379259	1,71051161	1,02122844	1,6691E-06	1,02122844	0,10192609	0,91881534
VCA0072	1,20784805	0,10736571	1,06659747	1,0477E-07	1,06659747	0,10188135	0,91885086
VCA0780	1,08079411	-0,1869399	-1,0372463	-1,824E-07	-1,0372463	-0,1012587	0,91934511
VC0860	1,13600688	0,1474313	1,0474245	1,4386E-07	1,0474245	0,10121702	0,91937819
VC0300	1,02824353	-0,1115749	-1,0630296	-1,089E-07	-1,0630296	-0,1011262	0,91945026
VC2302	-1,0550728	-7,605795	-1,0429183	-7,422E-06	-1,0429183	-0,1008559	0,91966487
VC1610	1,09779581	0,37864219	1,01785698	3,6948E-07	1,01785698	0,10026248	0,92013594
VC0653	1,07475539	3,120252	1,03589045	3,0448E-06	1,03589045	0,10021094	0,92017687
VC2248	1,02170184	0,63848672	1,01049853	6,2304E-07	1,01049853	0,10001424	0,92033303
VC1472	-1,0145385	-0,360467	-1,0348605	-3,517E-07	-1,0348605	-0,0995968	0,92066443
VC1834	1,1182599	10,8127992	1,04160735	1,0551E-05	1,04160735	0,0995642	0,92069032
cobU	1,09893352	0,39613859	1,03456169	3,8656E-07	1,03456169	0,09920714	0,92097381
VC1539	-1,0246322	-4,1529465	-1,039906	-4,052E-06	-1,039906	-0,0987582	0,92133024
VCA0617	1,10820378	0,28606928	1,02277254	2,7915E-07	1,02277254	0,09829493	0,9216981
VC2474	1,08039255	0,45749603	1,01410824	4,4643E-07	1,01410824	0,09805195	0,92189105
VCA1013	-1,0106775	-1,2210451	-1,0403828	-1,192E-06	-1,0403828	-0,0980262	0,92191149
VC0209	1,02995016	0,90956419	1,01409439	8,8756E-07	1,01409439	0,09795159	0,92197073
VC0383	1,03728742	-0,247501	-1,0261814	-2,415E-07	-1,0261814	-0,0979508	0,92197133
VC2275	1,11486414	1,30883556	1,04278313	1,2772E-06	1,04278313	0,09768622	0,92218147
VCA0979	1,08626196	-0,221985	-1,028787	-2,166E-07	-1,028787	-0,0972085	0,9225608
VC0507	1,00456506	-0,7849666	-1,0184668	-7,66E-07	-1,0184668	-0,0967966	0,92288791
VC2253	1,05301042	1,13832787	1,01610912	1,1108E-06	1,01610912	0,09642358	0,92318417

VCA1055	1,07475329	0,31429443	1,01950921	3,0669E-07	1,01950921	0,09543975	0,92396555
VC1167	1,01448999	-0,3706286	-1,0190066	-3,617E-07	-1,0190066	-0,095182	0,92417028
VC1501	1,02717329	-0,2872148	-1,0210685	-2,803E-07	-1,0210685	-0,0947749	0,92449363
VC1540	1,0962688	0,29176482	1,03065314	2,8471E-07	1,03065314	0,09459395	0,92463735
anmK	1,06958267	-0,6858465	-1,0250017	-6,693E-07	-1,0250017	-0,0945094	0,92470453
VCA0801	1,03602205	1,40101901	1,05143943	1,3671E-06	1,05143943	0,09440635	0,92478637
VC0402	1,04588904	0,80168014	1,01157684	7,8229E-07	1,01157684	0,09417734	0,92496829
VCA0223	1,13530955	0,10770455	1,05628111	1,051E-07	1,05628111	0,0940412	0,92507643
VC1058	1,04826099	0,56416087	1,02767933	5,5051E-07	1,02767933	0,09381496	0,92525615
VC1458	1,06314153	-0,1435232	-1,0414294	-1,401E-07	-1,0414294	-0,093478	0,92552384
VC1307	1,07140068	0,92996789	1,03011052	9,0747E-07	1,03011052	0,09341683	0,92557242
VCA0410	1,05582547	-0,9281153	-1,0294483	-9,057E-07	-1,0294483	-0,0932084	0,92573798
VC0425	1,04919112	-0,3587419	-1,0256412	-3,501E-07	-1,0256412	-0,0930666	0,92585066
VC0773	1,33178706	0,05673155	1,10705655	5,5359E-08	1,10705655	0,09299115	0,9259106
VCA1110	1,31023794	0,04766905	1,12661058	4,6516E-08	1,12661058	0,092272	0,92648193
VC1773	1,10384816	1,56112162	1,04864286	1,5234E-06	1,04864286	0,0922292	0,92651594
VC0932	1,04188997	-0,1234966	-1,0469736	-1,205E-07	-1,0469736	-0,092206	0,92653435
VC2506	1,0984613	0,24026158	1,03832087	2,3445E-07	1,03832087	0,09203381	0,92667118
VCA0306	1,06215996	-0,2429679	-1,023222	-2,371E-07	-1,023222	-0,0914672	0,9271214
VC1932	1,08488663	0,21920021	1,02572323	2,139E-07	1,02572323	0,09138094	0,92718991
VC1630	-1,0487968	-2,1746281	-1,0335625	-2,122E-06	-1,0335625	-0,0913799	0,9271907
VC1655	1,084318	-0,2647582	-1,0292653	-2,584E-07	-1,0292653	-0,0910568	0,92744744
nadE	1,04512193	-0,4447301	-1,0124813	-4,34E-07	-1,0124813	-0,0909663	0,92751938
VC0509	1,06812281	-0,2004437	-1,0346465	-1,956E-07	-1,0346465	-0,0908702	0,92759572
VC0098	1,08771791	-0,1902203	-1,0290764	-1,856E-07	-1,0290764	-0,0904299	0,92794563
VC0674	-1,0037638	-1,3920584	-1,010523	-1,358E-06	-1,010523	-0,0901963	0,92813123
VCA0361	1,03310226	-0,32633	-1,0227565	-3,184E-07	-1,0227565	-0,0898788	0,9283835
VCA0147	1,1356051	0,14287211	1,03821915	1,3942E-07	1,03821915	0,08964999	0,92856536
VC1814	1,08616012	-0,1034826	-1,0528948	-1,01E-07	-1,0528948	-0,0894371	0,92873453
tbpA	1,04359831	-0,1648198	-1,0328054	-1,608E-07	-1,0328054	-0,0893287	0,92882071
VCA0387	1,09440149	0,36404295	1,01811764	3,5524E-07	1,01811764	0,08867846	0,92933746
VCA1012	-1,0486923	-2,1388426	-1,0342985	-2,087E-06	-1,0342985	-0,088366	0,92958577
VCA0722	1,07441014	-0,1756436	-1,0299627	-1,714E-07	-1,0299627	-0,0881911	0,92972481
VCA0625	1,0618767	-0,0908402	-1,0585725	-8,864E-08	-1,0585725	-0,088057	0,92983141
VC1683	1,10338788	0,54931054	1,02061704	5,3602E-07	1,02061704	0,08769318	0,93012054
VCA0855	1,0843474	-0,0531499	-1,100676	-5,186E-08	-1,100676	-0,0874169	0,93034012
VCA0216	1,12562343	0,24477624	1,02223615	2,3885E-07	1,02223615	0,08741582	0,930341
VC1131	1,07079068	0,66477771	1,02901105	6,487E-07	1,02901105	0,08728425	0,93044558
VCA0304	1,00647493	-0,2096736	-1,0325401	-2,046E-07	-1,0325401	-0,0868903	0,93075872
VC1629	-1,0171603	-0,7031199	-1,0289745	-6,861E-07	-1,0289745	-0,0864875	0,9310789
greA	1,12103713	-7,3537537	-1,018346	-7,176E-06	-1,018346	-0,0862423	0,93127382
VC1154	-1,0075561	-4,2266684	-1,0233429	-4,124E-06	-1,0233429	-0,086115	0,931375
VC2008	1,07118202	-0,7892337	-1,0283161	-7,701E-07	-1,0283161	-0,0857104	0,93169662
VC0159	1,02306874	-7,3273784	-1,0266411	-7,15E-06	-1,0266411	-0,0856857	0,9317163
VCA0800	1,0257253	-0,5452505	-1,0119696	-5,321E-07	-1,0119696	-0,0854473	0,93190577

VCA0414	1,04013334	-0,5780664	-1,0275046	-5,641E-07	-1,0275046	-0,0853739	0,93196413
VC0661	1,07629459	-0,1925516	-1,0255314	-1,879E-07	-1,0255314	-0,0853303	0,93199879
5Sb	-1,1594224	992,224048	1,02311193	0,00096822	1,02311193	0,08484466	0,93238489
VC2443	1,07950897	0,52134864	1,01355234	5,0874E-07	1,01355234	0,08429738	0,93282
VC1151	1,02949877	-0,5893999	-1,022377	-5,751E-07	-1,022377	-0,083771	0,93323851
VCA1032	1,0657537	0,33826026	1,01743941	3,3008E-07	1,01743941	0,08338062	0,9335489
VCA0032	1,09856879	0,18062972	1,0255681	1,7626E-07	1,0255681	0,08270515	0,93408599
VC1589	1,02964933	0,15849711	1,03965682	1,5466E-07	1,03965682	0,08225162	0,93444663
VCA0782	1,08289761	-0,1892505	-1,0240196	-1,847E-07	-1,0240196	-0,0820835	0,9345803
VCA1082	1,02687784	0,72488481	1,01217018	7,0735E-07	1,01217018	0,08195896	0,93467935
VC2007	1,01887899	-3,1311803	-1,0435065	-3,055E-06	-1,0435065	-0,0819137	0,93471536
VC1291	1,11582036	0,35787202	1,01255542	3,4921E-07	1,01255542	0,08184125	0,93477296
fliH	1,02461272	0,57938121	1,00769214	5,6537E-07	1,00769214	0,08160823	0,93495826
VCA0864	1,09294628	0,26253319	1,01697209	2,5618E-07	1,01697209	0,08140925	0,93511649
VC1349	1,08460404	0,21227498	1,02100449	2,0714E-07	1,02100449	0,08135507	0,93515958
VC0252	1,0836063	-10,272274	-1,0163698	-1,002E-05	-1,0163698	-0,0810775	0,93538034
djlA	1,07685147	0,40463143	1,01512699	3,9484E-07	1,01512699	0,08091724	0,93550778
VC0015a	1,07430904	-0,1494256	-1,0295768	-1,458E-07	-1,0295768	-0,0808252	0,93558093
VC0195	1,06907248	-0,1543118	-1,0285007	-1,506E-07	-1,0285007	-0,0806495	0,93572069
VC2529	-1,0119505	-3,1739442	-1,0203384	-3,097E-06	-1,0203384	-0,0805905	0,93576763
VC1520	-1,0149927	-0,9312361	-1,0274709	-9,087E-07	-1,0274709	-0,0793197	0,93677838
pssA	1,07802459	0,36737474	1,0112319	3,5849E-07	1,0112319	0,07845449	0,93746653
miaA	-1,0026296	0,81613612	1,00575685	7,9639E-07	1,00575685	0,07833389	0,93756246
VC0236	1,04371855	0,63690985	1,0160141	6,215E-07	1,0160141	0,07787593	0,93792675
VC0737	1,00722659	-1,8960174	-1,0368757	-1,85E-06	-1,0368757	-0,0775098	0,93821801
VCA0851	1,09404371	0,22629661	1,01783325	2,2082E-07	1,01783325	0,07745955	0,93825797
VCA0605	1,26103915	0,04842225	1,08611943	4,7251E-08	1,08611943	0,0774398	0,93827368
VCA0710	1,0726196	-0,3262262	-1,0162111	-3,183E-07	-1,0162111	-0,0772722	0,93840698
VC2135	1,06048199	0,41202453	1,0096745	4,0206E-07	1,0096745	0,07714052	0,93851176
VCA0224	1,00736187	-0,0496558	-1,0829836	-4,845E-08	-1,0829836	-0,0770369	0,93859419
VCA0642	1,40116875	0,0474289	1,08608335	4,6282E-08	1,08608335	0,07662598	0,93892109
VC0341	1,02611394	-0,519233	-1,0107007	-5,067E-07	-1,0107007	-0,0757077	0,93965163
VC1684	1,09154035	0,4790134	1,01723285	4,6743E-07	1,01723285	0,07569901	0,93965857
VC1699	-1,0061424	-2,5359759	-1,0191944	-2,475E-06	-1,0191944	-0,0750509	0,94017422
VC1817	1,25336969	0,07944014	1,04819596	7,7518E-08	1,04819596	0,07488598	0,94030543
lnt	1,02080126	-0,606287	-1,0131106	-5,916E-07	-1,0131106	-0,0743136	0,94076085
VC2740	1,07589172	0,38089402	1,0095408	3,7168E-07	1,0095408	0,07365719	0,94128317
VC0888	1,08684883	0,23030362	1,01581345	2,2473E-07	1,01581345	0,0736211	0,94131188
VCA1073	1,12093791	-0,422986	-1,050736	-4,128E-07	-1,050736	-0,0735671	0,94135489
VCA0546	1,06410297	0,43824933	1,01936787	4,2765E-07	1,01936787	0,07347926	0,94142475
VC2764	1,15520895	5,71230022	1,00835471	5,5741E-06	1,00835471	0,07345174	0,94144665
VCA0151	1,08734132	-0,0926106	-1,0395722	-9,037E-08	-1,0395722	-0,0734204	0,94147156
fabZ	1,01333301	-0,9496502	-1,0085762	-9,267E-07	-1,0085762	-0,0732234	0,94162832
VCA0117	1,07095176	-0,1200558	-1,0299269	-1,172E-07	-1,0299269	-0,0728692	0,9419102
VC0283	1,23181985	0,05900806	1,06147477	5,7581E-08	1,06147477	0,07265675	0,94207927

VC2073	1,02069172	-0,1779978	-1,0201722	-1,737E-07	-1,0201722	-0,072444	0,94224857
VC0311	1,07943438	0,26915372	1,01379657	2,6264E-07	1,01379657	0,07186333	0,94271068
VC1376	1,05748958	-1,6473748	-1,0269661	-1,608E-06	-1,0269661	-0,0717503	0,94280066
VC2750	1,13867894	0,43094558	1,01959829	4,2052E-07	1,01959829	0,07134262	0,94312507
tRNA-Cys-2	1,07616487	0,5759518	1,04315462	5,6202E-07	1,04315462	0,07129	0,94316695
VC1809	1,05851455	-0,2316419	-1,0145405	-2,26E-07	-1,0145405	-0,070823	0,94353862
VC1321	1,03829381	-0,445009	-1,0094042	-4,342E-07	-1,0094042	-0,0704585	0,94382873
VCA0127	1,09774666	-0,9908468	-1,0512246	-9,669E-07	-1,0512246	-0,0700065	0,94418851
VCA0181	1,04192335	-0,2698487	-1,0119658	-2,633E-07	-1,0119658	-0,0693884	0,94468047
VC2330	1,06449742	-0,1002241	-1,0319583	-9,78E-08	-1,0319583	-0,0687672	0,9451749
VCA0781	1,09502923	-0,13127	-1,0242815	-1,281E-07	-1,0242815	-0,0687301	0,94520443
VC1273	1,07435338	-0,0802606	-1,0397036	-7,832E-08	-1,0397036	-0,0684611	0,9454186
VC2004	1,13360952	0,13303868	1,02324899	1,2982E-07	1,02324899	0,06772175	0,94600714
VC2619	1,00550774	-0,4119364	-1,0260271	-4,02E-07	-1,0260271	-0,0674009	0,94626259
VC0340	1,04195035	-0,3917483	-1,0077542	-3,823E-07	-1,0077542	-0,0673733	0,94628451
VC1104	1,05913478	0,22550369	1,01350879	2,2005E-07	1,01350879	0,06737092	0,94628642
VC1902	1,07857141	0,26086957	1,01155122	2,5456E-07	1,01155122	0,0670387	0,9465509
VCA1101	1,16839806	0,07385687	1,04113142	7,207E-08	1,04113142	0,06682021	0,94672483
VC1878	1,03241297	0,54297319	1,00958575	5,2984E-07	1,00958575	0,06673276	0,94679445
VC1523	1,05441735	1,94432232	1,02739005	1,8973E-06	1,02739005	0,06647547	0,94699929
VC1052	1,03303278	-0,7466271	-1,014532	-7,286E-07	-1,014532	-0,0661616	0,94724919
VCA0122	1,06312754	0,17861852	1,01642754	1,743E-07	1,01642754	0,06607257	0,94732005
VC1050	1,06081369	-0,2794243	-1,0144733	-2,727E-07	-1,0144733	-0,0660511	0,94733712
VCA1069	1,0325778	-5,4871855	-1,0392565	-5,354E-06	-1,0392565	-0,0660478	0,94733974
VC1322	1,09278341	-0,2472543	-1,0117353	-2,413E-07	-1,0117353	-0,0656926	0,94762253
VCA0790	1,02505354	-0,3949096	-1,0202877	-3,854E-07	-1,0202877	-0,0655204	0,94775967
VC0935	1,13103647	0,10616523	1,02732413	1,036E-07	1,02732413	0,06551854	0,94776115
VC0489	1,06597183	-0,0971119	-1,0298628	-9,476E-08	-1,0298628	-0,0654681	0,94780132
VCA0152	1,13032329	0,13154798	1,02188296	1,2837E-07	1,02188296	0,06535503	0,94789133
VCA0583	1,07037478	-0,1041529	-1,027481	-1,016E-07	-1,027481	-0,0650782	0,94811175
VCA0220	1,10647272	2,65863098	1,02969609	2,5943E-06	1,02969609	0,06484721	0,94829566
VC0497	1,07627495	0,13943778	1,02010037	1,3606E-07	1,02010037	0,06451607	0,94855932
VC0804	1,07181453	-0,1146197	-1,0280652	-1,118E-07	-1,0280652	-0,0644708	0,94859537
VC2220	1,08536554	-0,2116182	-1,0152137	-2,065E-07	-1,0152137	-0,0643762	0,94867065
VCA0596	1,05116134	-0,1440299	-1,0192474	-1,405E-07	-1,0192474	-0,064177	0,94882927
VC2633	1,12364612	0,07221432	1,03854871	7,0467E-08	1,03854871	0,06400547	0,94896588
VCA0983	1,123243	-0,1543172	-1,0317244	-1,506E-07	-1,0317244	-0,0638914	0,94905672
VC2623	1,01604855	0,68750995	1,00804178	6,7088E-07	1,00804178	0,06377262	0,94915128
VC0116	1,08516413	0,3377053	1,01009702	3,2954E-07	1,01009702	0,06372856	0,94918637
VCA0973	1,077471	0,12712213	1,04295571	1,2405E-07	1,04295571	0,06346863	0,94939334
VC0282	1,14741078	0,38400392	1,0266634	3,7471E-07	1,0266634	0,06318583	0,94961853
VC2285	1,05798617	0,31700289	1,00828946	3,0933E-07	1,00828946	0,06265424	0,95004184
VC0844	1,03982879	-0,1734717	-1,0183837	-1,693E-07	-1,0183837	-0,0625107	0,95015616
VCA0148	1,17933858	0,09285384	1,0282667	9,0608E-08	1,0282667	0,06230692	0,95031842
VC1940	1,0662919	-0,1665613	-1,0156443	-1,625E-07	-1,0156443	-0,062276	0,95034306

VC1549	1,16577464	0,05940139	1,04440402	5,7964E-08	1,04440402	0,06221384	0,95039254
VC0684	1,06790978	0,32342258	1,00899814	3,156E-07	1,00899814	0,06164831	0,9508429
VC0826	-1,1036507	-37,385952	-1,0224364	-3,648E-05	-1,0224364	-0,0607427	0,95156414
VC1924	1,06747342	-0,1355621	-1,0181632	-1,323E-07	-1,0181632	-0,0604992	0,95175808
VCA0928	1,08401674	-0,1105817	-1,0222435	-1,079E-07	-1,0222435	-0,0604071	0,95183144
VCA0374	1,12420714	0,10664393	1,0230192	1,0406E-07	1,0230192	0,06033576	0,95188822
aspA	1,06819159	35,4308609	1,0336701	3,4574E-05	1,0336701	0,06029279	0,95192245
VC1879	1,07475166	0,28577015	1,01513506	2,7886E-07	1,01513506	0,05989684	0,9522378
VC0154	1,05251271	-0,1719666	-1,0139811	-1,678E-07	-1,0139811	-0,0598451	0,95227904
VCA1049	1,08960172	-0,0956428	-1,0252734	-9,333E-08	-1,0252734	-0,0598381	0,95228461
VC1382	1,02588306	-0,2275595	-1,0111002	-2,221E-07	-1,0111002	-0,0595425	0,95252006
VC1041	1,09103564	0,19036681	1,01230342	1,8576E-07	1,01230342	0,05909159	0,95287916
VCA0957	1,08051025	-0,1280676	-1,0181055	-1,25E-07	-1,0181055	-0,0587104	0,95318281
lpxK	1,04862908	0,59728811	1,01274837	5,8284E-07	1,01274837	0,05859157	0,95327742
VC2430	1,09151377	0,43387684	1,01401322	4,2338E-07	1,01401322	0,05844755	0,95339214
VCA0142	1,1009872	0,29938047	1,01654582	2,9214E-07	1,01654582	0,05842879	0,95340709
VC2544	1,07216434	3,06552553	1,03141726	2,9914E-06	1,03141726	0,05830008	0,95350961
VC2605	-1,0348715	-2,2900005	-1,0215078	-2,235E-06	-1,0215078	-0,0581679	0,9536149
VCA0287	1,20398227	6,38250494	1,01385506	6,2281E-06	1,01385506	0,05805929	0,95370141
VC1749	1,09906476	-0,0985152	-1,0228241	-9,613E-08	-1,0228241	-0,0577472	0,95395003
VCA0036	1,07478195	0,87938574	1,02161341	8,5811E-07	1,02161341	0,05768353	0,95400071
VC0401	1,06877345	0,32476835	1,00887262	3,1691E-07	1,00887262	0,05756176	0,95409771
VC2537	1,07353348	0,12302559	1,01810539	1,2005E-07	1,01810539	0,05754292	0,95411272
VC1270	1,07149616	0,54327425	1,01057937	5,3013E-07	1,01057937	0,05740808	0,95422013
VCA0409	1,10179925	0,60045903	1,01526299	5,8593E-07	1,01526299	0,05728585	0,95431749
VC0703	1,05412438	-0,1730368	-1,0125583	-1,689E-07	-1,0125583	-0,0569147	0,95461314
VC0149	1,02269972	-0,3975095	-1,0065962	-3,879E-07	-1,0065962	-0,056904	0,95462165
tRNA-Ser-3	1,01001563	0,67883864	1,01499105	6,6242E-07	1,01499105	0,05667225	0,95480628
VC1145	1,06385255	0,25978071	1,00944757	2,535E-07	1,00944757	0,05644255	0,95498927
VC1941	1,02967355	-0,2631618	-1,008458	-2,568E-07	-1,008458	-0,0563194	0,95508734
VC1287	1,04215424	-0,5506903	-1,0144846	-5,374E-07	-1,0144846	-0,0563051	0,95509873
phoR	1,0947671	0,12146559	1,01726355	1,1853E-07	1,01726355	0,05584348	0,9554665
VC0354	1,11943505	13,2937373	1,01639791	1,2972E-05	1,01639791	0,05565889	0,95561356
VC2561	-1,0756216	-0,0805179	-1,044778	-7,857E-08	-1,044778	-0,0555887	0,95566945
tRNA-Cys-3	1,01527202	-0,0418186	-1,0501135	-4,081E-08	-1,0501135	-0,0553776	0,95583764
maf (NC_002505 2179182..2179804)	1,03784176	0,58027668	1,01292527	5,6624E-07	1,01292527	0,05528437	0,95591192
VC1704	1,16143511	0,0839856	1,02443	8,1954E-08	1,02443	0,05514093	0,95602619
VC1334	1,22133794	0,05469292	1,03767305	5,337E-08	1,03767305	0,05507756	0,95607668
VC2484	-1,017089	2,60946757	1,01894212	2,5463E-06	1,01894212	0,05492255	0,95620017
VC0176	1,07273408	0,18119729	1,01257422	1,7681E-07	1,01257422	0,0542707	0,9567195
VCA0120	1,0346268	-0,1091433	-1,0179073	-1,065E-07	-1,0179073	-0,0539045	0,95701123
VCA0221	1,09229796	-0,0495044	-1,0398055	-4,831E-08	-1,0398055	-0,0538344	0,95706708
VC2054	-1,0500796	-2,3662559	-1,0214667	-2,309E-06	-1,0214667	-0,0537452	0,95713816
VC1653	1,05520739	0,15956468	1,01442523	1,557E-07	1,01442523	0,05344488	0,95737745
tRNA-Tyr-3	1,13737107	0,23782179	1,03630723	2,3207E-07	1,03630723	0,05343657	0,95738407

VCA0413	1,12860748	0,52631846	1,01756177	5,1359E-07	1,01756177	0,05338786	0,95742288
VCA0556	1,10076391	-0,0717447	-1,0267176	-7,001E-08	-1,0267176	-0,053267	0,95751919
VC2056	1,05905137	-0,2256893	-1,0083947	-2,202E-07	-1,0083947	-0,0531987	0,95757362
VC2505	1,00069583	-0,4393753	-1,0145671	-4,287E-07	-1,0145671	-0,0530761	0,9576713
VC1468	1,10219257	0,10174911	1,0183399	9,9288E-08	1,0183399	0,05266578	0,95799821
VC0192	1,07837246	-0,1185186	-1,015546	-1,157E-07	-1,015546	-0,0523682	0,95823529
fxsA	1,08368278	0,72077528	1,01302385	7,0334E-07	1,01302385	0,05217042	0,95839291
VC0208	1,04714202	-0,2509369	-1,007218	-2,449E-07	-1,007218	-0,052031	0,95850397
VC2042	1,05234205	-0,141219	-1,0128403	-1,378E-07	-1,0128403	-0,0519866	0,95853934
VC2530	1,02258775	9,12878488	1,02122711	8,9079E-06	1,02122711	0,05125899	0,95911915
tRNA-Pro-4	1,06377967	0,30228897	1,03088204	2,9498E-07	1,03088204	0,05061601	0,95963151
VC1327	1,03404998	-5,0679053	-1,0263606	-4,945E-06	-1,0263606	-0,0504667	0,95975046
VC1988	1,08885526	0,20762833	1,00934848	2,0261E-07	1,00934848	0,05023629	0,95993409
VCA0872	1,09648139	0,12003522	1,01401688	1,1713E-07	1,01401688	0,05006232	0,96007273
VC1505	1,09751333	0,22576229	1,0145636	2,203E-07	1,0145636	0,04954647	0,96048381
VC0806	1,07527928	0,14966787	1,01710861	1,4605E-07	1,01710861	0,04939422	0,96060514
VC1275	1,15436606	0,0881021	1,0185891	8,5971E-08	1,0185891	0,04933556	0,96065188
prfB	1,01510657	1,18672809	1,01314361	1,158E-06	1,01314361	0,04900393	0,96091617
5Sa	1,00370239	91,0096958	1,01848942	8,8808E-05	1,01848942	0,04873611	0,9611296
5Se	-1,3078267	-135,73507	-1,0157493	-0,0001325	-1,0157493	-0,0486458	0,96120158
VC2408	1,1051152	0,13674088	1,01159963	1,3343E-07	1,01159963	0,04863664	0,96120887
VCA0483	1,09616228	0,6805988	1,01629835	6,6413E-07	1,01629835	0,04850279	0,96131554
aat	1,11304525	0,06493389	1,024433	6,3363E-08	1,024433	0,04848792	0,9613274
VCA0307	1,07445642	0,38413428	1,01082756	3,7484E-07	1,01082756	0,04835522	0,96143315
VCA0531	1,07534034	0,11741279	1,01231701	1,1457E-07	1,01231701	0,04643279	0,9629653
VC0424	-1,0080941	-1,3425875	-1,016528	-1,31E-06	-1,016528	-0,0462022	0,96314909
VCA0635	1,07329883	-0,0851693	-1,0166785	-8,311E-08	-1,0166785	-0,0459688	0,9633351
VC0264	1,0654796	0,21866214	1,008565	2,1337E-07	1,008565	0,04595822	0,96334355
gmk	-1,0124041	-0,4863556	-1,0172191	-4,746E-07	-1,0172191	-0,0447735	0,96428784
VC0370	1,03368225	-0,2694591	-1,0151489	-2,629E-07	-1,0151489	-0,0447697	0,96429085
VC2732	1,0894656	0,18296796	1,00907446	1,7854E-07	1,00907446	0,04474896	0,9643074
VCA0389	1,06315206	0,20207902	1,01096225	1,9719E-07	1,01096225	0,04463147	0,96440106
tRNA-Arg-1	-1,0426181	-1,0985505	-1,017597	-1,072E-06	-1,017597	-0,0440748	0,96484475
VCA0599	1,06063258	-0,1256391	-1,0102982	-1,226E-07	-1,0102982	-0,0439416	0,96495092
VC0745	-1,0377956	-1,6226039	-1,0206698	-1,583E-06	-1,0206698	-0,0438086	0,96505695
VC1635	1,01628828	0,65681854	1,01008661	6,4093E-07	1,01008661	0,04324181	0,96550877
VCA0808	-1,009488	1,06977031	1,01215119	1,0439E-06	1,01215119	0,04287059	0,96580469
16Sg	1,03865195	-0,0959129	-1,012533	-9,359E-08	-1,012533	-0,0423308	0,96623503
VC2146	1,02061305	-0,4268974	-1,0095417	-4,166E-07	-1,0095417	-0,0421576	0,96637307
tRNA-Asp-3	-1,0058803	-0,390787	-1,0199671	-3,813E-07	-1,0199671	-0,0408937	0,96738064
VCA0783	1,10507291	0,14179597	1,00774406	1,3837E-07	1,00774406	0,04050669	0,96768917
VC0602	1,06209277	0,14024754	1,0076623	1,3685E-07	1,0076623	0,0400725	0,96803532
VC0969	1,02370326	-0,2460306	-1,0053114	-2,401E-07	-1,0053114	-0,0399116	0,9681636
VCA1108	1,0321321	-0,0488457	-1,0208183	-4,766E-08	-1,0208183	-0,0388538	0,96900695
VC1503	-1,0248015	-0,8517078	-1,0100936	-8,311E-07	-1,0100936	-0,0387541	0,9690864

VCA1040	1,07363663	0,15028669	1,00658304	1,4665E-07	1,00658304	0,03846009	0,96932085
VC0462	1,09239065	0,127292	1,00776529	1,2421E-07	1,00776529	0,03843147	0,96934367
VCA0121	1,09292341	0,07760049	1,01260573	7,5723E-08	1,01260573	0,03818553	0,96953976
VC2055	-1,0058089	1,29357418	1,01490453	1,2623E-06	1,01490453	0,03743213	0,97014046
VC0519	1,00639958	-12,261116	-1,0093206	-1,196E-05	-1,0093206	-0,0373508	0,97020534
sdhB	1,04027723	1,76932101	1,02049146	1,7265E-06	1,02049146	0,03558919	0,97160993
VC1372	1,04971462	-0,1204645	-1,0069585	-1,176E-07	-1,0069585	-0,0353984	0,97176209
VCA0735	1,04183664	0,25379346	1,00406012	2,4765E-07	1,00406012	0,03508665	0,97201065
slmA	1,07212239	-0,2529516	-1,0071398	-2,468E-07	-1,0071398	-0,0348925	0,97216547
VC0974	1,09370456	0,26840704	1,00758872	2,6191E-07	1,00758872	0,03487642	0,97217829
VC1446	1,07043163	-0,0887319	-1,0088784	-8,659E-08	-1,0088784	-0,0343	0,97263795
VC2471	1,01308036	1,35704339	1,00733132	1,3242E-06	1,00733132	0,03397567	0,97289656
VC1274	1,11545832	-0,0593571	-1,0129624	-5,792E-08	-1,0129624	-0,0338628	0,97298656
VCA0669	1,08314604	-0,040036	-1,0191448	-3,907E-08	-1,0191448	-0,0337465	0,97307933
tRNA-Tyr-5	1,03980194	-0,1559443	-1,0226811	-1,522E-07	-1,0226811	-0,0335035	0,97327307
VCA0328	1,03874613	-0,0881048	-1,008439	-8,597E-08	-1,008439	-0,0333257	0,9734149
VCA1089	1,01518684	-0,0451259	-1,0164711	-4,403E-08	-1,0164711	-0,0332536	0,97347232
VC1952	1,12186825	-0,0578654	-1,0127823	-5,647E-08	-1,0127823	-0,033203	0,97351268
VC0042	1,0632788	-0,1586999	-1,0046125	-1,549E-07	-1,0046125	-0,0330985	0,97359601
VC2379	1,04856393	0,17337162	1,00410465	1,6918E-07	1,00410465	0,03263897	0,97396249
VC0993	1,04712078	-1,1993925	-1,0114344	-1,17E-06	-1,0114344	-0,0325569	0,97402791
VC1009	1,02781306	-0,2463628	-1,0049493	-2,404E-07	-1,0049493	-0,0321803	0,97432824
VCA0547	1,09759812	-4,725167	-1,0133858	-4,611E-06	-1,0133858	-0,0321607	0,97434393
def (NC_002506 167966..168513)	1,03159468	0,20486183	1,01278152	1,9991E-07	1,01278152	0,03208194	0,97440671
VC2488	1,13170755	-0,0619188	-1,0110436	-6,042E-08	-1,0110436	-0,0319388	0,97452088
VC0611	1,07854124	0,07067372	1,00955708	6,8964E-08	1,00955708	0,03175439	0,97466793
VC0495	1,11520912	-0,0624121	-1,0106925	-6,09E-08	-1,0106925	-0,0315547	0,97482717
VCA0292	1,06307062	0,67317223	1,01159781	6,5689E-07	1,01159781	0,0314657	0,97489815
VC1609	1,0623064	0,11286597	1,00691099	1,1014E-07	1,00691099	0,03136922	0,97497509
VCA0428	-1,0159993	-0,5139312	-1,022673	-5,015E-07	-1,022673	-0,03114	0,97515788
VCA0875	1,00167041	-0,8939185	-1,0058515	-8,723E-07	-1,0058515	-0,0304798	0,97568444
VC1304	1,05909045	2,55745174	1,01374305	2,4956E-06	1,01374305	0,03019979	0,97590772
VC1396	1,04535209	0,11414468	1,00728071	1,1138E-07	1,00728071	0,0300449	0,97603124
VC2775	1,01228749	-0,3483524	-1,0050142	-3,399E-07	-1,0050142	-0,0290227	0,97684653
secD (NC_002505 795818..797712)	1,06763312	0,37965689	1,00557202	3,7047E-07	1,00557202	0,02891883	0,97692933
VC1339	1,14995519	0,04145005	1,01548716	4,0447E-08	1,01548716	0,02865621	0,97713877
VC2573	1,1249211	2,33532121	1,00216978	2,2788E-06	1,00216978	0,02859567	0,97718706
VCA0891	1,08580864	0,18997045	1,01331727	1,8537E-07	1,01331727	0,0285383	0,97723282
engA	-1,0113075	-0,935935	-1,0087575	-9,133E-07	-1,0087575	-0,0284128	0,97733292
VC2216	-1,0185294	0,33313472	1,0045652	3,2508E-07	1,0045652	0,02841122	0,97733419
VC0499	1,07162136	0,06780594	1,0078312	6,6166E-08	1,0078312	0,02816741	0,97752863
VC2773	1,03649438	0,39689016	1,00417429	3,8729E-07	1,00417429	0,02804902	0,97762305
VC1703	1,07107192	0,88007183	1,00866744	8,5878E-07	1,00866744	0,02773213	0,97787579
tRNA-Tyr-2	-1,0690288	-0,6139224	-1,0201142	-5,991E-07	-1,0201142	-0,0277108	0,97789282
VC0382	1,077094	-0,0840985	-1,0074935	-8,206E-08	-1,0074935	-0,0274292	0,9781174

VCA0686	1,0656401	0,36446009	1,03146764	3,5564E-07	1,03146764	0,0274202	0,97812458
VC1065	1,02646548	0,17374747	1,00288317	1,6954E-07	1,00288317	0,02739301	0,97814628
VC0434	1,03787152	-0,2191992	-1,0044812	-2,139E-07	-1,0044812	-0,0272513	0,97825934
VC1619	1,04426305	-0,0942327	-1,0053237	-9,195E-08	-1,0053237	-0,0270081	0,97845328
VC1160	1,04937827	-0,1425387	-1,0054053	-1,391E-07	-1,0054053	-0,0264463	0,97890137
VCA0682	1,09478868	0,16665175	1,01249172	1,6262E-07	1,01249172	0,02604354	0,97922261
VCA0552	1,09549901	0,06232065	1,00722077	6,0813E-08	1,00722077	0,02593418	0,97930984
VC1207	1,10702934	0,16976508	1,00713805	1,6566E-07	1,00713805	0,02584358	0,97938211
VC0017	1,03738672	0,06839512	1,00623896	6,6741E-08	1,00623896	0,02526039	0,97984727
VC1667	1,04670768	-0,073387	-1,0056883	-7,161E-08	-1,0056883	-0,0249882	0,98006441
VC0059	1,13251967	-4,8212275	-1,0109799	-4,705E-06	-1,0109799	-0,0241194	0,98075739
VC1323	1,10027107	-0,1124776	-1,0052014	-1,098E-07	-1,0052014	-0,0239967	0,98085527
VC0708	1,00036747	-0,3031706	-1,0019252	-2,958E-07	-1,0019252	-0,0237489	0,98105292
psd	1,05231805	-0,1154421	-1,003671	-1,126E-07	-1,003671	-0,0232746	0,98143123
VCA0091	1,07235675	-0,0666836	-1,0053648	-6,507E-08	-1,0053648	-0,023134	0,98154338
VCA0118	1,01383887	-0,029177	-1,011921	-2,847E-08	-1,011921	-0,0227736	0,98183084
VCA0969	1,113191	0,10958511	1,00521996	1,0693E-07	1,00521996	0,02261173	0,98195999
cobT	1,07442552	-0,1243305	-1,0070939	-1,213E-07	-1,0070939	-0,0225298	0,98202535
VC1049	-1,0018894	-0,8526893	-1,0080229	-8,321E-07	-1,0080229	-0,0224376	0,98209886
apaH	1,05170228	-0,1192041	-1,0036522	-1,163E-07	-1,0036522	-0,0223163	0,98219567
VC2547	1,05070296	0,09148154	1,00360681	8,9268E-08	1,00360681	0,02222736	0,9822666
VC2281	1,05759478	-0,0787336	-1,0040455	-7,683E-08	-1,0040455	-0,0218363	0,98257855
VC0306	1,12661482	-3,8692292	-1,005329	-3,776E-06	-1,005329	-0,0218253	0,98258735
VCA0232	1,07729531	-0,0233141	-1,0133591	-2,275E-08	-1,0133591	-0,0215426	0,98281284
VC1142	1,05135589	-0,3090225	-1,0044988	-3,015E-07	-1,0044988	-0,0215363	0,98281786
VC2359	1,06599675	-0,0817492	-1,003681	-7,977E-08	-1,003681	-0,0212265	0,98306501
VC1634	1,0429903	-0,114825	-1,0050145	-1,12E-07	-1,0050145	-0,0210794	0,9831823
VC1500	1,04836789	-0,0526145	-1,0055954	-5,134E-08	-1,0055954	-0,020985	0,98325765
VC2423	1,06910389	0,1467509	1,00623023	1,432E-07	1,00623023	0,02048962	0,9836528
nrdG	1,08085073	0,7167242	1,01314909	6,9939E-07	1,01314909	0,0203854	0,98373594
VCA0281	1,06353005	-0,5539978	-1,0081761	-5,406E-07	-1,0081761	-0,0201528	0,98392152
VC0629	1,05167578	0,05663924	1,00467786	5,5269E-08	1,00467786	0,01991238	0,98411327
VC0594	1,03733955	0,09992923	1,00260491	9,7512E-08	1,00260491	0,01974757	0,98424473
VC1706	1,088906	-0,0422328	-1,006379	-4,121E-08	-1,006379	-0,0196754	0,98430232
VCA1048	1,11184858	0,08315356	1,00390779	8,1142E-08	1,00390779	0,01960195	0,98436091
VCA0071	1,19671637	-0,0172127	-1,0149022	-1,68E-08	-1,0149022	-0,0195426	0,98440822
VC0549	1,08895298	0,52389395	1,00891599	5,1122E-07	1,00891599	0,01921302	0,98467117
VC2235	1,06352841	-0,1024033	-1,0023911	-9,993E-08	-1,0023911	-0,0191537	0,98471853
VCA1000	1,0825431	-0,0299811	-1,0085458	-2,926E-08	-1,0085458	-0,0185648	0,98518829
VC0998	1,0338889	-0,2747815	-1,0046625	-2,681E-07	-1,0046625	-0,0183762	0,98533877
VC1557	1,11075329	0,09285187	1,0044849	9,0606E-08	1,0044849	0,01762234	0,98594013
VCA0268	1,08520222	-0,0348382	-1,0059564	-3,4E-08	-1,0059564	-0,0176166	0,9859447
VCA0263	1,08023809	0,049067	1,00415052	4,788E-08	1,00415052	0,01745998	0,98606966
VCA0650	1,06197724	-0,0145404	-1,0139448	-1,419E-08	-1,0139448	-0,0173793	0,98613404
VC0697	1,10206719	0,15625544	1,00433647	1,5248E-07	1,00433647	0,01663555	0,98672736

VCA0274a	1,0540241	0,08312559	1,00372877	8,1115E-08	1,00372877	0,01654285	0,98680131
VC1401	1,06274666	-0,0246896	-1,0070547	-2,409E-08	-1,0070547	-0,0161354	0,98712639
VC0309	1,06509336	-0,0463642	-1,0037427	-4,524E-08	-1,0037427	-0,0161185	0,98713987
VC1570	1,08081429	0,03679611	1,00660604	3,5906E-08	1,00660604	0,01565237	0,98751174
VC2425	1,02044512	-0,0187072	-1,0085304	-1,825E-08	-1,0085304	-0,0154387	0,98768216
VC1781	1,16543565	0,23972025	1,00907233	2,3392E-07	1,00907233	0,01514725	0,98791472
VC0464	1,07504223	0,03864053	1,00343104	3,7706E-08	1,00343104	0,01408997	0,98875821
VC0494	1,07292345	-0,0449894	-1,0040282	-4,39E-08	-1,0040282	-0,0140248	0,98881016
VCA0364	1,05087634	0,29237172	1,00496065	2,853E-07	1,00496065	0,0139752	0,98884977
VC1034	-1,0074381	-0,4267944	-1,0030641	-4,165E-07	-1,0030641	-0,0137787	0,98900658
VC1535	1,14336059	0,02117602	1,00567514	2,0664E-08	1,00567514	0,0134073	0,98930285
VC2481	-1,0016257	0,16153582	1,00562905	1,5763E-07	1,00562905	0,01297889	0,98964462
VC0333	1,02723314	-0,0755802	-1,0014213	-7,375E-08	-1,0014213	-0,0126899	0,98987521
VC0872	-1,016671	-0,2357448	-1,0041435	-2,3E-07	-1,0041435	-0,0123883	0,9901158
VC2636	1,05924268	0,08405859	1,00262598	8,2025E-08	1,00262598	0,012198	0,99026766
VC1669	1,10283396	-0,0308852	-1,0031686	-3,014E-08	-1,0031686	-0,0121063	0,99034078
VC2237	1,08237273	-0,0616151	-1,0021611	-6,012E-08	-1,0021611	-0,0118538	0,99054222
VC1259	1,10948878	-0,0216603	-1,0040651	-2,114E-08	-1,0040651	-0,0114809	0,99083976
VCA0076	1,13192577	0,02097372	1,00411499	2,0466E-08	1,00411499	0,01136639	0,99093113
VC1857	1,05894076	-0,0483483	-1,0017692	-4,718E-08	-1,0017692	-0,0113225	0,99096614
VC2456	1,06332245	-0,0349813	-1,0021749	-3,414E-08	-1,0021749	-0,010677	0,99148114
alaS	1,00916397	0,13963311	1,00167643	1,3626E-07	1,00167643	0,01055451	0,9915789
VCA0602	1,17484918	-0,0062736	-1,0118709	-6,122E-09	-1,0118709	-0,010538	0,99159203
VCA0293	1,04766239	0,34530534	1,00355661	3,3695E-07	1,00355661	0,01048529	0,99163409
VC1338	1,12864464	0,0161254	1,00506288	1,5735E-08	1,00506288	0,00960331	0,99233779
ddl	-1,0031321	0,16723312	1,0006124	1,6319E-07	1,0006124	0,00941797	0,99248566
VC1618	1,08161032	0,01873386	1,00308408	1,8281E-08	1,00308408	0,00930226	0,99257797
VC1082	1,12909673	0,07280767	1,00371108	7,1046E-08	1,00371108	0,0091252	0,99271924
VC2734	1,09068964	0,03229319	1,00144664	3,1512E-08	1,00144664	0,00836815	0,99332326
VCA0850	1,08497369	0,02122635	1,00217561	2,0713E-08	1,00217561	0,00831839	0,99336298
VCA0365	-1,0252846	-0,015806	-1,0038417	-1,542E-08	-1,0038417	-0,0079499	0,993657
VCA0578	1,11916931	-0,0482281	-1,0020153	-4,706E-08	-1,0020153	-0,0075274	0,99399402
VCA0155	1,11878709	0,01258093	1,00292633	1,2277E-08	1,00292633	0,00742586	0,99407507
VC2498	1,10837675	0,03294449	1,00128694	3,2148E-08	1,00128694	0,00735573	0,99413101
VCA0038	1,11334869	0,05175936	1,00184927	5,0507E-08	1,00184927	0,00718322	0,99426866
VC0350	1,06238985	-0,358565	-1,0009693	-3,499E-07	-1,0009693	-0,0070134	0,99440417
VC2072	1,07696317	0,02031097	1,00159432	1,982E-08	1,00159432	0,00696671	0,99444143
VC1073	1,09106312	0,00899955	1,00353396	8,7818E-09	1,00353396	0,00690088	0,99449394
VCA0994	1,12587625	0,01887031	1,0022485	1,8414E-08	1,0022485	0,00674711	0,99461659
VC2728	1,06462658	-0,0441142	-1,0015664	-4,305E-08	-1,0015664	-0,0065733	0,99475535
VCA0648	1,14356338	-0,0041037	-1,0063249	-4,004E-09	-1,0063249	-0,0062298	0,99502939
VC2499	1,04230423	-0,0369666	-1,0008418	-3,607E-08	-1,0008418	-0,0062262	0,99503226
ndk	1,0330528	-0,5690937	-1,0019459	-5,553E-07	-1,0019459	-0,0061911	0,99506021
VC2512	1,04285733	0,4805176	1,00216046	4,6889E-07	1,00216046	0,00581426	0,99536093
VC2522	1,03591125	-0,0198943	-1,0011547	-1,941E-08	-1,0011547	-0,0057015	0,99545088

VCA0633	1,09536494	-0,0136965	-1,0013832	-1,337E-08	-1,0013832	-0,0053003	0,99577096
VC0197	1,08855439	0,00955857	1,00195633	9,3273E-09	1,00195633	0,00529361	0,9957763
VC2744	-1,0135659	0,12598126	1,00175979	1,2293E-07	1,00175979	0,00487432	0,99611088
VCA0573	1,03370817	-0,251466	-1,0014506	-2,454E-07	-1,0014506	-0,0046357	0,99630128
VC0803	1,06380095	0,01233957	1,00104267	1,2041E-08	1,00104267	0,00439197	0,99649571
tRNA-Asp-2	1,00406231	-0,0376715	-1,0019447	-3,676E-08	-1,0019447	-0,0043471	0,99653153
VC2356	1,00906483	0,11077134	1,00164249	1,0809E-07	1,00164249	0,00431445	0,99655759
VC0681	1,01554558	-0,0458717	-1,0007225	-4,476E-08	-1,0007225	-0,0037763	0,99698696
VC1646	1,21742543	-0,004775	-1,0015746	-4,659E-09	-1,0015746	-0,003357	0,99732154
VC1996	1,08251	-0,0605844	-1,0011192	-5,912E-08	-1,0011192	-0,0033107	0,99735846
VCA1093	1,06284939	-0,0045035	-1,0013321	-4,395E-09	-1,0013321	-0,0029988	0,99760735
VC1975	1,05228408	0,01037715	1,00057631	1,0126E-08	1,00057631	0,0029947	0,99761053
VC0215	1,0340995	0,00863578	1,00058972	8,4269E-09	1,00058972	0,0027635	0,99779507
VC2632	1,13316723	-0,0040577	-1,0011906	-3,96E-09	-1,0011906	-0,0026911	0,99785279
VC0449	1,12104497	-0,004336	-1,0009705	-4,231E-09	-1,0009705	-0,0025118	0,99799589
VCA0798	1,10786615	0,06529161	1,00089541	6,3712E-08	1,00089541	0,00225341	0,99820208
VC1692	1,10506676	0,0034	1,00094679	3,3177E-09	1,00094679	0,0021969	0,99824714
VC2494	1,05103315	0,00555103	1,00054206	5,4167E-09	1,00054206	0,00205637	0,99835928
rnfD	1,05942729	-0,0063379	-1,0002601	-6,185E-09	-1,0002601	-0,0013159	0,99895008
VCA0134	1,07922866	-0,002973	-1,0004006	-2,901E-09	-1,0004006	-0,0012095	0,99903496
VC0939	1,03498128	0,00758364	1,00026088	7,4002E-09	1,00026088	0,00099872	0,99920317
VC1244	1,09405649	0,00169297	1,00023608	1,652E-09	1,00023608	0,00077424	0,99938194
VC0702	1,0173625	0,00628814	1,00011556	6,136E-09	1,00011556	0,0006665	0,99946812
VC2308	1,0715251	-0,0011941	-1,0001338	-1,165E-09	-1,0001338	-0,0004895	0,99960944
greB	1,12643403	0,0001861	1,00002286	1,816E-10	1,00002286	7,8614E-05	0,99993411

Table S2: Function prediction of ncRNA386 using CopaRNA (1), Prodoric virtual footprint (2) and Bprom (3) tools.

(1)

fdr	p-value	NC_002505	NC_011744	NC_009457	Annotation	Additional ho	Amount samp
0,67133365	0,00047039	vca1077(N/A|	vs_rs14880(N	vc0395_rs00830(N/A|-11.88730|0.05013			0
0,67133365	0,00061721	vc1781(N/A|-19.15150|0.0		vc0395_rs122	N-acetylmannosamine-6-pho		1
0,67133365	0,00100862	vc1647(N/A|-	vs_rs20450(N	vc0395_rs11650(N/A|-18.14830|0.00062			0
0,67133365	0,00119614	vc1403(N/A|-12.36740|0.0		vc0395_rs104	methyl-accepting chemotaxi		1
0,67133365	0,00125118	vc1199(N/A|-	vs_rs05685(N	vc0395_rs09455(N/A|-15.36210|0.00529			0
0,67133365	0,00151222	vc2281(N/A|-	vs_rs20455(N	vc0395_rs147	ribosomal protein S6 modifi		0
0,67133365	0,00152885	vc1052(N/A|-	vs_rs04240(N	vc0395_rs08215(N/A|-11.08940|0.07915			0
0,67133365	0,00160577	vc0032(N/A|-	vs_rs14695(N	vc0395_rs177	ComM-like protein		0
0,67133365	0,00178546	vca0179(N/A|	vs_rs16310(N	vc0395_rs051	NupC family protein		0
0,72344254	0,00213783	vc0190(uvrD|	vs_rs14450(u	vc0395_rs181	DNA-dependent helicase II		0
0,76310201	0,00248053	vc1312(alr|-13.84970|0.01		vc0395_rs100	alanine racemase		1
0,81833189	0,00290189	vca0083(N/A|	vs_rs16260(N	vc0395_rs002	multidrug resistance protein		0
0,86989414	0,00348331	vca1015(N/A|	vs_rs21435(N	vc0395_rs011	Na+/H+ antiporter		0
0,86989414	0,00374343	vca0513(N/A|	vs_rs18175(N	vc0395_rs021	aromatic amino acid aminot		0
0,86989414	0,00400451	vc2550(N/A|-	vs_rs13055(N	vc0395_rs15990(N/A|-19.62410|0.00017			0
0,86989414	0,00411297	vca0892(N/A|	vs_rs16845(N	vc0395_rs01670(N/A|-17.69280|0.00090			0
0,96177228	0,00504086	vc2552(N/A|-	vs_rs13060(N	vc0395_rs16000(N/A|-16.60970|0.00211			0
0,96177228	0,00608944	vc1684(N/A|-	vs_rs09365(N	vc0395_rs118	peptide ABC transporter ATP		0
0,96177228	0,00654767	vca0727(N/A|	vs_rs18205(N	vc0395_rs032	cobyric acid synthase		0
0,96177228	0,00688622	vc2003(rrmA	vs_rs04305(rr	vc0395_rs133	23S rRNA (guanine(745)-N(1		0
0,96177228	0,00692035	vca0867(N/A|	vs_rs20750(N	vc0395_rs017	outer membrane protein W		0
0,96177228	0,00733564	vca0988(N/A|-12.05430|0.0		vc0395_rs012	methyl-accepting chemotaxi		1
0,96177228	0,00784734	vc1944(N/A|-10.24010|0.1		vc0395_rs130	pyoverdine biosynthesis pro		1
0,96177228	0,00788481	vca0281(N/A|	vs_rs05730(N	vc0395_rs045	integrase		0

0,96177228	0,00793299	vc0652(N/A|-	vs_rs11630(N	vc0395_rs062	protease		0
0,96177228	0,00793777	vc1044(N/A|-	vs_rs03995(N	vc0395_rs08170(N/A|-11.45410|0.06442			0
0,96177228	0,0079538	vc1125(N/A|-	vs_rs21580(N	vc0395_rs08565(N/A|-13.88420|0.01450			0
0,96177228	0,00851087	vc1228(purT|	vs_rs08025(N	vc0395_rs096	phosphoribosylglycinamide		0
0,96177228	0,00943368	vc1570(N/A|-15.50820|0.0		vc0395_rs112	quinol oxidase subunit II		1
0,96177228	0,01047807	vca0557(N/A|-14.30300|0.(		vc0395_rs023	diguanylate cyclase		1
0,96177228	0,01051466	vc1493(N/A|-10.49660|0.1		vc0395_rs10880(N/A|-10.35260|0.11816			1
0,96177228	0,01055318	vca1028(lamE	vs_rs15560(N	vc0395_rs010	maltoporin		0
0,96177228	0,01070212	vc1246(N/A|-	vs_rs07875(N	vc0395_rs09680(N/A|-13.03100|0.02505			0
0,96177228	0,01115173	vca0753(N/A|	vs_rs18110(N	vc0395_rs03320(N/A|-9.34270|0.197207			0
0,96177228	0,01124335	vca1000(N/A|	vs_rs16515(N	vc0395_rs01175(N/A|-9.53263|0.179732			0
0,96177228	0,01147732	vca1057(N/A|	vs_rs20745(N	vc0395_rs009	short chain dehydrogenase/ı		0
0,96177228	0,01165943	vca0927(N/A|-14.66740|0.(		vc0395_rs01505(N/A|-14.67740|0.00853			1
0,96177228	0,01189884	vca0529(N/A|-11.90870|0.(		vc0395_rs022	potassium uptake protein K		1
0,96177228	0,0126299	vc2309(N/A|-	vs_rs03130(N	vc0395_rs148	class V aminotransferase		0
0,96177228	0,01388258	vc0026(N/A|-	vs_rs00190(N	vc0395_rs177	zinc-binding alcohol dehydr		0
0,96177228	0,01394383	vc1941(N/A|-12.24450|0.0		vc0395_rs13035(N/A|-12.24450|0.04056			1
0,96177228	0,01425891	vc1833(N/A|-	vs_rs05235(N	vc0395_rs125	quinolinate synthetase		0
0,96177228	0,01477909	vc2544(N/A|-	vs_rs13025(N	vc0395_rs159	fructose-1 6-bisphosphatase		0
0,96177228	0,01508287	vca0843(N/A|	vs_rs16440(N	vc0395_rs019	glyceraldehyde-3-phosphate		0
0,96177228	0,01533392	vc2665(groES	vs_rs01095(gı	vc0395_rs165	co-chaperonin GroES		0
0,96177228	0,01538447	vc1821(N/A|-	vs_rs20960(N	vc0395_rs124	PTS system fructose-specific		0
0,96177228	0,01552815	vc2706(N/A|-	vs_rs14410(N	vc0395_rs16735(N/A|-12.26410|0.04009			0
0,96177228	0,01557809	vc0632(N/A|-	vs_rs11740(N	vc0395_rs061	D-alanyl-D-alanine carboxyp		0
0,96177228	0,01566796	vc0474(N/A|-15.16530|0.0		vc0395_rs055	iron-regulated virulence regu		1

0,96177228	0,01567577	vca0774(N/A|	vs_rs18335(N	vc0395_rs034	UDP-glucose 4-epimerase		0
0,96177228	0,01674201	vc1183(N/A|-	vs_rs05580(N	vc0395_rs09380(N/A|-16.04250|0.00323			0
0,96177228	0,01675882	vc0035(N/A|-	vs_rs14680(N	vc0395_rs177	serine/threonine protein kin		0
0,96177228	0,01693918	vc0233(N/A|-	vs_rs00865(N	vc0395_rs183	3-deoxy-D-manno-octulosor		0
0,96177228	0,01698483	vc0300(N/A|-	vs_rs13830(N	vc0395_rs18745(N/A|-9.23014|0.208182			0
0,96177228	0,01726985	vca1067(N/A|	vs_rs05805(N	vc0395_rs008	aldehyde dehydrogenase		0
0,96177228	0,01745259	vc0788(N/A|-	vs_rs19715(N	vc0395_rs069	DOPA-dioxygenase-like prote		0
0,96177228	0,01750979	vc2542(N/A|-	vs_rs13020(N	vc0395_rs159	UDP-N-acetylmuramate:L-ala		0
0,96177228	0,01811381	vc0790(N/A|-16.00690|0.0		vc0395_rs069	transcriptional regulator Cit		1
0,96177228	0,01844746	vc1675(N/A|-	vs_rs09320(N	vc0395_rs117	multidrug resistance protein		0
0,96177228	0,01850848	vca0386(N/A|-11.17030|0.(		vc0395_rs04440(N/A|-11.17030|0.07565			1
0,96177228	0,01888105	vca0217(N/A|-15.47000|0.(		vc0395_rs047	diguanylate cyclase		1
0,96177228	0,01890993	vc2209(vibF|-13.52890|0.0		vc0395_rs143	nonribosomal peptide synth		1
0,96177228	0,01982672	vc1418(N/A|-16.44920|0.0		vc0395_rs10495(N/A|-16.44920|0.00239			1
0,96177228	0,02027173	vca0154(N/A|-11.55830|0.(		vc0395_rs05310(N/A|-11.55830|0.06069			1
0,96177228	0,02027869	vc1063(N/A|-	vs_rs10145(N	vc0395_rs082	acyl-CoA thioesterase II		0
0,96177228	0,02065843	vc1899(N/A|-	vs_rs09760(N	vc0395_rs12835(N/A|-10.06280|0.13750			0
0,96177228	0,02077914	vc2625(N/A|-	vs_rs13480(N	vc0395_rs163	ribulose-phosphate 3-epime		0
0,96177228	0,02111223	vc1111(N/A|-	vs_rs09565(N	vc0395_rs084	adenosylmethionine--8-amir		0
0,96177228	0,02285477	vc2183(N/A|-	vs_rs03440(N	vc0395_rs141	ribose-phosphate pyrophosp		0
0,96177228	0,02301722	vc0117(N/A|-	vs_rs14345(N	vc0395_rs173	protoheme IX synthesis prot		0
0,96177228	0,02323439	vc1664(N/A|-	vs_rs05460(N	vc0395_rs117	ABC transporter substrate-bi		0
0,96177228	0,02339939	vca0837(N/A|	vs_rs03025(N	vc0395_rs019	hemolysin		0
0,96177228	0,0234136	vc0170(N/A|-	vs_rs00325(N	vc0395_rs181	peptide ABC transporter ATP		0
0,96177228	0,02370758	vca0677(N/A|	vs_rs17635(N	vc0395_rs029	nitrate reductase assembly p		0
0,96177228	0,02379802	vc2046(N/A|-	vs_rs04455(N	vc0395_rs13540(N/A|-9.72305|0.163486			0
0,96177228	0,02415393	vc0081(N/A|-	vs_rs00430(N	vc0395_rs17475(N/A|-13.71210|0.01622			0
0,96177228	0,02444667	vc1170(N/A|-	vs_rs09230(N	vc0395_rs093	tryptophan synthase subunit		0
0,96177228	0,02453345	vca0105(N/A|	vs_rs17705(N	vc0395_rs00170(N/A|-9.88.		vca0284	0
0,96177228	0,02455566	vc0396(N/A|-	vs_rs01470(N	vc0395_rs193	LuxR family transcriptional r		0
0,96177228	0,02475606	vc0862(N/A|-11.65160|0.0		vc0395_rs07290(N/A|-11.51890|0.06208			1
0,96177228	0,02481513	vca0936(N/A|	vs_rs16085(N	vc0395_rs01470(N/A|-12.94740|0.02640			0
0,96177228	0,02590472	vca0036(N/A|	vs_rs15640(N	vc0395_rs004	serine/threonine transporte		0
0,96177228	0,02641594	vc2111(N/A|-	vs_rs04160(N	vc0395_rs13845(N/A|-10.88680|0.08856			0
0,96177228	0,02686364	vc0968(N/A|-	vs_rs10740(N	vc0395_rs078	cysteine synthase A		0
0,96177228	0,02728307	vc0674(N/A|-	vs_rs02420(N	vc0395_rs063	prolipoprotein diacylglycery		0
0,96177228	0,0273354	vc2113(N/A|-	vs_rs04150(N	vc0395_rs13855(N/A|-11.68960|0.05626			0
0,96177228	0,0274794	vc0678(N/A|-	vs_rs02450(N	vc0395_rs064	transcriptional activator Hly		0
0,96177228	0,02794084	vca0851(N/A|	vs_rs21700(N	vc0395_rs01870(N/A|-12.16330|0.04258			0
0,96177228	0,02796304	vc1275(N/A|-	vs_rs08960(N	vc0395_rs09820(N/A|-14.68360|0.00849			0
0,96177228	0,02871491	vc0550(N/A|-	vs_rs12110(N	vc0395_rs057	oxaloacetate decarboxylase		0
0,96177228	0,02907195	vc0947(N/A|-	vs_rs03260(N	vc0395_rs077	D-alanyl-D-alanine carboxyp		0
0,96177228	0,02943709	vc1210(N/A|-	vs_rs09170(N	vc0395_rs09510(N/A|-10.74550|0.09568			0
0,96177228	0,03053746	vc1502(yebU	vs_rs06625(ye	vc0395_rs109	16S rRNA (cytosine(1407)-C(		0
0,96177228	0,03070528	vc1780(N/A|-16.16140|0.0		vc0395_rs12270(N/A|-16.16140|0.00296			1
0,96177228	0,03078269	vc1712(N/A|-	vs_rs15620(N	vc0395_rs11960(N/A|-13.20910|0.02240			0
0,96177228	0,03095321	vca0164(N/A|	vs_rs20505(N	vc0395_rs05260(N/A|-10.62300|0.10225			0
0,96177228	0,03127869	vca0650(N/A|	vs_rs13115(N	vc0395_rs02845(N/A|-11.11840|0.07788			0
0,96177228	0,03134949	vc1380(N/A|-	vs_rs08300(N	vc0395_rs10330(N/A|-16.22570|0.00282			0
0,96177228	0,03173774	vc0142a(N/A|-10.90540|0.(		vc0395_rs17200(N/A|-10.90540|0.08765			1
0,96177228	0,03187251	vc0040(N/A|-	vs_rs14660(N	vc0395_rs177	hemolysin		0

fdr	p-value	NC_002505	NC_011744	NC_009457	Annotation	Additional ho	Amount samp
0,7047429	0,0002085	vc0969(N/A|-	vs_rs10735(N	vc0395_rs078	sulfate transport protein Cys		0
0,72258242	0,00066371	vca0535(N/A|-16.45890|0.0		vc0395_rs02255(N/A|-16.28460|0.00113			1
0,72258242	0,00080215	vca1028(lamE	vs_rs15560(N	vc0395_rs010	maltoporin		0
0,72258242	0,00093523	vc0734(N/A|-	vs_rs16065(N	vc0395_rs066	malate synthase		0
0,72258242	0,00181617	vc2269(N/A|-	vs_rs11335(N	vc0395_rs146	3 4-dihydroxy-2-butanone-4		0
0,72258242	0,00203364	vc0299(N/A|-	vs_rs13835(N	vc0395_rs187	DNA polymerase III subunit e		0
0,72258242	0,00220172	vc2120(flhB|-	vs_rs03870(N	vc0395_rs138	flagellar biosynthesis protein		0
0,72258242	0,00235208	vc2551(N/A|-18.02100|0.0		vc0395_rs15995(N/A|-17.65710|0.00028			1
0,72258242	0,00247155	vc1571(N/A|-15.54290|0.0		vc0395_rs112	quinol oxidase subunit I		1
0,72258242	0,00276011	vc2341(N/A|-	vs_rs11525(N	vc0395_rs149	long-chain-fatty-acid--CoA li		0
0,72258242	0,00288888	vca1091(N/A|-12.39940|0.0		vc0395_rs007	chemotaxis protein CheR		1
0,72258242	0,00325645	vc1045(N/A|-	vs_rs04000(N	vc0395_rs081	RNA polymerase sigma factor		0
0,72258242	0,00340164	vc2678(N/A|-	vs_rs13625(N	vc0395_rs166	primosome assembly protein		0
0,72258242	0,00351211	vc1330(N/A|-19.06540|5.6		vc0395_rs10090(N/A|-19.06540|5.61605			1
0,72258242	0,00376348	vc2112(N/A|-	vs_rs04155(N	vc0395_rs13850(N/A|-13.90110|0.00868			0
0,72258242	0,00384544	vc1124(N/A|-	vs_rs21575(N	vc0395_rs08560(N/A|-13.88420|0.00880			0
0,72258242	0,0039303	vc0234(N/A|-15.39800|0.0		vc0395_rs18370(N/A|-15.39800|0.00253			1
0,72258242	0,0039557	vc1683(N/A|-	vs_rs09360(N	vc0395_rs118	peptide ABC transporter ATP		0
0,72258242	0,00406185	vca1073(N/A|	vs_rs14895(N	vc0395_rs008	bifunctional proline dehydro		0
0,76229193	0,00501556	vc0795(N/A|-15.37000|0.0		vc0395_rs069	citrate/sodium symporter		1
0,76229193	0,00506414	vc2714(ompR	vs_rs00700(o	vc0395_rs167	osmolarity response regulato		0
0,76229193	0,00511154	vc1888(N/A|-14.59300|0.0		vc0395_rs127	hemolysin-like protein		1
0,76229193	0,00518719	vc1417(N/A|-16.33530|0.0		vc0395_rs10490(N/A|-16.33530|0.00108			1
0,77508134	0,00574572	vc0475(N/A|-	vs_rs07620(N	vc0395_rs055	enterobactin receptor prote		0
0,77508134	0,00575849	vc1328(mglC|-14.15800|0.		vc0395_rs100	beta-methylgalactoside tran		1
0,77508134	0,00638798	vca0621(N/A|	vs_rs17005(N	vc0395_rs027	SorC family transcriptional r		0
0,77508134	0,00661863	vc0653(N/A|-	vs_rs11625(N	vc0395_rs062	c-di-GMP phosphodiesterase		0
0,77508134	0,00682871	vca0223(N/A|	vs_rs19785(N	vc0395_rs047	protease		0
0,77508134	0,00684278	vc2454(N/A|-11.63590|0.0		vc0395_rs155	GGDEF family protein		1
0,77508134	0,00687942	vc2308(N/A|-13.40890|0.0		vc0395_rs148	4-methyl-5(B-hydroxyethyl)-		1
0,81772983	0,00749989	vc0861(N/A|-	vs_rs03015(N	vc0395_rs072	type IV pilin		0
0,8353599	0,0087217	vc1317(N/A|-12.93670|0.0		vc0395_rs10025(N/A|-12.93670|0.01787			1
0,8353599	0,00884169	vc2046(N/A|-	vs_rs04455(N	vc0395_rs13540(N/A|-13.56780|0.01121			0
0,8353599	0,00895575	vca0090(N/A|	vs_rs15250(N	vc0395_rs00245(N/A|-13.59600|0.01097			0
0,8353599	0,00923794	vca0104(N/A|	vs_rs20410(N	vc0395_rs001	ribosomal large subunit pseu		0
0,8353599	0,00966643	vc2337(N/A|-	vs_rs21270(N	vc0395_rs149	LacI family transcription reg		0
0,8353599	0,0099177	vc1820(N/A|-	vs_rs20955(N	vc0395_rs124	PTS system fructose-specific		0
0,8353599	0,00992829	vc0605(N/A|-	vs_rs11875(N	vc0395_rs06035(N/A|-10.83440|0.07263			0
0,8353599	0,01011842	vca0676(N/A|	vs_rs17640(N	vc0395_rs029	(Fe-S)-binding protein		0
0,8353599	0,010179	vc2489(N/A|-	vs_rs01755(N	vc0395_rs156	TetR family transcriptional r		0
0,8353599	0,0104376	vc1612(N/A|-	vs_rs07835(N	vc0395_rs114	fimbrial biogenesis and twitc		0
0,8353599	0,01060633	vc1557(N/A|-	vs_rs15270(N	vc0395_rs111	LacI family transcription reg		0
0,8353599	0,01062736	vc2318(N/A|-14.64220|0.0		vc0395_rs14865(N/A|-14.49120|0.00543			1
0,89523845	0,01165399	vc0332(hemE	vs_rs13925(h	vc0395_rs189	uroporphyrinogen decarbox		0
0,90699675	0,01237922	vc1274(N/A|-	vs_rs08965(N	vc0395_rs09815(N/A|-14.89070|0.00391			0
0,90699675	0,01283052	vc1354(N/A|-	vs_rs06545(N	vc0395_rs10200(N/A|-12.12710|0.03150			0
0,90699675	0,01310509	vca0471(N/A|-13.00440|0.0		vc0395_rs03845(N/A|-13.00440|0.01701			1

0,90699675	0,0133838	vc2421(N/A|-	vs_rs11990(N	vc0395_rs153	N-acetyl-anhydromuranmyl-		0
0,90699675	0,01356139	vc2103(N/A|-12.78870|0.0		vc0395_rs138	LysR family transcriptional re		1
0,90699675	0,01365468	vc0506(N/A|-	vs_rs07755(N/A|-7.67656|0.2552526|148|159|166|17				1
0,90699675	0,01368545	vc1105(N/A|-	vs_rs04655(N/A|-13.13910|0.007982914|183|212|149				1
0,91070222	0,0140108	vc0118(N/A|-	vs_rs14340(N	vc0395_rs173	uroporphyrin-III C-methyltra		0
0,91806554	0,0143957	vc0191(N/A|-	vs_rs14435(N	vc0395_rs18165(N/A|-12.77340|0.02009			0
0,93732673	0,01508615	vc1193(N/A|-	vs_rs05655(N	vc0395_rs09425(N/A|-11.13730|0.06018			0
0,93732673	0,01525236	vc2710(N/A|-	vs_rs00715(N	vc0395_rs167	bifunctional (p)ppGpp synth		0
0,94474656	0,01573798	vca0747(glpA|-12.92320|0		vc0395_rs032	sn-glycerol-3-phosphate deh		1
0,94474656	0,01624288	vca0150(def|-12.12540|0.0		vc0395_rs053	peptide deformylase		1
0,94474656	0,0163942	vc0509(N/A|-	vs_rs07735(N/A|-17.04020|0.0003319843|6|49|149|1				1
0,94474656	0,01649114	vc1165(N/A|-	vs_rs05150(N	vc0395_rs09025(N/A|-11.65510|0.04316			0
0,94795314	0,01735956	vca0832(N/A|	vs_rs16950(N	vc0395_rs019	methyltransferase		0
0,94795314	0,01736028	vca0852(N/A|	vs_rs21705(N	vc0395_rs01865(N/A|-12.16330|0.03073			0
0,94795314	0,01738849	vc2690(fieF|-	vs_rs13700(fi	vc0395_rs166	ferrous iron efflux protein F		0
0,95877982	0,01816075	vca0937(N/A|	vs_rs15875(N	vc0395_rs014	AraC family transcriptional r		0
0,95877982	0,01861071	vc1435(N/A|-	vs_rs06870(N	vc0395_rs10585(N/A|-9.00220|0.204151			0
0,95877982	0,01878804	vc0549(N/A|-	vs_rs12115(N	vc0395_rs057	oxaloacetate decarboxylase s		0
0,95877982	0,01895165	vc2472(N/A|-	vs_rs12390(N	vc0395_rs15590(N/A|-11.64280|0.04351			0
0,95877982	0,0190054	vc0036(N/A|-	vs_rs14675(N	vc0395_rs177	FixG-like protein		0
0,96551865	0,01999628	vc2182(ipk|-9	vs_rs03445(N	vc0395_rs141	4-diphosphocytidyl-2C-meth		0
0,96551865	0,02023693	vc0188(N/A|-	vs_rs00375(N	vc0395_rs181	oligopeptidase A		0
0,96551865	0,02035321	vc0628(N/A|-	vs_rs11765(N	vc0395_rs06150(N/A|-13.84430|0.00907			0
0,96551865	0,02082078	vc0452(N/A|-	vs_rs12680(N	vc0395_rs053	A/G-specific adenine glycosy		0
0,96551865	0,02101114	vc1166(aspS|	vs_rs05155(N	vc0395_rs090	aspartyl-tRNA synthetase		0
0,96551865	0,02144085	vc2001(N/A|-	vs_rs04315(N	vc0395_rs13305(N/A|-12.55370|0.02346			0
0,96551865	0,02160732	vca0651(N/A|	vs_rs13110(N	vc0395_rs02850(N/A|-11.08730|0.06210			0
0,96551865	0,02226931	vc0755(N/A|-	vs_rs02815(N	vc0395_rs067	aminopeptidase		0
0,96551865	0,02245986	vca0933(N/A|	vs_rs00795(N	vc0395_rs014	cold shock do	vca0166	0
0,96551865	0,0228798	vc0142a(N/A|-11.37390|0.0		vc0395_rs17200(N/A|-11.37390|0.05178			1
0,96551865	0,02334764	vc2119(N/A|-	vs_rs04025(N	vc0395_rs13880(N/A|-11.52910|0.04685			0
0,96551865	0,02336704	vc0282(N/A|-13.18480|0.0		vc0395_rs185	methyl-accepting chemotaxi		1
0,96551865	0,02354968	vca0653(N/A|-12.26510|0.0		vc0395_rs028	PTS system sucrose-specific t		1
0,96551865	0,02363142	vc2094(N/A|-	vs_rs10550(N	vc0395_rs13770(N/A|-11.16730|0.05905			0
0,96551865	0,02364395	vc1521(N/A|-	vs_rs07010(N	vc0395_rs110	sensor histidine kinase		0
0,96551865	0,02399223	vc1544(N/A|-	vs_rs18015(N	vc0395_rs111	TonB2 protein		0
0,96551865	0,02399514	vc0673(N/A|-	vs_rs02415(N	vc0395_rs06375(N/A|-10.29960|0.10009			0
0,96619257	0,02459458	vca0245(N/A|	vs_rs16290(N	vc0395_rs046	PTS system transporter subu		0
0,96619257	0,02476053	vc2552(N/A|-	vs_rs13060(N	vc0395_rs16000(N/A|-14.04000|0.00779			0
0,96619257	0,02498759	vca0742(N/A|-12.41760|0.0		vc0395_rs03270(N/A|-13.96260|0.00828			1
0,96619257	0,02578974	vc2380(N/A|-	vs_rs02190(N	vc0395_rs151	adenosylcobinamide-phosph		0
0,96619257	0,02613617	vc1180(N/A|-	vs_rs05545(N	vc0395_rs093	cysteine/glutathione ABC tra		0
0,96619257	0,02616266	vca0971(N/A|	vs_rs21370(N	vc0395_rs01310(N/A|-11.40510|0.05076			0
0,96619257	0,02664766	vca1037(N/A|	vs_rs17335(gl	vc0395_rs010	amino acid ABC transporter A		0
0,96619257	0,02703454	vc0622(N/A|-	vs_rs11795(N	vc0395_rs061	sensory box sensor histidine		0
0,96619257	0,02708075	vc1171(N/A|-	vs_rs09225(N	vc0395_rs093	bifunctional indole-3-glycero		0
0,96619257	0,02724277	vca0475(N/A|-8.43769|0.20		vc0395_rs038	doc protein		1
0,96619257	0,02736236	vc2771(N/A|-	vs_rs14780(N	vc0395_rs179	F0F1 ATP synthase subunit I		0
0,96619257	0,02744216	vc1581(N/A|-11.29700|0.0		vc0395_rs113	NADH dehydrogenase subun		1
0,96941656	0,02823143	vc1356(N/A|-	vs_rs06560(N	vc0395_rs102	sulfite reductase subunit gan		0
0,96941656	0,02845592	vc1184(N/A|-	vs_rs05585(N	vc0395_rs093	NifS-like protein		0
0,96941656	0,02925497	vc0046(def|-8	vs_rs14630(N	vc0395_rs176	peptide deformylase		0
0,96941656	0,02941438	vca0785(N/A|	vs_rs18380(N	vc0395_rs034	diguanylate cyclase		0

(2)

Matrix	Position	Strand	Binding Seque	Score	Core Score	Locus (Gene)	Dist to ATG	Product
MX000366	18	+	AGCGAGCCAA	5.124725521	3.712140441	undefined (un	undefined	undefined
MX000366	81	-	TTAGATCCAGT	4.889834628	3.712140441	undefined (un	undefined	undefined
MX000366	48	+	TGTGATCCTAC	4.794736798	3.567173032	undefined (un	undefined	undefined
MX000366	20	+	CGAGCCAAAT	4.359936236	2.852052152	undefined (un	undefined	undefined
MX000366	43	-	GTAGGATCAC	4.211242630	3.307393011	undefined (un	undefined	undefined
MX000366	168	*	TACGATTTACC	4.188143612	3.225760358	undefined (un	undefined	undefined

(3)

For promoter at 64:
ompR: AAATCACA at position 27 Score - 7
crp: ATCACAAA at position 29 Score - 10
arcA: TCACAAAA at position 30 Score - 10
ihf: ACAAAAAA at position 32 Score - 9

Table S3: Strains, plasmids and primers

**Strain number**	**Phenotype of interest**
Acinetobacter baumannii ATCC 19606	WT
E. coli MG1655	WT
Klebsiella pneumoniae ML21	WT
Pseudomonas aeruginosa ATCC 10145	WT
Vibrio cholerae N16961	WT
V. tasmaniensis	WT
V. mimicus	WT
V. parahaemolyticus	WT
V. vulnificus	WT
D908	N16961 ΔctrR clean deletion (formerly known as Δ ncRNA586)
F862	*V. cholerae N16961 with* PctrRVch plasmid that contains ctrR-pBAD24
F863	*V. cholerae N16961* with PctrRVta plasmid containing VsR_217-pBAD24
F864	V. cholerae N16961 with empty-pBAD24
H114/H115	V. cholerae N16961 with PctrRVch-CC plasmid that contains the mutated version ctrR-CC-pBAD24
K830	ΔVC1820-28 clean deletion using plasmid A200 and oligos 5VC181, 7VC181, 8VC181, 6VC181 that amplify 500 bp upstream and dowstream from VC1820-28 locus
L147	ΔlamB, clean deletion using plasmid A200 and oligos 5lamB, 7lamB, 8lamB, 6lamB that amplify 500 bp upstream and dowstream from the maltoporin gene
L148	Δmalop, clean deletion using plasmid A200 and oligos 5malop1, 7malop1, 8malop1, 6malop1 that amplify 500 bp upstream and dowstream from the maltose transport operon
Top 10	F- mcrA Δ(mrr-hsdRMS-mcrBC) Φ80lacZΔM15 Δ lacX74 recA1 araD139 Δ(ara-leu)7697 galU galK rpsL (StrR) endA1 nupG
π3813 cells	To grow plasmids for mutant construction before allelic exchange
β3914	β2163 [(F-) RP4-2-Tc::Mu ΔdapA::(erm-pir116)] gyrA462 zei-298::Tn10

**Plasmid**	**Description**
pF585	pBAD24 with ctrRVch cloned for Ara overexpression
pF587	pBAD24 with ctrRVta cloned for Ara overexpression
pBAD24	ori pBR322, carbenicillin resistance
A200	Flipase continaing plasmid
pSW7848	Plasmid contining frt sites surrounding the aph gene for allelic exchange of target genes
pSC1819	Plasmid containing the mariner tranposon and a spec resistance gene for saturated mutant library generation

**Oligo name**	**Sequence**	**Use**
5malop1	CTATTATTTAAACTCTTTCCCAACCACTGGGATGGAGCG	Amplification of the 500 bp upstream of the maltose operon for clean deletion
7malop1	CTACACAATCGCTCAAGACGTGTGGCGTTACGCATAGCTGGC	Amplification of the 500 bp upstream of the maltose operon for clean deletion
8malop1	CTAATTCCCATGTCAGCCGTTCCTCCTAACCCCTACGCC	Amplification of the 500 bp downstream of the maltose operon for clean deletion
6malop1	TACGTAGAATGTATCAGACTATCAAGTGGTTTCGTTGGCAGCG	Amplification of the 500 bp downstream of the maltose operon for clean deletion
5vc181	CTATTATTTAAACTCTTTCCACCTCGCCTACCGTATGG	Amplification of the 500 bp upstream

		of the VC1820-27 locus for clean deletion
7vc181	CTACACAATCGCTCAAGACGTGTTCTTGAAGTGGCAGCGG	Amplification of the 500 bp upstream of the VC1820-27 locus for clean deletion
8vc181	CTAATTCCCATGTCAGCCGTGCACATATCGAGGAAATAGCCG	Amplification of the 500 bp downstream of the VC1820-27locus for clean deletion
6vc181	TACGTAGAATGTATCAGACTTATTCGTCATGCAGCGGC	Amplification of the 500 bp downstream of the VC1820-27 locus for clean deletion
5lamB	CTATTATTTAAACTCTTTCCAGAAGGACCAGAGACATCCAGC	Amplification of the 500 bp upstream of the lamB gene for clean deletion
7lamB	CTACACAATCGCTCAAGACGTGAGCGGGTTGTCAGAGTACAGC	Amplification of the 500 bp upstream of the lamB gene for clean deletion
8lamB	CTAATTCCCATGTCAGCCGTAGGCAGAAAGTTGGCAGC	Amplification of the 500 bp downstream of the lamB gene for clean deletion
6lamB	TACGTAGAATGTATCAGACTAATGCAATTCATCATTGAGCTGG	Amplification of the 500 bp downstream of the lamB gene for clean deletion
crpfix500F	TTGTAGGGCTCACCAAGAGG	Amplification of the ctrR promoter region for EMSA
crpfix500R	ACATCAAGTGGTTTCGTTGGC	Amplification of the ctrR promoter region for EMSA
micX500F	ACCAACCGCAAGCGTGTACG	Amplification of the micX promoter region for EMSA
micX500R	TGCTGATGGTTCAGTCACTCCACC	Amplification of the micX promoter region for EMSA
MV268	CACGTCTTGAGCGATTGTGTAG	For amplification of construction verification of pSW7848 plasmid for clean deletion of different mutants constructed
MV269	ACGGCTGACATGGGAATTAG	For amplification of construction verification of pSW7848 plasmid for clean deletion of different mutants constructed
MV450	GGAAAGAGTTTAAATAATAGTGCAGGAATTCGATATCAAG	For amplification of construction verification of pSW7848 plasmid for clean deletion of different mutants constructed
MV451	AGTCTGATACATTCTACGTAGGGATCCACTAGTTCTAGAG	For amplification of construction verification of pSW7848 plasmid for clean deletion of different mutants constructed
4347	TCTCCATATCCCAGAGAGGATGGTAAGAGCCTTTCC	Amplification of the 197 bp containing ctrR sequence from V. cholerae
4348	TATTTGATGAAAAGAAAGGGTGAACGCTCCGGC	Amplification of the 197 bp containing ctrR sequence from V. cholerae
4343	CTGGGATATGGAGAAACAGTAGAGAGTTGCGATAAAAAGC	Amplification of pBAD24 plasmid for cloning ctrR-Vch
4344	TTCTTTTCATCAAATAAAACGAAAGGCTCAGTCGAAAG	Amplification of pBAD24 plasmid for cloning ctrR-Vch
ML 435	CGCGAATTCATCCAAGAGTGGATAGTAAGAACCC	Amplification Vsr217-Vta
ML 436	CGCTCTAGATTAACCAAGCCCACCCGTCATAGCC	Amplification VsR217-Vta
ML466	cgctctagaCACGGGACTGCCGCGAAAAGTAGG	Point mutation in ctrR
dRT-PCR		
ML 455	[FAM] CTGGTTCCACCGCCAAGTAGGTTGT [BHQ1]	Probe
ML 456	GTTGTTTCCCCCTACACAAAG	Reverse
ML 457	CCCAGAGAGGATGGTAAGAG	forward
